# Precise targeting of transcriptional co-activators YAP/TAZ annihilates chemoresistant brCSCs by alteration of their mitochondrial homeostasis

**DOI:** 10.1038/s41392-025-02133-x

**Published:** 2025-02-21

**Authors:** Priyanka Dey Talukdar, Kunal Pramanik, Priya Gatti, Pritha Mukherjee, Deepshikha Ghosh, Himansu Roy, Marc Germain, Urmi Chatterji

**Affiliations:** 1 Cancer Research Laboratory, Department of Zoology, University of Calcutta, Kolkata, West Bengal India; 2 Groupe de Recherche en Signalisation Cellulaire and Département de Biologie Médicale, Université du Québec à Trois-Rivières, Trois-Rivières, QC Canada; 3 CSIR-Indian Institute of Chemical Biology, Kolkata, India; 4 Department of Surgery, Medical College, Kolkata, India

**Keywords:** Cancer stem cells, Cancer stem cells

## Abstract

Persistence of drug-resistant breast cancer stem cells (brCSCs) after a chemotherapeutic regime correlates with disease recurrence and elevated mortality. Therefore, deciphering mechanisms that dictate their drug-resistant phenotype is imperative for designing targeted and more effective therapeutic strategies. The transcription factor SOX2 has been recognized as a protagonist in brCSC maintenance, and previous studies have confirmed that inhibition of SOX2 purportedly eliminated these brCSCs. However, pharmacological targeting of transcription factors like SOX2 is challenging due to their structural incongruities and intrinsic disorders in their binding interfaces. Therefore, transcriptional co-activators may serve as a feasible alternative for effectively targeting the brCSCs. Incidentally, transcriptional co-activators YAP/TAZ were found to be upregulated in CD44^+^/CD24^-^/ALDH^+^ cells isolated from patient breast tumors and CSC-enriched mammospheres. Interestingly, it was observed that YAP/TAZ exhibited direct physical interaction with SOX2 and silencing *YAP/TAZ* attenuated SOX2 expression in mammospheres, leading to significantly reduced sphere forming efficiency and cell viability. YAP/TAZ additionally manipulated redox homeostasis and regulated mitochondrial dynamics by restraining the expression of the mitochondrial fission marker, DRP1. Furthermore, YAP/TAZ inhibition induced DRP1 expression and impaired OXPHOS, consequently inducing apoptosis in mammospheres. In order to enhance clinical relevance of the study, an FDA-approved drug verteporfin (VP), was used for pharmacological inhibition of YAP/TAZ. Surprisingly, VP administration was found to reduce tumor-initiating capacity of the mammospheres, concomitant with disrupted mitochondrial homeostasis and significantly reduced brCSC population. Therefore, VP holds immense potential for repurposing and decisively eliminating the chemoresistant brCSCs, offering a potent strategy for managing tumor recurrence effectively.

## Introduction

Comprehending the progression of malignancies requires extrapolation of the mechanisms by which cancer stem cells (CSCs) exploit the differentially regulated cellular proteome to steer tumor development.^1^ Considerable expanse of studies in this area have underscored the involvement of multifactorial remoulding.^2^ Among these, DNA-binding transcription factors (TFs) emerged as pivotal players at the core of the transcriptional regulatory networks responsible for controlling the expression of genes crucial for defining cell identity.^3^ However, directly targeting these fundamental drivers is difficult, owing to the intrinsic disorder in their binding interfaces.^4^ Therefore, transcriptional co-activators which oversee the functionality of TFs by coordinating ATP-dependent chromatin remodelling and post translational modifications can be considered as alternate targets for modulation.^5^ Transcriptional co-activators are acknowledged as evolutionarily conserved elements, stringently regulating signaling pathways and upholding metabolic and genomic stability.^5,6^ In recent years, considerable attention has been directed towards the downstream effectors of the Hippo signaling pathway, viz., the transcriptional co-activators YAP (yes-associated protein) and TAZ (transcriptional co-activator with PDZ-binding motif), owing to their potential role in regulating organ growth, amplifying tissue-specific progenitor cells during renewal and regeneration, and facilitating cell proliferation. Moreover, their constitutive persistence and retention in the cells have been reported to integrate intracellular and extracellular oncogenic cues, thereby promoting tumorigenesis.^7^ In addition, emerging studies suggested the association of YAP/TAZ with stemness.^8–10^ However, there are few systematic analyses regarding their mechanisms in regulating stemness within breast cancer stem cells (brCSCs).^11,12^

It is well-established that stem cells maintain low levels of intracellular reactive oxygen species (ROS), a redox state critical for regulating stem cell quiescence and self-renewal. While limited studies have explored the role of redox dynamics in the CSC biology, the resistance of CSCs to conventional anticancer therapies has been increasingly linked to the upregulation of ROS-scavenging mechanisms.^13^ A pivotal factor in this process is the nuclear factor erythroid 2-related factor 2 (NRF2), a master regulator of cellular redox homeostasis, which acts in conjunction with its negative regulator KEAP1, by enhancing the expression of antioxidant enzymes.^14^ It has also been demonstrated that mammospheres derived from the breast cancer cell lines, MCF7 and MDA-MB-231, exhibited reduced levels of ROS compared to their monolayer counterparts.^15^ Furthermore, elevated ROS levels in NRF2-deficient mammospheres resulted in impaired sphere growth and induction of apoptosis.^16^ Considering the critical role of NRF2 in CSCs and accumulating evidence emphasizing the association between NRF2 and the transcriptional co-regulators YAP/TAZ,^17,18^ we sought to investigate the multifaceted role of YAP/TAZ in modulating key CSC properties in breast cancer.

Mitochondria play a central role in regulating redox signaling and maintaining redox homeostasis in both normal and cancer cells. By integrating metabolic, bioenergetic, and redox signals, the mitochondrial network functions as a critical hub, influencing a broad range of cellular processes.^19^ Increasing evidence has suggested the dependence of CSCs on mitochondrial metabolism for their sustenance, which is often designated as ‘mitostemness’.^20^ Mitochondria exist along a dynamic continuum between fragmented and elongated stage, which is orchestrated by constant cycling between two opposing yet essential pathways, mitochondrial fission and fusion.^21^ CSCs manipulate these dynamics to regulate physiological and biochemical processes, enabling them to adapt and maintain homeostasis, even in an unfavourable milieu.^22^ In light of these observations, it is essential to explore the mitochondrial dynamics-driven potential therapeutic vulnerabilities. Identification of such jeopardies could have a pronounced impact in disrupting CSCs across several cancer types.

In this study, we attempted to investigate the involvement of YAP/TAZ in stemness maintenance and mitochondrial rheostasis in breast cancer. Our findings elucidated that YAP, TAZ, and SOX2 worked in tandem, and silencing *YAP/TAZ* reduced the expression of SOX2 and its downstream target NANOG, indicating that YAP/TAZ/SOX2 formed a tripartite complex where YAP/TAZ possibly functioned as co-activators of SOX2. Subsequently, it was also observed that YAP/TAZ directly interacted with the mitochondrial fission protein DRP1 to fine-tune its expression in mammospheres. Moreover, silencing *YAP/TAZ* in mammospheres disrupted mitochondrial respiration, reduced sphere-forming efficiency across generations, and induced apoptosis. Since gene silencing to drug implementation is a prolonged and uncertain path, we searched for drugs having similar inhibitory effects and which could be repurposed for combinatorial therapy in the clinics. Verteporfin (VP), a benzoporphyrin derivative, is conventionally recognized as an inhibitor of YAP/TAZ, primarily by preventing the binding of YAP/TAZ to TEAD through its interaction with the TEAD-binding domain (TBD).^23^ Administration of VP in mammospheres yielded similar results, with reduced brCSC population and diminished tumor-initiating potential, further conforming to the observed paradigm. Together, this study positions YAP and TAZ as potential master regulators of the brCSC regulatory blueprint, offering insights into the potential of rescripting YAP/TAZ expression, to overcome refractoriness to therapies and abrogate the brCSC population for complete remission and good patient prognosis.

## Results

### Transcriptional co-activators YAP/TAZ interacts with transcription factor SOX2 in breast cancer

**Fig. 1:**
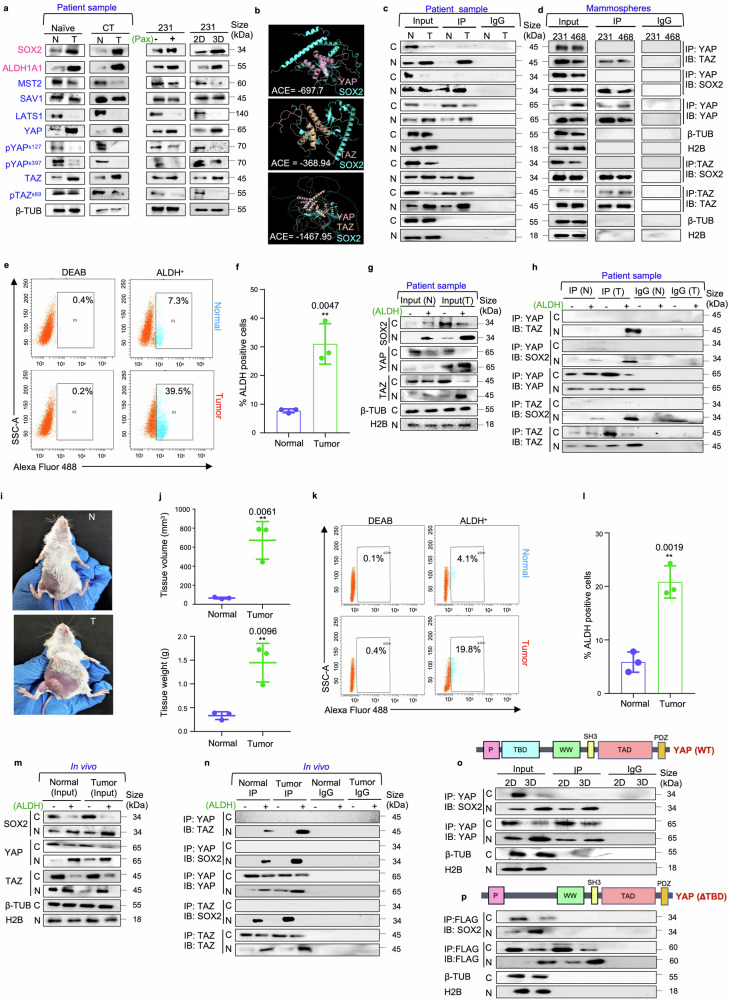
**YAP/TAZ transcriptional co-activators interact with SOX2 transcription factor in triple negative breast cancer. a** Western blot representations depicting the expression of the markers related to the Hippo signaling pathway and stemness in naïve (*n* = 10), chemo-treated patient breast tumors (*n* = 10), paclitaxel-treated MDA-MB-231 cells (*n* = 3) and MDA-MB-231 mammospheres (*n* = 3) in comparison to adjacent normal breast tissue, untreated MDA-MB-231 cells and adherent culture of MDA-MB-231 respectively. Markers associated with stemness are indicated in pink, while markers related to the Hippo signaling pathway are denoted in blue. **b** Molecular docking model depicting the SOX2/YAP, SOX2/TAZ and SOX2/YAP/TAZ complexes. **c** Co-immunoprecipitation (Co-IP) analyses using either control IgG or antibodies against YAP and TAZ. Western blot analyses were performed to analyse differential interaction pattern in the cytoplasm and nucleus of patient breast tumors in comparison to their adjacent normal (*n* = 5) **d** Co-immunoprecipitation (Co-IP) analyses using either control IgG or antibodies against YAP and TAZ. Western blot analyses were performed to analyse differential interaction pattern in the cytoplasm and nucleus of mammospheres (*n* = 3). **e** Gating strategy for flow cytometry-based isolation of ALDH^+^ and ALDH^−^ cell population from patient breast tumors and respective adjacent normal tissues (*n* = 3). **f** Graphical representation of ALDH activity in patient breast tumors compared to their respective adjacent normal tissues (*n* = 3). **g** Western blot analyses depicting the expression of SOX2, YAP and TAZ in cytoplasm and nucleus of ALDH^+^ and ALDH^−^ cell population (*n* = 3). **h** Co-immunoprecipitation experiments using either control IgG or anti-YAP/anti-TAZ antibodies, followed by western blot analyses in the ALDH^+^ and ALDH^−^ cells isolated from patient breast tumors and respective adjacent normal tissues (*n* = 3). **i** Female BALB/c mice were inoculated with 1 × 10^4^ 4T1 cells and allowed to develop tumors for 14 days. The control group received vehicle control treatment for the same duration. After the incubation period, **j** Volume and weight of mammary tissues of both the group of mice were assessed and compared (*n* = 3). **k** Gating strategy for flow cytometry-based isolation of ALDH^+^ and ALDH^−^ cell population from normal mammary tissue and 4T1-induced mammary tumor-bearing mice (*n* = 3). **l** Graphical representation of ALDH activity in normal mammary tissue and 4T1-induced mammary tumor-bearing mice (*n* = 3). **m** Western blot analyses depicting the expression of SOX2, YAP and TAZ in cytoplasm and nucleus of ALDH^+^ and ALDH^−^ cell population (*n* = 3). **n** Co-immunoprecipitation experiments using either control IgG or anti-YAP/anti-TAZ antibodies, followed by western blot analyses in the ALDH^+^ and ALDH^−^ cells isolated from normal and 4T1- induced mammary tumor bearing mice (*n* = 3). **o** Co-immunoprecipitation experiments using either control IgG or anti-YAP antibodies, followed by western blot analyses in the adherent and mammosphere culture of MDA-MB-231 (*n* = 3). **p** *YAP* deletion mutant (ΔTBD) was transfected into MDA-MB-231 adherent cells and mammospheres. Co-immunoprecipitation experiments were performed with anti-FLAG antibody and immunoblots were probed with anti-SOX2 or anti-FLAG antibodies (*n* = 3). Cytosolic and nuclear protein expressions were normalized against β-tubulin and H2B respectively. The data are presented as the mean ± standard deviation (SD), with “n” representing the number of biological replicates per experimental group. Significance was assessed using an unpaired Student’s *t*-test, and the associated two-tailed *p*-value is indicated in the bar plots. Compared to the control group: **p < 0.01. N Normal, T Tumor, CT Chemo-treated, 2D Adherent cells, 3D Mammospheres, 231, MDA-MB-231; ACE Atomic contact energy, C Cytoplasm, N Nucleus, β-TUB β-tubulin, 3D, Mammospheres IP Immunoprecipitate, IB Immunoblot, ALDH Aldehyde dehydrogenase, BAAA BODIPY-aminoacetaldehyde, BAA BODIPY-aminoacetate

Under normal conditions, activated Hippo pathway blocks YAP/TAZ.^24^ A gene profiler array in our lab^25^ indicated that *YAP/TAZ* was significantly upregulated and *LATS1* was downregulated in brCSCs isolated from chemo-treated patients with triple-negative breast tumors. Analyses of Hippo pathway markers in patient samples revealed that compared to their respective normal tissues, enhanced expressions of YAP/TAZ and the stemness markers, SOX2 and ALDH1A1, were observed in both naïve and chemotherapy-treated breast tumors. Concurrently, downregulation of total endogenous levels of MST2, LATS1 and the phosphorylated forms of YAP/TAZ was observed, indicating deregulation of Hippo signaling pathway in breast tumors. However, no significant alteration was observed in the expression of SAV1 (Fig. 1a; Supplementary Fig. S1a, b). Furthermore, compared to naïve tumors, higher expression of SOX2, YAP, and TAZ was observed in chemo-treated patient breast tumors (Supplementary Fig. S1c, d). To simulate the chemo-treatment condition in vitro, triple-negative breast cancer (TNBC) cells MDA-MB-231 and MDA-MB-468 were treated with 2 nM paclitaxel for 24 hours. This dose was selected based on our previous study as treatment with this particular dose enhanced the expression of stemness markers without significantly affecting the cell viability.^25^ Similar to the chemo-treated patient tumors, treatment of breast cancer cell lines with the chemo-therapeutic drug paclitaxel increased the expression of SOX2, ALDH1A1, and YAP/TAZ (Fig. 1a; Supplementary Fig. S2a–c). The data from the chemotreated patient breast tumors and paclitaxel-treated cancer cells indicated that YAP/TAZ might play a significant role in the maintenance of drug-resistant cells, a small population of cells that can evade cell death induced by chemotherapy. These cells exhibit stem cell-like properties,^26^ implying that exposure to chemotherapeutic drugs may induce a shift towards a stem cell phenotype.^27^ Therefore, the TNBC cells were cultured as mammospheres under non-adherent, serum-free conditions to enrich CSCs,^28^ and assess the expression of YAP/TAZ in the spheres. It was observed that YAP/TAZ was significantly upregulated in mammospheres (3D) in comparison to the adherent cells (2D) (Fig. 1a; Supplementary Fig. S2d–f). In breast CSCs, a CD44^+^/CD24^−^ phenotype and high aldehyde dehydrogenase (ALDH) activity are extensively used to determine stemness.^29^ Hence, to ascertain increased expression of YAP/TAZ specifically in CSCs, CD44^+^/CD24^−^ and ALDH^+^ cell populations were isolated from chemo-treated patient breast tumors (Supplementary Fig. S3a), adherent cancer cells and mammospheres (Supplementary Fig. S4a–d). Interestingly, YAP/TAZ and SOX2 were found to be significantly upregulated in the CD44^+^/CD24^−^ and ALDH^+^ population isolated from patient breast tumors (Supplementary Fig. S3b, c), adherent cells (Supplementary Fig. S4e, f) and mammospheres (Supplementary Fig. S4g, h). However, heterogeneity of quiescent and/or proliferative brCSCs may present a limitation for flow cytometry-based sorting of the CSC population, since less prominent CSC phenotypes may require multiple markers and subsequent analysis to be efficiently captured by flow cytometry. Subsequently, since sphere formation across multiple generations is indicative of self-renewal and clonogenic potential,^30^ the expression of YAP, TAZ, and SOX2 were further assessed in multiple generations of mammospheres. Intriguingly, expressions of YAP/TAZ and SOX2 were found to be upregulated in secondary (G2) and tertiary (G3) mammospheres in comparison to primary mammospheres (G1) (Supplementary Fig. S4i, j).

Next, to assess if YAP/TAZ works in conjunction with SOX2 to drive tumorigenesis, computational prediction using docking algorithms were employed. Results revealed physicochemical complementarity between protein interfaces of YAP-SOX2, TAZ-SOX2 as well as YAP and TAZ. Moreover, docking using multi-LZerD predicted that YAP, TAZ and SOX2 could potentially establish a stable trimeric complex (Fig. 1b; Supplementary Fig. S5–7; Supplementary Table S1). To ascertain the clinical relevance of these in silico interactions, co-immunoprecipitation assays were conducted using chemo-treated patient breast tumors. The physical interaction between YAP and TAZ was significantly (*p* < 0.001) higher in the nucleus of patient breast tumors than the adjacent normal tissues. Moreover, interaction between SOX2 and YAP/TAZ was evident in the nucleus of breast tissues, with significant increase in the interaction between YAP/SOX2 (*p* < 0.001) and TAZ/SOX2 (*p* < 0.001) in the breast tumors in comparison to their respective adjacent normal tissues (Fig. 1c; supplementary Fig. S8a, b). Furthermore, YAP/TAZ was also found to co-immunoprecipitate with each other and with SOX2 specifically within the nucleus of the mammospheres (Fig. 1d). Further, to validate possible association of the YAP/TAZ/SOX2 interactome with breast cancer stemness, ALDH^+^ and ALDH^−^ cell populations from patient breast tissues and mice mammary tissues were analyzed. It was observed that ALDH activity was higher in breast tumors when compared to adjacent normal tissues (Fig. 1e, f). Moreover, ALDH^+^ cells isolated from patient breast tumors exhibited elevated expression of YAP, TAZ, and SOX2 (Fig. 1g; Supplementary Fig. S9a), concomitant with enhanced interaction between YAP/TAZ, YAP/SOX2 and TAZ/SOX2 in the nucleus (Fig. 1h; Supplementary Fig. S9b). To reaffirm these findings in vivo, palpable tumors were grown, weighed and measured. A significant increase in both weight and volume of the tumors compared to normal mammary tissues was observed (*p* < 0.01) (Fig. 1i, j). Next, ALDH^+^ and ALDH^−^ cell populations were isolated from normal mammary tissues and mice mammary tumors (Fig. 1k, l). Consequently, overexpression of SOX2, YAP and TAZ was observed in the nuclear fractions of ALDH^+^ cells from mammary tumors (Fig. 1m; Supplementary Fig. S9c). Moreover, enhanced interactions between YAP/TAZ, YAP/SOX2 and TAZ/SOX2 were observed in the ALDH^+^ cells of mice mammary tumors (Fig. 1n; Supplementary Fig. S9d).

Docking analysis had indicated that YAP/TAZ interacts with SOX2 primarily through their TEAD-binding domain (TBD). Hence, to explore the functional importance of this domain in the YAP-SOX2 interaction, *YAP* constructs were engineered with a targeted deletion of the TBD (ΔTBD). Using FLAG-tagged constructs of TBD deleted *YAP* (ΔTBD) co-immunoprecipitation studies were performed. It was observed that deletion of TBD significantly disrupted the ability of YAP to interact with SOX2 (Fig. 1o, p; Supplementary Fig. S10a, b). The impaired binding suggests that the presence of TBD in YAP is necessary for maintaining the integrity of this interaction, which may be crucial for the regulation of stemness properties.

### Silencing *YAP/TAZ* impedes stemness properties and disrupts redox homeostasis in mammospheres

**Fig. 2:**
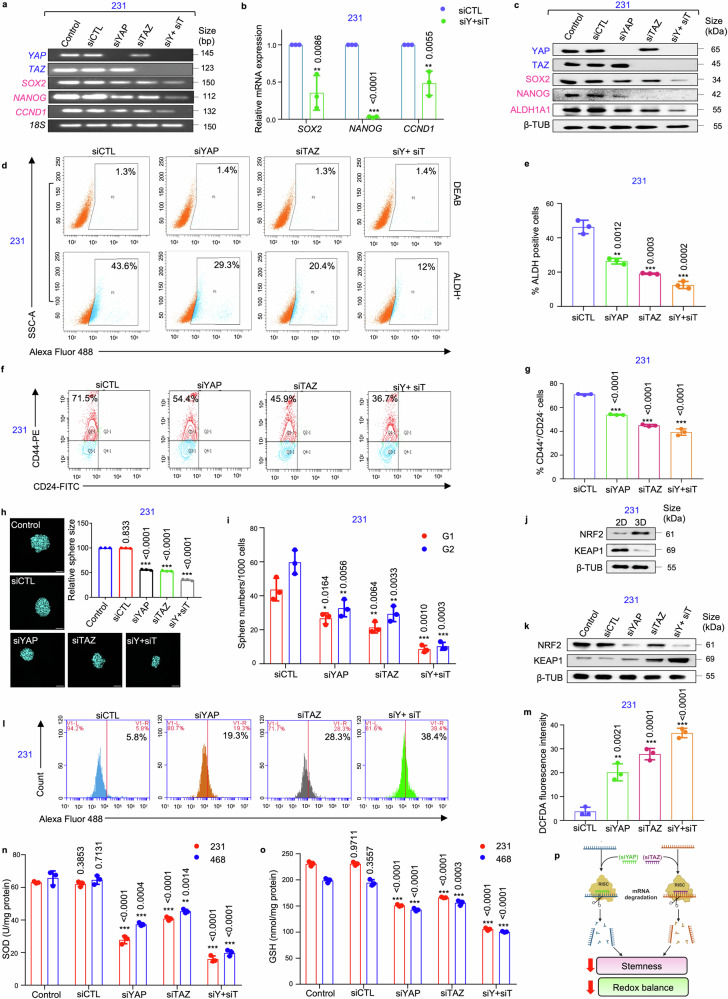
**siRNA-mediated knockdown of** ***YAP/TAZ*** **impairs stemness properties and disrupts redox equilibrium in mammospheres. a** The mRNA expression levels of transcriptional co-activators *YAP*/*TAZ*, transcription factor *SOX2*, and SOX2-target genes *NANOG* and *CCND1* following YAP/TAZ knockdown using semi-quantitative PCR (*n* = 3). **b** Quantitative PCR (qPCR) analysis of *SOX2* and its downstream target genes following depletion of *YAP/TAZ* in mammospheres (*n* = 3). **c** Western blot analyses depicting the expression of stemness markers SOX2, ALDH1A1, and NANOG following *YAP* and *TAZ* knockdown in MDA-MB-231 mammospheres (*n* = 3). **d** Representative plots and **e** quantitative analysis of ALDH activity using ALDEFLUOR assay in MDA-MB-231 mammospheres following 48 h of treatment with siRNA targeting *YAP* and *TAZ*. DEAB, an inhibitor staining control, was used to establish the ALDEFLUOR staining intensity threshold (*n* = 3). **f** Representative plots and **g** quantitative analysis of the percentage of CD44^+^/CD24^−^ populations in mammospheres following *YAP/TAZ* depletion as analyzed by flow-cytometry (*n* = 3). **h** Confocal microscopy images and graphical representation of relative cross-sectional area of MDA-MB-231 spheroids following siRNA treatment (*n* = 3). Nuclei stained with DAPI is represented in cyan. Scale bar: 50 µm. **i** Graphical representation of sphere numbers over generations in MDA-MB-231 mammospheres post siRNA treatment (*n* = 3). **j** Western blot analyses of NRF2 and KEAP1 expression in mammospheres in comparison to adherent cell population (*n* = 3). **k** Western blot analyses of NRF2 and KEAP1 expression in *YAP/TAZ*-depleted MDA-MB-231 mammospheres (*n* = 3). **l**, **m** Assessment of alterations in reactive oxygen species (ROS) levels following genetic depletion of *YAP* and *TAZ* in mammospheres was measured using H2DCFDA assay (*n* = 3). **n** Evaluation of changes in the levels of SOD (superoxide dismutase) and **o** GSH (glutathione) in mammospheres after treatment with control siRNA and *YAP/TAZ*-specific siRNA, either individually or in combination (*n* = 3). **p** Schematic representation of functional outcome of *YAP/TAZ* ablation in mammospheres. This schematic was created using BioRender (https://biorender.com/). All mRNA and protein expressions were normalized against *18S* rRNA and β-tubulin respectively, which served as the internal loading controls. The data are presented as the mean ± standard deviation (SD), with “n” representing the number of biological replicates per experimental group. Significance was assessed using an unpaired Student’s *t*-test, and the associated two-tailed *p*-value is indicated in the bar plots. Compared to the untreated control group: *p < 0.05, **p < 0.01 and ***p < 0.001. Markers associated with stemness are represented in pink, while markers linked to the Hippo signaling pathway are depicted in blue. 231 MDA-MB-231, 468 MDA-MB-468, siCTL control siRNA, siY+siT siYAP+siTAZ, ALDH Aldehyde dehydrogenase, 3D Mammospheres, β-TUB β-tubulin, G1 Generation 1 mammospheres, G2 Generation 2 mammospheres

To validate if the oncogenic potential of SOX2 is governed by coordinated interplay of YAP/TAZ, the expression of *SOX2* and its downstream targets were analyzed following siRNA-mediated knockdown of *YAP/TAZ*. It was observed that loss of *YAP/TAZ* led to a significant reduction in the expressions of SOX2-target genes, *SOX2*, *NANOG* and *CCND1* at the mRNA level (*p* < 0.001) (Fig. 2a, b, Supplementary Fig. S11a–d) and protein levels (*p* < 0.001) (Fig. 2c, Supplementary Fig. S11e–g). Subsequently, *YAP/TAZ* depletion also revealed a significant reduction in the ALDH^+^ cell population in mammospheres (*p* < 0.01) (Fig. 2d, e; Supplementary Fig. S11h, i). Furthermore, *YAP/TAZ* knockdown significantly decreased CD44^+^/CD24^−^ cell population in the mammospheres (*p* < 0.001) (Fig. 2f, g), along with reduction in sphere size (Fig. 2h), number (Fig. 2i) and sphere formation efficiency (Supplementary Fig. S12a).

An upregulation of NRF2 and downregulation of KEAP1 was observed in the mammospheres when compared to adherent cells (Fig. 2j; Supplementary Fig. S12b–d). *YAP/TAZ* depletion significantly reduced the expression of NRF2 along with an increase in the expression of KEAP1 (*p* < 0.001) (Fig. 2k; Supplementary Fig. S12e–g). Concomitantly, an increase in the levels of ROS (Fig. 2l, m; Supplementary Fig. S12h, i), associated with reduced levels of the antioxidants, superoxide dismutase (SOD) and glutathione (GSH), were observed (Fig. 2n, o) in the mammospheres. Collectively, silencing *YAP/TAZ* effectively disrupts both stemness and redox balance in the breast mammospheres (Fig. 2p).

### Mammospheres exhibited fragmented mitochondrial morphology and are metabolically less active with increased susceptibility to OXPHOS inhibition

**Fig. 3:**
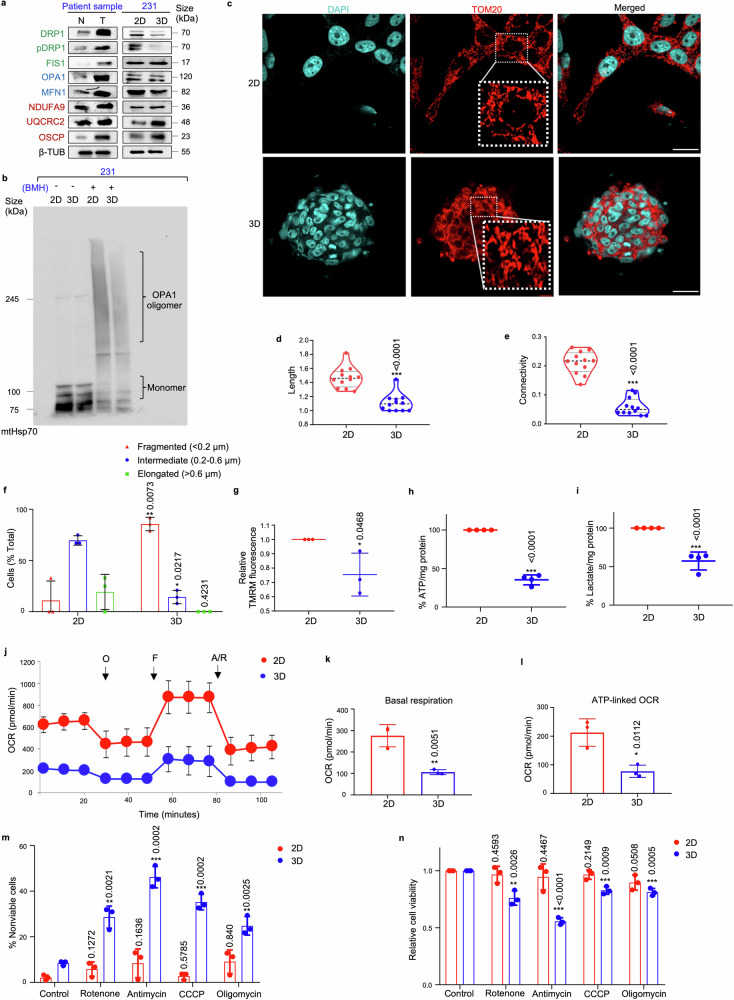
**Mammospheres exhibit fragmented mitochondrial morphology and are metabolically less active. a** Western blot analyses were performed to assess the protein levels of mitochondrial fission markers (DRP1, pDRP1, and FIS1, indicated in green), mitochondrial fusion markers (OPA1 and MFN1, indicated in blue), and ETC complex proteins (NDUFA9, UQCRC2, and OSCP, indicated in red) in patient breast tumors compared to adjacent normal tissues (*n* = 10) and in MDA-MB-231 mammospheres in comparison with the adherent cell population (*n* = 3). **b** Comparison of OPA1 oligomerization pattern between MDA-MB-231 mammospheres and adherent cell population (*n* = 3). **c** Representative confocal microscopic images analysing the mitochondrial morphology of MDA-MB-231 adherent and mammosphere cultures as observed using indirect immunofluorescence for TOM20 (mitochondria, red; nuclei stained with DAPI, cyan). Scale bar: 20 µm (*n* = 3). Quantification of **d** length and **e** connectivity of mitochondria in mammospheres and adherent cell population (*n* = 3; Each point represents length or connectivity of individual cells). **f** Bar plots representing quantification of mitochondrial morphology in mammospheres in comparison to adherent cell population (*n* = 3). **g** The bar graph quantifies TMRM signals in MDA-MB-231 cells and mammospheres (*n* = 3). **h** Quantification of ATP levels and **i** lactate levels in MDA-MB-231 cells and mammospheres (*n* = 4). **j** Mitochondrial respiration rate as reflected by the oxygen consumption rate (OCR) was analysed using Seahorse XF-24 analyzer. The evaluation was performed on MDA-MB-231 adherent cells and mammospheres under basal conditions and following the addition of oligomycin (2 μM), FCCP (1 μM), or rotenone/antimycin (0.5 μM). OCR levels were normalized to the concentration of total protein (*n* = 3). **k** Bar plots representing basal respiration rate and **l** ATP-linked OCR in mammospheres when compared to adherent cell population (*n* = 3). **m** Percentage of non-viable cells were measured using trypan blue exclusion assay in MDA-MB-231 adherent cells and mammospheres after treatment with OXPHOS inhibitors: rotenone, antimycin, CCCP and oligomycin (*n* = 3). **n** Relative cell viability was measured using MTT assay in MDA-MB-231 adherent cells and mammospheres after treatment with OXPHOS inhibitors: rotenone, antimycin, CCCP and oligomycin (*n* = 3). All protein expressions were normalized against β-tubulin, which served as the internal loading control. The data are presented as the mean ± standard deviation (SD), with “*n*” representing the number of biological replicates per experimental group. Significance was assessed using two-way ANOVA followed by Tukey’s post hoc test for Fig. 3f and unpaired Student’s *t*-test for rest of the experiments. The associated *p*-values are indicated in the bar plots. Compared to their respective control group: *p < 0.05, **p < 0.01, and ***p < 0.001. N Normal, T Tumor, 231 MDA-MB-231, 2D Adherent cells, 3D Mammospheres, β-TUB β-tubulin, BMH 1,6-Bis(maleimido)hexane, mt Mitochondria. Adherent cells; 3D Mammospheres, OCR Oxygen consumption rate, O oligomycin, F FCCP, R/A rotenone/antimycin

Since the mitochondria is indispensable for maintenance of stemness,^20^ the expression of mitochondria-shaping proteins and electron transport chain (ETC) complex markers was assessed in chemo-treated patient breast tumors. The results indicated an upregulation of all fission, fusion and ETC complex proteins in chemo-treated patient breast tumors (Fig. 3a; Supplementary Fig. S13a). Assessment of the expression of these markers specifically in mammospheres, in contrast to the adherent cells, revealed a significant reduction in expressions of the mitochondrial fission protein DRP1 and fusion proteins OPA1 and MFN1 (Fig. 3a, Supplementary Fig. S13b–d). Notably, expression of the fission marker FIS1 remained unaltered. Furthermore, OPA1 oligomerization, which is essential for mitochondrial fusion, was also reduced in the mammospheres (Fig. 3b). Contrarily, the mammospheres exhibited a significant increase in the expression of ETC complex proteins (Fig. 3a, Supplementary Fig. S13b–d). Next, the cells were labelled with the mitochondrial outer membrane protein TOM20 and visualized. Mammospheres predominantly exhibited fragmented mitochondrial morphology (Fig. 3c). Quantification using Momito^31^ further revealed significant reduction in length (*p* < 0.001) and connectivity (*p* < 0.001) of mitochondria in the mammospheres compared to adherent cells (Fig. 3d, e). The mitochondrial structures were further manually assessed and categorized as elongated, intermediate, or fragmented (Fig. 3f). In the adherent cells, diverse mitochondrial phenotype was observed, with the majority displaying an intermediate (~70%) phenotype. However, mammospheres predominantly exhibited fragmented (~90%) mitochondrial morphology (Fig. 3f, Supplementary Table S2). The mammospheres exhibited detectable TMRM fluorescence intensity, indicating functional mitochondria notwithstanding repressed dynamic activities (Fig. 3g, Supplementary Fig. S13e).

Subsequently, the correlation between changes in mitochondrial structure dynamics and metabolism was investigated. Quantitative assessment of ATP and lactate levels indicated that the adherent cells produced significantly greater amounts of ATP and lactate, compared to mammospheres (*p* < 0.001) (Fig. 3h, i). Moreover, extracellular flux analysis revealed that mammospheres exhibited significantly lower basal OCR (*p* < 0.01) and ATP-linked OCR (*p* < 0.05) relative to adherent cells (Fig. 3j–l). In order to assess the significance of OXPHOS in mammospheres, we investigated whether inhibiting OXPHOS affected the population of viable cells. Our analysis revealed that treatment with a range of OXPHOS inhibitors-specifically rotenone, antimycin, CCCP (carbonyl cyanide m-chlorophenyl hydrazone), and oligomycin resulted in a pronounced increase in the percentage of non-viable cells, particularly evident within the mammosphere population as opposed to the adherent cell fraction (Fig. 3m, n). The observed increase in non-viable cells following OXPHOS inhibitor treatment underscores the essential role of OXPHOS in maintaining cell viability.

### YAP/TAZ-mediates DRP1 stability in mammospheres to maintain mitochondrial homeostasis

**Fig. 4:**
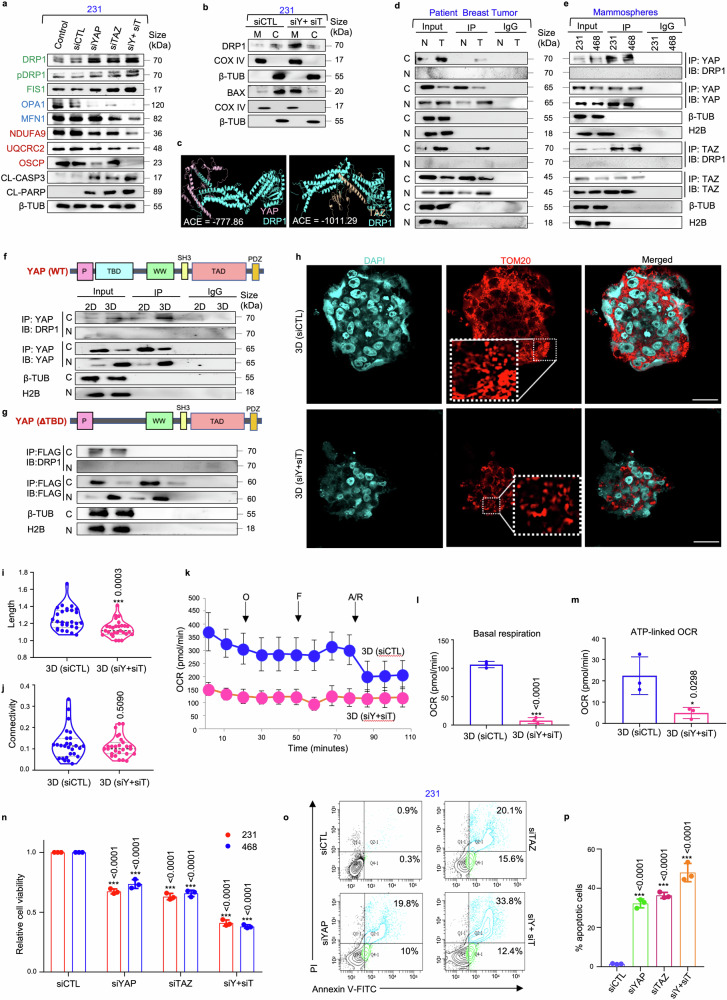
**Silencing YAP/TAZ disrupts mitochondrial homeostasis and induces cell death in mammospheres. a** Western blot analyses were performed to assess the expression of mitochondrial fission markers (DRP1, pDRP1, FIS1), fusion markers (OPA1, MFN1), ETC complex proteins (NDUFA9, UQCRC2, OSCP) and apoptosis markers (CL-caspase 3, CL-PARP) in *YAP/TAZ* depleted mammospheres. Mitochondrial fission, fusion and ETC complex markers are denoted in green, blue, and red respectively (*n* = 3). **b** The cytoplasmic and mitochondrial fractions were analysed for the expression of DRP1 and BAX in mammospheres (*n* = 3). **c** Molecular docking model depicting the DRP1/YAP and DRP1/TAZ complexes. **d** Co-immunoprecipitation experiments utilizing control IgG or anti-YAP/anti-TAZ antibody, for assessing the interaction between YAP/DRP1 and TAZ/DRP1 in patient samples (*n* = 5) and (**e**) in mammospheres (*n* = 3). **f** Co-immunoprecipitation experiments using either control IgG or anti-YAP antibodies, followed by western blot analyses in the adherent and mammosphere culture of MDA-MB-231 (*n* = 3). **g** *YAP* deletion mutant (ΔTBD) was transfected into MDA-MB-231 adherent cells and mammospheres. Co-immunoprecipitation experiments were performed with anti-FLAG antibody and immunoblots were probed with anti-DRP1 or anti-FLAG antibodies (*n* = 3). **h** Representative confocal microscopy images depicting mitochondrial morphology of MDA-MB-231 mammospheres after treatment with control siRNA and dual knockdown with *YAP* and *TAZ*-specific siRNA. Indirect immunofluorescence for TOM20 was utilized to visualize mitochondrial morphology (mitochondria, red; nuclei stained with DAPI, cyan) (*n* = 3). Scale bar: 20 µm. **i** Quantification of length and **j** connectivity of mitochondria in mammospheres (*n* = 3; Each point represents individual cells). **k** Mitochondrial respiration as reflected by the oxygen consumption rate (OCR) in MDA-MB-231 mammospheres after treatment with control and *YAP/TAZ*-specific siRNA under basal conditions and after addition of oligomycin (2 μM), FCCP (1 μM) or rotenone/antimycin (0.5 μM) (*n* = 3). **l** Bar plots representing basal respiration rate and **m** ATP-linked OCR in control mammospheres in comparison to *YAP/TAZ-*depleted mammospheres (*n* = 3). **n** Relative cell viability was measured using MTT assay in mammospheres after treatment with *YAP/TAZ*-specific siRNA (*n* = 3). **o** Representative plots and **p** quantitative analysis of the percentage of apoptotic cells in MDA-MB-231-derived mammospheres following *YAP/TAZ* depletion as analyzed by flow-cytometry using Annexin-V/PI analysis (*n* = 3). Cytosolic, nuclear and mitochondrial protein expressions were normalized against β-tubulin, H2B and COX IV respectively. The data are presented as the mean ± standard deviation (SD), with “n” representing the number of biological replicates per experimental group. Significance was assessed using unpaired Student’s *t*-test, and the associated two-tailed *p*-value is indicated in the bar plots. Compared to their respective untreated control group: *p < 0.05 and ***p < 0.001. 231 MDA-MB-231, 468 MDA-MB-468, siCTL control siRNA, siY+siT siYAP+siTAZ, CL-CASP3 cleaved-caspase 3, CL-PARP cleaved-PARP, β-TUB β-tubulin, ACE Atomic contact energy, 3D Mammospheres, OCR Oxygen consumption rate, O oligomycin, F FCCP, R/A rotenone/antimycin, C Cytoplasm, Nuc Nucleus, IP Immunoprecipitate, IB Immunoblot, M Mitochondria

Based on the established link between YAP/TAZ and redox equilibrium, we next explored whether YAP/TAZ support cell survival by maintaining mitochondrial homeostasis. Consequently, siRNA-mediated suppression of YAP/TAZ led to a reduction in the expressions of OPA1, MFN1, NDUFA9, UQCRC2, and OSCP (Fig. 4a). However, a significant increase in the expressions of DRP1, pDRP1 and FIS1 were observed in the mammospheres, along with increased expression of the apoptosis markers, cleaved caspase-3 and cleaved PARP (Fig. 4a; Supplementary Fig. S14a–c).

DRP1 is generally recruited to outer mitochondrial membrane during mitochondrial fission.^32^ In mammospheres, DRP1 was predominantly localized within the cytoplasm. However, following *YAP/TAZ* silencing, there was an observed increase in DRP1 expression in the mitochondria (Fig. 4b; Supplementary Fig. S14d–f). Cell death, if any, was further evaluated upon *YAP/TAZ* knockdown. Similar to DRP1, siRNA-mediated knockdown of *YAP/TAZ* enhanced the expression of BAX in the mitochondria (Fig. 4b; Supplementary Fig. S14d–f), indicating induction of apoptosis.

To unravel the mechanisms behind YAP/TAZ-mediated regulation of DRP1, in silico docking analyses were performed, and both YAP and TAZ showed spontaneous binding affinities for DRP1 (Fig. 4c; Supplementary Figs. S15, S16a, b). Co-immunoprecipitation assays further revealed that YAP/TAZ interacted with DRP1 in the cytoplasm of chemo-treated patient breast tumors (Fig. 4d; Supplementary Fig. S16c, d) and mammospheres (Fig. 4e). Moreover, the domain of YAP responsible for binding to DRP1 was characterized. Docking analyses revealed that the interaction primarily occurs through the TBD, as indicated by the interface residues (Supplementary Fig. S15, S16a, b). To confirm the interaction domain, deletion constructs were made to assess the interaction of YAP with DRP1. Targeted deletion of the TBD (ΔTBD) significantly impaired the ability of YAP to interact with DRP1 in the cytoplasm (Fig. 4f, g; Supplementary Fig. S17a, b).

Furthermore, *YAP/TAZ* depletion resulted in persistent fragmented mitochondrial morphology (Fig. 4h), accompanied by a significant reduction in mitochondrial length (*p* < 0.001) (Fig. 4i), without any notable alterations in mitochondrial connectivity in MDA-MB-231 mammospheres (Fig. 4j). Severely impaired mitochondrial respiration with significant reduction in basal (*p* < 0.001) and ATP-linked OCR (*p* < 0.05) was also observed (Fig. 4k–m). Furthermore, the change in mitochondrial dynamics and metabolism was also associated with a significant decrease in the viable cell population (*p* < 0.001) (Fig. 4n; Supplementary Fig. S17c). This finding was further validated by annexin V/PI apoptosis assay, where an increase in apoptotic cell percentage was observed (*p* < 0.001) (Fig. 4o, p).

### Inhibition of *YAP/TAZ* impairs stemness and destabilizes mitochondrial homeostasis to promote cell death in TNBC patient-derived spheroids

**Fig. 5:**
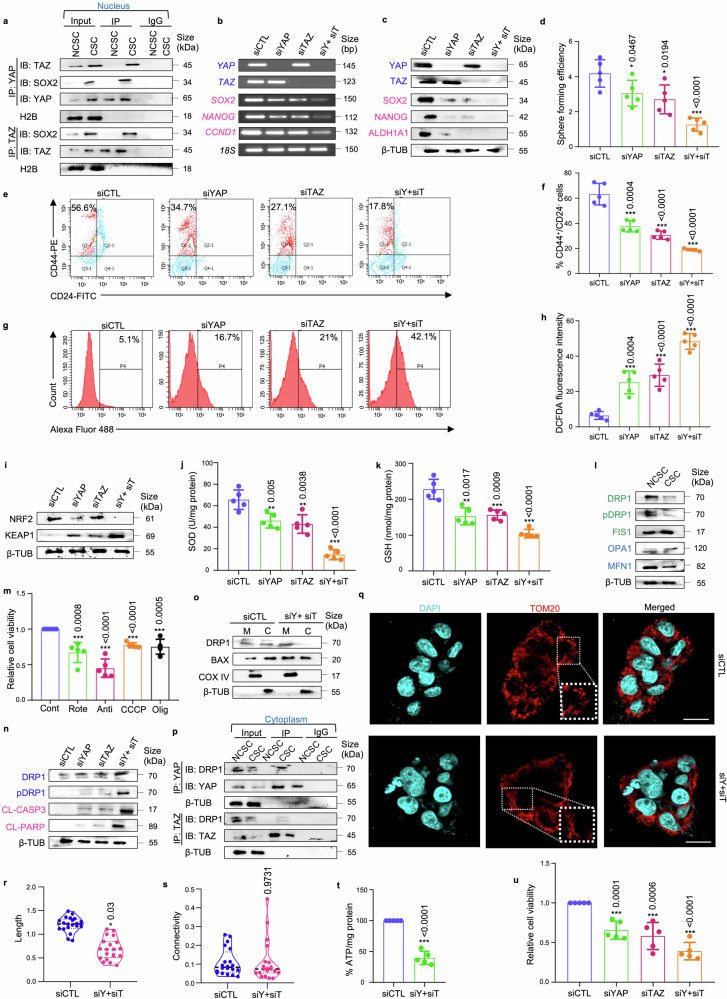
***YAP/TAZ*** **depletion disrupts cellular homeostasis in TNBC patient-derived spheroids. a** Co-immunoprecipitation (Co-IP) analyses using either control IgG or antibodies against YAP and TAZ. Western blot analyses were performed to analyse differential interaction pattern in the nucleus of CD44^+^/CD24^−^ cell population (representing CSCs) in comparison with rest of the cell population (representing non-CSCs) isolated from patient breast tumors (*n* = 5). **b** The mRNA expression levels of transcriptional co-activators *YAP*/*TAZ*, transcription factor *SOX2*, and *SOX2*-target genes *NANOG* and *CCND1* following *YAP/TAZ* knockdown in patient-derived spheroids using semi-quantitative PCR (*n* = 5). **c** Western blot analyses depicting the expression of stemness markers SOX2, ALDH1A1, and NANOG following *YAP* and *TAZ* knockdown in patient-derived spheroids (*n* = 5). The hippo pathway associated markers are indicated in blue and stemness markers are indicated in pink. **d** Graphical representation of sphere forming efficiency post siRNA treatment in patient-derived spheroids (*n* = 5). **e** Representative plots and **f** quantitative analysis of the percentage of CD44^+^/CD24^−^ populations in patient-derived spheroids following *YAP/TAZ* depletion as analyzed by flow-cytometry (*n* = 5). **g**, **h** Assessment of alterations in reactive oxygen species (ROS) levels following genetic depletion of *YAP* and *TAZ* in patient-derived spheroids was measured using H2DCFDA assay (*n* = 5). **i** Western blot analyses of NRF2 and KEAP1 expression in patient-derived spheroids post *YAP/TAZ* knockdown (*n* = 5). **j** Evaluation of changes in the levels of SOD (superoxide dismutase) and **k** GSH (glutathione) in patient-derived spheroids after treatment with control siRNA and *YAP/TAZ*-specific siRNA, either individually or in combination (*n* = 5). **l** Western blot analyses were performed to assess the expression of mitochondrial fission markers (DRP1, pDRP1, FIS1) and fusion markers (OPA1, MFN1) in CSCs compared to NCSCs (*n* = 5). Mitochondrial fission and fusion markers are denoted in green and blue respectively. **m** Relative cell viability was measured using MTT assay in patient-derived spheroids after treatment with OXPHOS inhibitors: rotenone, antimycin, CCCP and oligomycin (*n* = 5). **n** Western blot analyses were performed to assess the expression of mitochondrial fission markers (DRP1, pDRP1) and apoptosis markers (CL-caspase 3, CL-PARP) in *YAP/TAZ* depleted spheroids. Mitochondrial fission and apoptosis markers are denoted in blue and pink respectively (*n* = 5). **o** The cytoplasmic and mitochondrial fractions were analysed for the expression of DRP1 and BAX in spheroids (*n* = 5). **p** Co-immunoprecipitation experiments utilizing control IgG or anti-YAP/anti-TAZ antibody, for assessing the interaction between YAP/DRP1 and TAZ/DRP1 in the cytoplasm of CD44^+^/CD24^−^ cell population (representing CSCs) in comparison with rest of the cell population (representing non-CSCs) isolated from patient breast tumors (*n* = 5). **q** Representative confocal microscopy images depicting mitochondrial morphology of patient-derived spheroids after treatment with control siRNA and dual knockdown with *YAP* and *TAZ*-specific siRNA. Indirect immunofluorescence for TOM20 was utilized to visualize mitochondrial morphology (mitochondria, red; nuclei stained with DAPI, cyan) (*n* = 5). Scale bar: 20 µm. **r** Quantification of length and **s** connectivity of mitochondria in patient-derived spheroids (*n* = 5; Each point represents individual cells). **t** Quantification of ATP levels in spheroids post siRNA treatment (*n* = 5). **u** Relative cell viability was measured using MTT assay in patient-derived spheroids after treatment with *YAP/TAZ*-specific siRNA (*n* = 5). mRNA expressions were normalized against 18S rRNA. Protein expressions in nuclear, cytoplasmic and mitochondrial fraction were normalized against H2B, β-tubulin and COXIV respectively. The data are presented as mean ± standard deviation (SD), with “*n*” representing the number of biological replicates per experimental group. Significance was assessed using an unpaired Student’s *t*-test, and the associated two-tailed *p*-value is indicated in the bar plots. Compared to the untreated control group: *p < 0.05, **p < 0.01 and ***p < 0.001. IP immunoprecipitate, IB immunoblot, NCSC non-cancer stem cells, CSC cancer stem cells, siCTL control siRNA, siY+siT siYAP+siTAZ, β-TUB β-tubulin, Cont control, Rote Rotenone, Anti Antimycin, Olig Oligomycin, M mitochondria, C Cytoplasm

To explore the clinical relevance of our findings, patient-derived spheroids from chemotreated TNBC tumors were established. Initially, the association of YAP and TAZ with SOX2 was re-validated by isolating the CD44^+^/CD24^−^ cell population from patient tumor samples. Notably, the interaction between YAP/SOX2 and TAZ/SOX2 was exclusively observed in the nucleus of the CD44^+^/CD24^−^ cells, suggesting a unique characteristic of brCSCs (Fig. 5a; Supplementary Fig. S18a, b). Subsequently, to assess the role of YAP/TAZ in regulating breast cancer stemness, siRNA-mediated knockdown of YAP/TAZ was performed in the patient-derived spheroids. Consistent with observations in mammospheres derived from TNBC cell lines, silencing *YAP/TAZ* led to a substantial reduction in *SOX2* expression and its downstream targets *NANOG* and *CCND1* at both mRNA and protein levels (Fig. 5b, c; Supplementary Fig. S19a, b). This reduction in stemness markers correlated with a marked decline in sphere forming efficiency (Fig. 5d) and the CD44^+^/CD24^−^ cell population (Fig. 5e, f). *YAP/TAZ* depletion also resulted in a notable increase in reactive oxygen species (ROS) levels (Fig. 5g, h) associated with a decrease in NRF2 expression and an increase in KEAP1 expression (Fig. 5i; Supplementary Fig. S19c). Furthermore, a marked reduction in superoxide dismutase (SOD) and glutathione (GSH) levels was observed (Fig. 5j, k). Next, in order to revisit the relationship between YAP/TAZ and mitochondrial dynamics, the expression of mitochondrial fission and fusion markers were analyzed in the CD44^+^/CD24^−^ cell population compared to the non-CSC population. Similar to the mammospheres, CSCs exhibited decreased levels of fission/fusion markers (Fig. 5l; Supplementary Fig. S20a) and demonstrated susceptibility to OXPHOS inhibition (Fig. 5m). *YAP/TAZ* depletion further resulted in an increase in the expression of DRP1, pDRP1 and apoptosis markers in mammospheres (Fig. 5n; Supplementary Fig. S20b). This upregulation of DRP1 correlates with elevated mitochondrial levels of BAX (Fig. 5o; Supplementary Fig. S20c). YAP/TAZ was also found to interact with DRP1 in the cytoplasm, with this interaction being exclusive to the CD44^+^/CD24^−^ cell population isolated from patient breast tumors (Fig. 5p; Supplementary Fig. S21a, b). Additionally, *YAP/TAZ* depletion led to a significant reduction in mitochondrial length (*p* < 0.01), while no statistically significant change was observed in the mitochondrial connectivity (Fig. 5q–s). Collectively, these alterations resulted in a significant decrease in ATP levels (*p* < 0.001) (Fig. 5t) and relative cell viability of patient-derived spheroids (*p* < 0.001) (Fig. 5u).

### Verteporfin, a YAP/TAZ inhibitor, reduces stemness properties and tumor initiating potential of mammospheres

**Fig. 6:**
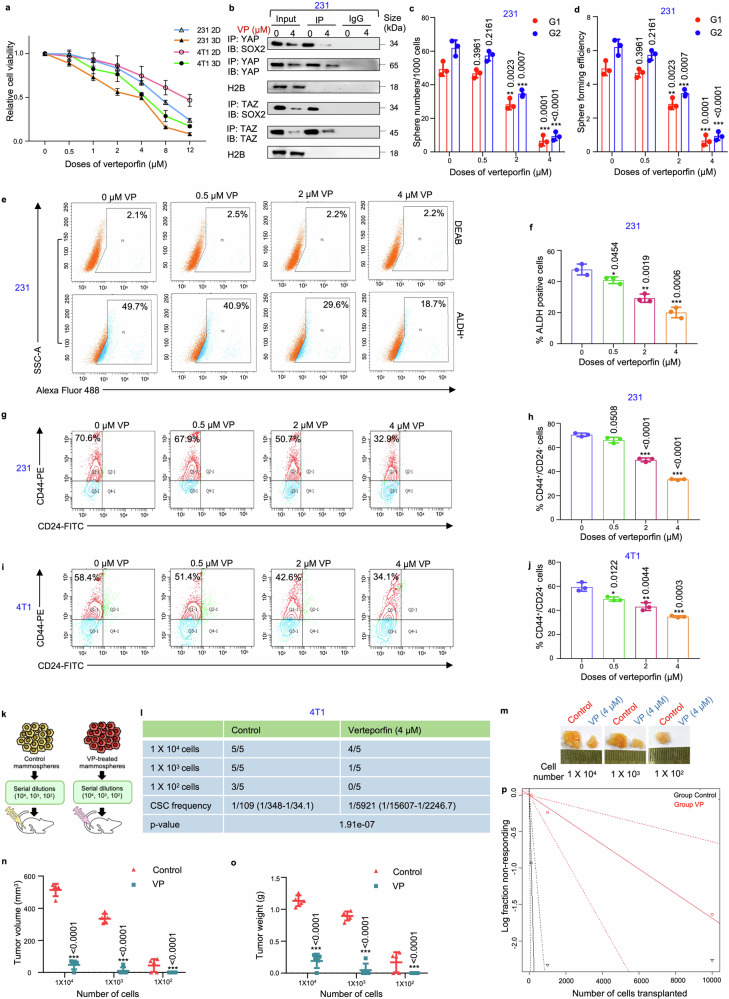
**Verteporfin reduces stemness in triple negative breast cancer-derived mammospheres. a** Adherent and mammosphere cultures of MDA-MB-231 and 4T1 cells were treated with various concentrations of VP (0, 0.5, 1, 2, 4, 8, 12 µM) for a duration of 48 hours. Following the incubation, cell viability was assessed using the MTT assay, and the results are presented as relative cell viability compared to the DMSO control (*n* = 3). **b** Co-immunoprecipitation analysis using control IgG and anti-YAP/anti-TAZ antibody in the nucleus of 4 µM VP-treated MDA-MB-231 mammospheres, followed by western blot analyses (*n* = 3). **c** Graphical representation of sphere numbers and **d** sphere forming efficiency over generations in MDA-MB-231 mammospheres post VP treatment (*n* = 3). **e** Representative plots and **f** quantitative analysis of ALDH activity using ALDEFLUOR assay in MDA-MB-231 mammospheres following 48 h of treatment with different doses of VP. DEAB, an inhibitor staining control, was used to establish the ALDEFLUOR staining intensity threshold (*n* = 3). **g** Representative plots and **h** quantitative analysis of the percentage of CD44^+^/CD24^−^ populations in MDA-MB-231 mammospheres following VP treatment as analyzed by flow-cytometry (*n* = 3). **i** Representative plots and **j** quantitative analysis of the percentage of CD44^+^/CD24^−^ populations in 4T1 mammospheres following VP treatment as analyzed by flow-cytometry (*n* = 3). **k** A schematic illustration depicting the experimental framework for the limiting dilution transplantation assay. **l** In vivo limiting dilution transplantation assay using 4T1 mammospheres treated with either DMSO or VP (4 µM) for 48 h. Different cell dilutions (10^4^, 10^3^, or 10^2^ cells per injection site, *n* = 5) were utilized. The frequency of breast cancer stem cells (brCSCs) was determined using ELDA (extreme limiting dilution analysis, http://bioinf.wehi.edu.au/software/elda/). **m** Representative images of tumor formation in BALB/c mice. **n** Harvested tumors were measured for volume and **o** weight at the end of the experiment. **p** A log-fraction plot derived from the limiting dilution model. The slope of the line represents the log active cell fraction, with dotted lines indicating the 95% confidence interval. The control group is depicted in black, while the VP-treated group is shown in red. Nuclear protein expressions were normalized against H2B, which served as the internal loading control. The data are presented as the mean ± standard deviation (SD), with “*n*” representing the number of biological replicates per experimental group. Significance was assessed using unpaired Student’s *t*-test, and the associated two-tailed *p*-value is indicated in the bar plots. Compared to their respective untreated controls: *p < 0.05, **p < 0.01, ***p < 0.001. 231 MDA-MB-231, 2D Adherent cells, 3D Mammospheres, ALDH Aldehyde dehydrogenase, VP Verteporfin, IP Immunoprecipitate, IB Immunoblot, G1 Generation 1, G2 Generation 2

In accordance with gene silencing of YAP/TAZ, VP, a small molecule inhibitor of YAP/TAZ, was used to pharmacologically deplete YAP/TAZ. Molecular docking analysis validated that VP interacted with both YAP and TAZ (Supplementary Fig. S22, 23). Subsequently, MDA-MB-231 cells and mammospheres were treated with different doses of VP (0, 0.5, 1, 2, 4, 8, 12 µM) and cell viability was assessed. It was observed that mammospheres had a lower IC_50_ value (4 µM) in comparison to the adherent breast cancer cells (7 µM). Comparable results were observed with the 4T1 and MDA-MB-468 cell line (Fig. 6a; Supplementary Table S3; Fig. S24a). Co-immunoprecipitation assays revealed that 4 µM VP not only downregulated the expressions of YAP, TAZ and SOX2, it consequently disrupted the association between YAP/SOX2 and TAZ/SOX2 (Fig. 6b; Supplementary Fig. S24b, c). Reduced expression and interaction of YAP/TAZ with SOX2 following VP treatment was associated with a significant decrease in sphere number and sphere forming efficiency (*p* < 0.01) in MDA-MB-231 mammospheres over two generations (Fig. 6c, d). Furthermore, VP treatment downregulated both the ALDH^+^ population (Fig. 6e, f; Supplementary Fig. S24d, e) and CD44^+^/CD24^−^ cell population (Fig. 6g, h) in the mammospheres. VP treatment was also found to reduce the CD44^+^/CD24^−^ cell population in 4T1-mammospheres (Fig. 6i, j). As an orthogonal method, limiting dilution transplantation assay, was performed. 4T1 mammospheres implanted at a cell density of 1 × 10^4^ and 1 × 10^3^ formed tumors with 100% efficiency in the control groups, whereas in VP-treated groups, the tumor formation efficiency reduced to 80% and 20%, respectively, along with significant decrease in tumor size, volume and CSC frequency, as calculated by ELDA (extreme limiting dilution analysis). Remarkably, implantation with 1 × 10^2^ cells from 4T1 mammospheres formed tumors with 60% efficiency in the control group, whereas no tumor formation was observed in the VP-treated group (Fig. 6k–p).

### Verteporfin disrupts mitochondrial and redox homeostasis in mammospheres

**Fig. 7:**
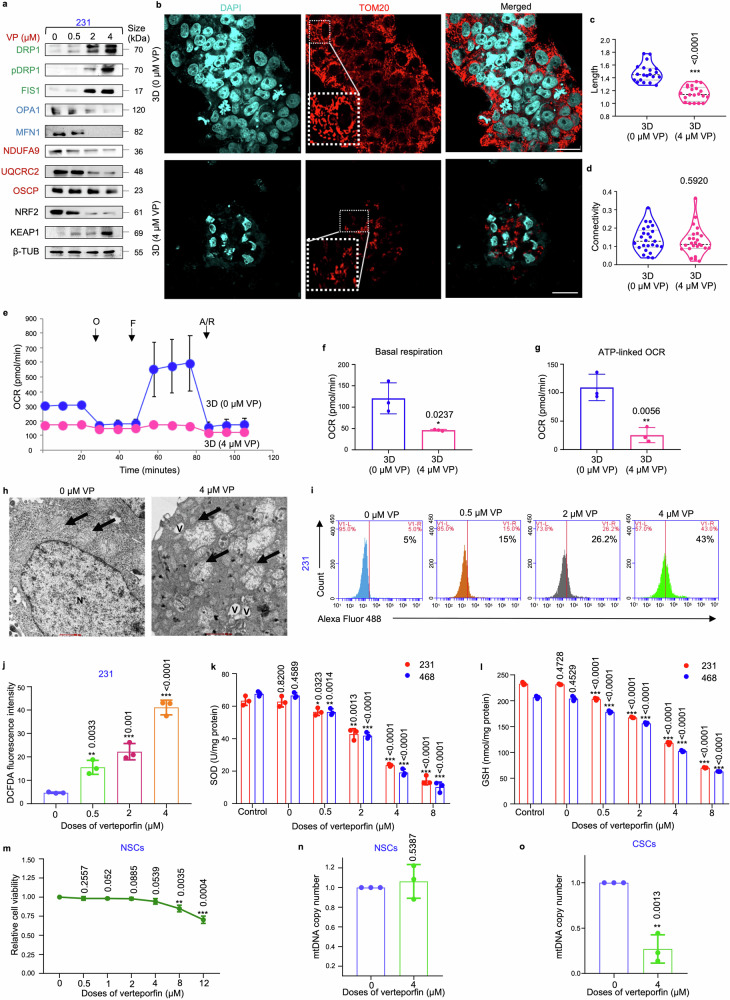
**Verteporfin induces mitochondrial dysfunction in mammospheres. a** Mammospheres were subjected to treatment with varying concentrations of VP (0.5, 2, 4 µM) for 48 h. Subsequently, western blot analyses were conducted to assess the expression levels of mitochondrial fission markers (DRP1, pDRP1, FIS1, indicated in green), fusion markers (OPA1, MFN1, indicated in blue), ETC complex proteins (NDUFA9, UQCRC2, OSCP, indicated in red), NRF2 and KEAP1 (*n* = 3). **b** Representative confocal microscopic images illustrating the mitochondrial morphology of MDA-MB-231 mammospheres treated with DMSO control and 4 µM VP for 48 h. Indirect immunofluorescence for TOM20 was used to visualize the mitochondrial morphology (mitochondria, red; nuclei stained with DAPI, cyan). Scale bar: 20 µm (*n* = 3). **c** Quantification of length and **d** connectivity of mitochondria in mammospheres and adherent cell population (*n* = 3; Each point represents individual cells). **e** Mitochondrial respiration as reflected by the oxygen consumption rate (OCR) was analysed in MDA-MB-231 mammospheres after treatment with 4 µM VP in comparison to control mammospheres under basal conditions and after addition of oligomycin (2 μM), FCCP (1 μM) or rotenone/antimycin (0.5 μM) (*n* = 3). **f** Bar plots representing basal respiration rate and **g** ATP-linked OCR in control mammospheres in comparison to 4 µM VP-treated mammospheres (*n* = 3). **h** Representative transmission electron microscopic images depicting control and 4 µM VP-treated MDA-MB-231 mammospheres. Mitochondria are identified by black arrows. *N* represents nucleus, and V represents vacuoles. Scale bar: 500 nm (*n* = 3). **i**, **j** Change in ROS levels following VP treatment of MDA-MB-231 mammospheres were quantified using H2DCFDA assay (*n* = 3). **k** Estimation of the change in the levels of the antioxidants SOD (superoxide dismutase) and **l** GSH (glutathione) in mammospheres after treatment with VP (*n* = 3). **m** Relative cell viability of ALDH^+^ cells isolated from human normal breast tissue (representing normal stem cells) upon VP treatment (*n* = 3). **n** Assessment of mitochondrial DNA copy number in VP treated ALDH^+^ cells isolated from human normal breast tissue (representing normal stem cells; NSC) and **o** human breast tumor tissue (representing cancer stem cells; CSC). All protein expressions were normalized against β-tubulin, which served as the internal loading control. The data are presented as the mean ± standard deviation (SD), with “*n*” representing the number of biological replicates per experimental group. Significance was assessed using an unpaired Student’s *t*-test, and the associated two-tailed *p*-value is indicated in the bar plots. Compared to the untreated control group: *p < 0.05, **p < 0.01 and ***p < 0.001. 231 MDA-MB-231, 468 MDA-MB-468, VP Verteporfin, β-TUB β-tubulin, 3D Mammospheres, OCR Oxygen consumption rate, O oligomycin, F FCCP, R/A rotenone/antimycin, mtDNA mitochondrial DNA

Next, the effect of VP on mitochondrial physiology was investigated. Similar to siRNA-mediated silencing of *YAP/TAZ*, VP triggered a dose-dependent increase in the expressions of DRP1, FIS1 and KEAP1 (Fig. 7a). In addition, reduced levels of mitochondrial fusion proteins, ETC complex proteins and NRF2 were observed (Fig. 7a; supplementary Fig. S25a–c). Treatment with the IC_50_ dose of VP (4 µM) further resulted in persistent fragmented mitochondrial morphology (Fig. 7b), with significant decrease in mitochondrial length (Fig. 7c). However, no significant change in mitochondrial connectivity was observed (Fig. 7d). Impaired mitochondrial respiration, resulting in a significant reduction in basal and ATP-linked OCR was also observed in VP-treated mammospheres (Fig. 7e–g). TEM images indicated that control mammospheres exhibited fragmented mitochondrial morphology, with definite oval shape and organized cristae. In contrast, VP treatment resulted in fragmented mitochondria characterized by irregular shape, mitochondrial matrix swelling, disintegrated cristae, increased granularity, and heightened cytoplasmic vacuolation, indicative of mitochondria-mediated cell death (Fig. 7h). VP treatment also led to increased intracellular ROS levels (Fig. 7i, j; Supplementary Fig. S25d, e) and decreased levels of SOD and GSH (Fig. 7k, l). Interestingly, the results revealed that VP exerted no significant toxicity in mammospheres cultured from normal breast tissues (Fig. 7m), or initiated change in mitochondrial biogenesis upon treatment with 4 µM VP (Fig. 7n). Contrarily, VP significantly reduced mitochondrial biogenesis in the mammospheres cultured from patient breast tumors (Fig. 7o).

### Verteporfin, in combination with paclitaxel, induces apoptosis-mediated cell death in CSCs from cell lines and patient-derived organoids

**Fig. 8:**
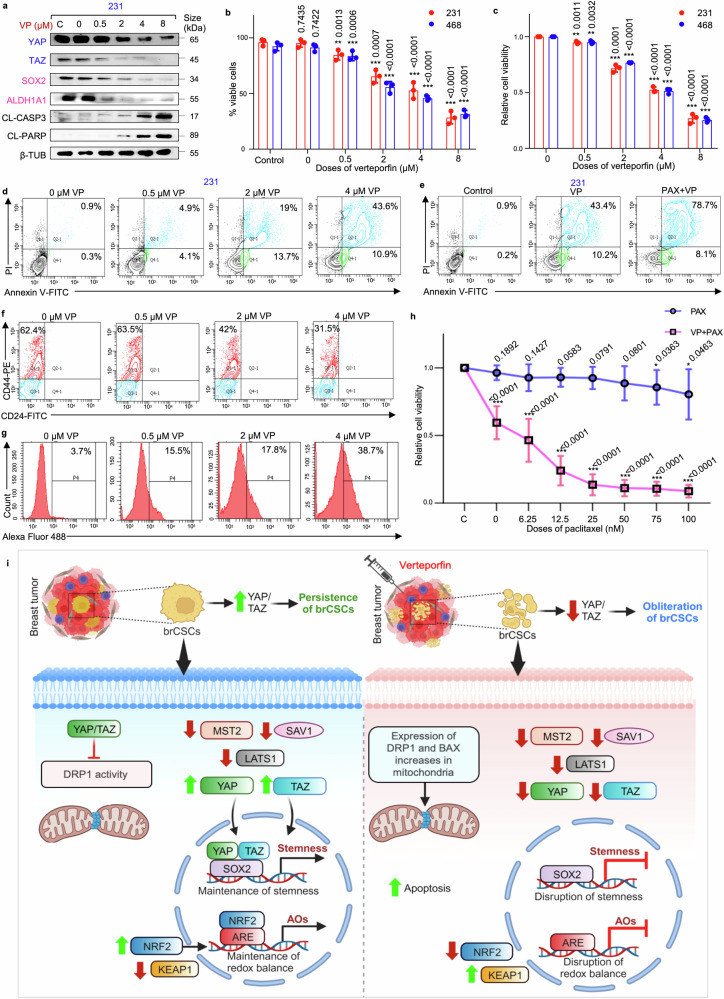
**Verteporfin in combination with paclitaxel induces apoptosis-mediated cell death in CSCs from cell lines and patient-derived organoids. a** Western blot analyses depict the expression levels of YAP/TAZ, stemness markers (SOX2 and ALDH1A1) and apoptosis markers (cleaved-caspase 3 and cleaved-PARP) in VP-treated MDA-MB-231 mammospheres (*n* = 3). Markers associated with Hippo signaling pathway are depicted in blue, while markers linked to stemness are represented in pink. **b** Assessment of the percentage viable cells and **c** relative cell viability using trypan blue exclusion assay and MTT assay, respectively, in mammospheres after treatment with VP (*n* = 3). **d** Representative plots of the percentage apoptotic cells in mammospheres following VP treatment as analyzed by flow-cytometry using Annexin V/PI analysis (*n* = 3). **e** Representative plots of apoptotic cell percentage in mammospheres following treatment with VP alone and in combination with paclitaxel, assessed by flow cytometry using Annexin V/PI analysis (*n* = 3). **f** Representative plots of the percentage of CD44^+^/CD24^−^ populations in patient-derived spheroids following VP treatment as analyzed by flow-cytometry (*n* = 5). **g** Change in ROS levels following VP treatment of patient-derived spheroids were quantified using H2DCFDA assay (*n* = 5). **h** Cell viability of untreated and 4 µM VP-treated TNBC patient-derived organoids following paclitaxel exposure. Organoids were pre-treated with VP for 48 h, then exposed to graded doses of paclitaxel. Viability was then quantified after 24 h (*n* = 5). **i** Hypothetical model delineating possible mechanistic pathway of YAP/TAZ-mediated regulation of brCSCs on the left, where YAP/TAZ might promote persistence of brCSCs. On the right, an alternative mechanism illustrates the putative cascade that might promote the eradication of the brCSCs upon VP treatment, subsequent to the depletion of YAP/TAZ. This schematic was created using BioRender (https://biorender.com). All protein expressions were normalized against β-tubulin, which served as the internal loading control. The data are presented as the mean ± standard deviation (SD), with “*n*” representing the number of biological replicates per experimental group. Significance was assessed using an unpaired Student’s *t*-test, and the associated two-tailed *p*-value is indicated in the bar plots. Compared to the untreated control group: *p < 0.05, **p < 0.01 and ***p < 0.001. 231 MDA-MB-231, 468 MDA-MB-468, VP verteporfin, PAX paclitaxel, CL-CASP3 cleaved-caspase 3, CL-PARP cleaved-PARP, β-TUB β-tubulin, brCSC breast cancer stem cells, ARE anti-oxidant response element, AOs anti-oxidants

It was observed that VP treatment led to a dose-dependent increase in the expression of apoptotic markers (cleaved-caspase3 and cleaved-PARP) accompanied by decrease in the expression of YAP/TAZ and stemness markers (SOX2, ALDH1A1) (Fig. 8a; Supplementary Fig. S26a–c). Furthermore, it was observed that VP led to a significant dose-dependent decrease in the viable cell population (Fig. 8b, c), concomitant with a significant increase in apoptotic cell percentage (Fig. 8d, Supplementary Fig. S27a). A previous study^25^ in our laboratory had depicted that the IC_50_ of Pax in spheres developed from MDA-MB-231 cells was 7 nM, comparable to the cancer cells. Paclitaxel alone at 7 nM did not affect the viability of the mammospheres. However, when combined with 4 µM VP, a significant reduction in mammosphere viability (~80%; *p* < 0.01) and a concurrent increase in the percentage of apoptotic cells was observed, compared to paclitaxel monotherapy (Fig. 8e, Supplementary Fig. S27b, c).

Subsequently, a patient-derived spheroid model was further utilized to evaluate the dose-specific response to VP. At comparable doses, VP significantly reduced the CD44^+^/CD24^−^ cell population (*p* < 0.001) (Fig. 8f, Supplementary Fig. S27d) while markedly increasing cellular ROS levels (*p* < 0.001) (Fig. 8g, Supplementary Fig. S27e). Consistent with findings from mammospheres, these alterations were associated with decreased sphere-forming efficiency and relative cell viability (*p* < 0.001) (Supplementary Fig. S27f, g). Next, patient-derived organoids (PDOs) were used to investigate the effects of a combination treatment involving IC_50_ dose of VP (4 µM) and the conventional chemotherapeutic agent paclitaxel. The PDOs predominantly formed large, spherical structures. At clinically relevant concentrations, paclitaxel alone exhibited minimal cytotoxicity, with only marginal loss in cell viability observed at higher doses (75 nM and 100 nM, *p* < 0.05) (Fig. 8h). However, treatment with 4 µM VP alone resulted in a significant decrease in cell viability. When the PDOs were pre-treated with VP, and exposed to various doses of paclitaxel, a significant decline in cell viability was observed even at lower doses of paclitaxel (*p* < 0.001), suggesting that VP sensitizes the PDOs to paclitaxel chemotherapy (Fig. 8h).

## Discussion

Conventional therapeutic strategies currently administered to patients effectively target the majority of actively proliferating bulk tumor cells. Yet the persistence of drug-resistant CSCs remains a challenge, since they may contribute significantly to disease relapse and therapy resistance,^26^ leading to worse patient prognosis. Therefore, eliminating these CSCs is crucial to mitigate the risk of tumor recurrence. While conventional chemotherapeutic agents often fall short in this regard, drugs specifically targeting the CSC population hold promise for achieving a more comprehensive cancer treatment. Thus, comprehending the mechanisms underlying the therapy resistant CSCs is essential for devising effective therapeutic strategies.

A plethora of evidence suggests that the transcription factor SOX2 plays a pivotal role as an orchestrator of stemness. Given the heightened expression of SOX2 in brCSCs,^25,33^ it was judicious to target brCSCs by inhibiting SOX2. Paradoxically, direct targeting of transcription factors presents a formidable challenge, as they lack specific druggable binding pockets.^4,5^ Moreover, it has been reported that as an independent transcription factor, the DNA binding capacity of SOX2 is mostly limited.^33^ Acquisition of stemness is driven conjointly at the transcriptional level by diverse co-activators that stabilize the foundation of SOX2 transcription initiation complexes.^34,35^ Therefore, we hypothesized that an alternative strategy to indirectly target SOX2 could prove to be a prudent approach in drug designing. One such approach, which involved targeting transcriptional co-activators, has been extremely promising, as seen in this study. A gene profiler array performed earlier in our laboratory^25^ identified transcriptional co-activators YAP/TAZ, to be upregulated in brCSCs isolated from chemotreated patient breast tumors. When the Hippo pathway is activated, MST1/2, along with its scaffold protein SAV1, phosphorylates LATS1/2, which then phosphorylates and induces proteasomal degradation of YAP/TAZ.^24^ However, in brCSCs, we observed a distinct downregulation of MST2 and LATS1, coupled with a significant increase in the levels of YAP/TAZ, suggesting that the Hippo pathway is inactive in these cells. Interestingly, while the levels of YAP/TAZ were elevated, no significant change in SAV1 expression was observed. This lack of SAV1 modulation can be explained by the fact that in the context of decreased MST2 and LATS1, the function of SAV1 as an adaptor for MST2 in promoting LATS1 phosphorylation becomes redundant.

Subsequently, our findings revealed that SOX2 spontaneously engaged in physical interactions with transcriptional co-activators YAP and TAZ, the key effectors of Hippo signaling pathway in brCSCs. It is widely acknowledged that SOX2 not only potentiates its own transcription but also controls that of other transcription factors which maintain stemness, like *NANOG*.^36,37^ *YAP/TAZ* depletion in mammospheres resulted in significant decrease in the expression of SOX2 downstream targets, implying that YAP/TAZ may act as transcriptional co-activators to accentuate the activity of SOX2. This finding underscores the therapeutic potential of selectively targeting the brCSCs by indirectly perturbing SOX2 signaling as a consequence of ablation of YAP/TAZ expression.

Since redox sensors play a pivotal role in the survival and persistence of CSCs,^38^ downregulation of NRF2 and disruption of redox balance upon *YAP/TAZ* knockdown, indicated that YAP/TAZ may exert a dual influence by regulating the expression of SOX2 and its downstream target genes on one hand, while simultaneously modulating the dynamic regulatory mechanisms that control cellular responses to stress. This bifurcated regulatory function turns out to be crucial for the maintenance of brCSCs, and was successfully targeted by repression of the YAP/TAZ/SOX2 complex.

There is growing recognition that the function of the mitochondria is indispensable for the maintenance of stemness,^20^ suggesting a possible regulatory link between redox equilibrium and mitochondria in the brCSCs.^39^ Mammospheres exhibited fragmented mitochondrial morphology and reduced expression of the fission/fusion markers, indicating repressed dynamic activity in comparison to the cancer cells. Mitochondrial elongation impairs metastasis in breast cancer, a key attribute of the CSCs.^40^ This finding reinforces our observations, suggesting that the presence of fragmented mitochondria within spheroids is essential for sustaining their stemness properties. Furthermore, the mammospheres displayed lower levels of ATP and lactate, coupled with diminished mitochondrial respiration, indicative of a relatively quiescent state with reduced energy demands. This metabolic profile may confer a survival advantage to quiescent mammospheres by potentially enabling evasion of drug interventions.^41^ However, since our assessments were limited to mitochondrial function related to energy generation, definitive conclusions regarding overall mitochondrial function alterations could not be made. As for the consequences on cell growth or survival, the loss of cell viability observed in response to ETC inhibition suggests that despite these mitochondria being fragmented, they still perform essential roles in these cells. While this is unlikely to be related to ATP production, ETC inhibitors could affect lipid synthesis (requiring TCA cycle-derived citrate), amino acid metabolism or nucleotide metabolism. Moreover, regardless of their specific metabolic phenotype, mitochondrial biogenesis remains crucial for both glycolytic and oxidative phosphorylation (OXPHOS)-dependent CSCs,^42,43^ underscoring the importance of functional mitochondria in these cells.

Counterintuitively, despite the diminished presence of the master fission regulator DRP1, mammospheres still exhibited fragmented mitochondrial morphology. Interestingly, the fission regulatory protein FIS1, capable of blocking mitochondrial fusion,^44^ was found to be consistently overexpressed in the mammospheres. Substantial evidence has linked FIS1 to the regulation of cancer stemness through promotion of fragmentation-related mitophagy.^45^ Collectively, our study suggests that albeit DRP1 was downregulated, sustained expression of FIS1 possibly maintained the fragmented phenotype in the mammospheres. However, the role of FIS1 in context of the brCSCs warrant further exploration.

Increased expression of DRP1 has often been associated with cancer cell death^32^ and it is well-established that BAX and DRP1 directly interact during apoptosis.^46^ *YAP/TAZ* knockdown significantly increased the levels of both BAX and DRP1 in the mitochondria, a shift that correlated with increased apoptosis. Additionally, YAP/TAZ were shown to interact with DRP1 in the cytoplasm, suggesting that YAP/TAZ plays a pivotal role in maintaining mitochondrial homeostasis, potentially through finely modulating the expression of DRP1 to support brCSC maintenance. Concurrent silencing of both *YAP* and *TAZ* promoted DRP1 localization to the mitochondria, suggesting that both co-activators play equal integral roles in regulating breast cancer cell survival.

YAP and TAZ share analogous structural characteristics, with a TEAD-binding domain (TBD) at the N-terminus, and transcriptional activation domains (TAD), featuring a PDZ-binding motif and a coiled-coil region, at the C-terminus. Notably, YAP is distinguished from TAZ by the presence of an additional SH3-binding domain.^47^ To identify the specific domain through which these co-activators engage with SOX2 and DRP1, we strategically selected YAP for defining the interaction congruence. Deletion analysis confirmed that both SOX2 and DRP1 interacted with YAP in the TBD. Our results further suggested that the TBD of YAP switches its binding to either SOX2 or DRP1 depending on its subcellular localization. In the cytoplasm, YAP interacted with DRP1, whereas upon translocation to the nucleus, YAP associated with SOX2. Under both circumstances, the stemness of brCSCs was maintained. Thus, developing drugs to competitively inhibit the TBD may offer promising therapeutic strategies for manipulating survival of the brCSCs.

Though there are few reports describing VP/YAP interactions, information regarding VP/TAZ interaction is lacking. Docking analysis confirmed that similar to YAP, VP can efficiently interact with TAZ at the TBD, which is pivotal for binding to both SOX2 and DRP1, via hydrogen bonding or hydrophobic interactions, and is fundamentally essential for the preservation of stemness. This competitive interference of VP with SOX2 and/or DRP1 may provide a plausible explanation for the observed disruption of stemness in brCSCs following VP treatment. In addition to its interaction with the TBD, VP also induced an increase in the levels of 14-3-3σ, a chaperone protein that facilitates the retention of YAP in the cytoplasm, thereby promoting its proteasomal degradation.^48^ The observed reduction in YAP/TAZ and SOX2 expression following VP treatment eventually disrupted formation of the YAP/TAZ/SOX2 complex. Therefore, the more pronounced effects of VP treatment, in comparison to the gene silencing effects, can be attributed to its dual action of targeting both the expression and function of YAP/TAZ. Furthermore, since VP simultaneously targets both co-activators, its effects are more substantial than individual depletion of *YAP* and *TAZ*, and supports the potential of VP as a promising therapeutic drug for targeting YAP/TAZ and obliterating the brCSCs (Fig. 8i).

Comprehensive chemotherapeutic treatment has long been a challenge in the management of recurrent breast cancer.^25^ However, emerging studies using cell lines have demonstrated synergistic enhancement of chemotherapeutic efficacy in combination with VP across various cancer types.^49–51^ The biggest advantage of VP as revealed in this study is its efficacy in specifically targeting the brCSCs derived from patient tumors, cells lines and mice tumors, unlike the conventional chemotherapeutic drug paclitaxel. Combining VP with paclitaxel could potentially sensitize the brCSCs to the anti-cancer effects of both paclitaxel and VP, and block the critical escape pathway of brCSCs, mitigating treatment resistance. Additionally, combination with VP reduced the dose of paclitaxel during treatment of patient-derived organoids, with the promise of reducing systemic toxicity and risks of severe side effects. Considering the prolonged and costly trajectory of novel drug development, the forthcoming era is poised to prioritize the repurposing of existing drugs for heightened efficiency and fiscal prudence.^5^ Hence, strategic repurposing of VP, which is an FDA-approved drug currently used for treatment of age-related macular degeneration,^52^ holds significant promise as an effective therapeutic option for patients with triple negative breast cancer, in mono- or combination therapy.

## Materials and methods

### Human ethics approval

Normal and tumor breast tissues were obtained from cancer patients at the Department of Surgery, Medical College, Kolkata, India (MC/KOL/IEC/NON-SPON/204/12-2015), as per the Institutional Review Board and in accordance with the Institutional Human Ethical Committee. All the samples were procured following informed consent from patients, and processing of human tissues for different experiments were performed according to the mandates of the Institutional Human Ethical Committee.

### Animal ethics approval

All animal experiments were conducted in accordance with the guidelines of principles of laboratory animal care (NIH publication no. 85-23, revised in 1985) and also in accordance with the guidelines and approval of the Institutional Animal Ethical Committee, Government of India (Registration Number 885/GO/RE/S/05/CPCSEA; UC/ZOO/2024/1). Throughout the experimental schedule, specific Indian Laws of Animal Protection (ILAP) were followed.

### Human breast tissue sample procurement

Primary-site triple negative breast tumors (TNBCs), either naïve or chemo-treated, were exclusively used in this study. Normal tissues were extracted at a distance of 6 cm from or diagonally opposite to the tumor site via MRM (modified radical mastectomy).^25^ For chemotreated patient samples, tumors exceeding 5 cm in size were included in the study. Prior to surgery, patients underwent 4 cycles of TAC (taxane, adriamycin, and cyclophosphamide). Tumors from non-responders or partial responders were excised out following the completion of the 4-cycle TAC regimen. Patient characteristics are presented in supplementary Table S4.

### Mammosphere culture from patient-breast tumor

Normal breast tissue and patient-breast tumors were mechanically and enzymatically dissociated. Following enzymatic digestion, the cells were seeded at a density of 2 × 10^4^ cells per well in 6-well ultralow attachment plates containing DMEM/F12 supplemented with 5 μg/mL human recombinant insulin, B27 supplement, 20 ng/mL recombinant epidermal growth factor, and antibiotic-antimycotic mix. Mammospheres formed within 7 days were exposed to siYAP/siTAZ or IC_50_ dose of VP for 48 h, after which they were harvested for further analysis.^25^

### Patient-derived organoid culture

Breast tissues obtained from modified radical mastectomy were minced and subjected to enzymatic digestion with collagenase A (1.6 U/mL) for 1–2 h at 37 °C. Red blood cells were first eliminated using ammonium chloride solution. Following digestion, cells from resected tumours were filtered through a 70 μm cell strainer and centrifuged at 300 × *g* for 5 min. Next, cells were resuspended in 40 μl Cultrex Reduced Growth Factor Basement Membrane Extract (BME), Type 2 (R&D Systems), seeded into Matrigel in 24-well plates (Corning), and incubated for 20 min at 37 °C to solidify the Matrigel. Subsequently, 400 μl of complete human organ culture medium (Advanced DMEM/F12 medium containing HEPES, penicillin/streptomycin, Glutamax, B27, R-spondin 1, neuregulin 1, Noggin, EGF, FGF7, FGF10, n-acetylcysteine, primocin, nicotinamide, Y-27632, a selective inhibitor of TGF-βRI, ALK4, ALK7, and p38 inhibitor) was overlayed. The complete media was refreshed every 3–4 days, and organoids were passaged using TrypLE Express (Invitrogen, 12605036) approximately every 2–4 weeks. Viability of the organoids was measured after lysis with CellTiter-Glo (Promega).^53^

### Cell culture

MDA-MB-231, MDA-MB-468 and 4T1 cell lines were procured from the National Centre for Cell Science (NCCS), Pune, India. Cells were maintained in DMEM (HiMedia) supplemented with 10% FBS and 1X antibiotic/antimycotic mix in a 5% CO_2_ incubator at 37 °C. Approximately, 90% confluent cells were dissociated with 0.25% (w/v) trypsin/EDTA and sub-cultured to assess any contamination, cells were routinely tested by PCR.^54,55^

### Drug treatment

Paclitaxel and VP used in the study were purchased from Sigma Aldrich. Both the drugs were reconstituted in DMSO. The control cells were treated with equal volume of DMSO, not exceeding 1%. The control sets comprised untreated cells (designated as Control) and cells treated with an equal volume of DMSO-treated cells (designated as 0). Adherent cells were treated with 2 nM paclitaxel for 24 h and mammospheres were treated with different doses of VP for 48 h. For combination drug treatment, mammospheres pre-treated with 4 µM VP for 48 h, were treated with 7 nM paclitaxel for 24 h. After treatment, cells were harvested for further analysis.

### Mammosphere culture

Breast cancer cells were seeded in 6-well ultra-low attachment plates (Corning) and grown in serum-free DMEM/F-12 (HiMedia) supplemented with 1X antibiotic/antimycotic mixture, B27 (Gibco), 20 ng/ml human epidermal growth factor (Sigma-Aldrich) and 5 µg/mL human recombinant insulin (Sigma-Aldrich).^54^ Generation 1 (G1) mammospheres were subjected to treatments with *YAP/TAZ* siRNA or specific doses of VP/paclitaxel. Mammospheres with a diameter ≥50 μm were manually counted. After treatment, G1 mammospheres were collected, dissociated using 0.05% trypsin and re-plated in ultra-low attachment plates without further treatment to allow the formation of secondary mammospheres (G2). Similarly, G2 mammospheres were processed for the culture of secondary mammospheres (G3). The size and number of spheres were quantified and compared across experimental conditions. Sphere formation efficiency was determined by dividing the total number of spheres formed by the initial number of live cells seeded, multiplied by one hundred.^56^

### Protein extraction and western blot analyses

Cells were initially harvested in RIPA (radioimmunoprecipitation assay) buffer (150 mM NaCl, 50 mm Tris, 0.1% Triton X-100 and 0.1% SDS) containing protease inhibitor cocktail (Abcam). Equal concentrations of proteins were fractionated by polyacrylamide gel electrophoresis and transferred onto PVDF membrane. Protein blots were probed overnight with primary antibodies at 4 °C. Subsequently, the blots were incubated with HRP-tagged secondary antibodies and analysed using Gel Doc XR type imaging system (BioRad). The bands were detected using chemiluminescence and band intensity was quantified using ImageJ software (https://imagej.nih.gov/ij/).^57^ The antibodies used for western blot analysis were as follows: SOX2, YAP, TAZ, β-TUBULIN, BAX, NRF-2, KEAP1, FIS1, UQCRC2, OSCP, cl-caspase3, cl-PARP (Santa Cruz Biotechnology, USA); ALDH1A1, NANOG, COXIV, MFN1 (Abcam); Hippo Signaling Antibody Sampler Kit, pTAZs89, pDRP1, H2B (Cell Signaling Technologies); DRP1, OPA1 (BD Biosciences); NDUFA9 (Invitrogen).

### Immunophenotyping with CD44 and CD24

Breast tissues and cells were suspended in buffer and exposed to antibodies targeting PE-conjugated CD44 (BD Biosciences) and FITC-conjugated CD24 (BD Biosciences), alongside their respective isotype controls. The stained cells were analysed using flow cytometry (BD FACSAria™ III, BD Biosciences, USA and the cell populations sorted out. The threshold settings were determined based on the isotype control of the antibodies utilized. The accuracy of the dual immunostaining was validated through comparison with single immunostaining of CD44 and CD24, respectively. The data were interpreted using BD FACS Diva software.^58^

### Detection of ALDH^+^ population

Aldehyde dehydrogenase (ALDH) enzyme activity was determined using the ALDEFLUOR™ kit (STEMCELL Technologies) following manufacturer’s instructions. Single cell suspensions (1 × 10^6^ cells/mL) were incubated in aldefluor assay buffer containing 5 µL of activated BODIPY-aminoacetaldehyde (BAAA), the ALDH reagent, for 45 min at 37 °C. 5 µL of diethylaminobenzaldehyde (DEAB), a specific ALDH1 enzyme inhibitor, was used as a negative control. ALDH-activity was measured through detecting the fluorescence intensity using FACSAria™ III, BD Biosciences. Thereafter, both ALDH^−^ and ALDH^+^ populations were sorted.^59^

### In silico docking analysis

Primarily, the homology model structures of YAP, TAZ, SOX2 and DRP1 were predicted using AlphaFold.^60^ Protein-protein docking was performed using Patchdock^61^ and HDOCK.^62^ Protein-protein docking was also performed using sequence-based docking module of HDOCK.^62^ The best docked structures were selected according to their scores and binding energy. For docking multiple proteins, Multi-LZerD docking webserver^63^ was utilized. To further validate the docking interactions, the crystal structures of YAP (3KYS), TAZ (5GN0), SOX2 (6WX7) and DRP1 (3W6N) was retrieved from protein data bank (https://www.rcsb.org). Subsequently, protein-protein docking was performed using HDOCK webserver.^62^ After docking, 2D (two dimensional) representations of protein-protein complexes, indicating their interactions, were generated and analyzed using LIGPLOT program.^64^ For protein-protein interactions, DimPlot was employed to study intermolecular interactions using standard input files of Protein Data Bank (PDB). PostScript files were generated as an output. Intermolecular interactions which includes hydrogen bonds and hydrophobic interactions were illustrated. To scrutinize drug-protein interactions, the structure of VP was retrieved from the ZINC20 database.^65^ The CB-DOCK2 webserver was then employed to predict these interactions.^66^ The AutoDock based blind docking approach of CB-DOCK2 server was utilized for the detection of possible binding sites and modes of peptide ligands on scanning the entire surface of protein targets, thereby providing unbiased mapping of the binding patterns of drug candidates.^67^ Followed by docking, the LigPlot analysis was carried out determine the drug-protein interacting residues. PyMOL, used for visualizing complex structures, generated images for the configurations with the lowest binding energies. The PDB files with detailed atomic coordinate information are available publicly at https://www.rcsb.org.

### Subcellular fractionation

Cells were homogenized for 1 min using the STM buffer composed of 250 mM sucrose, 50 mM Tris HCl (pH 7.4), 5 mM MgCl_2_, protease and phosphatase inhibitor cocktails, and kept on ice for 30 min. The homogenate was vortexed for 15 s at maximum speed and centrifuged at 800 × *g* for 15 min. The pellet (P0) and the supernatant (S0) were collected separately. The STM buffer was added to P0 containing nuclei and debris. The pellet was resuspended, vortexed for 15 s, and centrifuged at 500 × *g* for 15 min. The pellet obtained was the nuclear pellet (P1). The P1 pellet was further washed using STM buffer, labelled as P2 and resuspended in NET buffer, comprising 20 mM HEPES (pH 7.9), 1.5 mM MgCl_2_, 0.5 M NaCl, 0.2 mM EDTA, 20% glycerol, 1% Triton-X-100, protease and phosphatase inhibitors, vortexed at maximum speed for 15 seconds and incubated on ice for 30 min. Subsequently, the pellet was sonicated and centrifuged at 9000 × *g* for 30 min at 4 °C, the resultant supernatant, the nuclear fraction, was preserved. From the supernatant S0, the mitochondrial and the cytosolic fractions were isolated. S0 was centrifuged at 800 × *g* for 10 min, followed by centrifugation at 11,000 × *g* for 10 min. The resultant supernatant (S1) and the resultant pellet (P3) were separated. The S1 containing the cytosolic and the microsomal fractions was precipitated in ice-cold 100% acetone at −20 °C for 1 h and then centrifuged at 12,000 × *g* for 5 min. The resultant pellet, resuspended in STM buffer, was the cytosolic fraction. The pellet P3 was resuspended in STM buffer and centrifuged at 11,000 × *g* for 10 min. The supernatant was discarded and the resultant mitochondrial pellet (P4) was resuspended in a buffer composed of 50 mM Tris HCl (pH 6.8), 1 mM EDTA, 0.5% Triton-X-100, protease and phosphatase inhibitors, sonicated on ice for 10 s at high setting with 30 s pauses and the resultant mitochondrial fraction was collected for further experiments.^68^

### Co-immunoprecipitation assay

Cell lysates of the samples were incubated with antibodies for YAP and TAZ protein or with IgG control at 4 °C overnight, to facilitate the formation of immunocomplexes. 25 µL of protein A/G beads were incubated with the lysate/antibody mix for 4 h at 4 °C. Subsequently, the immunoprecipitates were washed, eluted with sample buffer, heated at 100 °C in a water bath for 10 min, resolved using SDS-PAGE and analysed by immunoblotting with anti-SOX2/anti-DRP1/anti-YAP/anti-TAZ antibodies.^69^

### Animals

Female BALB/c mice weighing 20 ± 2 g were procured and maintained under standard laboratory conditions (25 ± 2 °C temperature, 50 ± 15% relative humidity and 12-h light-dark cycles) throughout the experiment. The animals were given food pellets and water ad libitum and allowed to acclimatize to the laboratory environment before commencement of experiments.^57^

### Mice mammary tumor development

Female BALB/c mice (*n* = 3) were used for development of mammary tumors. Viable triple negative mouse mammary carcinoma 4T1 (10^4^ cells in 50 µL of PBS) cells were injected into the inguinal 4th mammary fat pad of mice and tumors were allowed to develop for 14 days. Vehicle-treated mice were injected with 50 µL of PBS. Subsequently, the mammary gland and mammary tumors were excised from vehicle-treated and tumor-bearing mice, respectively. The mammary tissues were further processed for subsequent experiments.^55^

### Plasmids and transfection

For the deletion studies, the YAP protein sequence served as a reference for identifying both the TBD domain and the full-length YAP protein (UniProt ID: P46937). The ΔTBD *YAP* deletion mutants were engineered to create a *YAP* variant that lacks the TBD domain, specifically encompassing amino acids 48-102, as per the methodologies outlined in a previous study.^32^ For ensuring efficient transfer, the PCR-amplified mutant YAP constructs were subsequently cloned into pcDNA 3.1(+) vectors equipped with a FLAG tag. These vectors were then transfected into MDA-MB-231 mammospheres using Lipofectamine® 3000, following the manufacturer’s protocol. After a 48-hour incubation period, the cells were harvested for co-immunoprecipitation analysis using anti-YAP and anti-FLAG antibodies.^32^

### siRNA transfection

RNA transfection was performed using Lipofectamine® 3000 (Thermo Fisher Scientific) according to the manufacturer’s instructions. The mammospheres were grown in 6-well ultra-low attachment plates. 100 pmol YAP/TAZ siRNA duplex (Santa Cruz Biotechnologies) along with 5 µL Lipofectamine® 3000 were added to each well. Non-targeting siRNA was used as negative control. After 48 h of siRNA treatment, the cells were harvested and eventually processed for subsequent experiments.^70^

### RNA isolation and qRT-PCR

RNA was isolated from sorted cells and mammospheres using TRIzol® reagent. Subsequently, reverse transcription was conducted using the Superscript III cDNA synthesis kit. Gene expression levels were determined relative to *18S* RNA using quantitative real-time PCR (qRT-PCR) with SYBR-green chemistry. Each reaction was performed in triplicate, and the resulting products were analyzed using step-one analysis software. Data were presented as 2^−ΔΔCt^ (cycle threshold) values, reflecting the normalization ratio of target mRNA quantities relative to controls. Fold changes compared to controls were calculated for comparison.^25^ The primers and their respective cycling conditions used are detailed in Supplementary Table S5.

### Measurement of ROS levels

The oxidation sensitive fluorescence probe, 2’,7’-dichlorofluorescein diacetate (DCFDA) (Sigma-Aldrich) was used to measure the intracellular ROS levels. Untreated and treated mammospheres were resuspended in PBS. 10 µM DCFDA was added and incubated for 30 min in the dark at 37 °C. Fluorescence was detected using BD Accuri™ C6 Flow Cytometer (BD Biosciences).^71^

### Estimation of ROS scavengers

Superoxide dismutase (SOD) activity was assessed using the WST-1 method, according to the manufacturer’s protocol and the T-SOD activity kit by Elabscience. The activity of SOD is negatively proportional to the yellow coloured formazan and was used to analyse the SOD activity. Reduced glutathione (GSH) content was determined from cell lysates using reduced glutathione (GSH) colorimetric assay kit (Elabscience), based on the reduction of dithionitrobenzoic acid (DTNB) to nitrobenzoic acid and glutathione disulfide. The amount of GSH is directly proportional to the amount of nitrobenzoic acid, with maximum absorption at 420 nm.^72,73^

### Annexin/V PI apoptosis assay

The Annexin V-FITC Apoptosis Detection Kit (Sigma-Aldrich) was employed according to the manufacturer’s protocol. Briefly, untreated and treated cells (1 × 10^6^) were suspended in 100 μL of 1X Annexin binding buffer. To each sample, 5 μL of Annexin V-FITC and 1 μL of PI (100 μg/mL) were added and incubated at room temperature for 15 min. Subsequently, 400 μL of 1X Annexin binding buffer was gently mixed into each sample. The cells were then analyzed using a FACSAria III flow cytometer (BD Biosciences).^74^

### Analysis of OPA1 oligomers

Rapid changes in OPA1 oligomerization was analysed in adherent cells and mammospheres derived from MDA-MB-231 cells after incubating with cell permeable 1 mM BMH (1,6-Bis(maleimido)hexane, Thermo Fisher Scientific) for 20 min at 37 °C. After crosslinking with BMH, cells were quenched, washed twice in PBS with 0.1% beta-mercaptoethanol, and lysed. Western blot on NuPAGE Novex 3–8% Tris-acetate gradient gels was performed to analyse the OPA1 oligomers.^75^

### Immunofluorescence

MDA-MB-231 cells were seeded on glass coverslips and allowed to adhere overnight. Mammospheres were collected in microcentrifuge tubes. Both the adherent cells and mammospheres were fixed in 4% paraformaldehyde for 15 min. Cells were washed in PBS and incubated with permeabilization solution (0.2% Triton-X), followed by blocking with 1% BSA/0.1% Triton-X in PBS. Cells were incubated with primary antibody (anti-TOM20; 1:250; Abcam) in blocking solution, followed by addition of Alexa Fluor 488-conjugated anti-rabbit secondary antibody (1:500; Jackson Immunoresearch). Nuclei were stained using DAPI. The cells were observed using FV 3000 laser-scanning confocal microscope (Olympus). Length analysis categorized a cell into specific groups, based on the lengths of its mitochondria. Cells were therefore classified into categories depending on whether at least 70% of their mitochondria fell within certain length ranges: less than 0.2 µm for fragmented, between 0.2 and 0.6 µm for intermediate, and more than 0.6 µm for elongated.^76^

### TMRM analysis

3 × 10^5^ cells were plated in a 6-well microplate, incubated overnight and exposed to 10 nM TMRM dye (Sigma-Aldrich) for 30 min. For suspension cells, spheres were dissociated into individual cells using trypsin before treatment with 10 nM TMRM for 30 min. Following treatment, adherent cells were rinsed with PBS, trypsinized, and converted into a single-cell suspension. Suspension cells, on the other hand, were collected and washed with PBS. Control cells that had not received TMRM treatment were also collected. Subsequently, cell pellets were resuspended in PBS and analysed using flow cytometry. TMRM fluorescence was assessed using a Cytoflex FACS analyser (Beckman). Fluorescence was also detected using a Leica TSC SP8 confocal microscope.^76^

### ATP and lactate assay

Cells were initially seeded in 96-well plates, which included both transparent and opaque-walled plates, and were incubated overnight at 37 °C. They were further treated with 10 µM oligomycin (Sigma-Aldrich) for 1 h. Subsequently, CellTiter-Glo® reagent (Promega) was added to the cells in the opaque plates at a 1:1 ratio and incubated at room temperature for 10 min in dark. The luminescent signal produced was subsequently quantified using a plate reader set at a gain value of 100%. The transparent plate was employed for lactate and protein quantification. To assess lactate levels, a 10 µL aliquot of media from each well was transferred to fresh wells in duplicate, and the volume was adjusted to 50 µL using PBS. Subsequently, 50 μL of lactate reagent (Promega) was added, and the mixture was incubated for 10 min at room temperature. The resulting absorbance was measured at a wavelength of 450 nm using a plate reader. The media from the transparent plates was removed, leaving the cells at the bottom of the wells. The cells were then rinsed with PBS and subsequently lysed. The protein content of the lysate was determined using the DC protein assay kit (BioRad) and was employed for the normalization of ATP and lactate values.^76^

### Measurement of oxygen consumption rate

Oxygen consumption rate (OCR) was measured using the Mito Stress kit following the manufacturer’s protocol. Prior to the assay, the wells were coated with Corning™ Cell-Tak following manufacturer’s protocol. After incubation, Cell-Tak was aspirated from the plates and washed twice with sterile water at 37 °C. 24 h prior to the experiment, the MDA-MB-231 cells were allowed to attach to the assay plate. The mammospheres were transferred to the Cell-Tak coated wells and were allowed to attach for 20 min in a 37 °C non-CO_2_ incubator. All the cells were allowed to equilibrate in a 37 °C non-CO_2_ incubator for 20 min and analysed using XF assay analyser. OCR was measured by sequentially introducing oligomycin (2 μM), FCCP (1 μM), and rotenone/antimycin A (0.5 μM). Following the experiment, the cells were lysed and protein concentration was estimated to normalize the data.^77,78^

### Trypan blue dye exclusion assay

Trypan blue dye exclusion assay was used to determine the number of viable cells. Equal parts of cell suspensions and 0.4% trypan blue dye (Sigma-Aldrich) were mixed to obtain a 1:1 dilution. 20 µL of the suspension was placed on the hemocytometer. The cells were counted using a phase contrast microscope (ZEISS ProgRes CT3).^79^

### Cell viability assay

MTT assay is employed to assess cellular metabolic activity, serving as an indicator of proliferation, cell viability and cytotoxicity. Briefly, after drug treatment, a final concentration of 0.5 mg/mL MTT reagent (SRL) was added to each well containing the cells and incubated for 3 h at room temperature. The purple formazan crystal was dissolved in DMSO. The absorbance was recorded at 570 nm using Multiskan™ GO microplate spectrophotometer (Thermo Scientific).^25^

### Limiting dilution assay

A single-cell suspension of 4T1 mammospheres (ranging from 1 × 10^4^ to 1 × 10^2^ cells) was prepared in DMEM and injected into the inguinal 4th mammary fat pad of female BALB/c mice. Tumors were allowed to develop for 14 days. Mice were randomly allocated to control and experimental groups. The frequency of breast cancer stem cells (brCSCs) was determined using ELDA (extreme limiting dilution analysis) software available at http://bioinf.wehi.edu.au/software/elda.^80^

### Transmission electron microscopy

Mammospheres were fixed in 2.5% glutaraldehyde (Sigma-Aldrich) and 2% paraformaldehyde (Sigma-Aldrich) in 0.1 M sodium phosphate buffer (pH 7.3) for 20 min at room temperature and for 12 h at 4 °C. After incubation, the cells were washed and fixed in 1% OsO_4_ for 1 h at 4 °C, followed by dehydration in ascending grades of acetone and infiltration. The samples were subsequently embedded in araldite CY212 (TAAB). Thin sections were cut and mounted onto 300 mesh-copper grids and contrasted with alcoholic uranyl acetate and alkaline lead citrate. The samples were then washed with distilled water and observed at an operating voltage of 80 kV under a Morgagni 268 D transmission electron microscope (Fei Company). CCD camera (Megaview III, Fei Company) using iTEM software (Sift Imaging System), attached to the microscope was used to acquire the images.^57^

### Quantification of mitochondrial copy number

The relative mitochondrial DNA (mtDNA) copy number was assessed using quantitative real-time PCR (q-PCR). Specifically, q-PCR was conducted with *ND1* (human NADH dehydrogenase subunit 1) and *β-actin* as the internal control. Amplification utilized SYBR green PCR master mix (Applied Biosystems) following the manufacturer’s protocol. The PCR program consisted of an initial denaturation step at 95 °C for 10 min, followed by 40 cycles of denaturation at 95 °C for 15 s and annealing/extension at their respective annealing temperatures for 1 min. Each sample was analyzed in triplicate. The mtDNA copy number was determined using the delta Ct (∆Ct) method, calculated as the difference between the average Ct values of mtDNA and nuclear DNA (∆Ct = CtmtDNA − Ctβ-actin). The relative mtDNA copy number was then calculated using the 2^−∆∆Ct^ method.^81^ The primers and their respective cycling conditions used are detailed in Supplementary Table S5.

### Statistical analysis

All the data were analysed using Graph Pad Prism 8.0 (GraphPad Software Inc.) and expressed as mean ± SD. Comparison between two groups was done by the unpaired Students’ *t*-test. For multiple group comparisons, two-way ANOVA followed by Tukey’s post hoc test were performed. Differences were considered statistically significant at *p* < 0.05. Minimum of three independent experiments were conducted to allow for statistical comparisons.

## Supplementary information

Supplementary material

Supplementary File

Supplementary files

Data Set 1

## Data Availability

The main text and supplementary information encompass all the essential data. Additional data that corroborate the results of this research are accessible upon request from the first author or the corresponding author.
