# Supplementary material for: Precise targeting of transcriptional co-activators YAP/TAZ annihilates chemoresistant brCSCs by alteration of their mitochondrial homeostasis
Source: Signal Transduct Target Ther. 2025 Feb 21;10:61. doi: 10.1038/s41392-025-02133-x (PMC11842803; doi:10.1038/s41392-025-02133-x)
Supplement: Supplementary file 1 — Supplementary material [file 41392_2025_2133_MOESM1_ESM.docx]

Supplementary Materials for

Precise targeting of transcriptional co-activators YAP/TAZ annihilates chemoresistant brCSCs by alteration of their mitochondrial homeostasis

Priyanka Dey Talukdar^1^, Kunal Pramanik^1^, Priya Gatti^2^, Pritha Mukherjee^1^, Deepshikha Ghosh^3^, Himansu Roy^4^, Marc Germain^2^, Urmi Chatterji^1^*

Correspondence to: urmichatterji@gmail.com

**This file includes:**

Figures S1 to S27

Tables S1 to S5

**Other Supplementary Materials for this manuscript include the following:**

Original Blots

Immunophenotyping Analysis

Flow Cytometry Gating Strategy

**
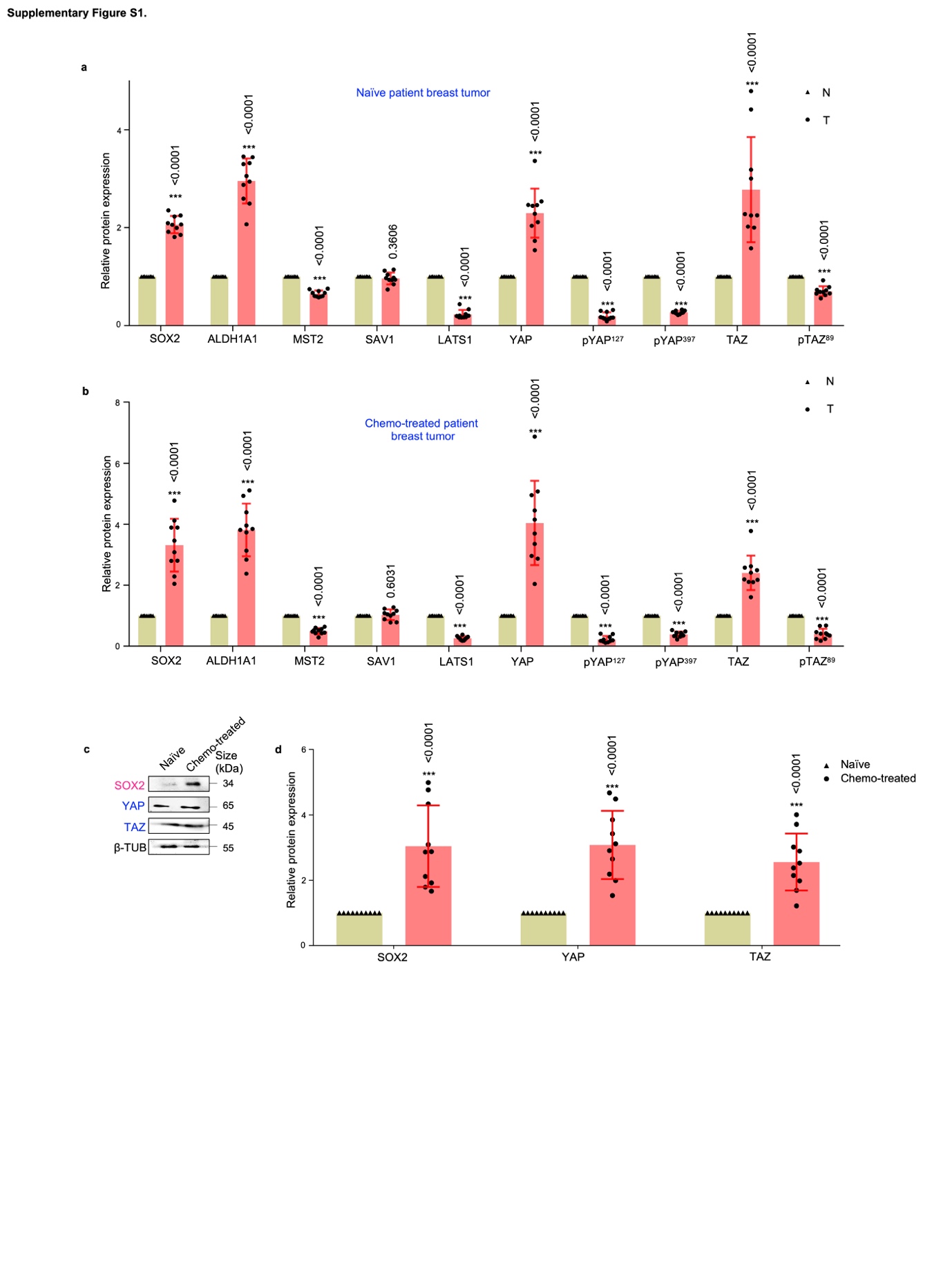
Figure. S1. Transcriptional co-activators YAP/TAZ are upregulated in chemo-treated patient breast tumors**

**a** Western blot quantification of the expression of hippo signaling pathway markers and stemness markers in naïve (n=10) and **b** chemo-treated patient breast tumors compared to their respective adjacent normal tissues (n=10). **c, d** Western blot analyses and quantification of the expression of SOX2, YAP and TAZ in chemo-treated patient breast tumors in comparison to naïve tumor tissues (n=10). All protein expressions were normalized against β-tubulin, which served as the internal loading control. The data are presented as the mean ± standard deviation (SD), with "n" representing the number of biological replicates per experimental group. Significance was assessed using an unpaired Student's t-test, and the associated two-tailed p-value is indicated in the bar plots. Compared to the untreated control group: ***p<0.001. Markers associated with stemness are represented in pink, while markers linked to the Hippo signaling pathway are depicted in blue. N, Normal; T, Tumor.

**
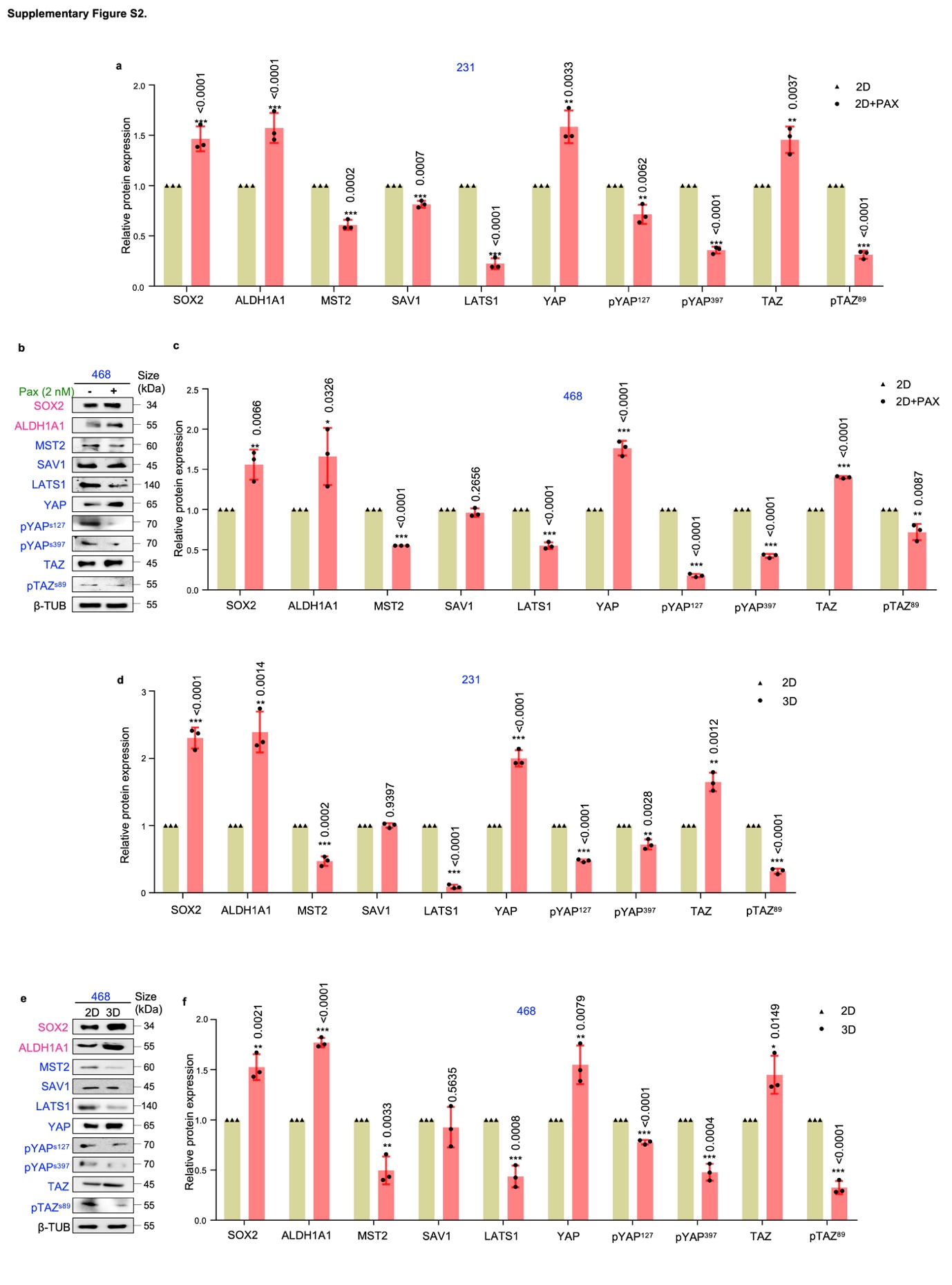
**

**Figure. S2. Transcriptional co-activators YAP/TAZ are upregulated in paclitaxel treated adherent cells and CSC-enriched mammospheres**

**a** Quantification of the expression of hippo signaling pathway markers and stemness markers in untreated and paclitaxel-treated MDA-MB-231 cells (n=3) **b, c** Western blot analysis and quantification of the expression of hippo signaling pathway markers and stemness markers in untreated and paclitaxel-treated MDA-MB-468 cells (n=3). **d** Western blot quantification of the expression of hippo signaling pathway markers and stemness markers in MDA-MB-231 mammospheres in comparison to the adherent cell population (n=3). **e, f** Western blot analyses and quantification of the expression of hippo signaling pathway markers and stemness markers in MDA-MB-468 mammospheres in comparison to the adherent cell population (n=3). All protein expressions were normalized against β-tubulin, which served as the internal loading control. The data are presented as the mean ± standard deviation (SD), with "n" representing the number of biological replicates per experimental group. Significance was assessed using an unpaired Student's t-test, and the associated two-tailed p-value is indicated in the bar plots. Compared to the untreated control group: *p<0.05, **p<0.01 and ***p<0.001. Markers associated with stemness are represented in pink, while markers linked to the Hippo signaling pathway are depicted in blue. Pax, Paclitaxel; 231, MDA-MB-231; 468, MDA-MB-468. 2D, Adherent cells; 3D, Mammospheres; 231, MDA-MB-231; β-TUB, β-tubulin.

**
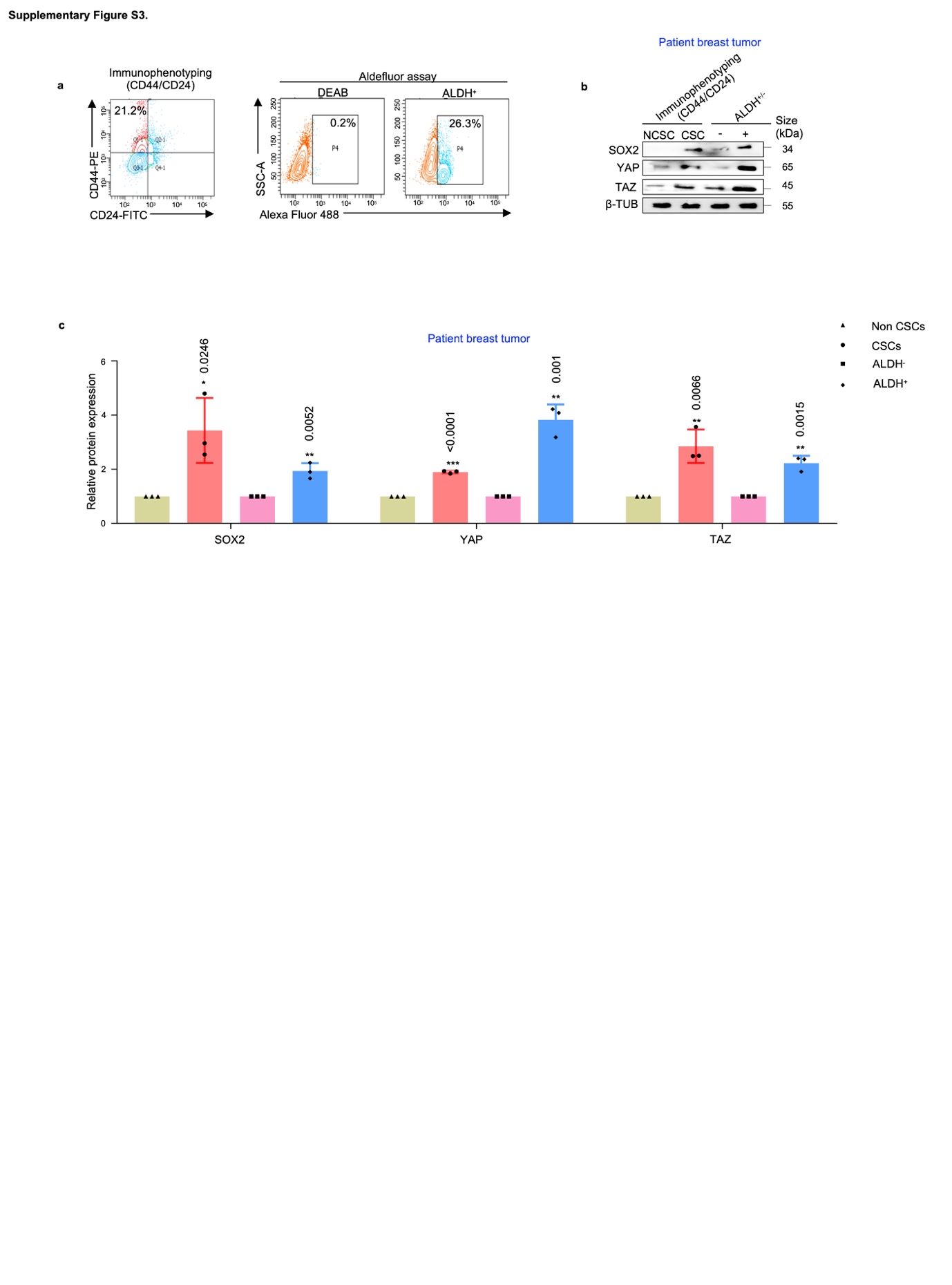
**

**Figure. S3. YAP/TAZ and SOX2 are significantly upregulated in CD44^+^/CD24^-^ and ALDH^+^ brCSC population isolated from patient breast tumor**

**a** Gating strategy for flow cytometry-based isolation of CD44^+^/CD24^-^ and ALDH^+^ cell population from patient breast tumors (n=3). **b, c** Western blot analyses and quantification of the differential expression of SOX2, YAP and TAZ in CD44^+^/CD24^-^ cell population (representing CSCs) in comparison with rest of the cell population (representing non-CSCs) and in ALDH^+^ cell population (representing CSCs) in comparison to ALDH^-^ cell population (representing non-CSCs) (n=3). All protein expressions were normalized against β-tubulin, which served as the internal loading control. The data are presented as the mean ± standard deviation (SD), with "n" representing the number of biological replicates per experimental group. Significance was assessed using an unpaired Student's t-test, and the associated two-tailed p-value is indicated in the bar plots. Compared to the untreated control group: *p<0.05, **p<0.01 and ***p<0.001. NCSC, non-cancer stem cells; CSC, cancer stem cells; ALDH, Aldehyde dehydrogenase.

**
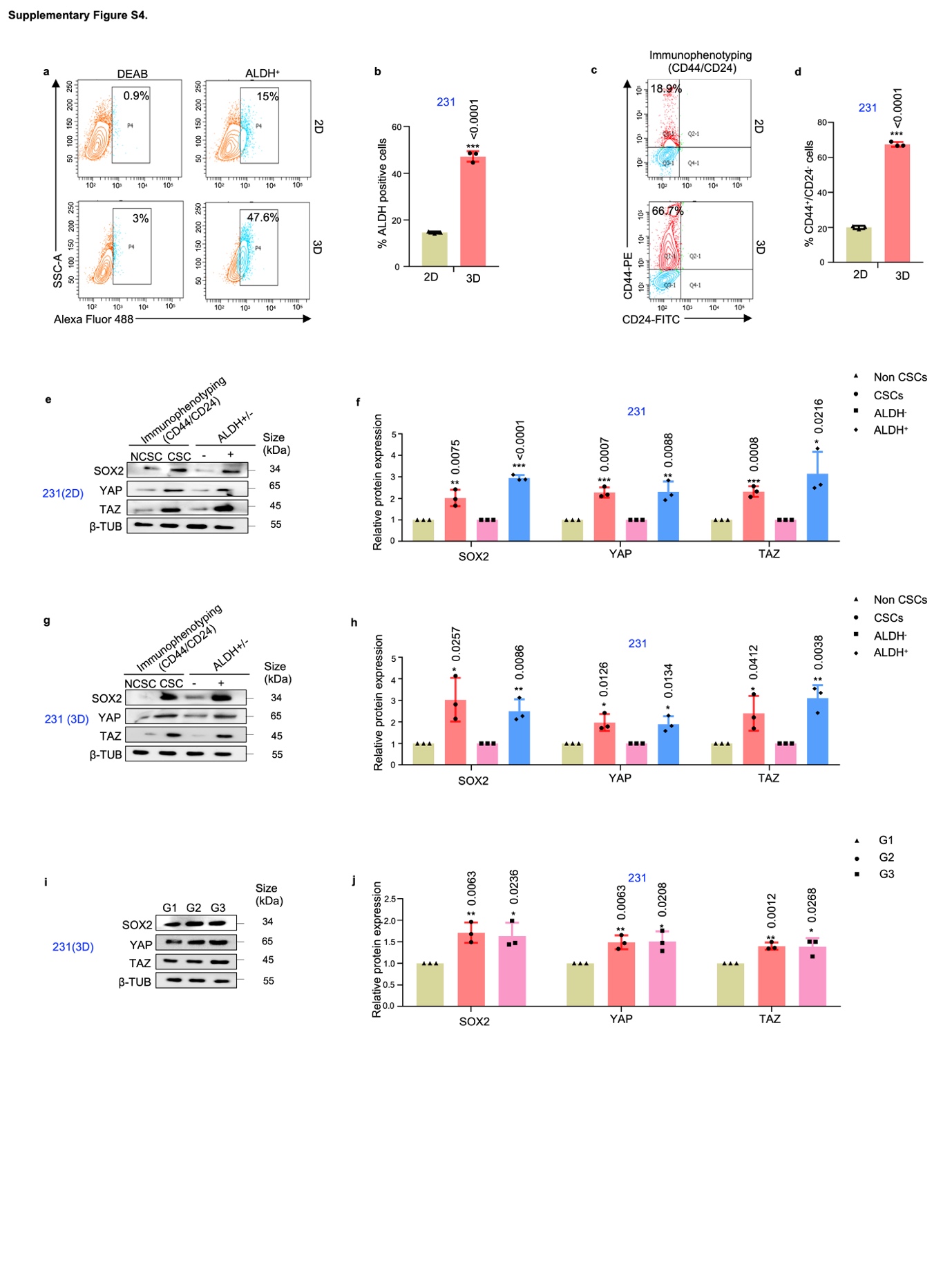
 Figure. S4. YAP/TAZ and SOX2 are significantly upregulated in CD44^+^/CD24^-^ and ALDH^+^ brCSC population isolated from adherent cells and mammospheres**

**a** Gating strategy for flow cytometry-based isolation of ALDH^+^ cell population from MDA-MB-231 adherent cells and mammospheres (n=3). **b** Quantitative analysis of ALDH activity using ALDEFLUOR assay in mammospheres in comparison to adherent cell population (n=3). **c** Gating strategy for flow cytometry-based isolation of CD44^+^/CD24^-^ cell population from MDA-MB-231 adherent cells and mammospheres (n=3). **d** Quantitative analysis of CD44^+^/CD24^-^ cell population in mammospheres in comparison to adherent cell population (n=3) **e, f** Western blot analyses and quantification of the differential expression of SOX2, YAP and TAZ in CD44^+^/CD24^-^ cell population (representing CSCs) in comparison with rest of the cell population (representing non-CSCs) and in ALDH^+^ cell population (representing CSCs) in comparison to ALDH^-^ cell population (representing non-CSCs) in adherent MDA-MB-231 cells (n=3). **g, h** Western blot analyses and quantification of the differential expression of SOX2, YAP and TAZ in CD44^+^/CD24^-^ cell population (representing CSCs) in comparison with rest of the cell population (representing non-CSCs) and in ALDH^+^ cell population (representing CSCs) in comparison to ALDH^-^ cell population (representing non-CSCs) in MDA-MB-231 mammospheres (n=3). **i, j** Western blot analyses and quantification of the differential expression of SOX2, YAP and TAZ in generation 1(G1), generation 2(G2) and generation 3(G3) mammospheres (n=3). All protein expressions were normalized against β-tubulin, which served as the internal loading control. The data are presented as the mean ± standard deviation (SD), with "n" representing the number of biological replicates per experimental group. Significance was assessed using an unpaired Student's t-test, and the associated two-tailed p-value is indicated in the bar plots. Compared to the untreated control group: *p<0.05, **p<0.01 and ***p<0.001. 231, MDA-MB-231; 468, MDA-MB-468; 2D, Adherent cells; 3D, Mammospheres; NCSC, non-cancer stem cells; CSC, cancer stem cells; ALDH, Aldehyde dehydrogenase.


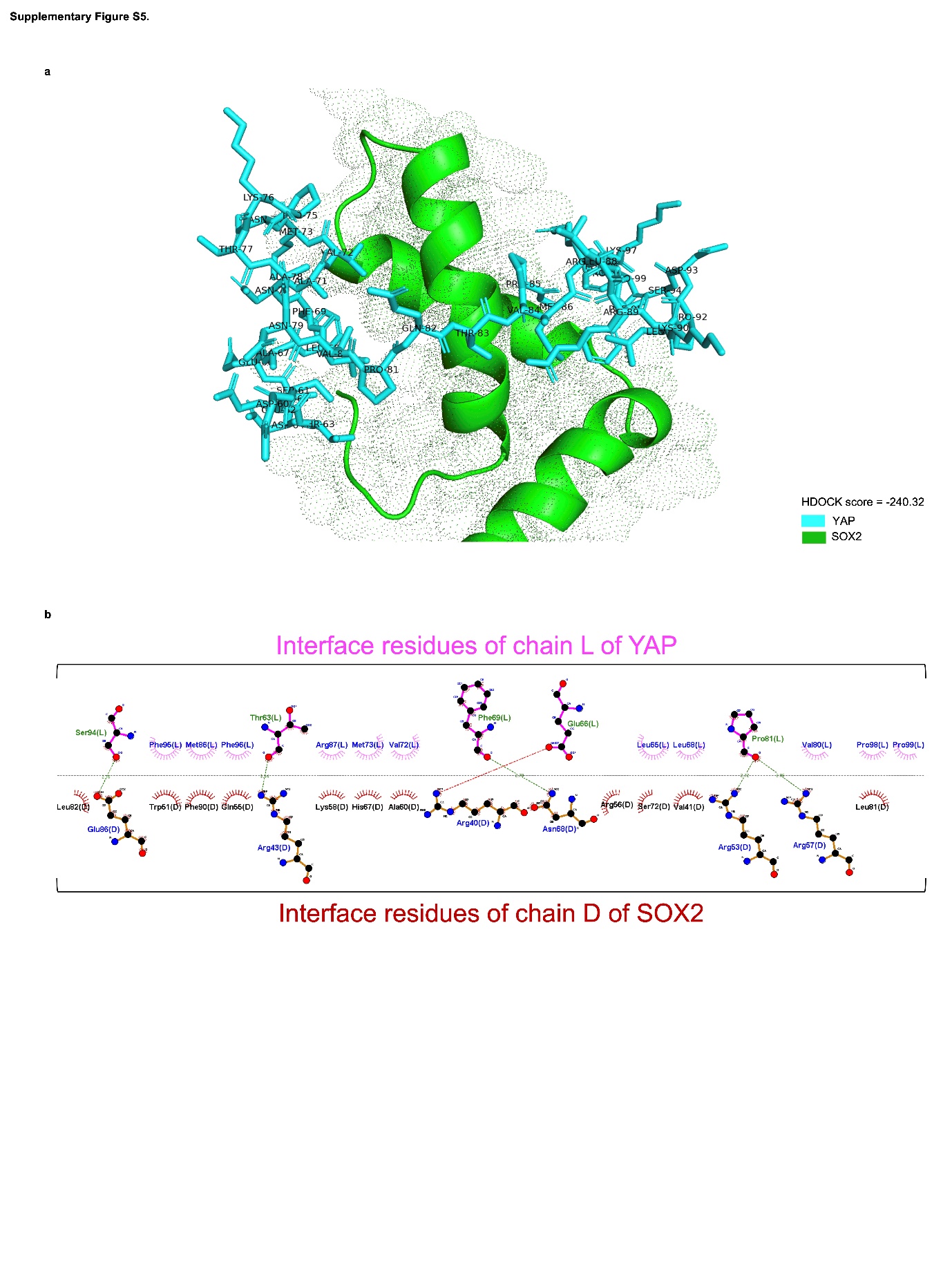


**Figure. S5. In silico docking interaction between SOX2 and YAP**

**a** Molecular docking model depicting the SOX2/YAP interaction. **b** DimPlot 2D-interaction plot illustrates the interactions between SOX2 Chain D and YAP Chain L. Hydrogen bond lengths are depicted by dotted lines, while hydrophobic interactions are represented by arcs. Residues of YAP involved in hydrophobic interactions are highlighted in blue with corresponding purple arcs and residues of SOX2 involved in hydrophobic interactions are highlighted in black with brick red arcs. Residues of YAP engaged in hydrogen bonding are depicted in green, while those of SOX2 involved in hydrogen bonding are shown in blue.


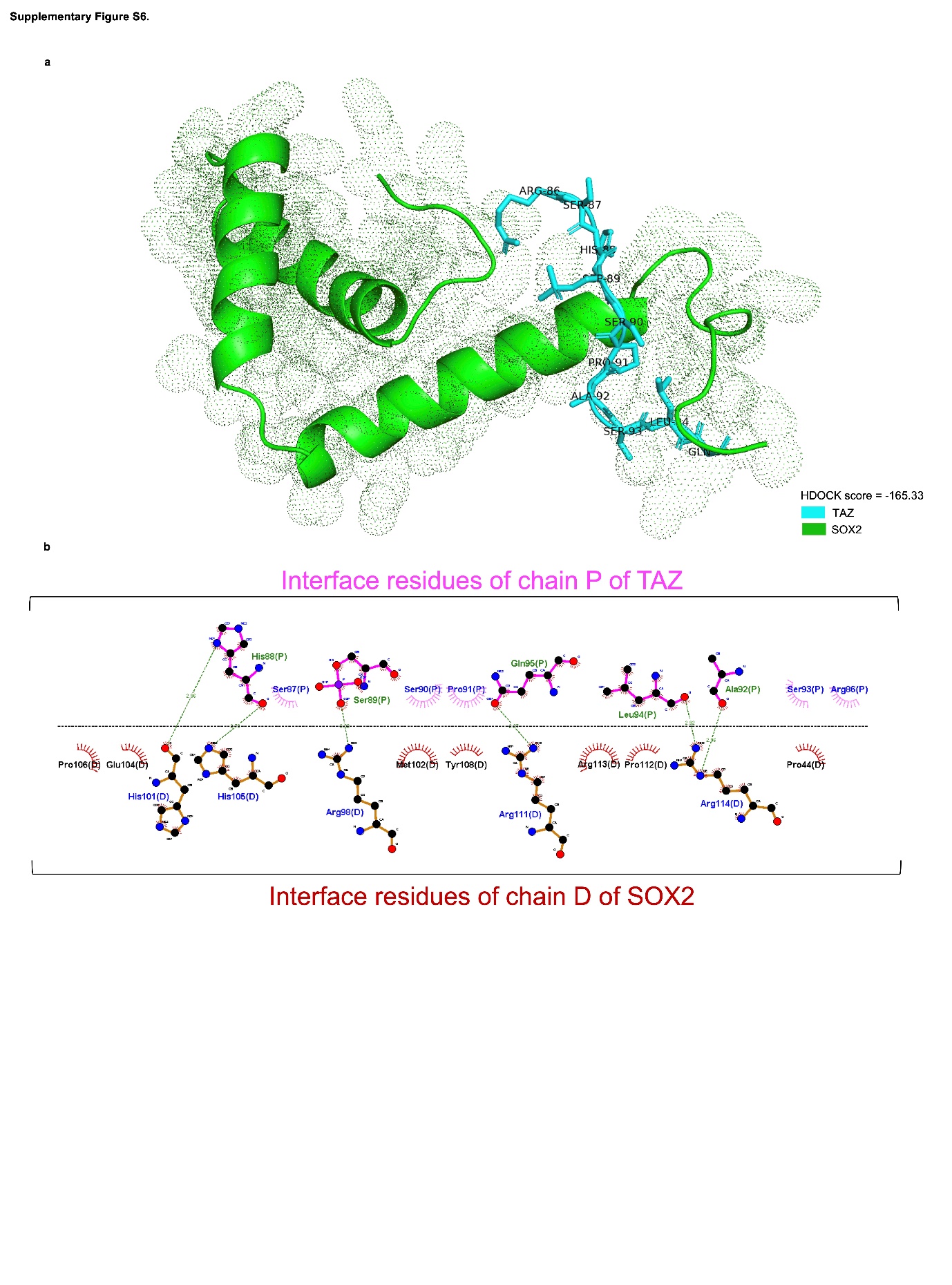


**Figure. S6. In silico docking interaction between SOX2 and TAZ**

**a** Molecular docking model depicting the SOX2/TAZ interaction. **b** DimPlot 2D-interaction plot illustrates the interactions between SOX2 Chain D and TAZ Chain P. Hydrogen bond lengths are depicted by dotted lines, while hydrophobic interactions are represented by arcs. Residues of TAZ involved in hydrophobic interactions are highlighted in blue with corresponding purple arcs and residues of SOX2 involved in hydrophobic interactions are highlighted in black with brick red arcs. Residues of TAZ engaged in hydrogen bonding are depicted in green, while those of SOX2 involved in hydrogen bonding are shown in blue.


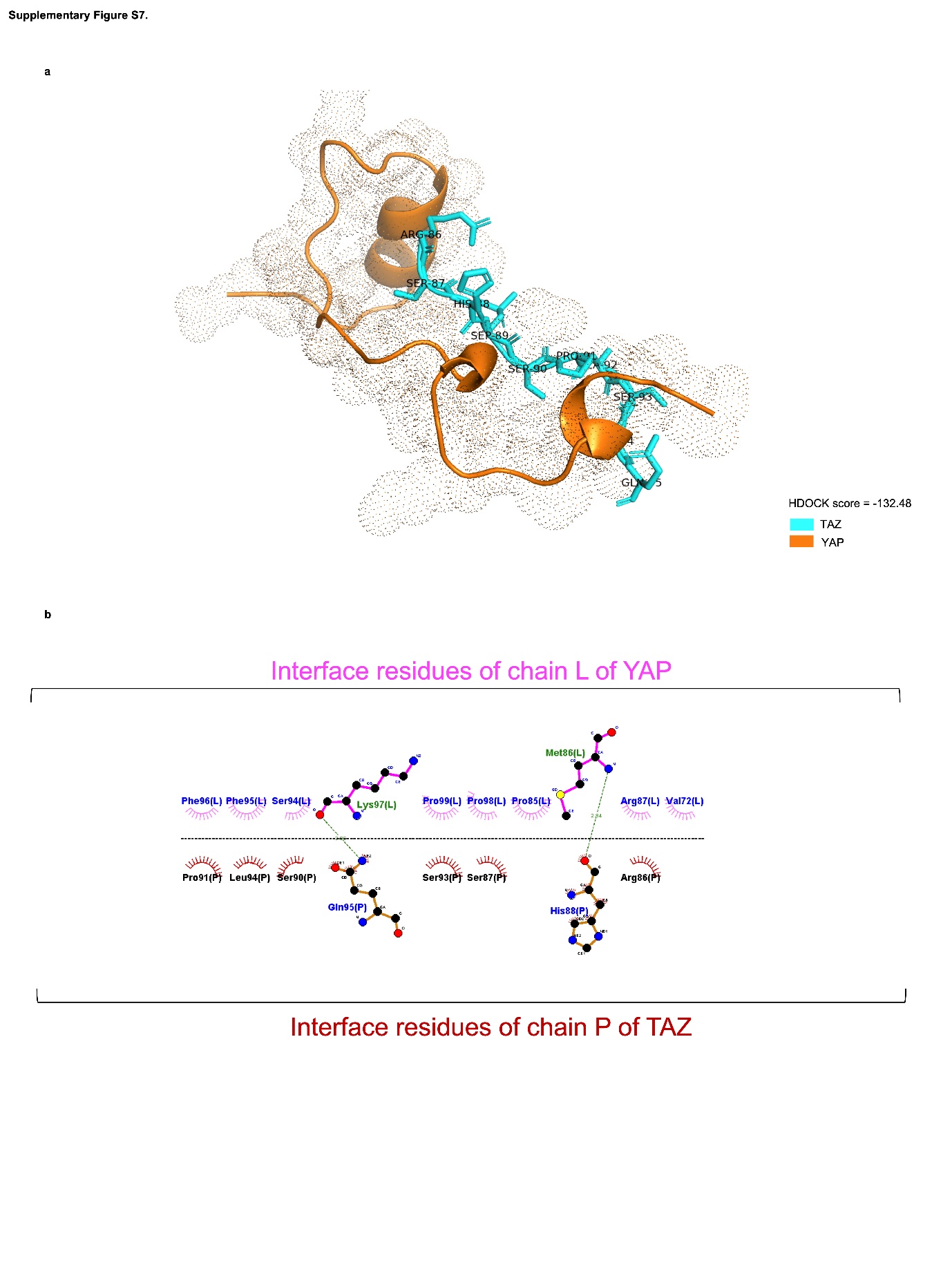


**Figure. S7. In silico docking interaction between YAP and TAZ**

**a** Molecular docking model depicting the YAP/TAZ interaction. **b** DimPlot 2D-interaction plot illustrates the interactions between YAP Chain L and TAZ Chain P. Hydrogen bond lengths are depicted by dotted lines, while hydrophobic interactions are represented by arcs. Residues of YAP involved in hydrophobic interactions are highlighted in blue with corresponding purple arcs and residues of TAZ involved in hydrophobic interactions are highlighted in black with brick red arcs. Residues of YAP engaged in hydrogen bonding are depicted in green, while those of TAZ involved in hydrogen bonding are shown in blue.

**
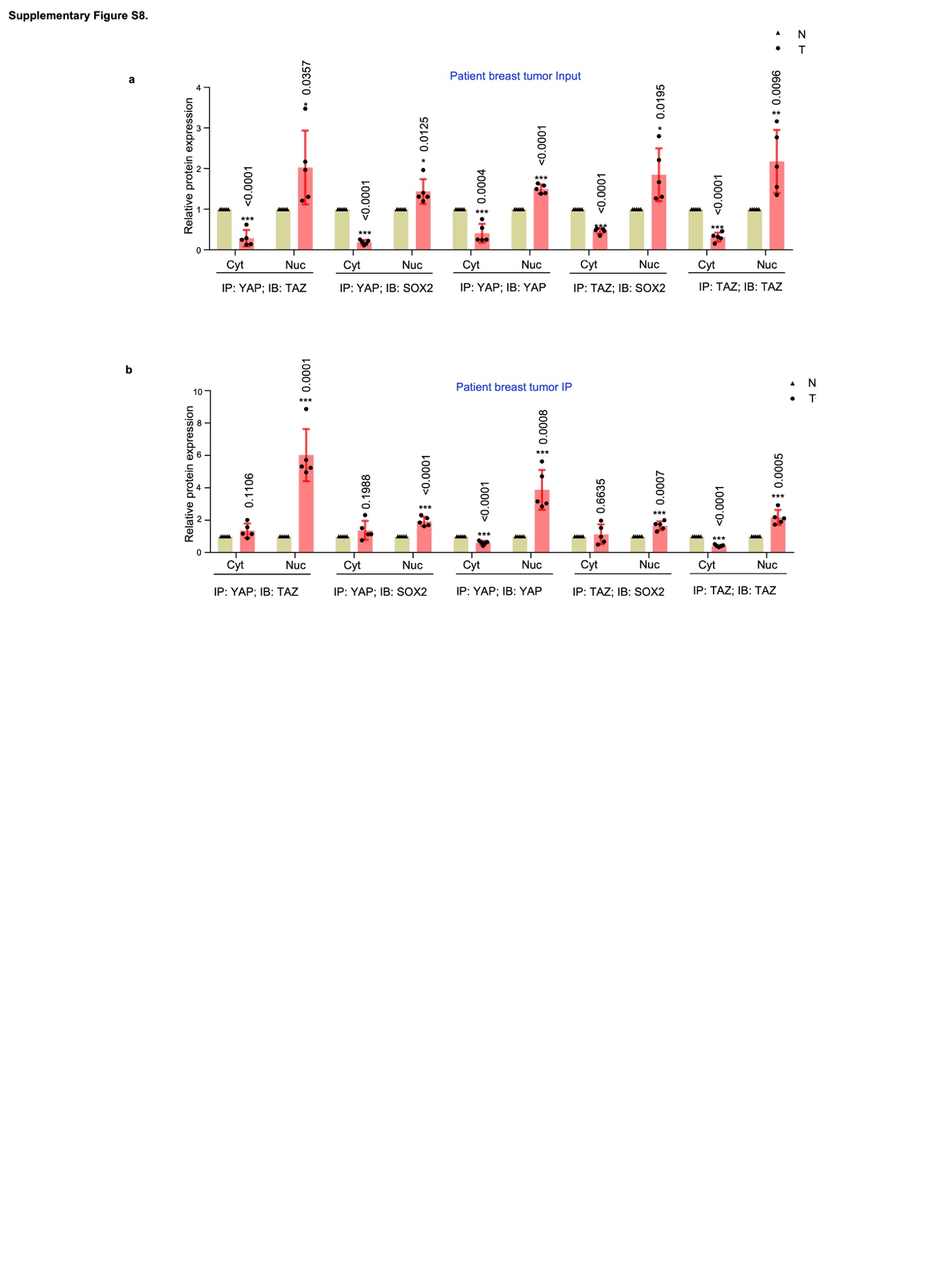
**

**Figure. S8. Co-immunoprecipitation analysis of the interaction between YAP/TAZ and SOX2 in patient breast tumors**

**a, b** Co-IP analyses using either control IgG or antibodies against YAP and TAZ. Western blot quantification was performed to analyse differential interaction pattern in the cytoplasm and nucleus of patient breast tumors in comparison to their adjacent normal (n=5). Cytosolic and nuclear protein expressions were normalized against β-tubulin and H2B respectively. The data are presented as the mean ± standard deviation (SD), with "n" representing the number of biological replicates per experimental group. Significance was assessed using an unpaired Student's t-test, and the associated two-tailed p-value is indicated in the bar plots. Compared to the control group: *p<0.05, **p<0.01 and ***p<0.001. N, Normal; T, Tumor; 231, MDA-MB-231; 468, MDA-MB-468; Cyt/C, Cytoplasm; Nuc/N, Nucleus; IP, Immunoprecipitate; IB, Immunoblot.

**
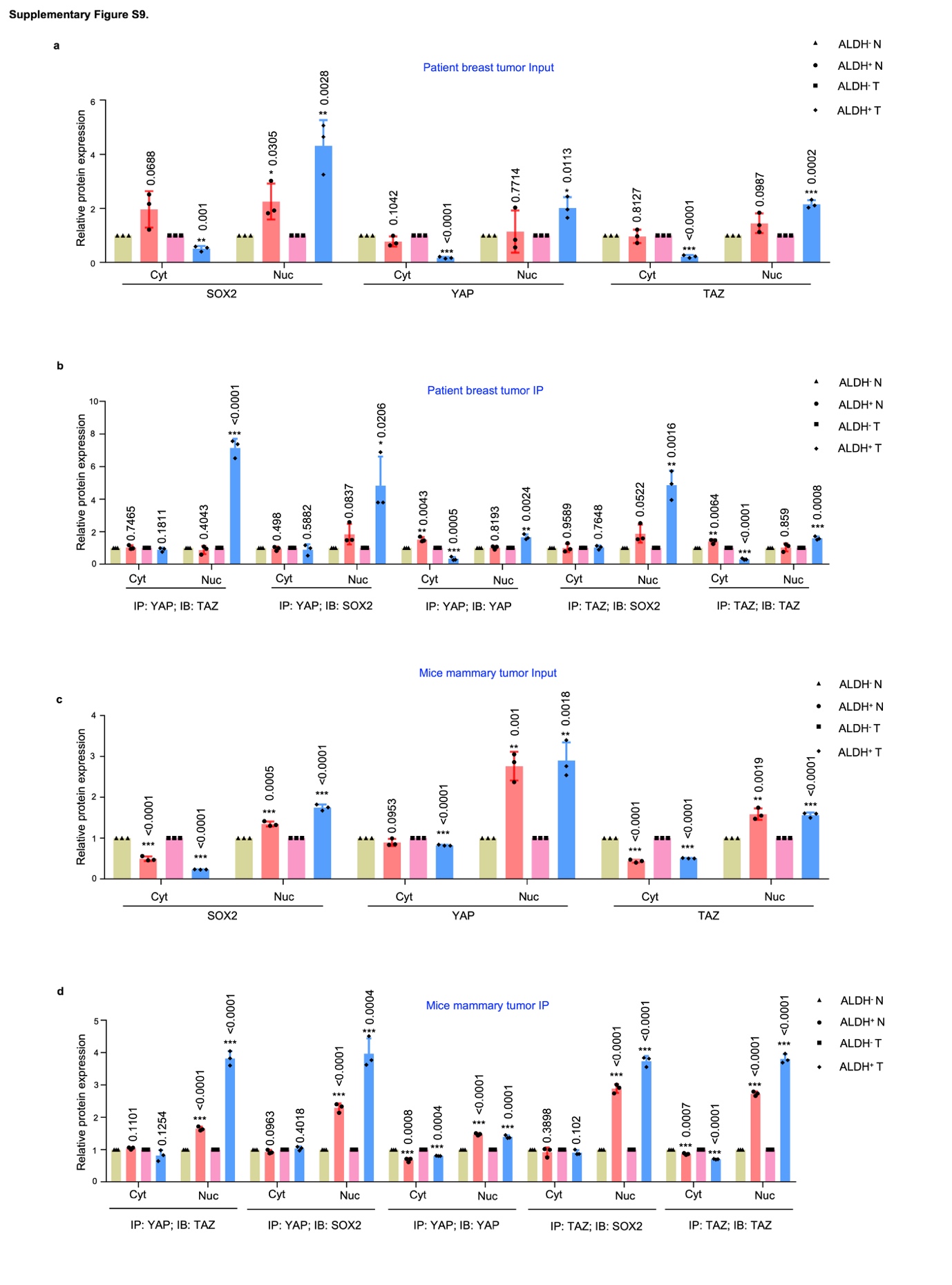
**

**Figure. S9. Co-immunoprecipitation analysis of YAP/TAZ/SOX2 interaction in ALDH^+^ cell population isolated from patient and mice mammary tissues**

**a** Western blot quantification of the expression of SOX2, YAP and TAZ in cytoplasm and nucleus of ALDH^+^ and ALDH^-^ cell population isolated from patient breast tumors and respective adjacent normal tissues (n=3). **b** Co-IP experiments using either control IgG or anti-YAP/anti-TAZ antibodies, followed by western blot quantification in the ALDH^+^ and ALDH^-^ cells isolated from patient breast tumors and respective adjacent normal tissues (n=3). **c** Western blot quantification of the expression of SOX2, YAP and TAZ in cytoplasm and nucleus of ALDH^+^ and ALDH^-^ cell population isolated from normal and 4T1- induced mammary tumor bearing mice (n=3). **d** Co-IP experiments using either control IgG or anti-YAP/anti-TAZ antibodies, followed by western blot quantification in the ALDH^+^ and ALDH^-^ cells isolated from normal and 4T1- induced mammary tumor bearing mice (n=3). Cytosolic and nuclear protein expressions were normalized against β-tubulin and H2B respectively, which served as the internal loading control. The data are presented as the mean ± standard deviation (SD), with "n" representing the number of biological replicates per experimental group. Significance was assessed using an unpaired Student's t-test, and the associated two-tailed p-value is indicated in the bar plots. Compared to the control group: *p<0.05, **p<0.01 and ***p<0.001. N, Normal; T, Tumor; ALDH, Aldehyde dehydrogenase; Cyt, Cytoplasm; Nuc, Nucleus; IP, Immunoprecipitate; IB, Immunoblot.

**
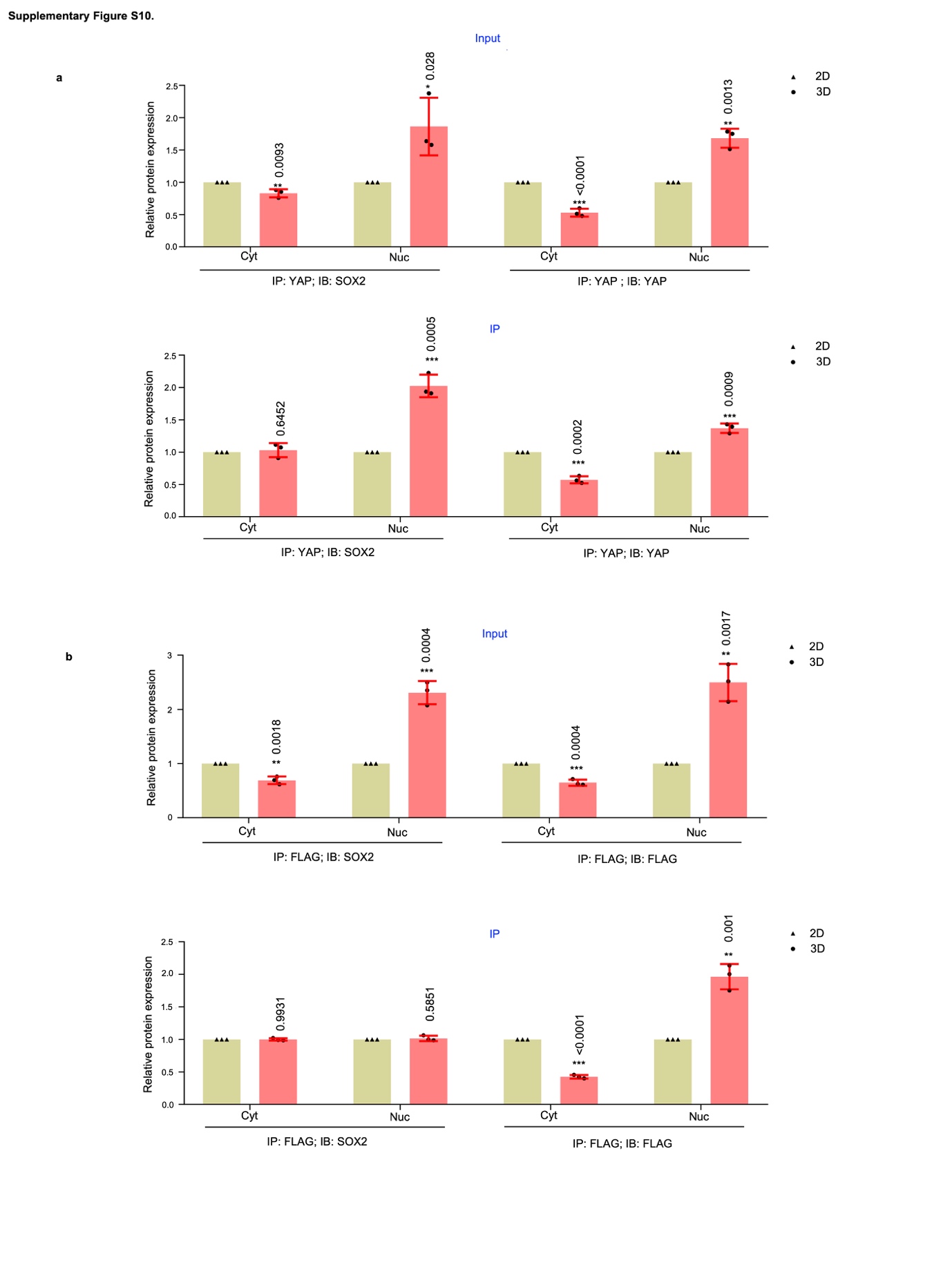
**

**Figure. S10. The TEAD binding domain (TBD) of YAP interacts with SOX2**

**a** Co-IP experiments using either control IgG or anti-YAP antibodies, followed by western blot quantification in the adherent and mammosphere culture of MDA-MB-231 (n=3). **b** *YAP* deletion mutant (ΔTBD) was transfected into MDA-MB-231 mammospheres and immunoprecipitations were performed with anti-FLAG antibody and immunoblots were probed with anti-SOX2 or anti-FLAG antibodies (n=3). Cytosolic and nuclear protein expressions were normalized against β-tubulin and H2B respectively. The data are presented as the mean ± standard deviation (SD), with "n" representing the number of biological replicates per experimental group. Significance was assessed using an unpaired Student's t-test, and the associated two-tailed p-value is indicated in the bar plots. Compared to the control group: *p<0.05, **p<0.01 and ***p<0.001. 2D, Adherent cells; 3D, mammospheres; Cyt, Cytoplasm; Nuc, Nucleus; IP, Immunoprecipitate; IB, Immunoblot.

**
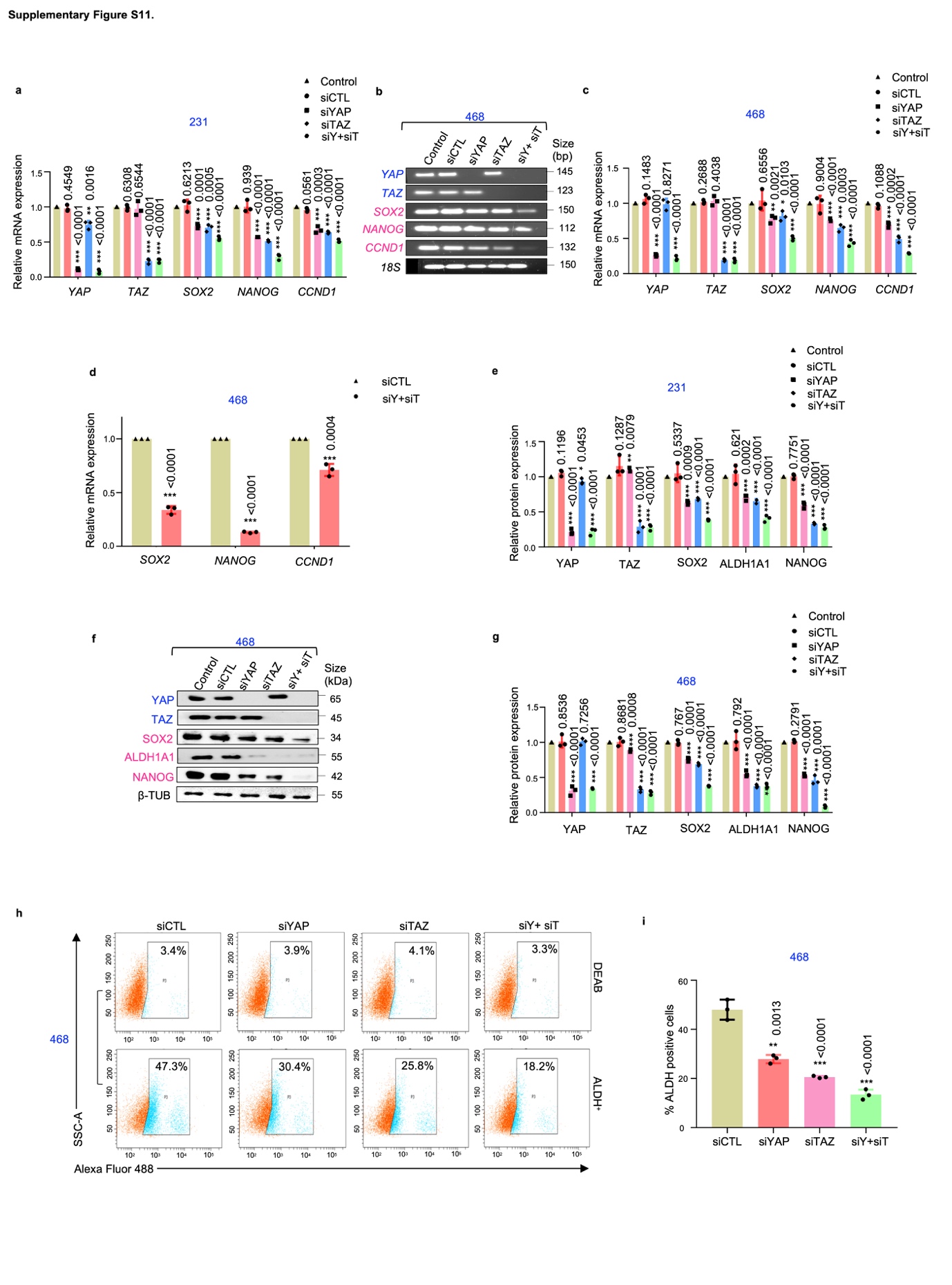
**

**Figure. S11. Inhibition of *YAP/TAZ* results in reduced expression of SOX2 and its downstream target genes, accompanied by a decrease in ALDH activity**

**a** mRNA expression quantification of transcriptional co-activators *YAP/TAZ*, transcription factor *SOX2*, and *SOX2*-target genes *NANOG* and *CCND1* following *YAP/TAZ* knockdown using semi-quantitative PCR in MDA-MB-231 mammospheres (n=3). **b, c** mRNA expression levels and quantification of transcriptional co-activators *YAP/TAZ*, transcription factor *SOX2*, and SOX2-target genes *NANOG* and *CCND1* following *YAP/TAZ* knockdown using semi-quantitative PCR in MDA-MB-468 mammospheres (n=3). **d** Quantitative PCR (qPCR) analysis of *SOX2* and its downstream target genes following depletion of *YAP/TAZ* in MDA-MB-468 mammospheres (n=3). **e** Western blot quantification of the expression of stemness markers SOX2, ALDH1A1, and NANOG following *YAP* and *TAZ* knockdown in MDA-MB-231 mammospheres (n=3). **f, g** Western blot analyses and quantification of the expression of stemness markers SOX2, ALDH1A1, and NANOG following *YAP* and *TAZ* knockdown in MDA-MB-468 mammospheres (n=3). **h** Representative plots and **i** quantitative analysis of ALDH activity using ALDEFLUOR assay in MDA-MB-468 mammospheres following 48 hours of treatment with siRNA targeting *YAP* and *TAZ*. DEAB, an inhibitor staining control, was used to establish the ALDEFLUOR staining intensity threshold (n=3). All mRNA and protein expressions were normalized against *18S* rRNA and β-tubulin respectively, which served as the internal loading controls. Markers associated with hippo signaling pathway are marked in blue and stemness markers are marked in pink. The data are presented as the mean ± standard deviation (SD), with "n" representing the number of biological replicates per experimental group. Significance was assessed using an unpaired Student's t-test, and the associated two-tailed p-value is indicated in the bar plots. Compared to the untreated control group: *p<0.05, **p<0.01 and ***p<0.001. 231, MDA-MB-231; 468, MDA-MB-468; siCTL, control siRNA; siY+siT, siYAP+siTAZ; ALDH, Aldehyde dehydrogenase; 3D, Mammospheres; β-TUB, β-tubulin.

**
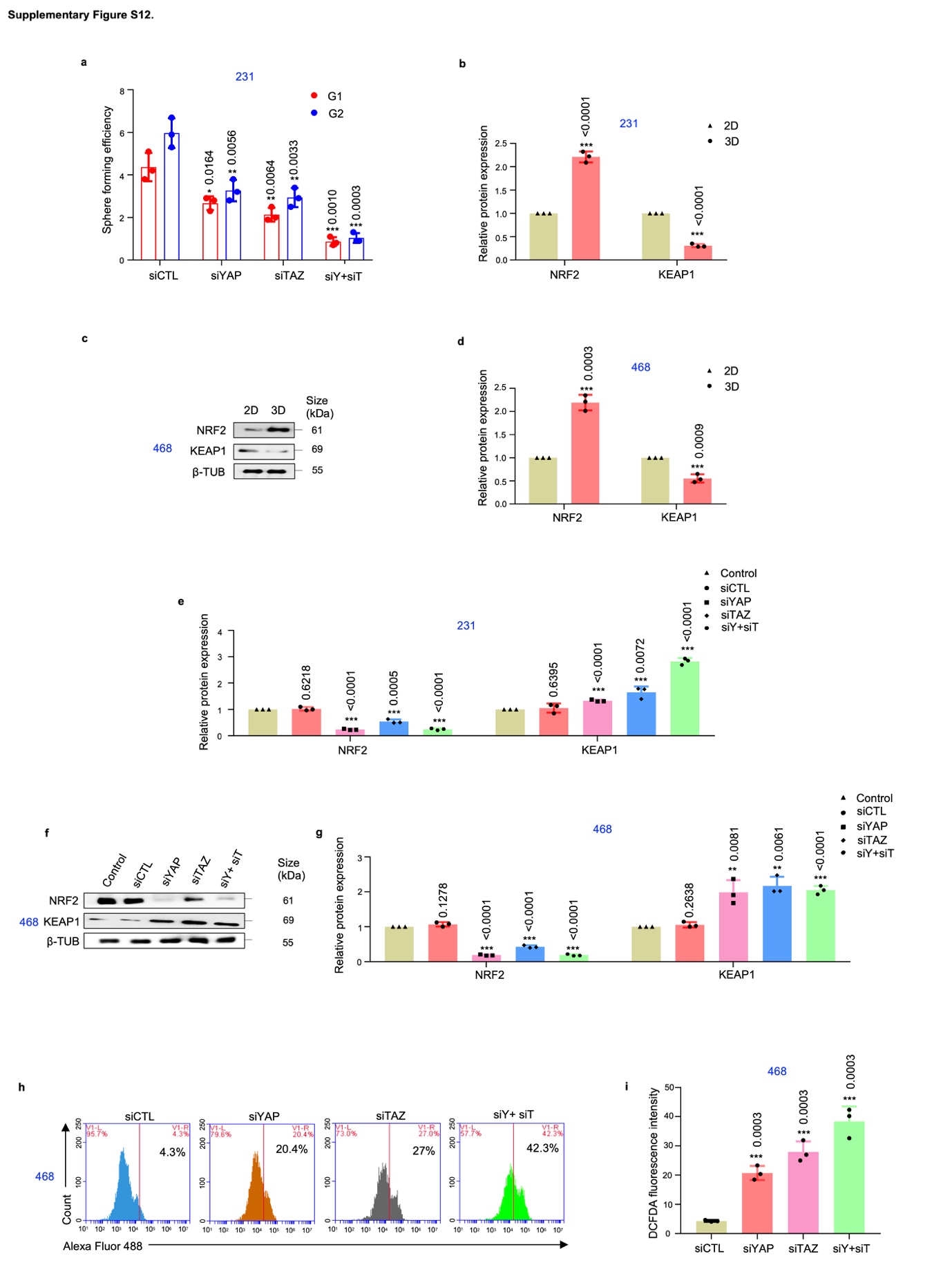
**

**Figure. S12. *YAP/TAZ* disruption leads to reduced stemness accompanied by redox imbalance in the mammospheres**

**a** Sphere forming efficiency of MDA-MB-231 mammospheres over generations upon *YAP/TAZ* silencing (n=3). **b** Western blot quantification of NRF2 and KEAP1 expression in MDA-MB-231 mammospheres in comparison to adherent cell population (n=3). **c, d** Western blot analyses and quantification of NRF2 and KEAP1 expression in MDA-MB-468 mammospheres in comparison to adherent cell population (n=3). **e** Western blot quantification of NRF2 and KEAP1 expression in *YAP/TAZ*-depleted MDA-MB-231 mammospheres (n=3). **f, g** Western blot analysis and quantification of NRF2 and KEAP1 expression in *YAP/TAZ*-depleted MDA-MB-468 mammospheres (n=3). **h, i** Assessment of alterations in reactive oxygen species (ROS) levels following genetic depletion of *YAP* and *TAZ* in MDA-MB-468 mammospheres using H2DCFDA assay (n = 3). All protein expressions were normalized against β-tubulin, which served as the internal loading control. The data are presented as the mean ± standard deviation (SD), with "n" representing the number of biological replicates per experimental group. Significance was assessed using an unpaired Student's t-test, and the associated two-tailed p-value is indicated in the bar plots. Compared to the untreated control group: *p<0.05, **p<0.01 and ***p<0.001. 2D, Adherent cells; 3D, Mammospheres; 231, MDA-MB-231; 468, MDA-MB-468; siCTL, control siRNA; siY+siT, siYAP+siTAZ; β-TUB, β-tubulin.

**
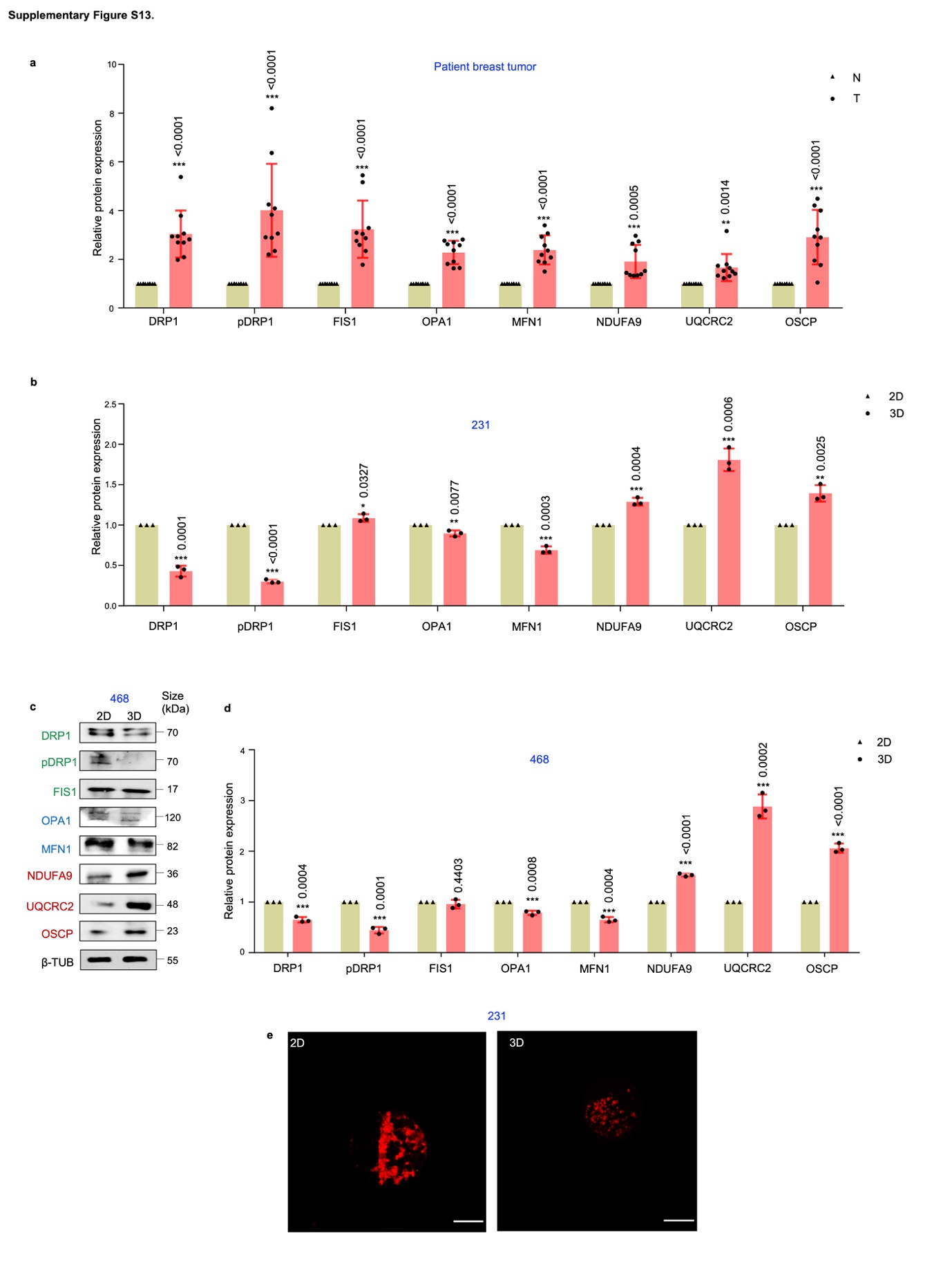
**

**Figure. S13. Differential expression of mitochondrial fission/fusion markers in breast tumors and mammospheres**

**a** Western blot quantification was performed to assess the protein levels of mitochondrial fission markers (DRP1, pDRP1, and FIS1), mitochondrial fusion markers (OPA1 and MFN1), and ETC complex proteins (NDUFA9, UQCRC2, and OSCP) in patient breast tumors compared to adjacent normal tissues (n=10) and **b** in MDA-MB-231 mammospheres in comparison with the adherent cell population (n=3). **c, d** Western blot analyses and quantification of mitochondrial fission markers (DRP1, pDRP1, and FIS1, indicated in green), mitochondrial fusion markers (OPA1 and MFN1, indicated in blue), and ETC complex proteins (NDUFA9, UQCRC2, and OSCP, indicated in red) in MDA-MB-468 mammospheres in comparison with the adherent cell population (n=3). **e** Representative confocal microscopy images depicting TMRM signals in MDA-MB-231 cells and mammospheres (n=3). Scale bar: 10 µm. All protein expressions were normalized against β-tubulin, which served as the internal loading control. The data are presented as the mean ± standard deviation (SD), with "n" representing the number of biological replicates per experimental group. Significance was assessed using an unpaired Student's t-test, and the associated two-tailed p-value is indicated in the bar plots. Compared to their respective control group: *p<0.05, **p<0.01, and ***p<0.001. N, Normal; T, Tumor; 231, MDA-MB-231; 468, MDA-MB-468; 2D, Adherent cells; 3D, Mammospheres; β-TUB, β-tubulin.

**
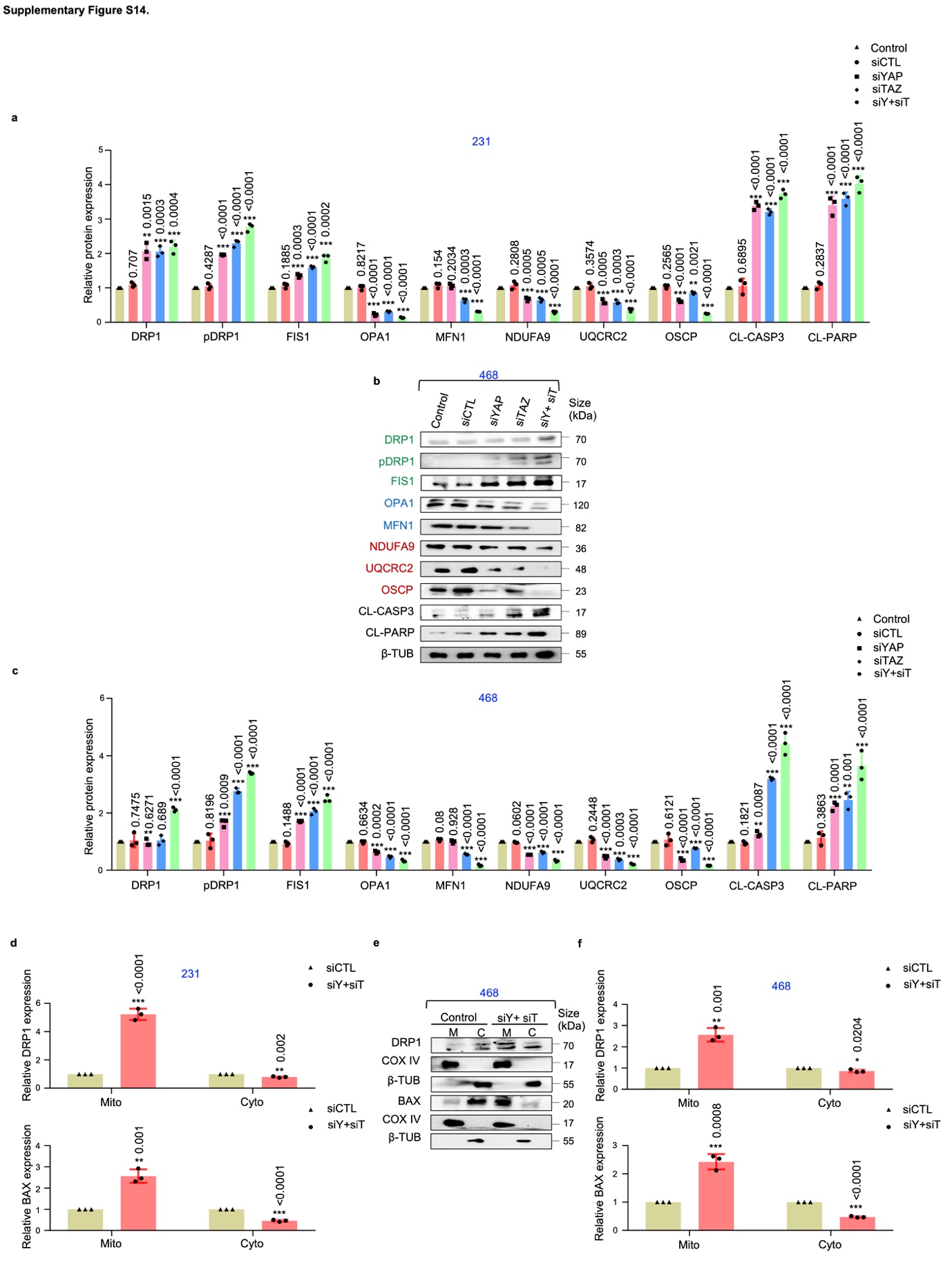
**

**Figure. S14. Alterations in the expression of mitochondrial markers following depletion of *YAP/TAZ***

**a** Western blot quantification of the expression of mitochondrial fission markers (DRP1, pDRP1, FIS1), fusion markers (OPA1, MFN1), ETC complex proteins (NDUFA9, UQCRC2, OSCP) and apoptosis markers (CL-caspase3, CL-PARP) in MDA-MB-231 mammospheres following *YAP/TAZ* depletion (n=3). **b, c** Western blot analyses and quantification of the expression of mitochondrial fission markers (DRP1, pDRP1, FIS1), fusion markers (OPA1, MFN1), ETC complex proteins (NDUFA9, UQCRC2, OSCP) and apoptosis markers (CL-caspase3, CL-PARP) in MDA-MB-468 mammospheres post *YAP/TAZ* silencing. Mitochondrial fission, fusion and ETC complex markers are denoted in green, blue, and red respectively (n=3). **d** Quantification of DRP1 and BAX expression in cytoplasmic and mitochondrial fractions in *YAP/TAZ* depleted MDA-MB-231 mammospheres (n=3). **e, f** Western blot analyses and quantification of DRP1 and BAX expression in cytoplasmic and mitochondrial fractions in *YAP/TAZ* depleted MDA-MB-468 mammospheres (n=3). Cytosolic and mitochondrial protein expressions were normalized against β-tubulin and COX IV respectively. The data are presented as the mean ± standard deviation (SD), with "n" representing the number of biological replicates per experimental group. Significance was assessed using unpaired Student’s t-test, and the associated two-tailed p-value is indicated in the bar plots. Compared to their respective untreated control group: *p<0.05, **p<0.01 and ***p<0.001. 231, MDA-MB-231; 468, MDA-MB-468; siCTL, control siRNA; siY+siT, siYAP+siTAZ; CL-CASP3, cleaved-caspase3, CL-PARP, cleaved-PARP; β-TUB, β-tubulin; Cyt/C, Cytoplasm; Mito/M; Mitochondria.


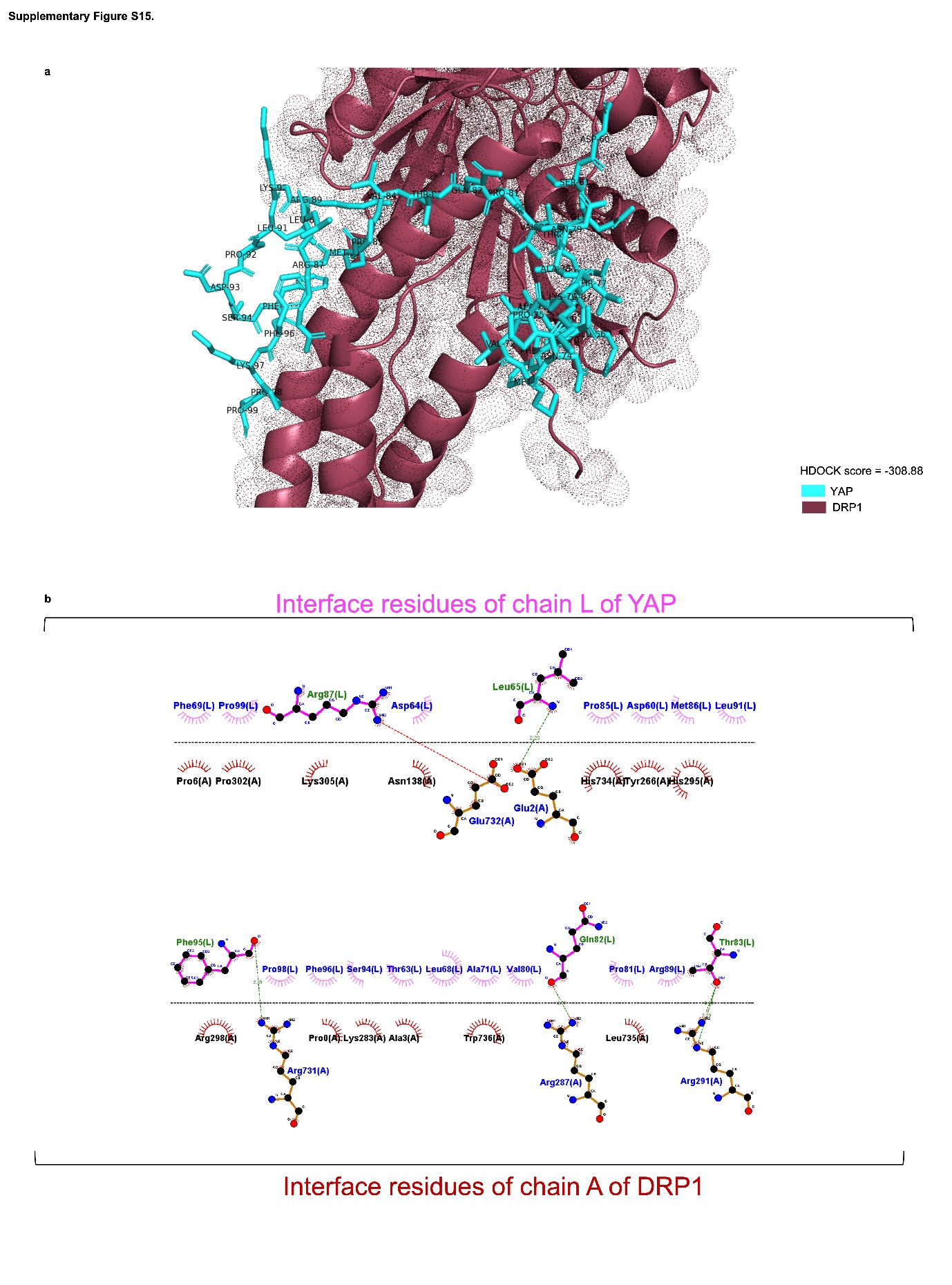


**Figure. S15. In silico docking interaction between YAP and DRP1**

**a** Molecular docking model depicting the YAP/DRP1 interaction. **b** DimPlot 2D-interaction plot illustrates the interactions between YAP Chain L and DRP1 Chain A. Hydrogen bond lengths are depicted by dotted lines, while hydrophobic interactions are represented by arcs. Residues of YAP involved in hydrophobic interactions are highlighted in blue with corresponding purple arcs and residues of DRP1 involved in hydrophobic interactions are highlighted in black with brick red arcs. Residues of YAP engaged in hydrogen bonding are depicted in green, while those of DRP1 involved in hydrogen bonding are shown in blue.


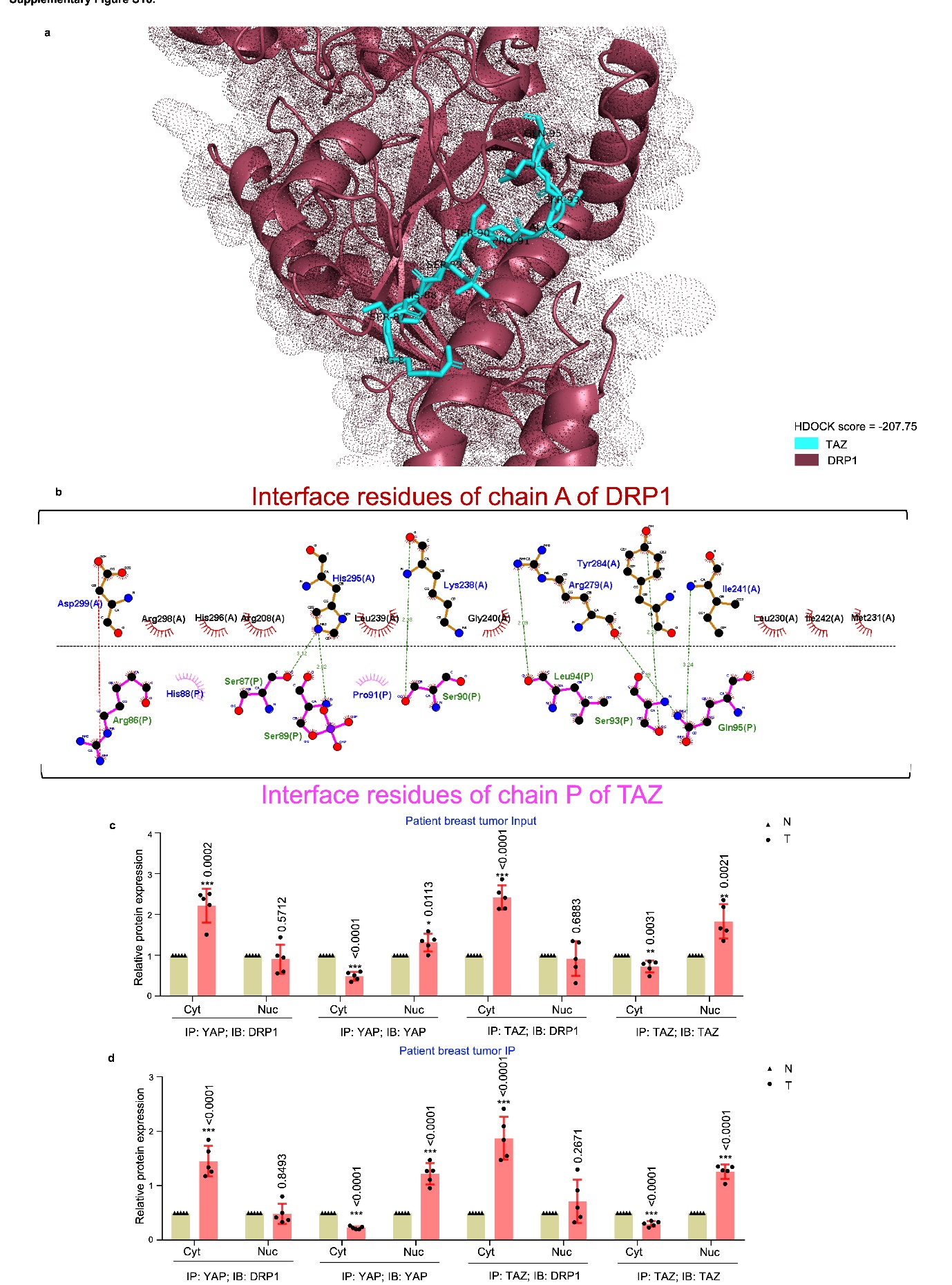


**Figure. S16. In silico and in vitro interaction between YAP/TAZ and DRP1**

**a** Molecular docking model depicting the TAZ/DRP1 interaction. **b** DimPlot 2D-interaction plot illustrates the interactions between TAZ Chain P and DRP1 Chain A. Hydrogen bond lengths are depicted by dotted lines, while hydrophobic interactions are represented by arcs. Residues of TAZ involved in hydrophobic interactions are highlighted in blue with corresponding purple arcs and residues of DRP1 involved in hydrophobic interactions are highlighted in black with brick red arcs. Residues of TAZ engaged in hydrogen bonding are depicted in green, while those of DRP1 involved in hydrogen bonding are shown in blue. **c, d** Co-IP analyses using either control IgG or antibodies against YAP and TAZ. Western blot quantification was performed to analyse differential interaction pattern in the cytoplasm and nucleus of patient breast tumors in comparison to their adjacent normal (n=5). Cytosolic and nuclear protein expressions were normalized against β-tubulin and H2B respectively, which served as the internal loading control. The data are presented as the mean ± standard deviation (SD), with "n" representing the number of biological replicates per experimental group. Significance was assessed using an unpaired Student's t-test, and the associated two-tailed p-value is indicated in the bar plots. Compared to the control group: *p<0.05, **p<0.01 and ***p<0.001. N, Normal; T, Tumor; Cyt, Cytoplasm; Nuc, Nucleus; IP, Immunoprecipitate; IB, Immunoblot.

**
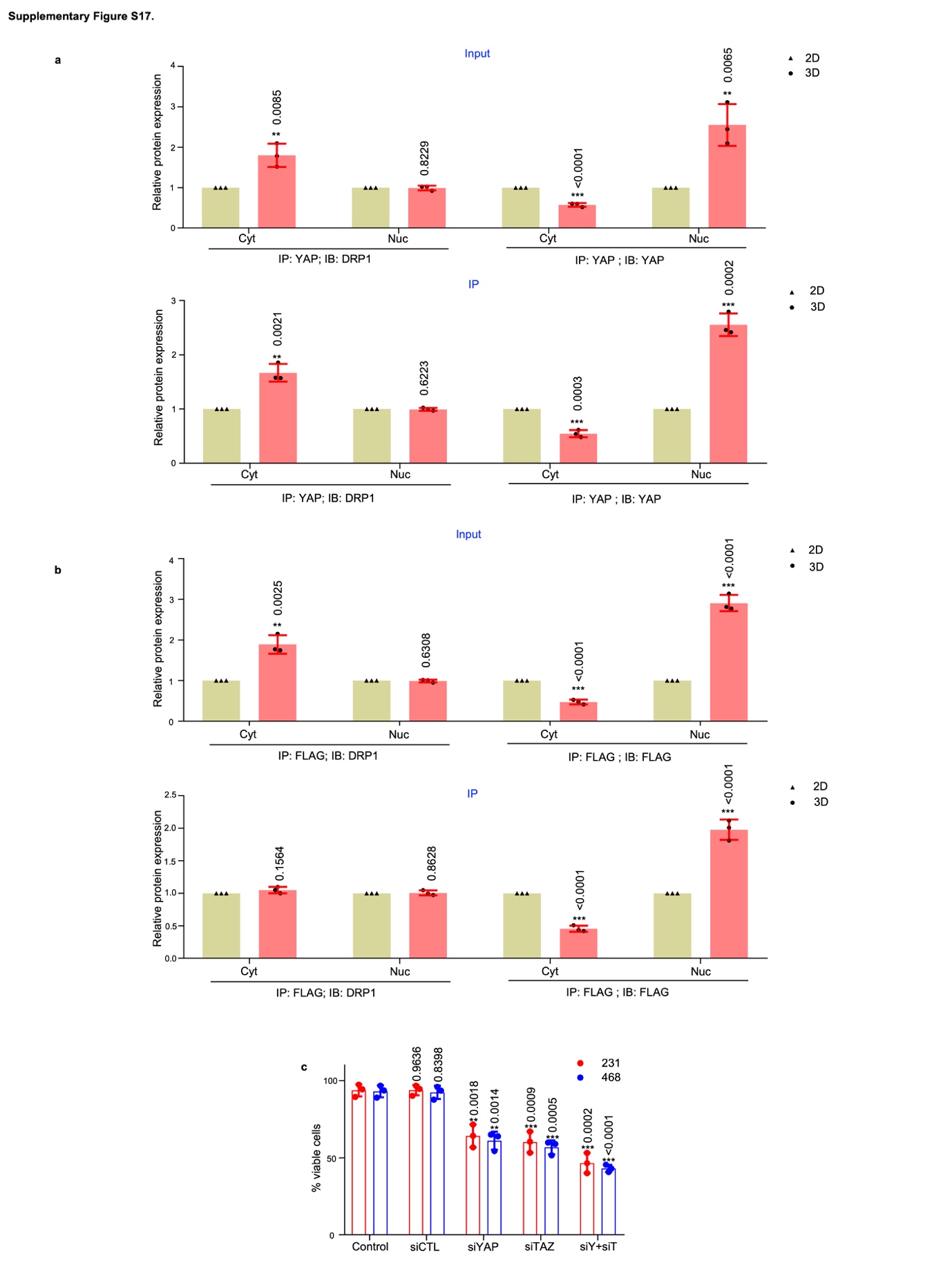
**

**Figure. S17. The TEAD binding domain (TBD) of YAP interacts with DRP1**

**a** Co-immunoprecipitation experiments using either control IgG or anti-YAP antibodies, followed by western blot quantification in the adherent and mammosphere culture of MDA-MB-231 (n=3) **b** *YAP* deletion mutant (ΔTBD) was transfected into MDA-MB-231 mammospheres and immunoprecipitations were performed with anti-FLAG antibody and immunoblots were probed with anti-DRP1 or anti-FLAG antibodies (n=3). **c** Assessment of the percentage viable cells as quantified using trypan blue in the MDA-MB-231 and MDA-MB-468 mammospheres upon siRNA mediated depletion of *YAP* and *TAZ* (n=3). Cytosolic and nuclear protein expressions were normalized against β-tubulin and H2B respectively. The data are presented as the mean ± standard deviation (SD), with "n" representing the number of biological replicates per experimental group. Significance was assessed using an unpaired Student's t-test, and the associated two-tailed p-value is indicated in the bar plots. Compared to the control group: **p<0.01 and ***p<0.001. 2D, Adherent cells; 3D, mammospheres; Cyt, Cytoplasm; Nuc, Nucleus; IP, Immunoprecipitate; IB, Immunoblot; 231, MDA-MB-231; 468, MDA-MB-468.

**
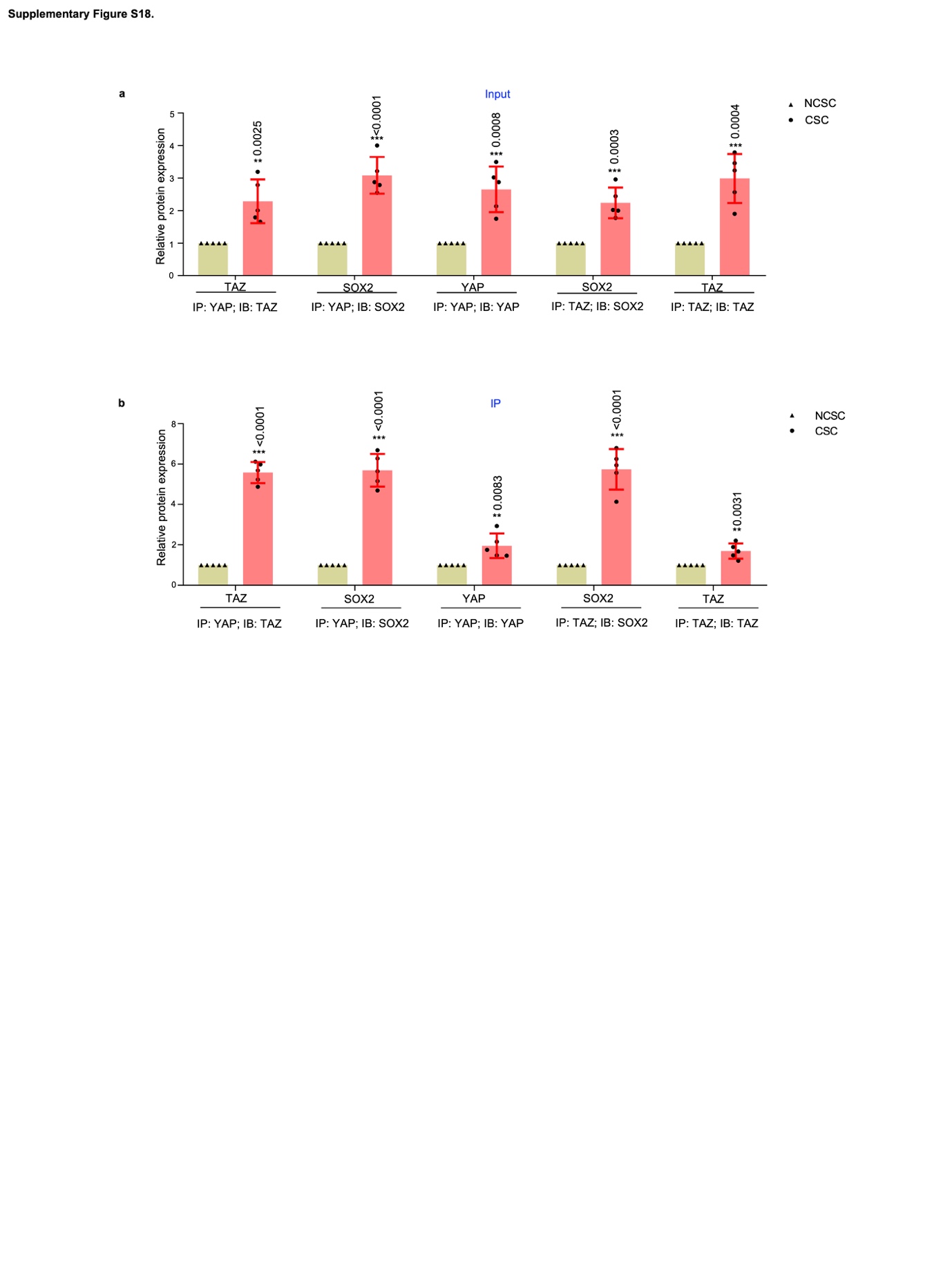
**

**Figure. S18. YAP/TAZ interacts with SOX2 in TNBC patient-derived spheroids a, b** Co-IP analyses using either control IgG or antibodies against YAP and TAZ. Western blot quantification was performed to analyse differential interaction pattern in the nucleus of CD44^+^/CD24^-^ cell population (representing CSCs) in comparison with rest of the cell population (representing non-CSCs) isolated from patient breast tumors (n=5). Nuclear protein expressions were normalized against H2B, which served as the internal loading control. The data are presented as the mean ± standard deviation (SD), with "n" representing the number of biological replicates per experimental group. Significance was assessed using an unpaired Student's t-test, and the associated two-tailed p-value is indicated in the bar plots. Compared to the control group: **p<0.01 and ***p<0.001. NCSC, non-cancer stem cells; CSC, Cancer stem cells; IP, Immunoprecipitate; IB, Immunoblot.

**
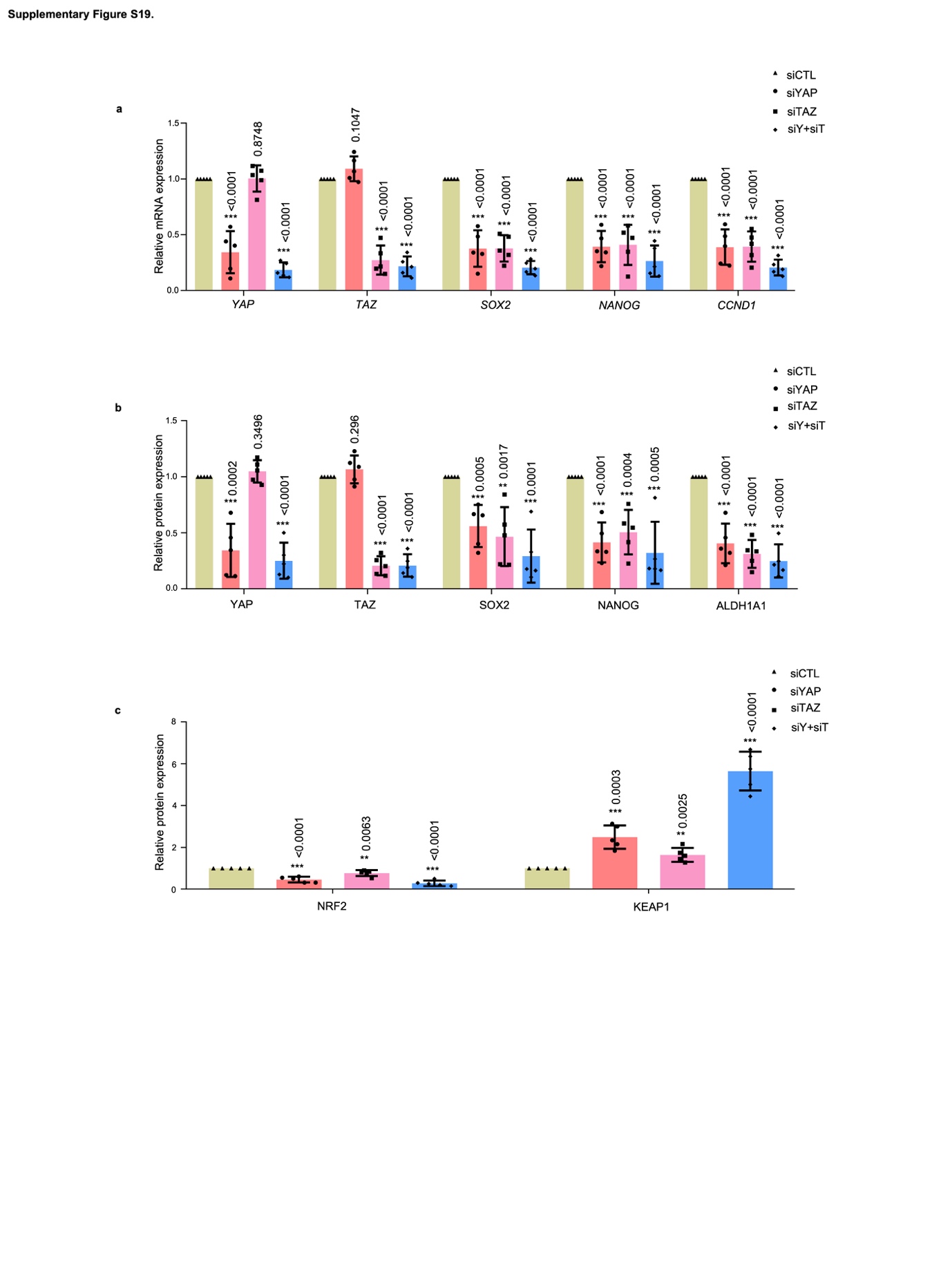
**

**Figure. S19. *YAP/TAZ* depletion leads to decrease in expression of stemness markers and redox regulator NRF2 in TNBC patient-derived spheroids**

**a** mRNA expression quantification of transcriptional co-activators *YAP/TAZ*, transcription factor *SOX2*, and *SOX2*-target genes *NANOG* and *CCND1* following YAP/TAZ knockdown using semi-quantitative PCR in patient-derived spheroids (n=5). **b** Western blot quantification of the expression of stemness markers SOX2, ALDH1A1, and NANOG following *YAP* and *TAZ* knockdown in patient-derived spheroids (n=5). **c** Western blot quantification of the expression of NRF2 and KEAP1 following *YAP* and *TAZ* knockdown in patient-derived spheroids (n=5). All mRNA and protein expressions were normalized against *18S* rRNA and β-tubulin respectively, which served as the internal loading control. The data are presented as the mean ± standard deviation (SD), with "n" representing the number of biological replicates per experimental group. Significance was assessed using an unpaired Student's t-test, and the associated two-tailed p-value is indicated in the bar plots. Compared to their respective control group: **p<0.01, and ***p<0.001. siCTL, Control siRNA; siY+siT, siYAP+siTAZ.

**
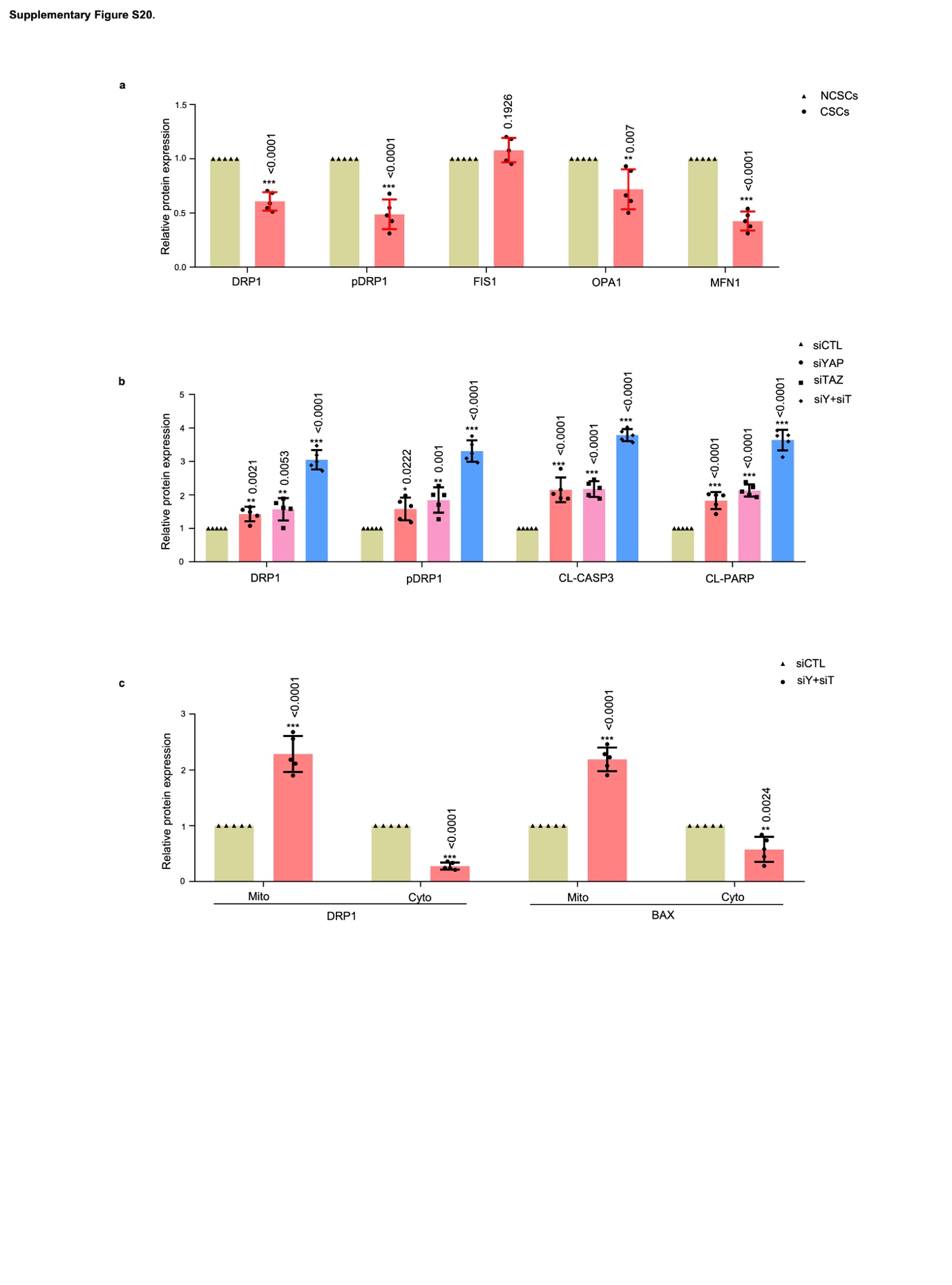
**

**Figure. S20. *YAP/TAZ* depletion promotes upregulation of DRP1 and apoptotic markers in patient-derived spheroids**

**a** Western blot quantification of the expression of mitochondrial fission markers (DRP1, pDRP1, FIS1) and fusion markers (OPA1, MFN1) in CD44^+^/CD24^-^ cell population (representing CSCs) in comparison with rest of the cell population (representing non-CSCs) (n=5). **b** Western blot quantification of the expression of mitochondrial fission markers (DRP1, pDRP1) and apoptosis markers (CL-CASP3, CL-PARP) in patient-derived spheroids (n=5). **c** Quantification of DRP1 and BAX expression in cytoplasmic and mitochondrial fractions in *YAP/TAZ* depleted patient-derived spheroids (n=5). Cytosolic and mitochondrial protein expressions were normalized against β-tubulin and COX IV respectively. The data are presented as the mean ± standard deviation (SD), with "n" representing the number of biological replicates per experimental group. Significance was assessed using unpaired Student’s t-test, and the associated two-tailed p-value is indicated in the bar plots. Compared to their respective untreated control group: *p<0.05, **p<0.01 and ***p<0.001. NCSC, non-cancer stem cells; CSC, Cancer stem cells; siCTL, control siRNA; siY+siT, siYAP+siTAZ; CL-CASP3, cleaved-caspase3, CL-PARP, cleaved-PARP; Cyto, Cytoplasm; Mito; Mitochondria.

**
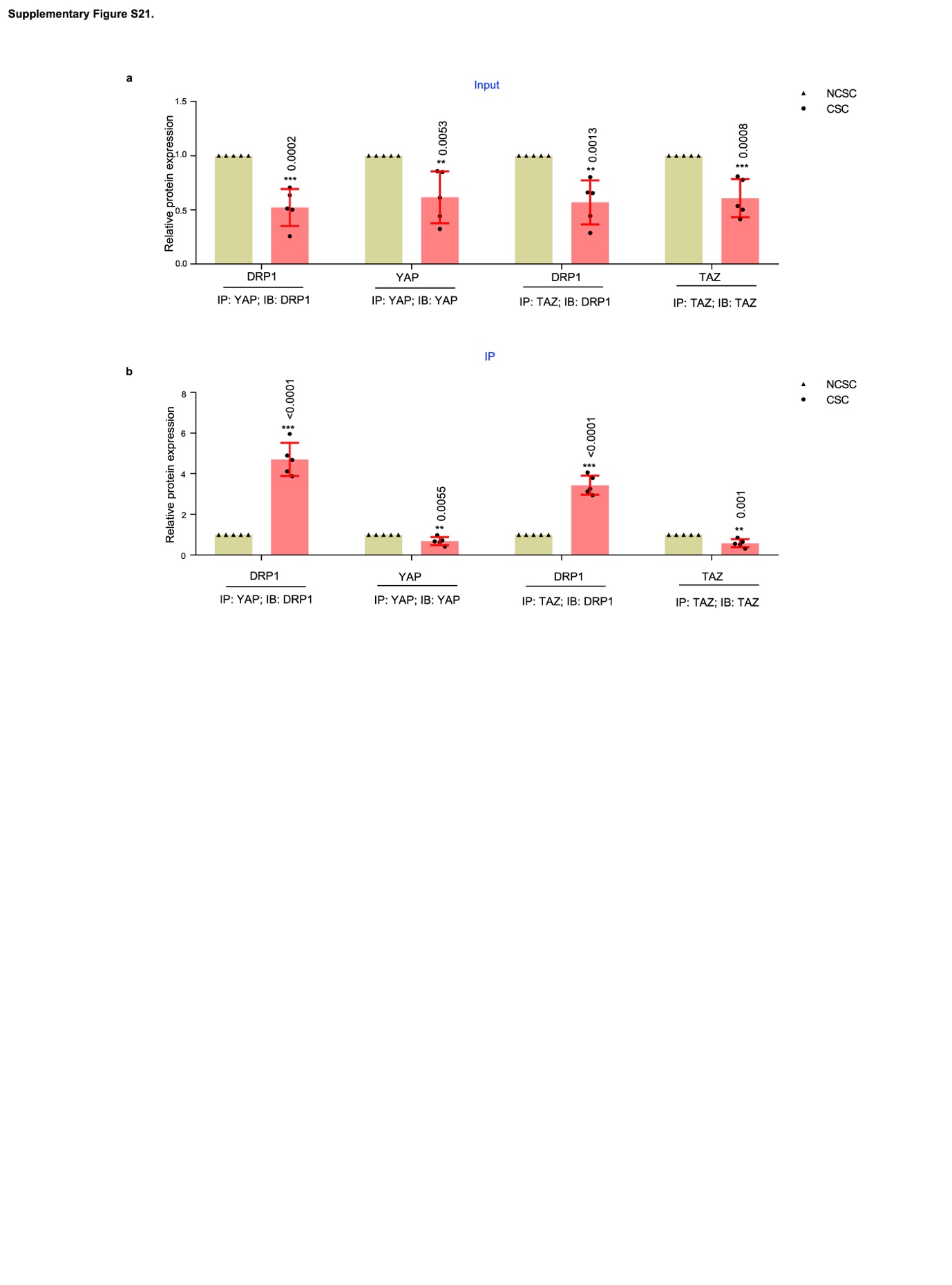
**

**Figure. S21. YAP/TAZ interacts with DRP1 in TNBC patient-derived spheroids a, b** Co-IP analyses using either control IgG or antibodies against YAP and TAZ. Western blot quantification was performed to analyse differential interaction pattern in the cytoplasm of CD44^+^/CD24^-^ cell population (representing CSCs) in comparison with rest of the cell population (representing non-CSCs) (n=5). Cytoplasmic protein expressions were normalized against β-tubulin, which served as the internal loading control. The data are presented as the mean ± standard deviation (SD), with "n" representing the number of biological replicates per experimental group. Significance was assessed using an unpaired Student's t-test, and the associated two-tailed p-value is indicated in the bar plots. Compared to the control group: **p<0.01 and ***p<0.001. NCSC, non-cancer stem cells; CSC, Cancer stem cells; IP, Immunoprecipitate; IB, Immunoblot.


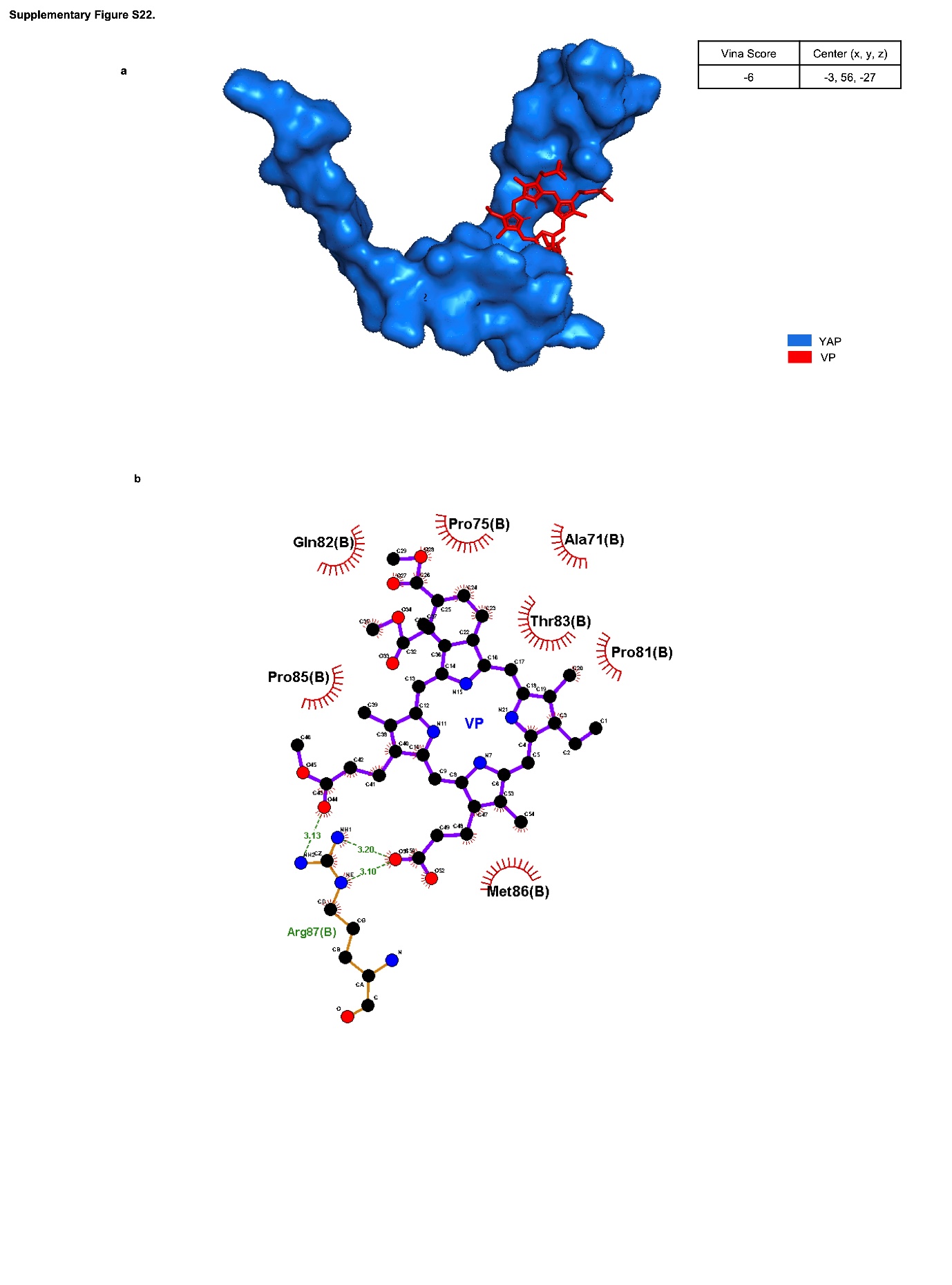


**Figure. S22. In silico docking interaction between YAP and Verteporfin**

**a** Molecular docking model depicting the YAP/VP interaction. **b** LigPlot diagrams depicting the interaction between YAP and verteporfin (VP). Brick red spokes indicate hydrophobic interactions extending towards the ligand atoms. The ligand itself is illustrated in purple, with carbon (C), nitrogen (N), and oxygen (O) atoms represented in black, blue, and red respectively. Hydrogen bond lengths are denoted by green dashed lines.


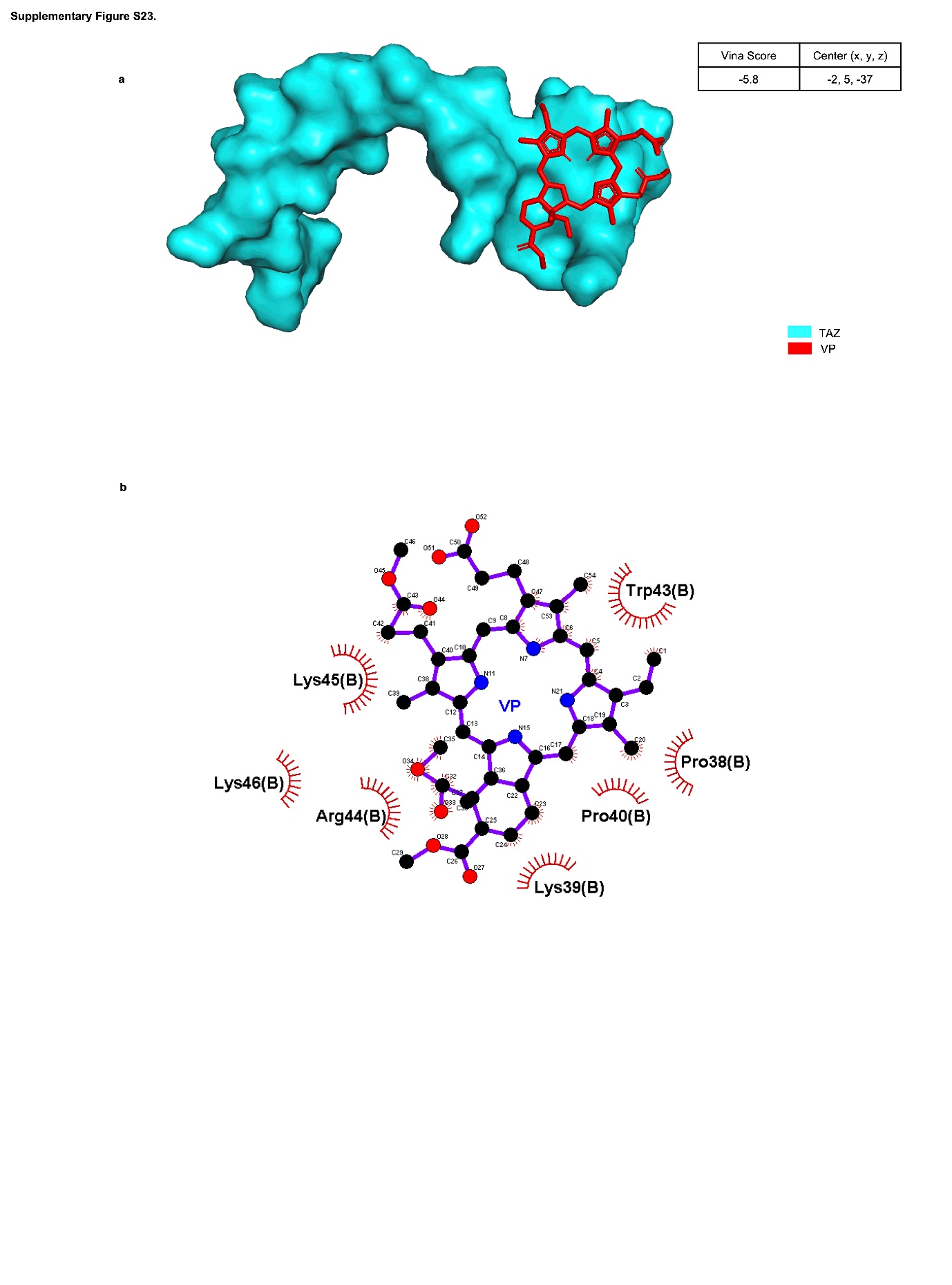


**Figure. S23. In silico docking interaction between TAZ and Verteporfin**

**a** Molecular docking model depicting the TAZ/VP interaction. **b** LigPlot diagrams depicting the interaction between TAZ and VP. Brick red spokes indicate hydrophobic interactions extending towards the ligand atoms. The ligand itself is illustrated in purple, with carbon (C), nitrogen (N), and oxygen (O) atoms represented in black, blue, and red respectively.


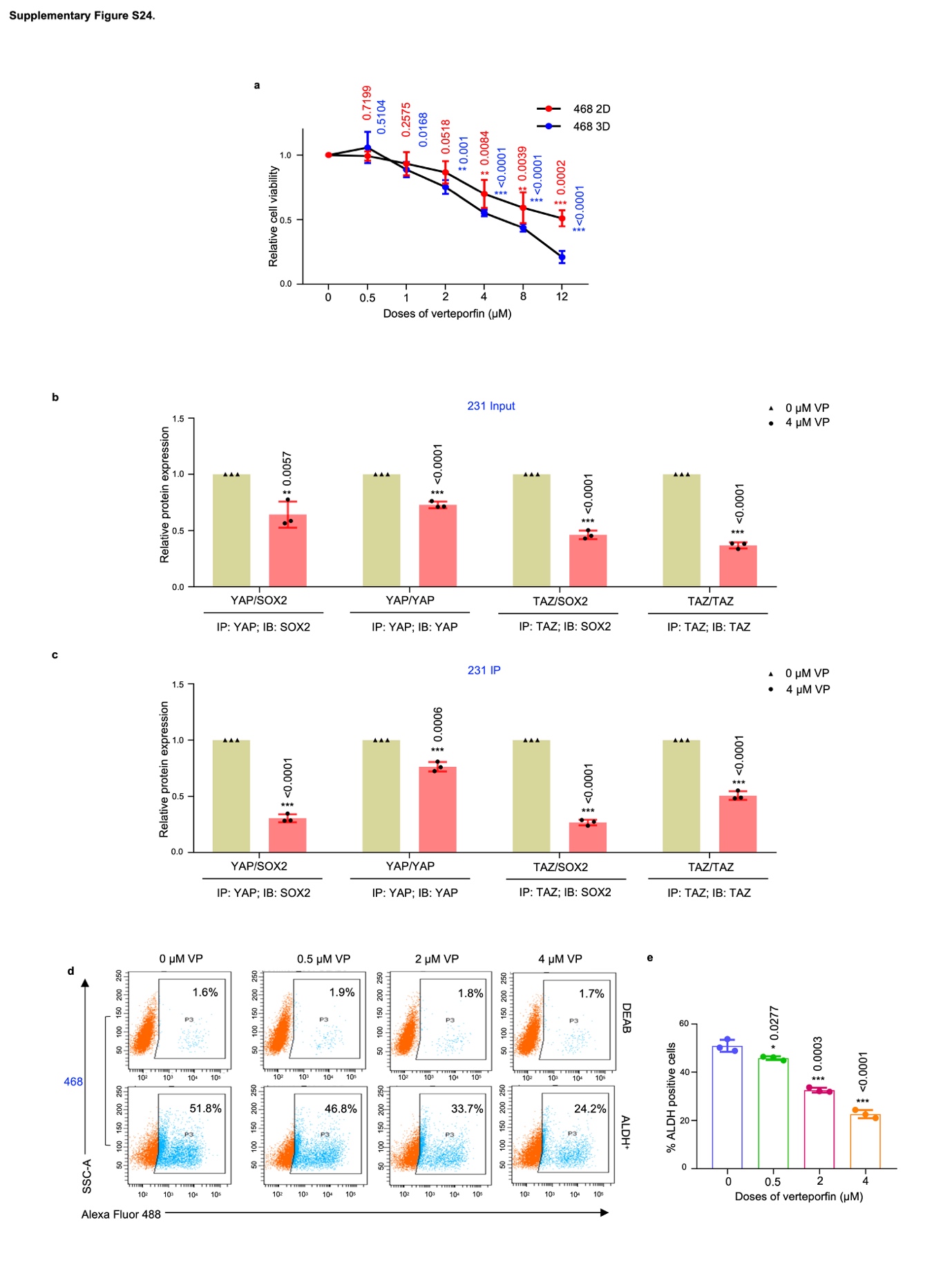


**Figure. S24. Verteporfin treatment impairs YAP/TAZ/SOX2 interaction leading to reduction in ALDH activity**

**a** Cell viability was assessed in adherent and mammosphere cultures of MDA-MB-468 following treatment with different doses of VP (n=3). **b, c** Co-IP of control IgG and anti-YAP/anti-TAZ antibody of 4 µM VP-treated MDA-MB-231 mammospheres, followed by western blot quantification (n=3). **d** Representative plots and **e** quantitative analysis of ALDH activity using ALDEFLUOR assay in MDA-MB-468 mammospheres following 48 hours of treatment with different doses of VP. DEAB, an inhibitor staining control, was used to establish the ALDEFLUOR staining intensity threshold (n=3). Nuclear protein expressions were normalized against H2B, which served as the internal loading control. The data are presented as the mean ± standard deviation (SD), with "n" representing the number of biological replicates per experimental group. Significance was assessed using unpaired Student’s t-test, and the associated two-tailed p-value is indicated in the bar plots. Compared to their respective untreated controls: *p<0.05, **p<0.01, ***p<0.001. 231, MDA-MB-231; 468, MDA-MB-468; ALDH, Aldehyde dehydrogenase; VP, Verteporfin; IP, Immunoprecipitate; IB, Immunoblot.

**
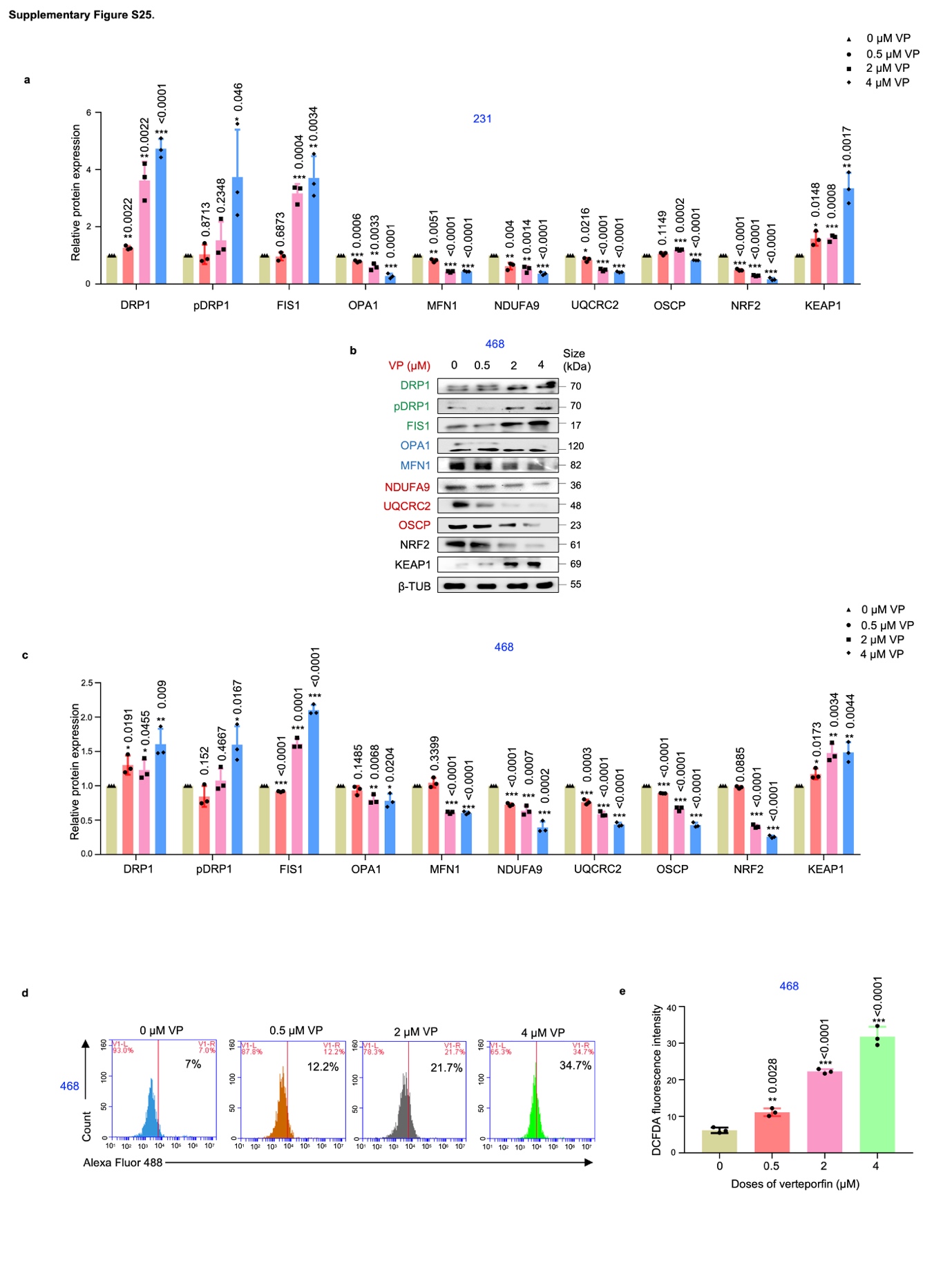
**

**Figure. S25. Pharmacological inhibition of YAP/TAZ causes disruption of mitochondrial homeostasis and alters redox balance**

**a** Mammospheres were subjected to treatment with varying concentrations of VP (0.5, 2, 4 µM) for 48 hours. Subsequently, the expression levels of mitochondrial fission markers (DRP1, pDRP1, FIS1), fusion markers (OPA1, MFN1), ETC complex proteins (NDUFA9, UQCRC2, OSCP), NRF2 and KEAP1 were quantified in MDA-MB-231 mammospheres (n=3). **b, c** Western blot analyses and quantification of the expression levels of mitochondrial fission markers (DRP1, pDRP1, FIS1, indicated in green), fusion markers (OPA1, MFN1, indicated in blue), ETC complex proteins (NDUFA9, UQCRC2, OSCP, indicated in red), NRF2 and KEAP1 in MDA-MB-468 mammospheres (n=3). **d, e** Assessment of alterations in reactive oxygen species (ROS) levels following genetic depletion of *YAP* and *TAZ* in MDA-MB-468 mammospheres using H2DCFDA assay (n=3). All protein expressions were normalized against β-tubulin, which served as the internal loading control. The data are presented as the mean ± standard deviation (SD), with "n" representing the number of biological replicates per experimental group. Significance was assessed using an unpaired Student's t-test, and the associated two-tailed p-value is indicated in the bar plots. Compared to the untreated control group: *p<0.05, **p<0.01 and ***p<0.001. 231, MDA-MB-231; 468, MDA-MB-468; VP, Verteporfin; β-TUB, β-tubulin.

**
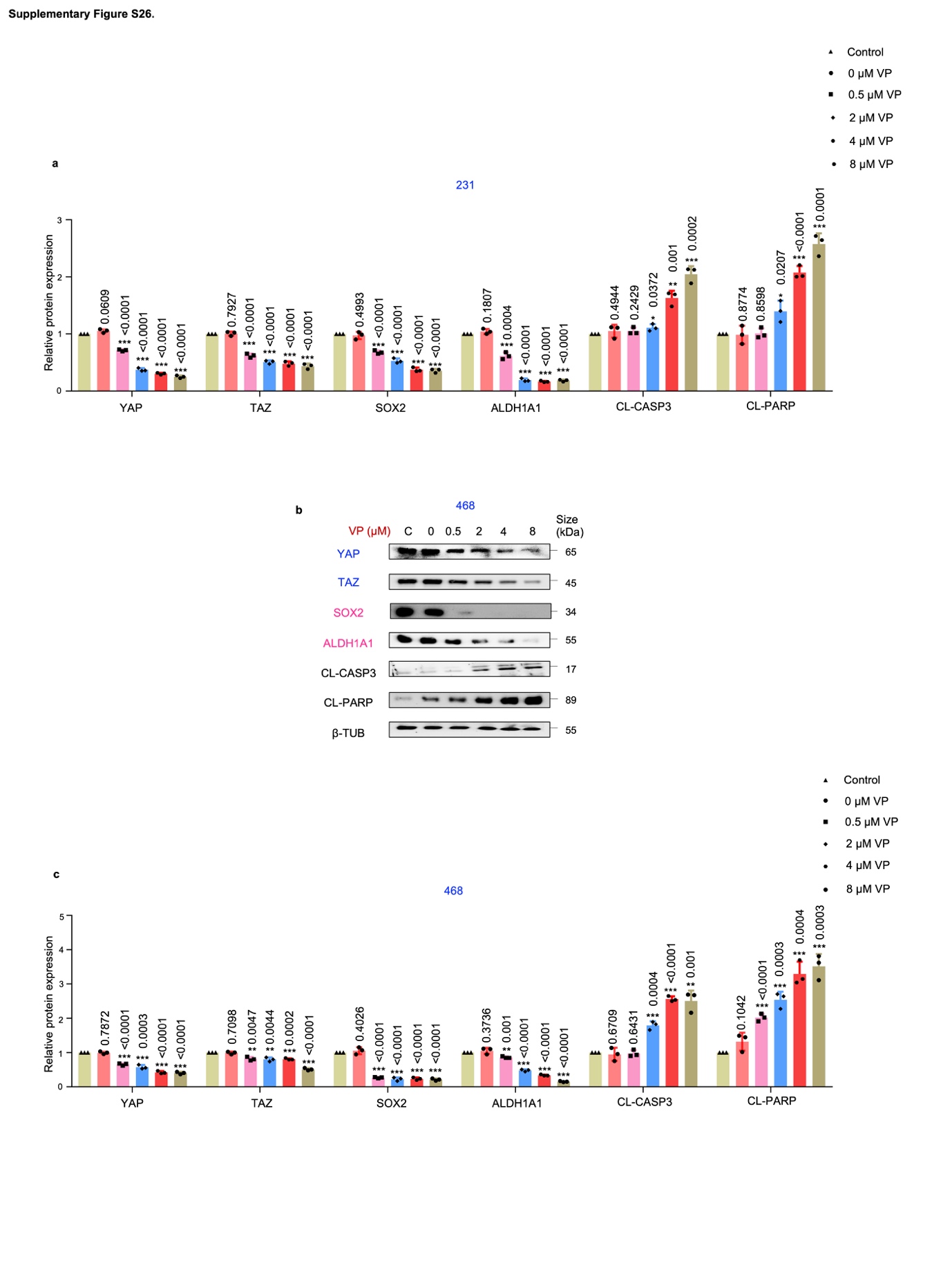
**

**Figure. S26. Verteporfin administration reduces the expression of stemness markers and increases the expression of apoptotic markers in mammospheres**

**a** Western blot quantification of the expression levels of YAP/TAZ, stemness markers (SOX2 and ALDH1A1) and apoptosis markers (cleaved-caspase3 and cleaved-PARP) in VP-treated MDA-MB-231 mammospheres (n=3). **b, c** Western blot analyses and quantification of the expression levels of YAP/TAZ, stemness markers (SOX2 and ALDH1A1) and apoptosis markers (cleaved-caspase3 and cleaved-PARP) in VP-treated MDA-MB-468 mammospheres (n=3). Markers associated with hippo signaling pathway and stemness are marked in blue, and pink respectively. All protein expressions were normalized against β-tubulin, which served as the internal loading control. The data are presented as the mean ± standard deviation (SD), with "n" representing the number of biological replicates per experimental group. Significance was assessed using an unpaired Student's t-test, and the associated two-tailed p-value is indicated in the bar plots. Compared to the untreated control group: *p<0.05, **p<0.01 and ***p<0.001. 231, MDA-MB-231; 468, MDA-MB-468; VP, Verteporfin; β-TUB, β-tubulin.

**
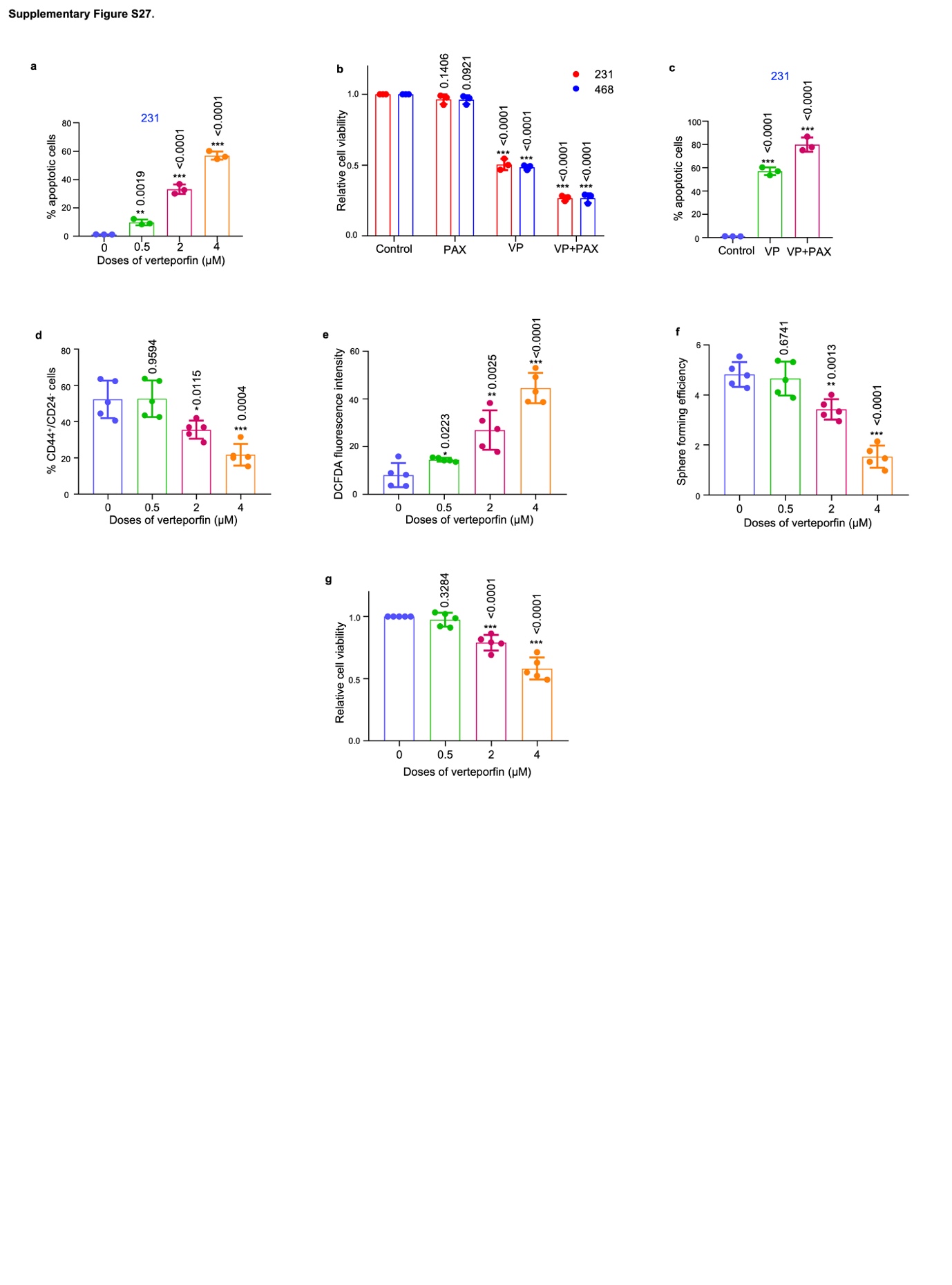
**

**Figure. S27. Verteporfin sensitizes the mammospheres and patient-derived organoids to paclitaxel chemotherapy**

**a** Quantitative analysis of the percentage of apoptotic cells in MDA-MB-231 mammospheres following VP treatment as analyzed by flow-cytometry using Annexin V/PI analysis (n=3). **b** Quantification of cell viability in mammospheres using MTT assay after treatment with either paclitaxel or VP alone, or in combination with paclitaxel and VP (n=3). **c** Quantitative analysis of apoptotic cell percentage in MDA-MB-231 mammospheres following treatment with VP alone and in combination with paclitaxel, assessed by flow cytometry using Annexin V/PI analysis (n=3). **d** Quantitative analysis of the percentage of CD44^+^/CD24^−^ populations in patient-derived spheroids following VP treatment as analyzed by flow-cytometry (n=5). **e** Quantitative analysis of changes in ROS level following VP treatment in patient-derived spheroids using H2DCFDA assay (n=5). **f** Graphical representation of sphere forming efficiency in patient-derived spheroids post VP treatment (n=5). **g** Assessment of relative cell viability using MTT assay in patient-derived spheroids after treatment with VP (n=5). The data are presented as the mean ± standard deviation (SD), with "n" representing the number of biological replicates per experimental group. Significance was assessed using an unpaired Student's t-test, and the associated two-tailed p-value is indicated in the bar plots. Compared to the untreated control group: *p<0.05, **p<0.01 and ***p<0.001. 231, MDA-MB-231; 468, MDA-MB-468; VP, Verteporfin; PAX, Paclitaxel.

**
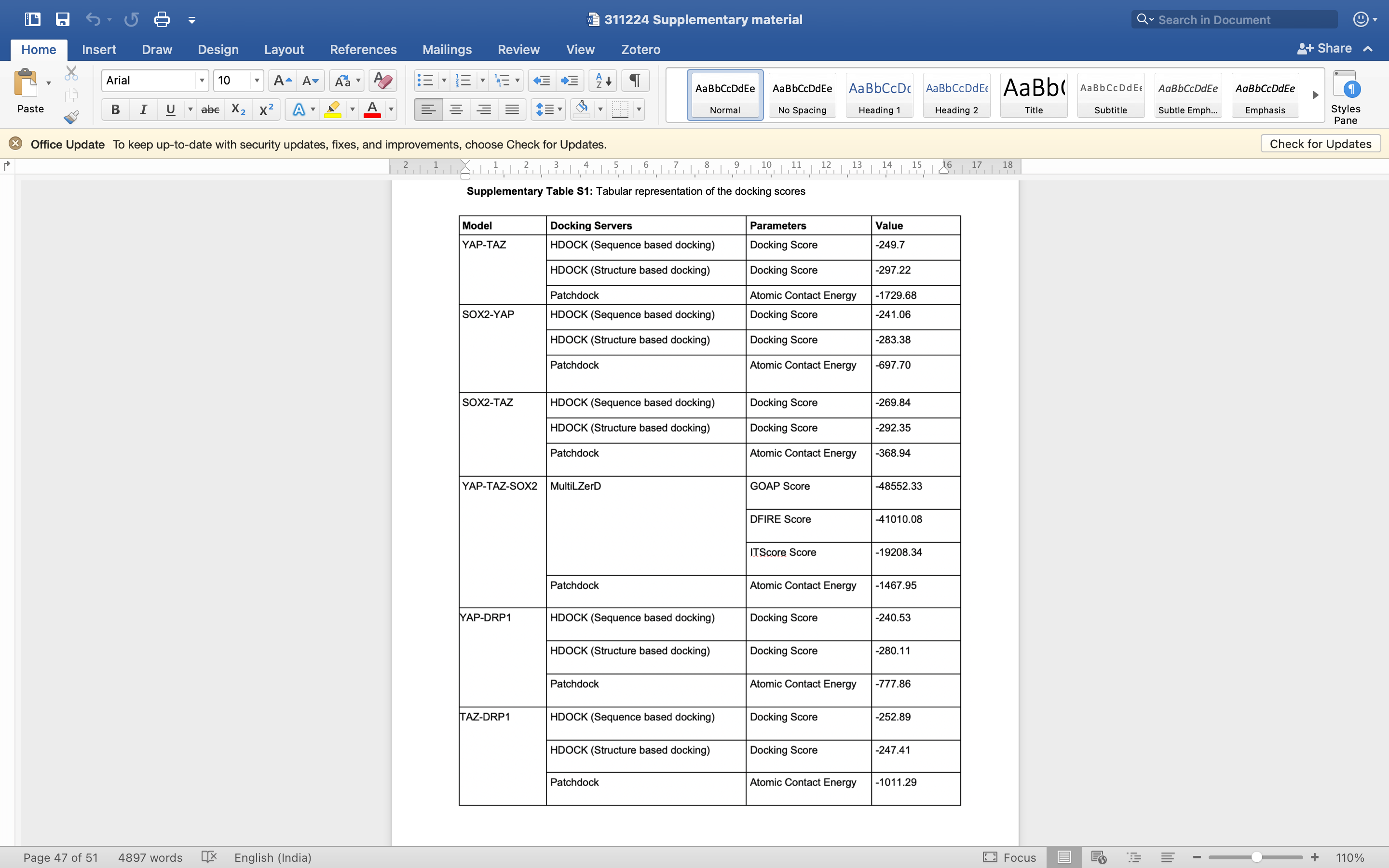
**

**Table S1.** Tabular representation of the docking scores.


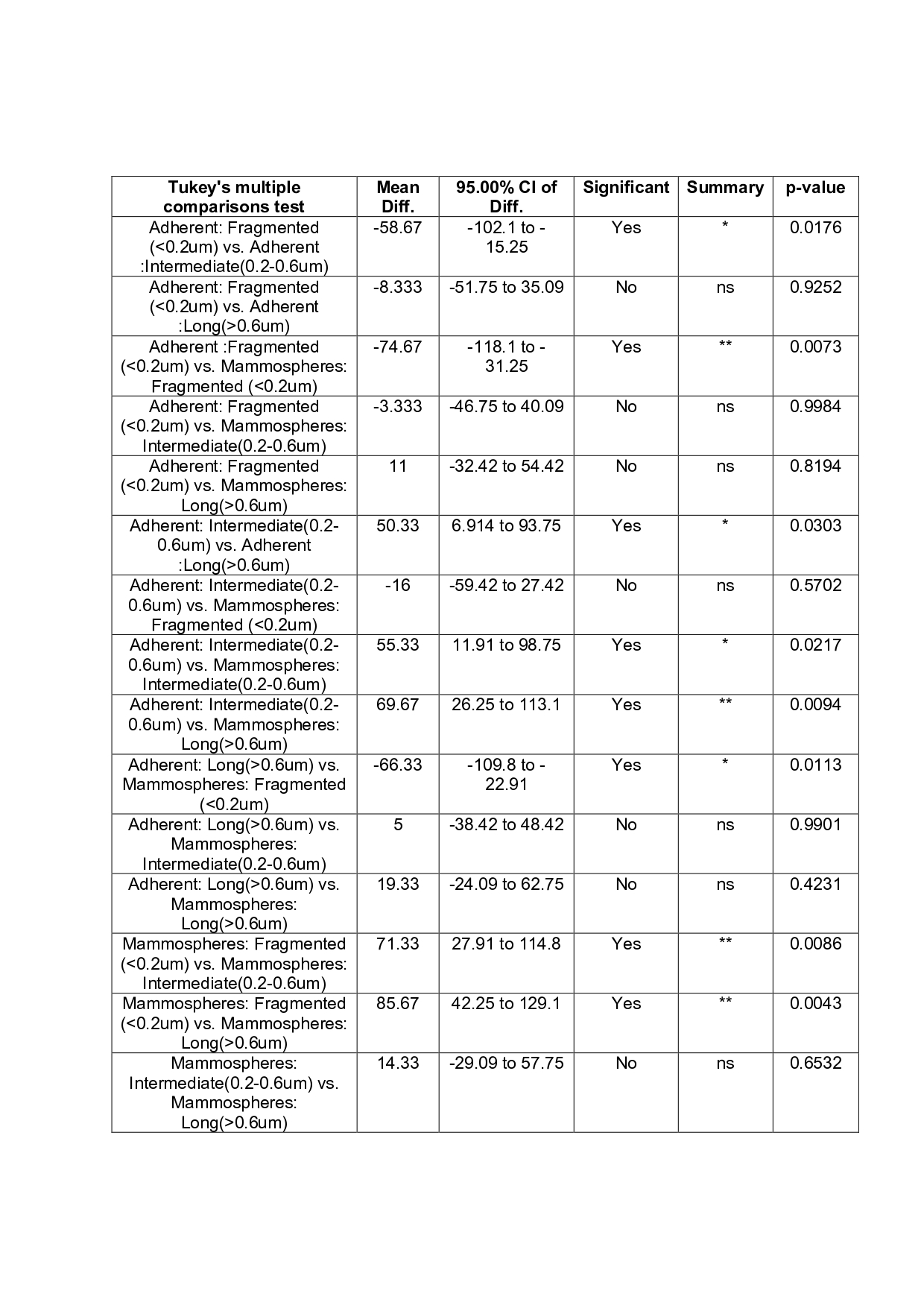


**Table S2.** Comparison of mitochondrial morphology between adherent MDA-MB-231 cells and MDA-MB-231 mammospheres.

**
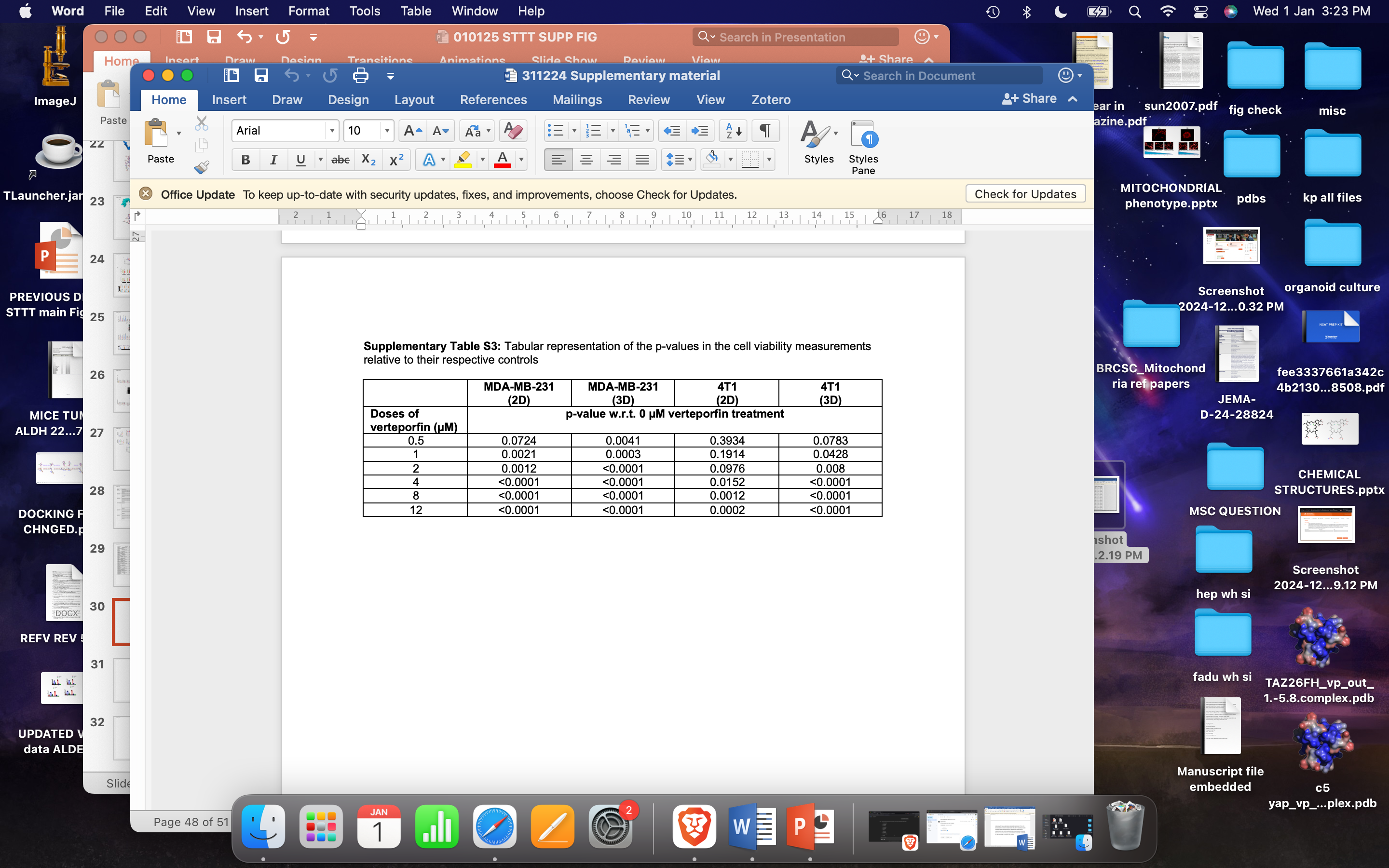
 Table S3.** Tabular representation of the p-values in the cell viability measurements relative to their respective controls.

**
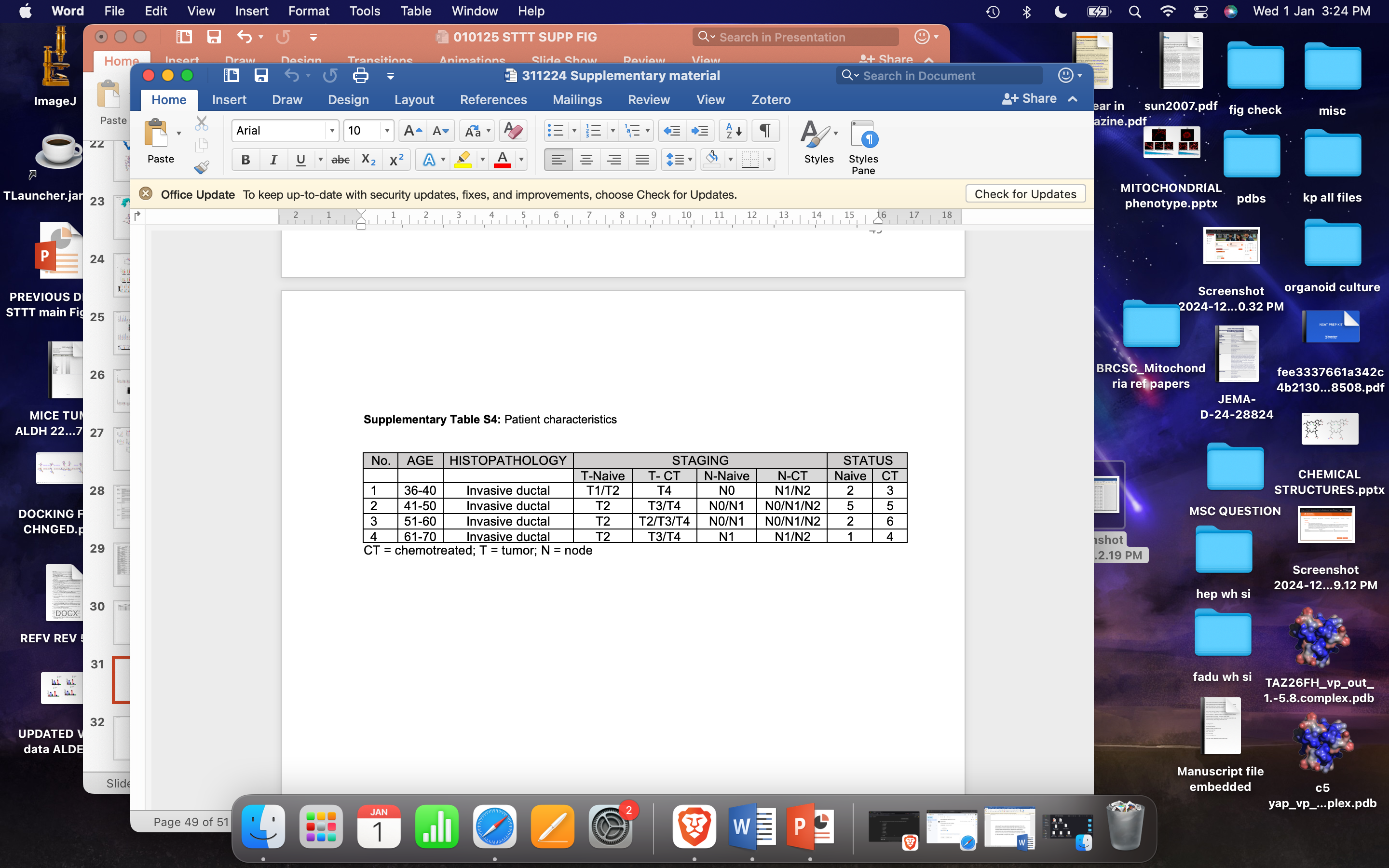
**

**Table S4.** Patient characteristics.

CT = chemotreated; T = tumor; N = node

**
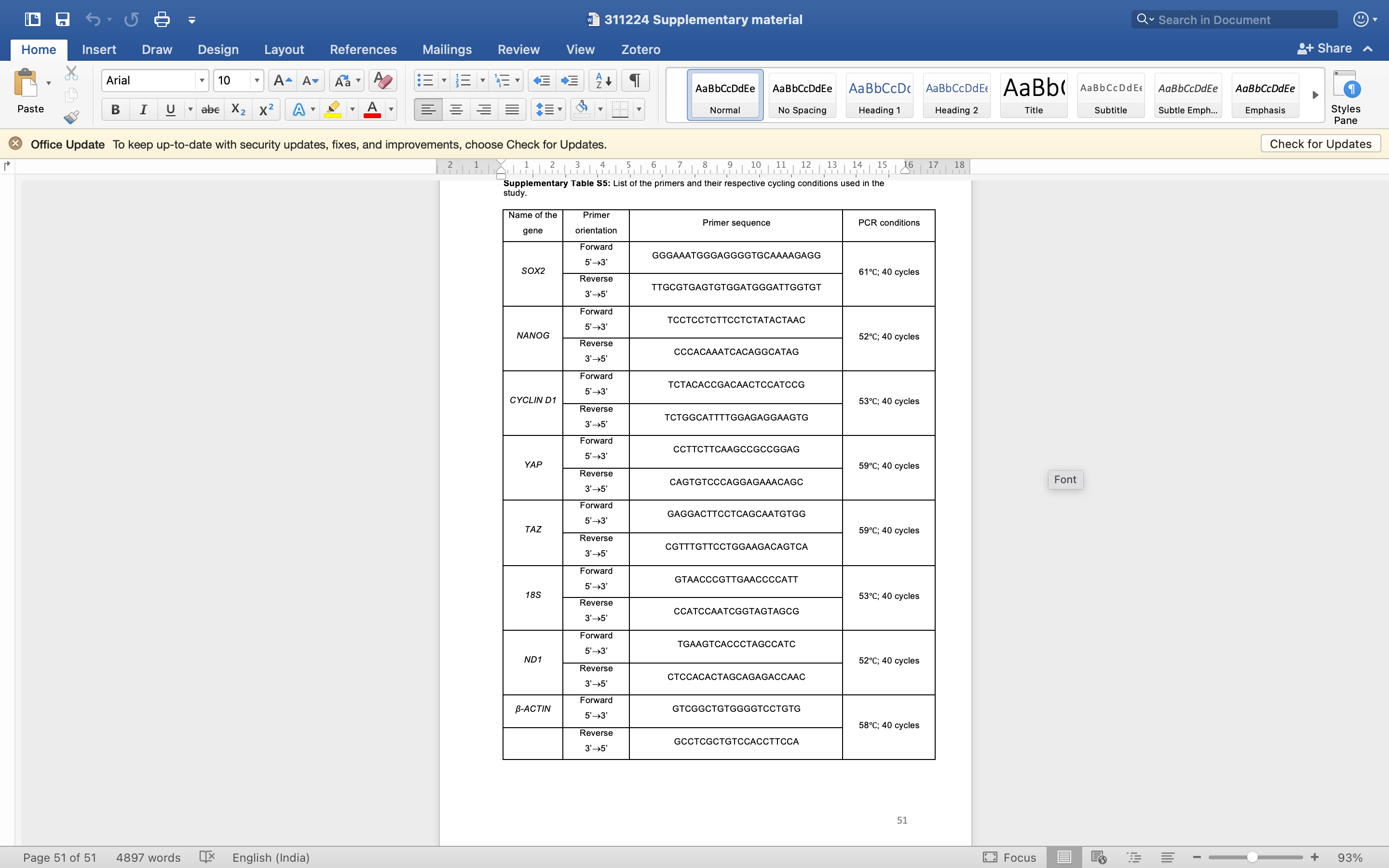
**

**Table S5.** List of the primers and their respective cycling conditions used in the study.
