# Supplementary material for: Precise targeting of transcriptional co-activators YAP/TAZ annihilates chemoresistant brCSCs by alteration of their mitochondrial homeostasis
Source: Signal Transduct Target Ther. 2025 Feb 21;10:61. doi: 10.1038/s41392-025-02133-x (PMC11842803; doi:10.1038/s41392-025-02133-x)

Gating strategy for flow cytometry

Figure 1-Figure 8

Figure 1e.

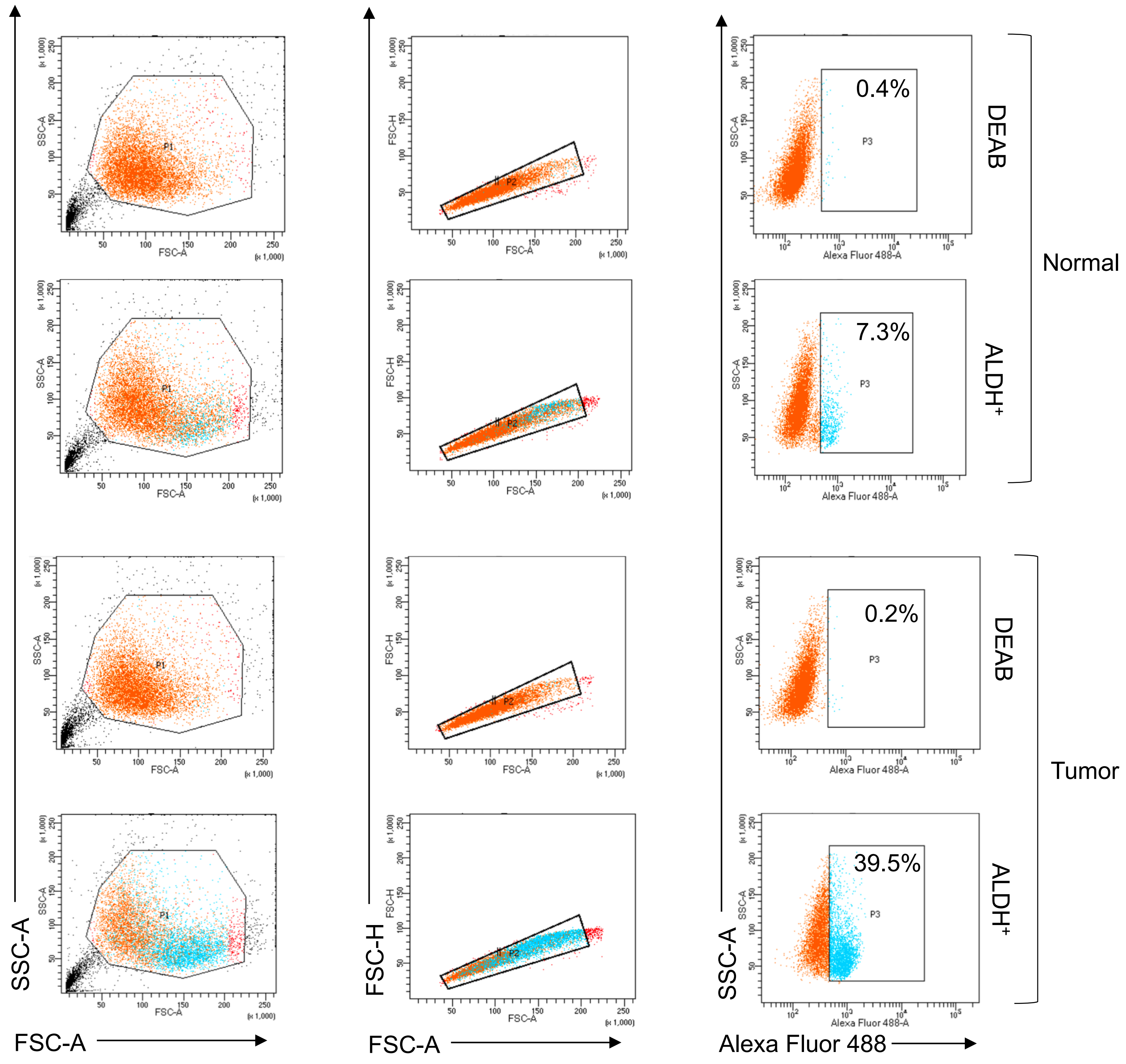
0.4%
P3
DEAB
7.3%
ALDH+
Normal
0.2%
DEAB
39.5%
ALDH+
Tumor
P1
P2
SSC-A
FSC-A
FSC-H
Alexa Fluor 488-A
SSC-A
FSC-A
FSC-H
FSC-A
SSC-A
Alexa Fluor 488

Figure 1k.

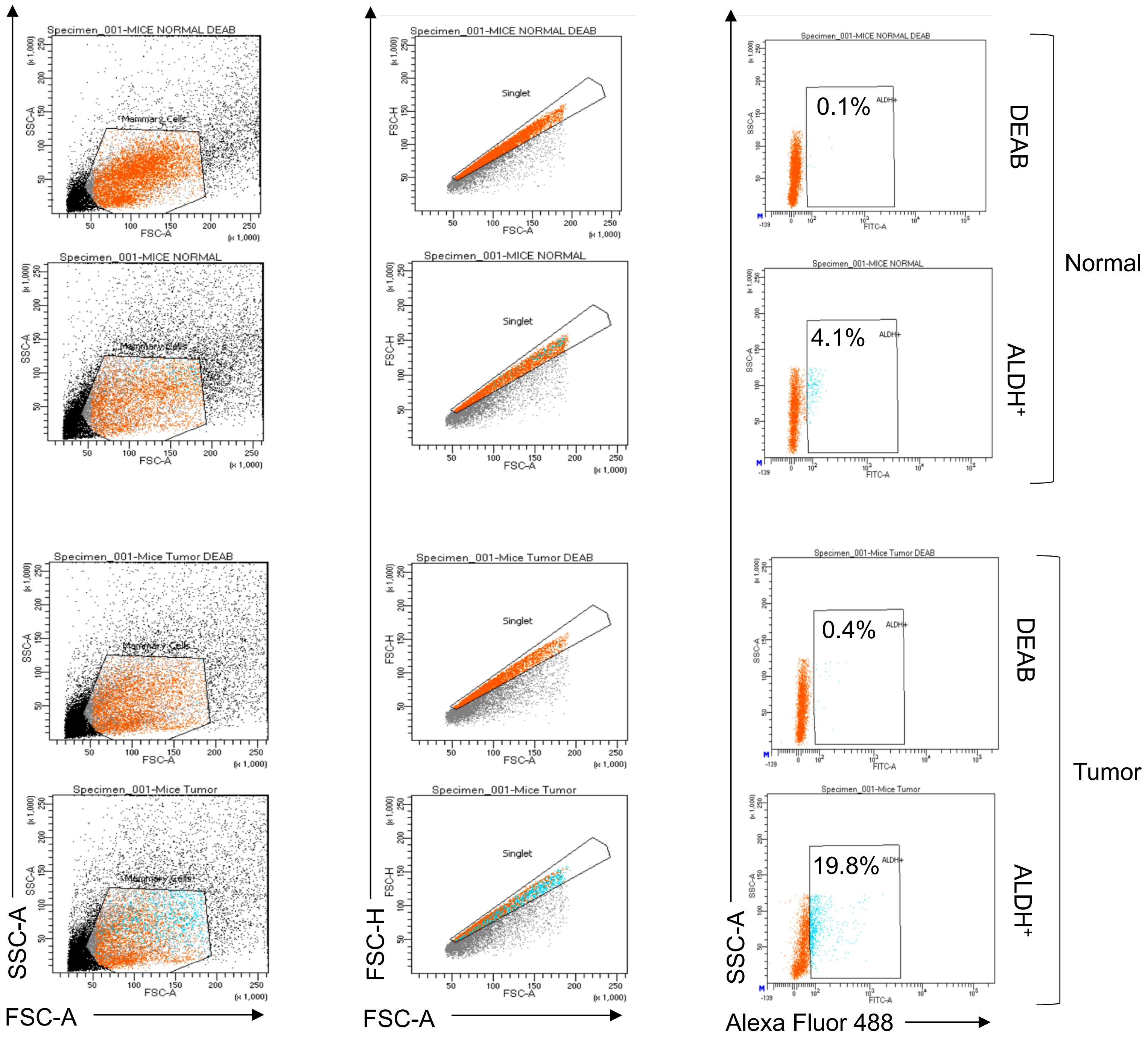
Specimen_001-MICE NORMAL DEAB
Specimen_001-MICE NORMAL
Specimen_001-Mice Tumor DEAB
Specimen_001-Mice Tumor
Mammary Cells
Singlet
ALDH+
0.1%
4.1%
0.4%
19.8%
SSC-A
FSC-A
FSC-H
FITC-A
Alexa Fluor 488
DEAB
ALDH⁺
Normal
Tumor

Figure 2d.

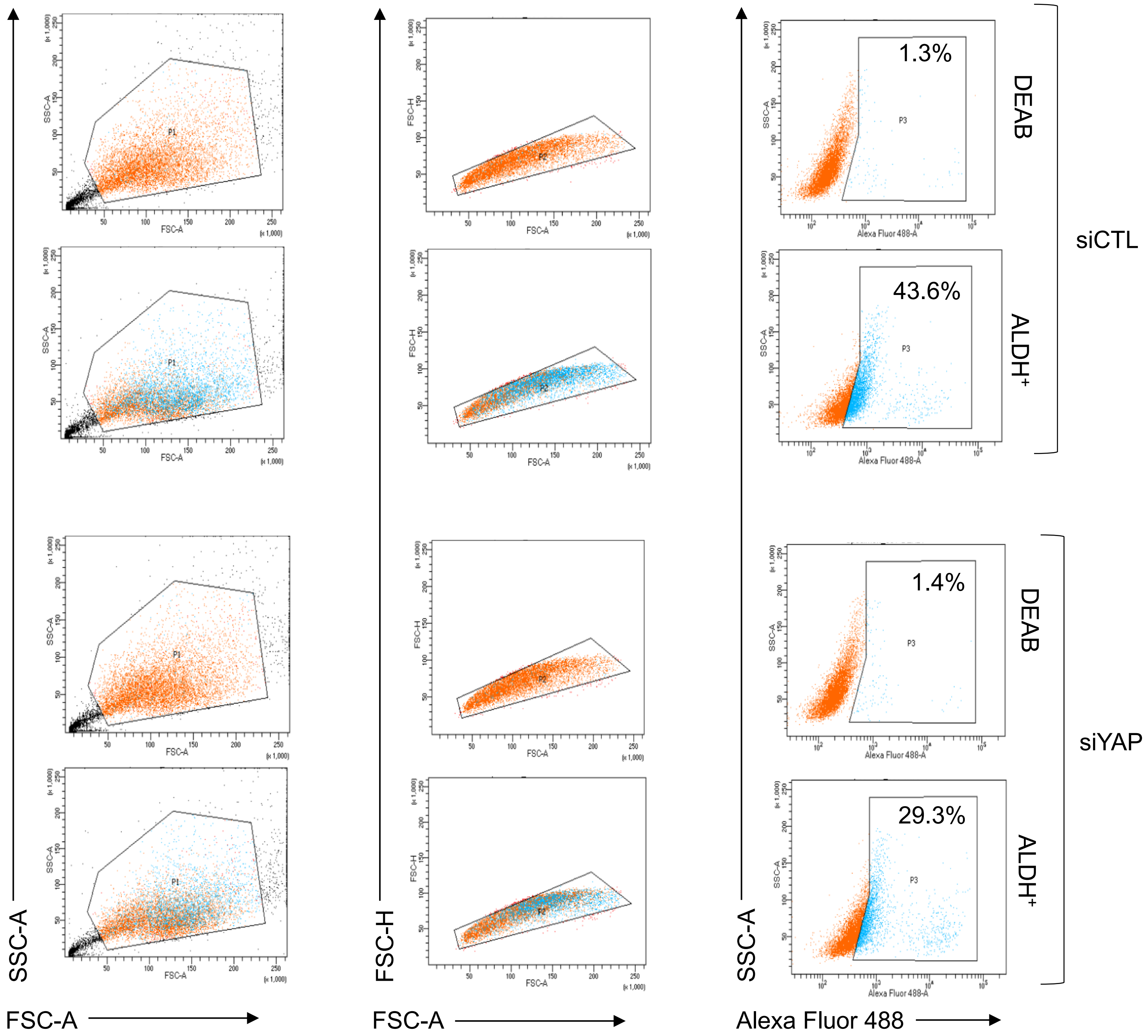
1.3%
DEAB
43.6%
ALDH+
siCTL
1.4%
DEAB
29.3%
ALDH+
siYAP
SSC-A
FSC-A
FSC-H
FSC-A
SSC-A
Alexa Fluor 488

Figure 2d.

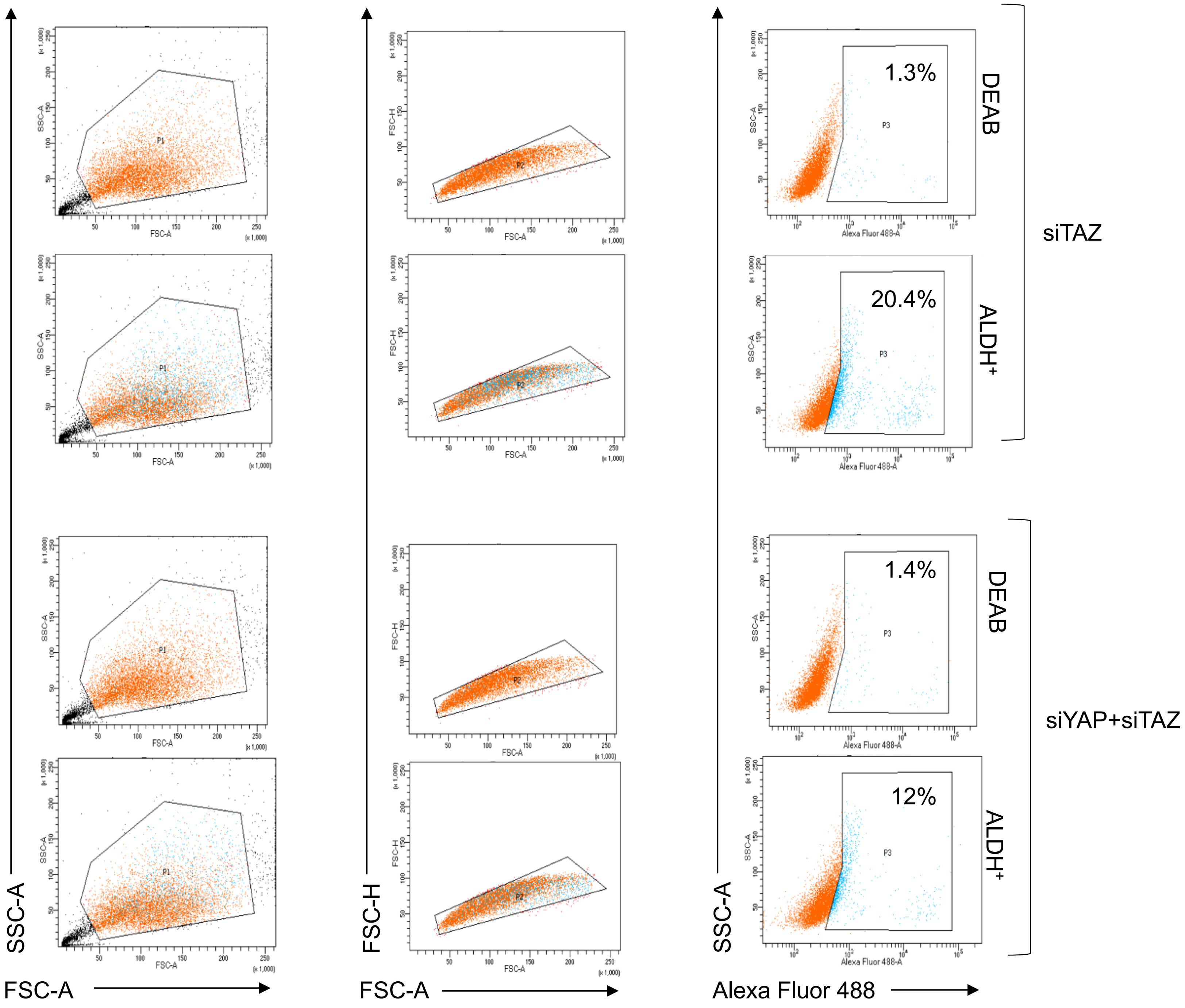

SSC-A
FSC-A
FSC-H
FSC-A
SSC-A
Alexa Fluor 488
1.3%
DEAB
20.4%
ALDH+
siTAZ
1.4%
DEAB
12%
ALDH+
siYAP+siTAZ

Figure 2I.

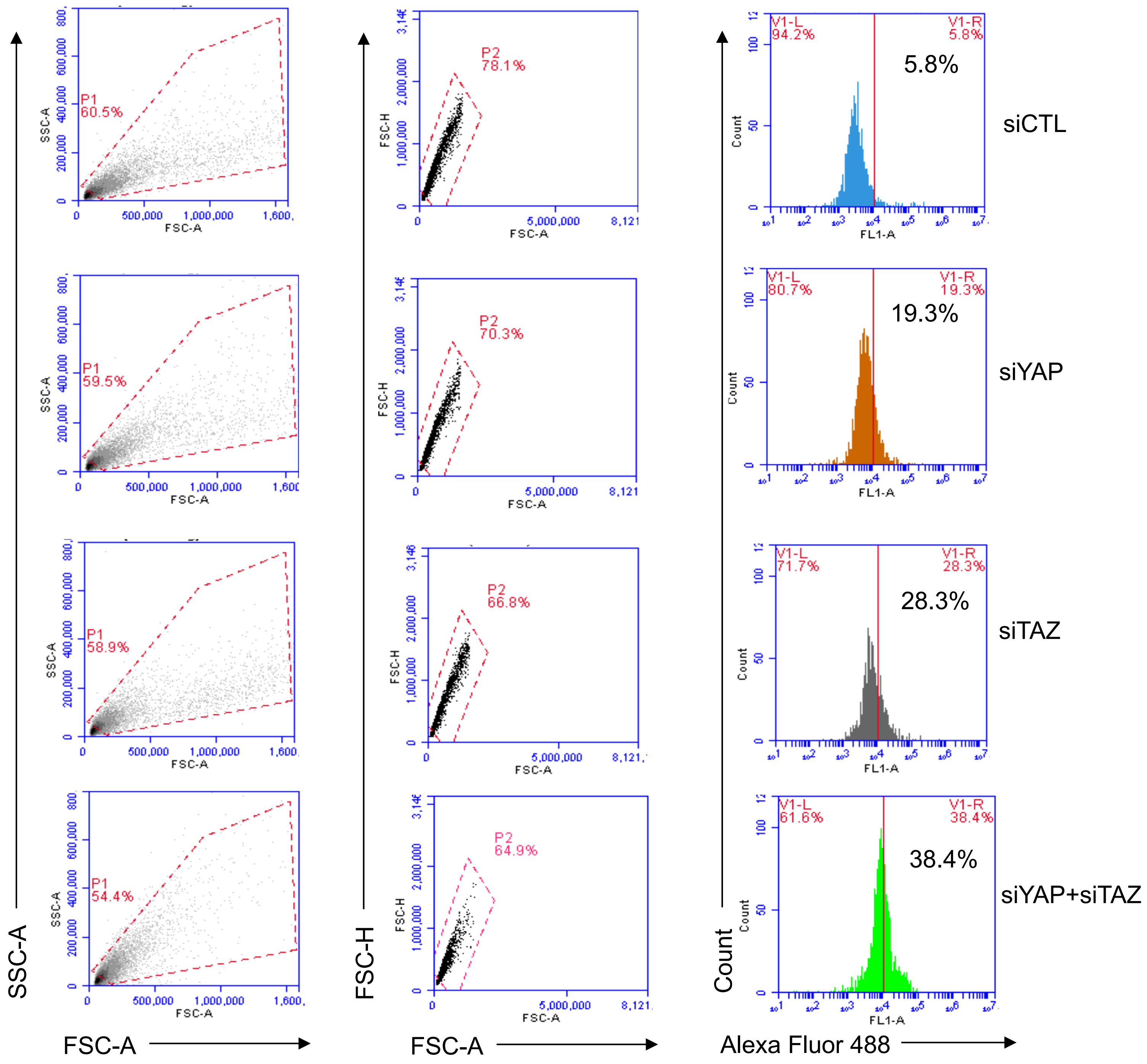
SSC-A
FSC-A
FSC-H
FSC-A
Count
Alexa Fluor 488
P1
60.5%
P2
78.1%
V1-L
94.2%
V1-R
5.8%
5.8%
siCTL
P1
59.5%
P2
70.3%
V1-L
80.7%
V1-R
19.3%
19.3%
siYAP
P1
58.9%
P2
66.8%
V1-L
71.7%
V1-R
28.3%
28.3%
siTAZ
P1
54.4%
P2
64.9%
V1-L
61.6%
V1-R
38.4%
38.4%
siYAP+siTAZ

Figure 4o.

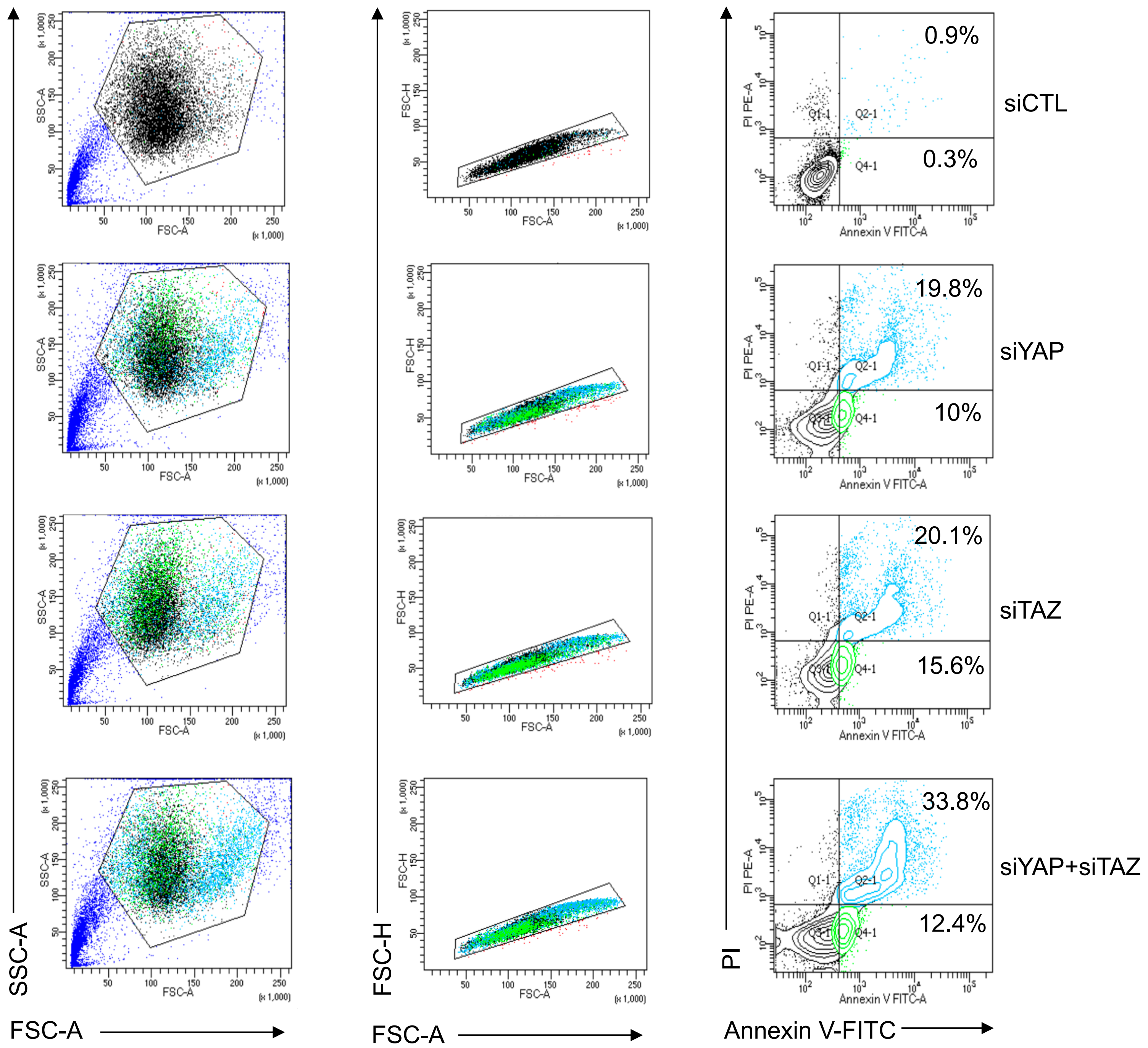
SSC-A
FSC-A
FSC-H
FSC-A
PI
Annexin V-FITC
0.9%
0.3%
siCTL
19.8%
10%
siYAP
20.1%
15.6%
siTAZ
33.8%
12.4%
siYAP+siTAZ

Figure 5g.

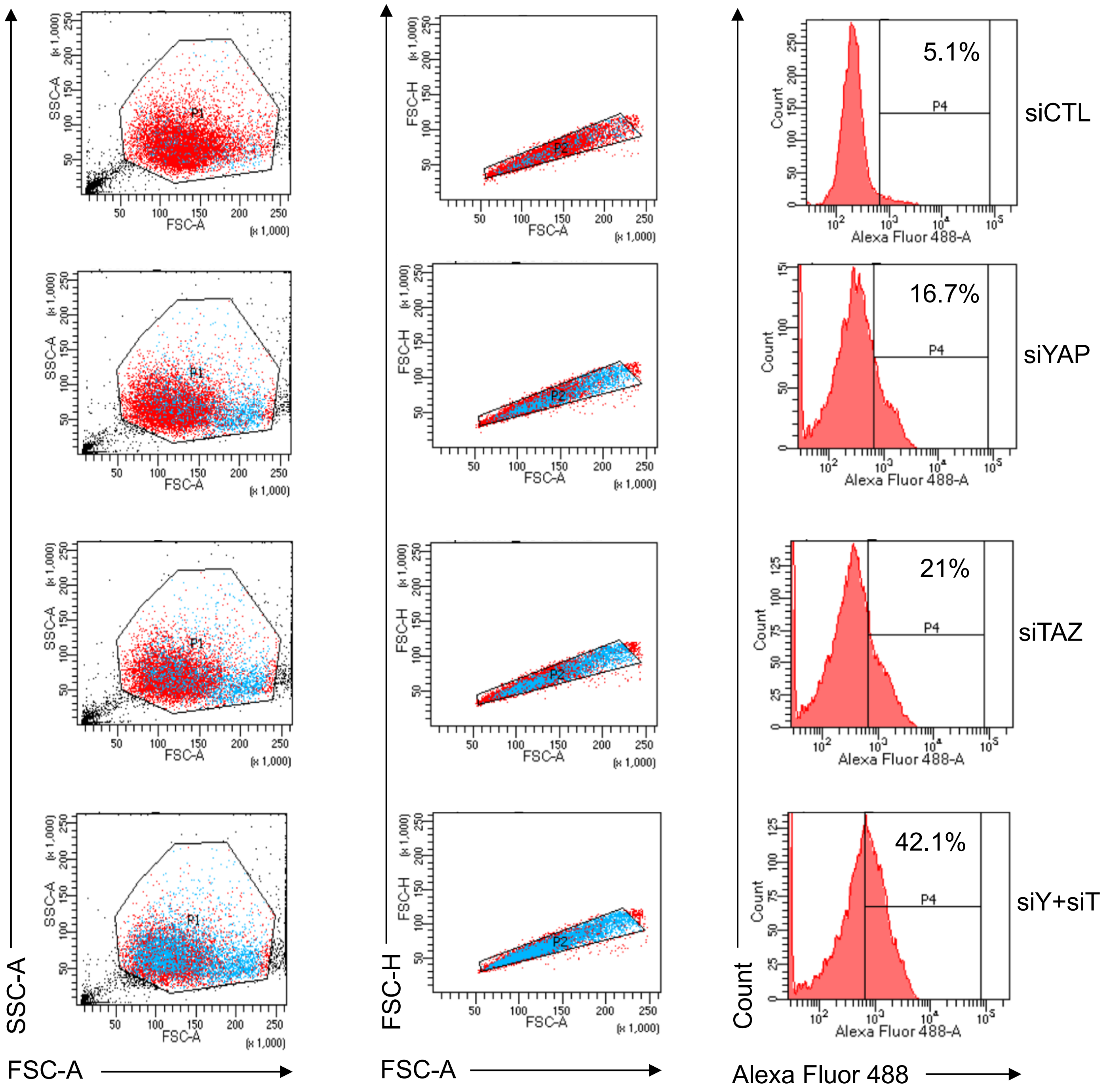
SSC-A
FSC-A
P1
FSC-H
P2
Count
Alexa Fluor 488-A
Alexa Fluor 488
P4
5.1%
siCTL
16.7%
siYAP
21%
siTAZ
42.1%
siY+siT

Figure 6e.

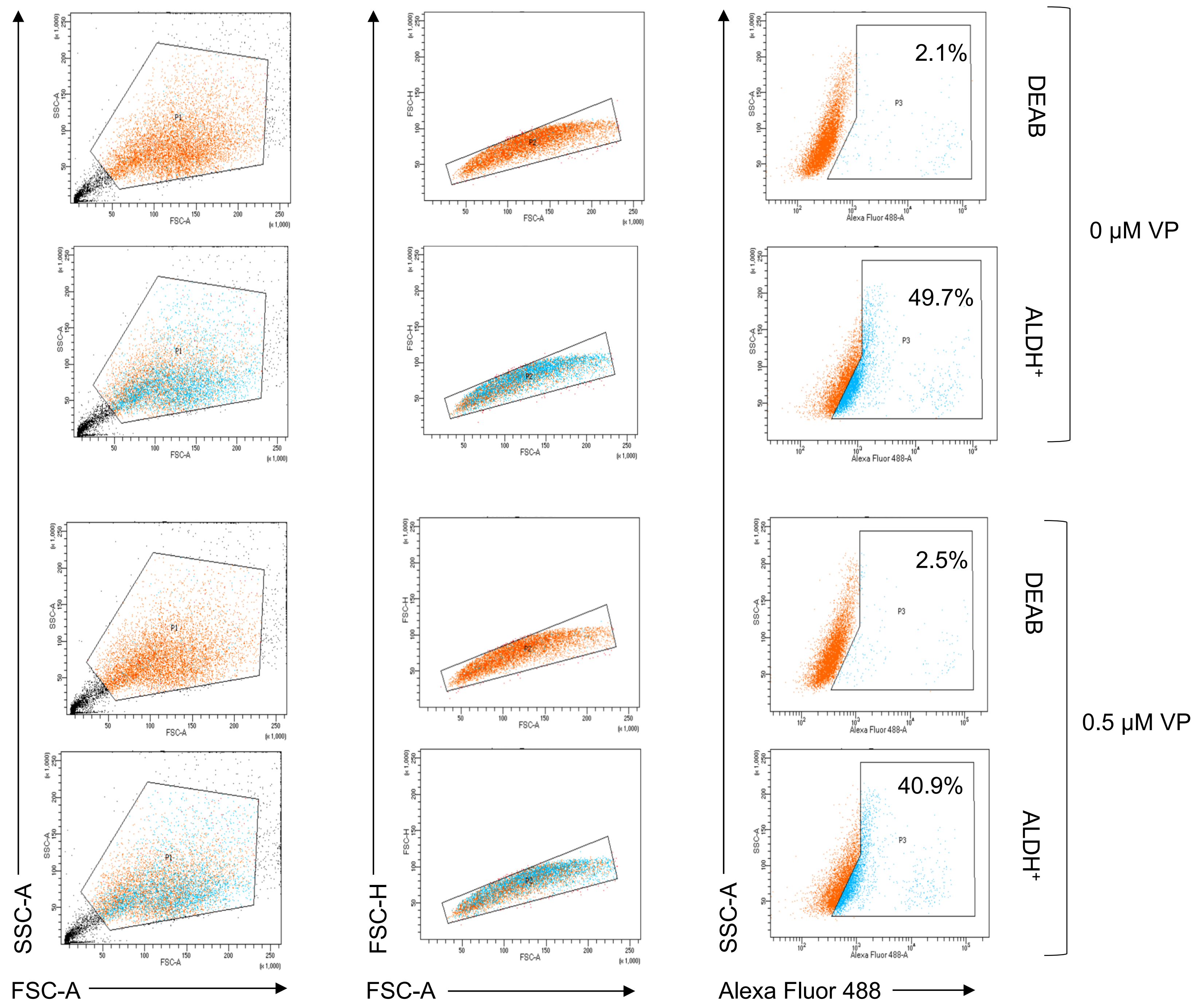
2.1%
DEAB
49.7%
ALDH+
0 μM VP
2.5%
DEAB
40.9%
ALDH+
0.5 μM VP
SSC-A
FSC-A
FSC-H
FSC-A
SSC-A
Alexa Fluor 488

Figure 6e.

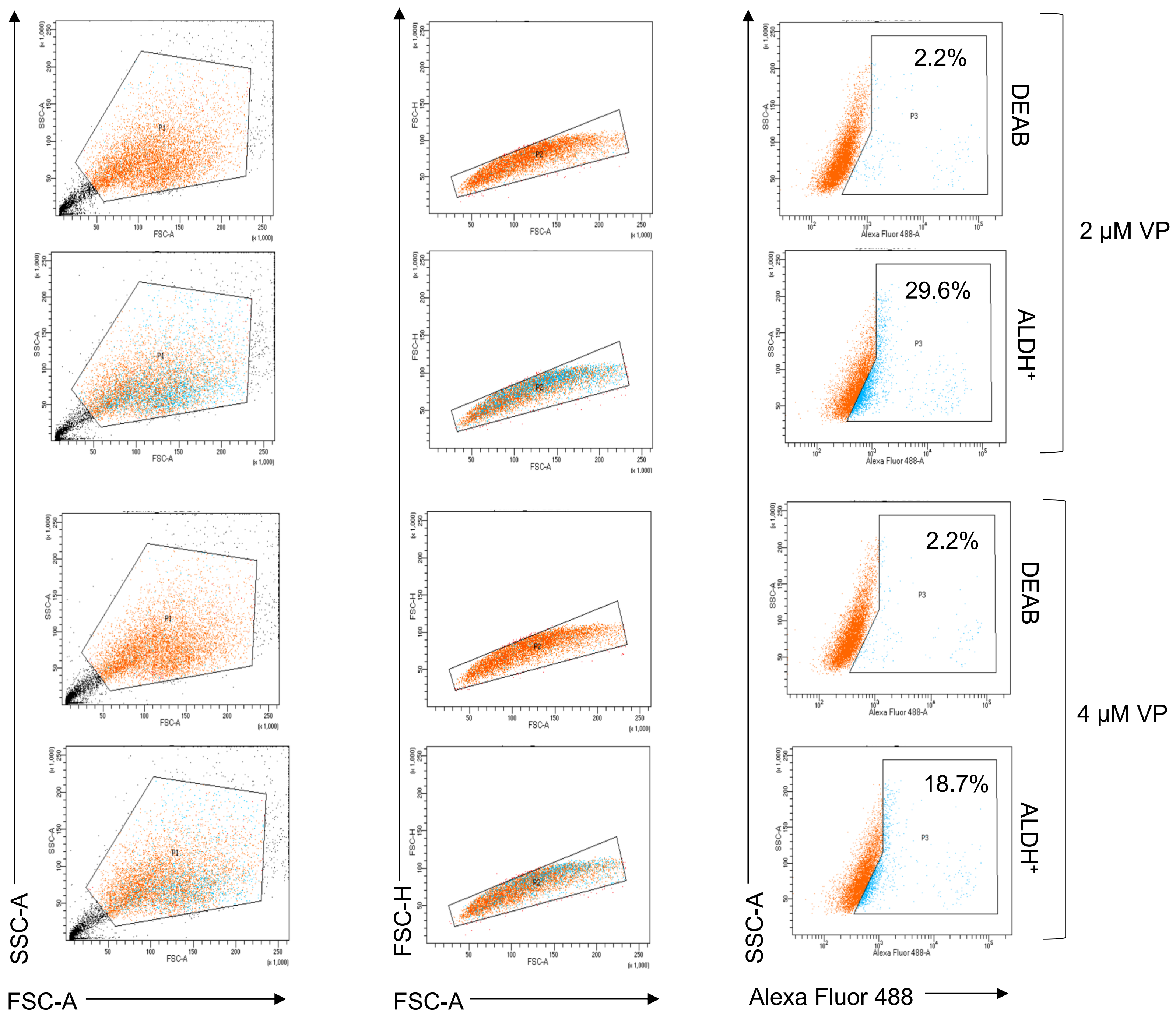
2.2%
DEAB
29.6%
ALDH+
2 μM VP
2.2%
DEAB
18.7%
ALDH+
4 μM VP
SSC-A
FSC-A
FSC-H
FSC-A
SSC-A
Alexa Fluor 488

Figure 7i.

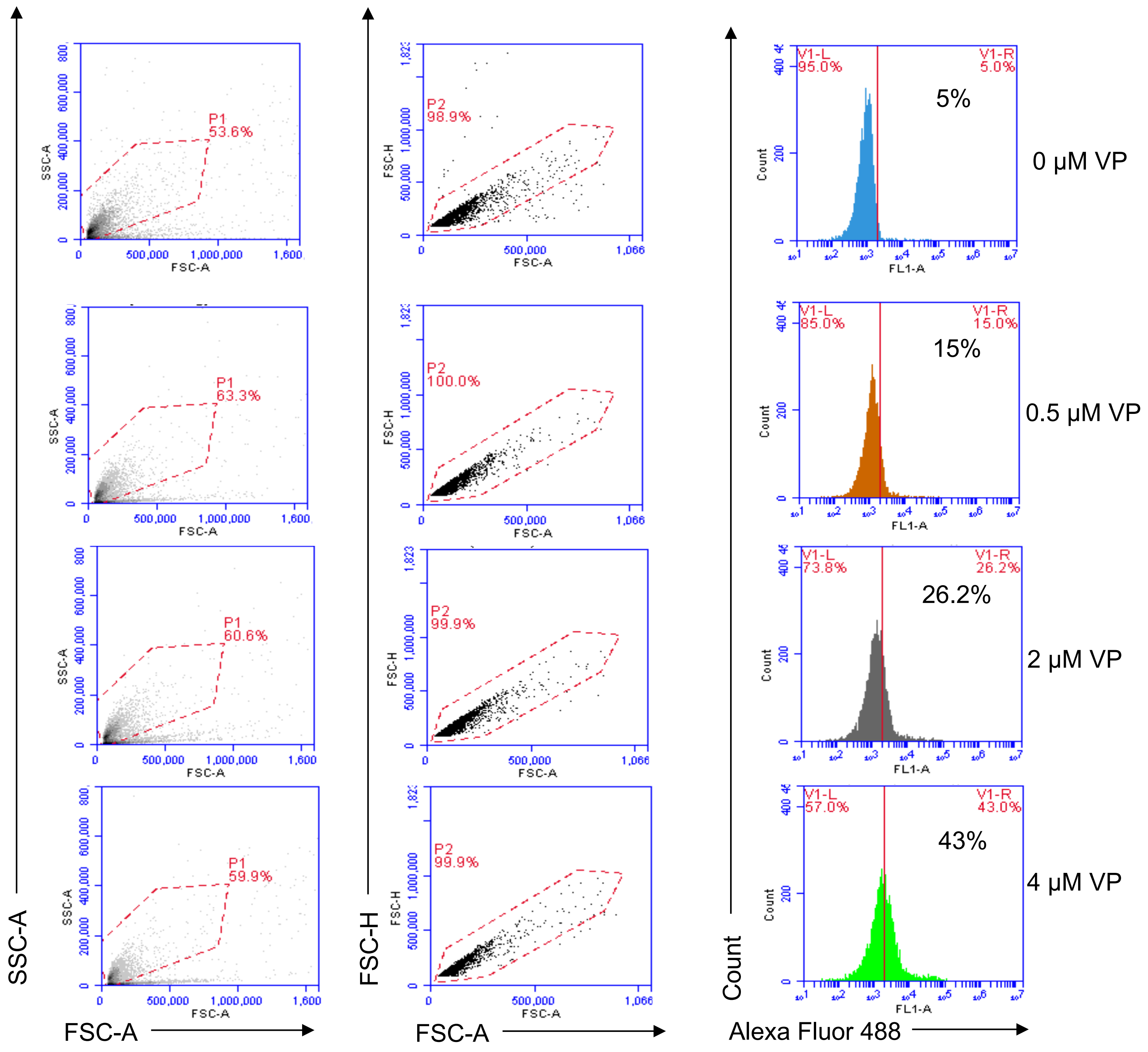
P1
53.6%
P2
98.9%
V1-L
95.0%
V1-R
5.0%
5%
0 µM VP
P1
63.3%
P2
100.0%
V1-L
85.0%
V1-R
15.0%
15%
0.5 µM VP
P1
60.6%
P2
99.9%
V1-L
73.8%
V1-R
26.2%
26.2%
2 µM VP
P1
59.9%
P2
99.9%
V1-L
57.0%
V1-R
43.0%
43%
4 µM VP
SSC-A
FSC-A
FSC-H
FSC-A
Count
Alexa Fluor 488
FL1-A

Figure 8d.

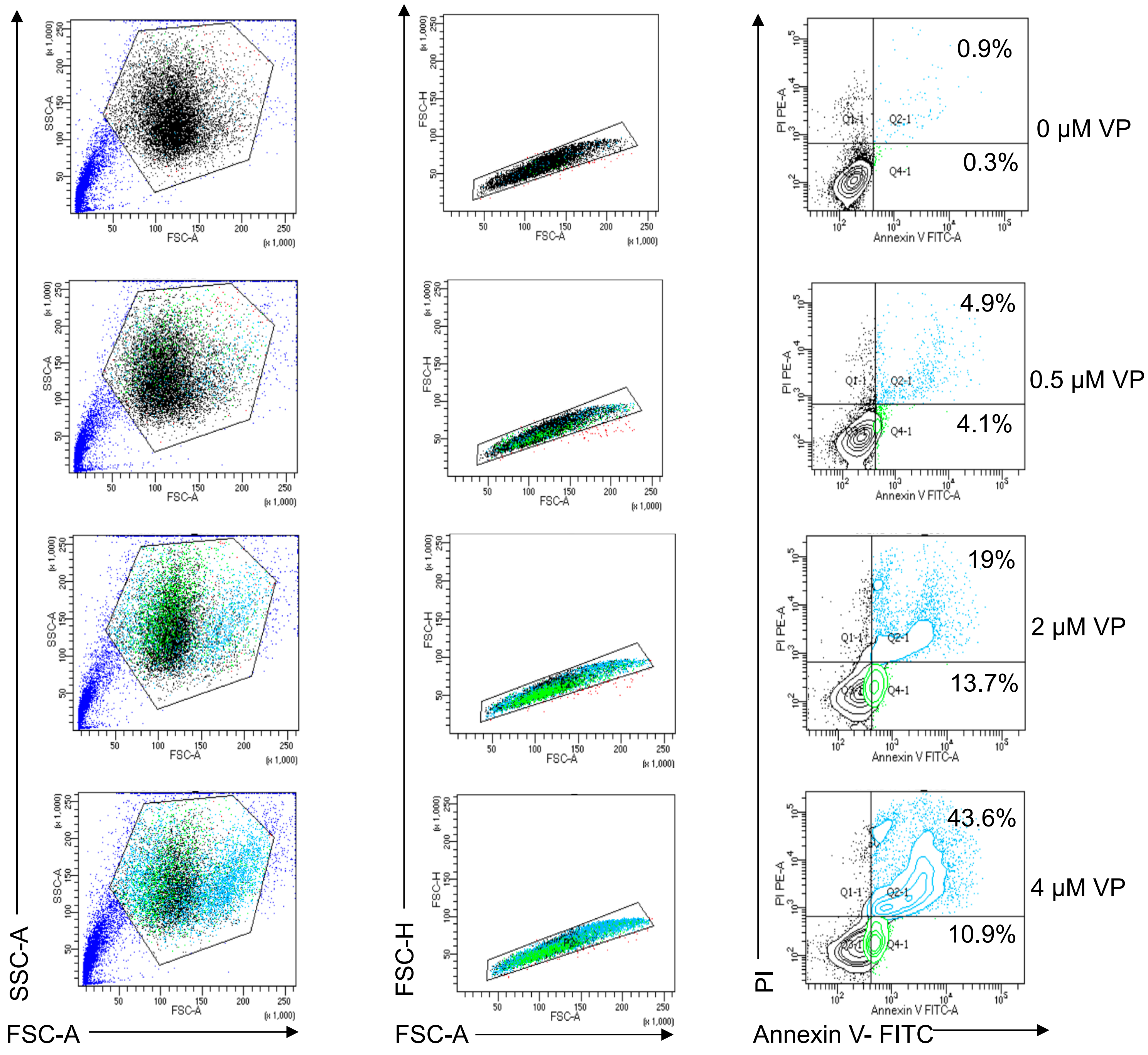

0.9%
0.3%
0 µM VP
4.9%
4.1%
0.5 µM VP
19%
13.7%
2 µM VP
43.6%
10.9%
4 µM VP
SSC-A
FSC-A
FSC-H
FSC-A
PI
Annexin V- FITC

Figure 8e.

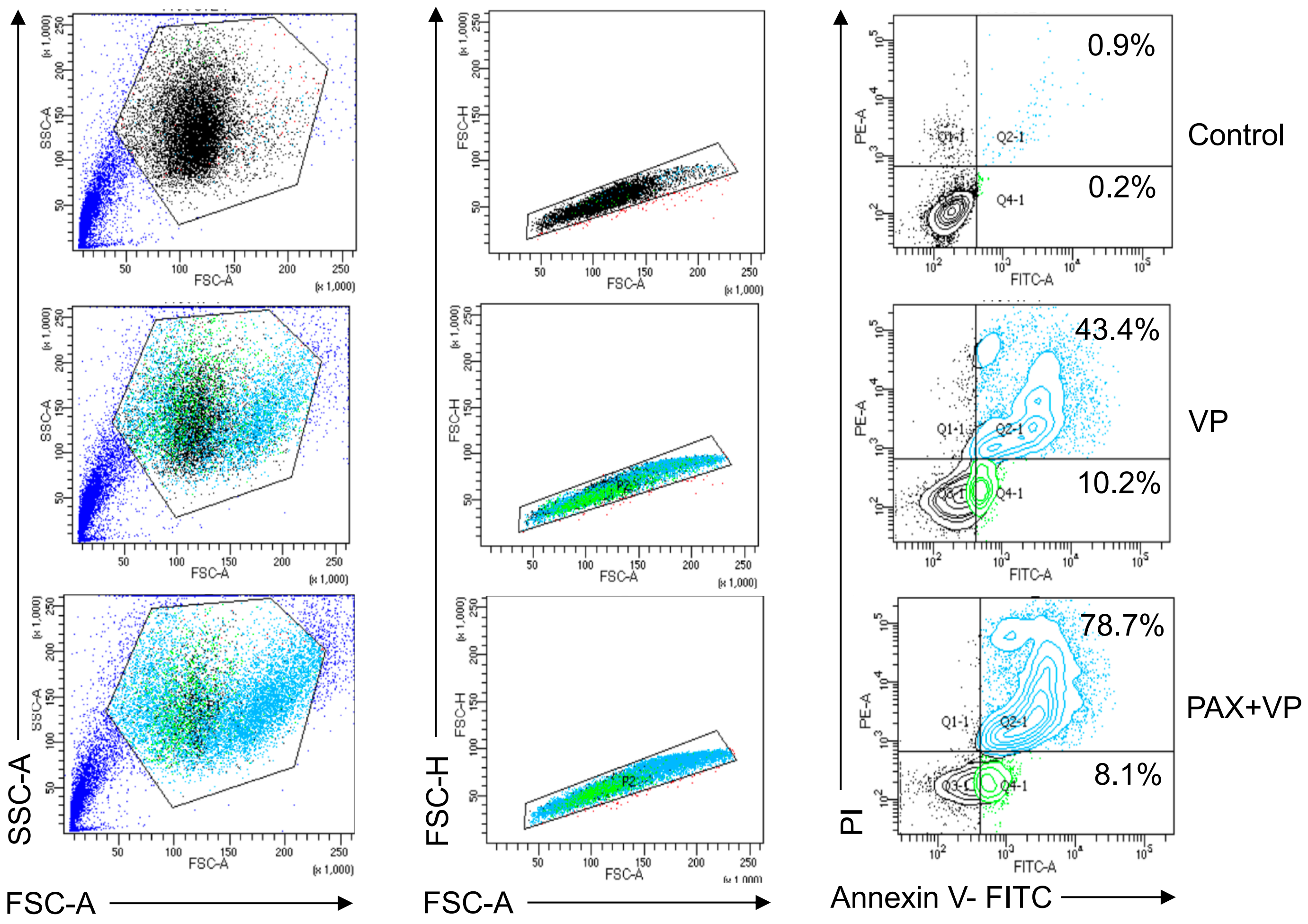
SSC-A
FSC-A
FSC-H
FSC-A
PI
Annexin V- FITC
Control
VP
PAX+VP
0.9%
0.2%
43.4%
10.2%
78.7%
8.1%
Q1-1
Q2-1
Q4-1
PE-A
FITC-A
P2

Figure 8g.

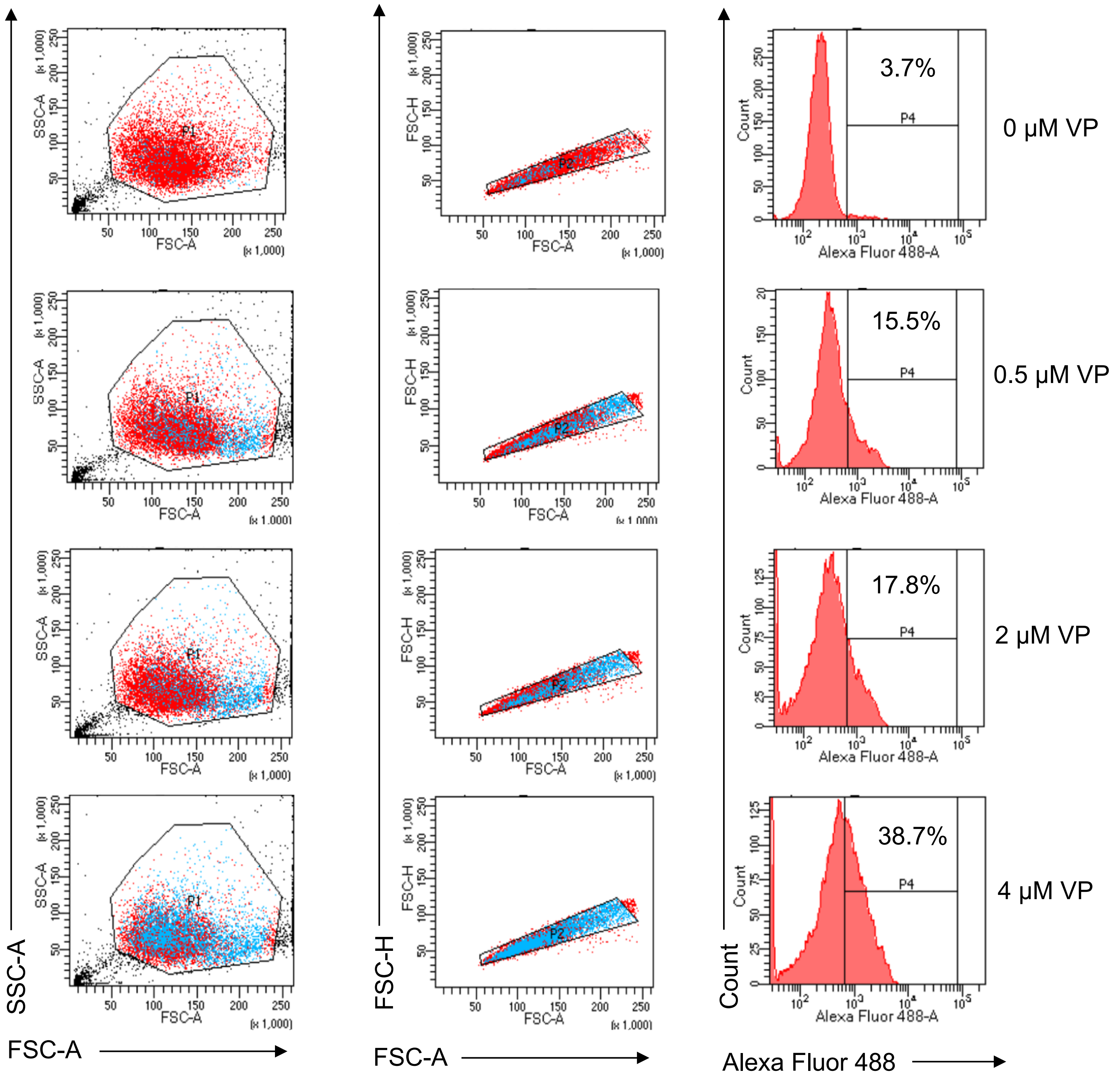

SSC-A
FSC-A
FSC-H
FSC-A
Count
Alexa Fluor 488
Alexa Fluor 488-A
P1
P2
P4
3.7%
0 µM VP
15.5%
0.5 µM VP
17.8%
2 µM VP
38.7%
4 µM VP

Gating strategy for flow cytometry

Supplementary Figure 1- Supplementary Figure 27

Supplementary Figure S3a.

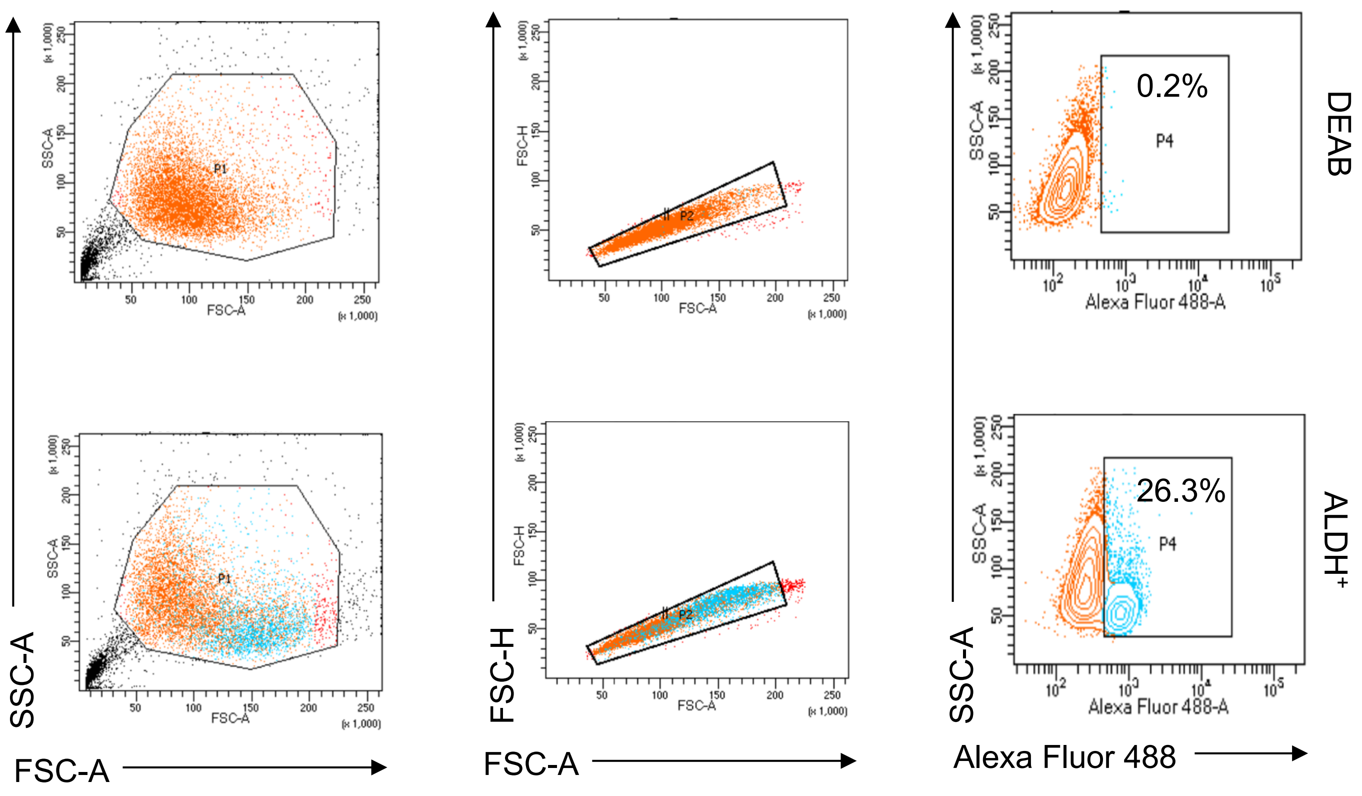

Supplementary Figure S4a.

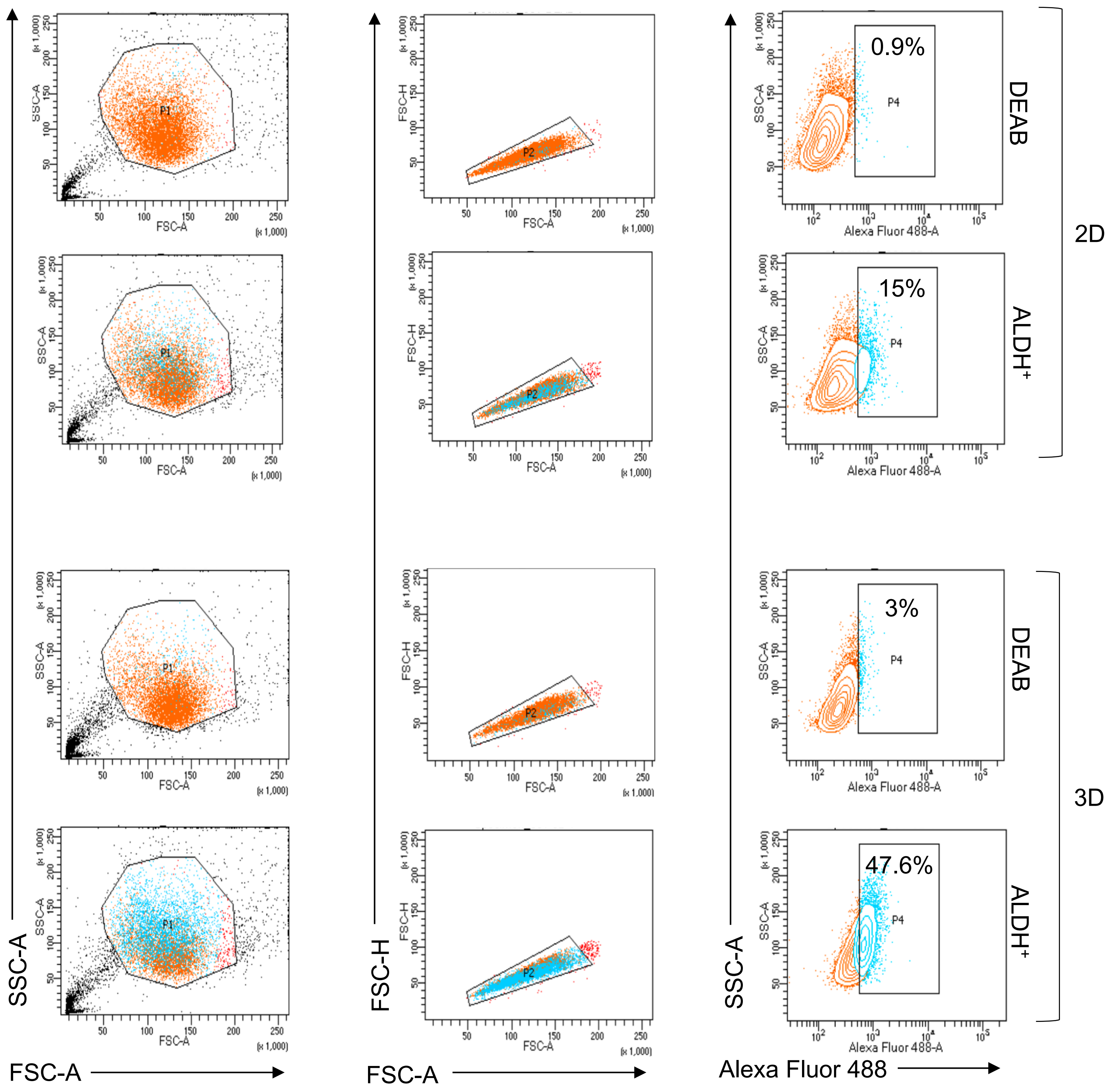

Supplementary Figure S11h.

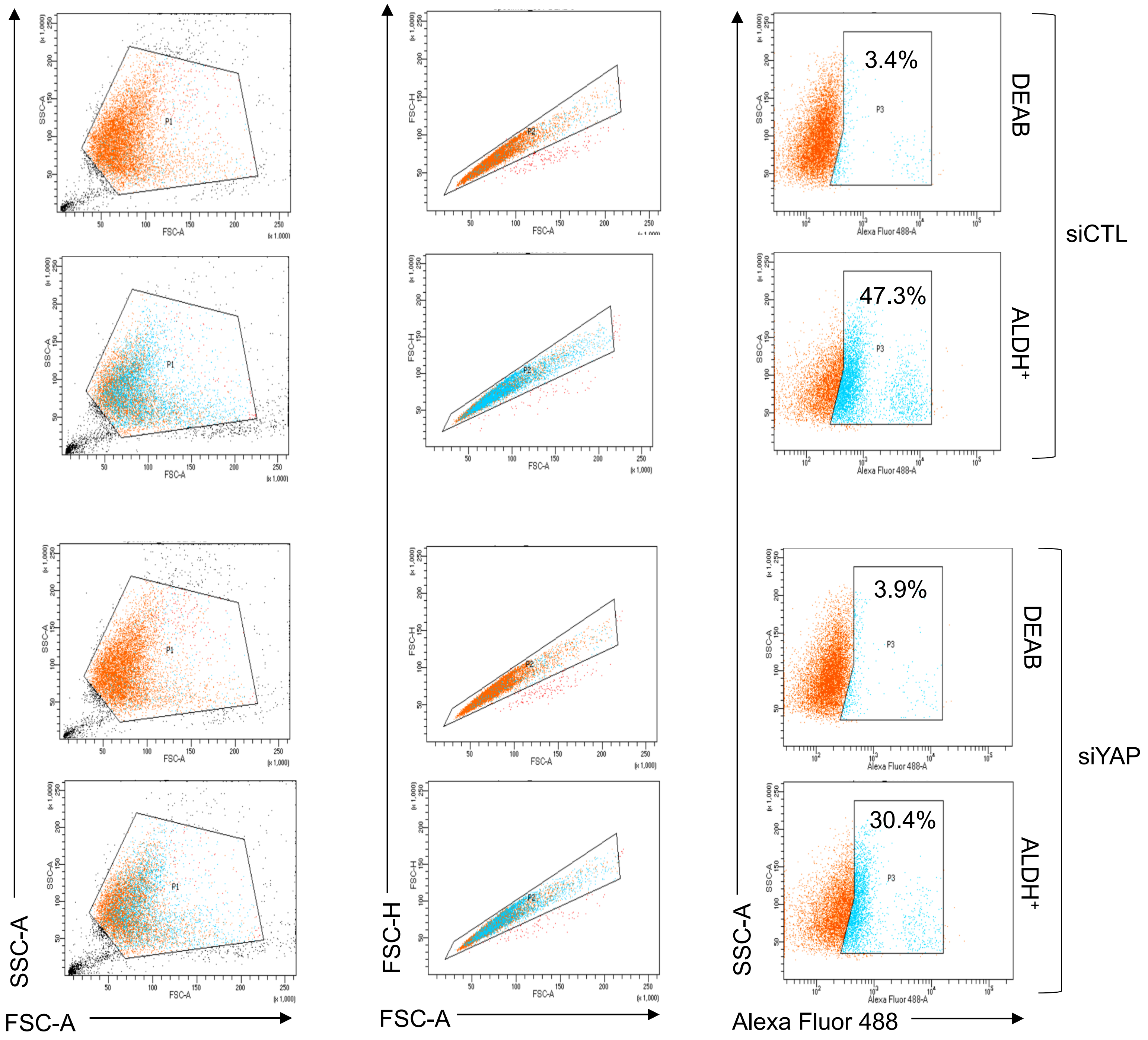

Supplementary Figure S11h.

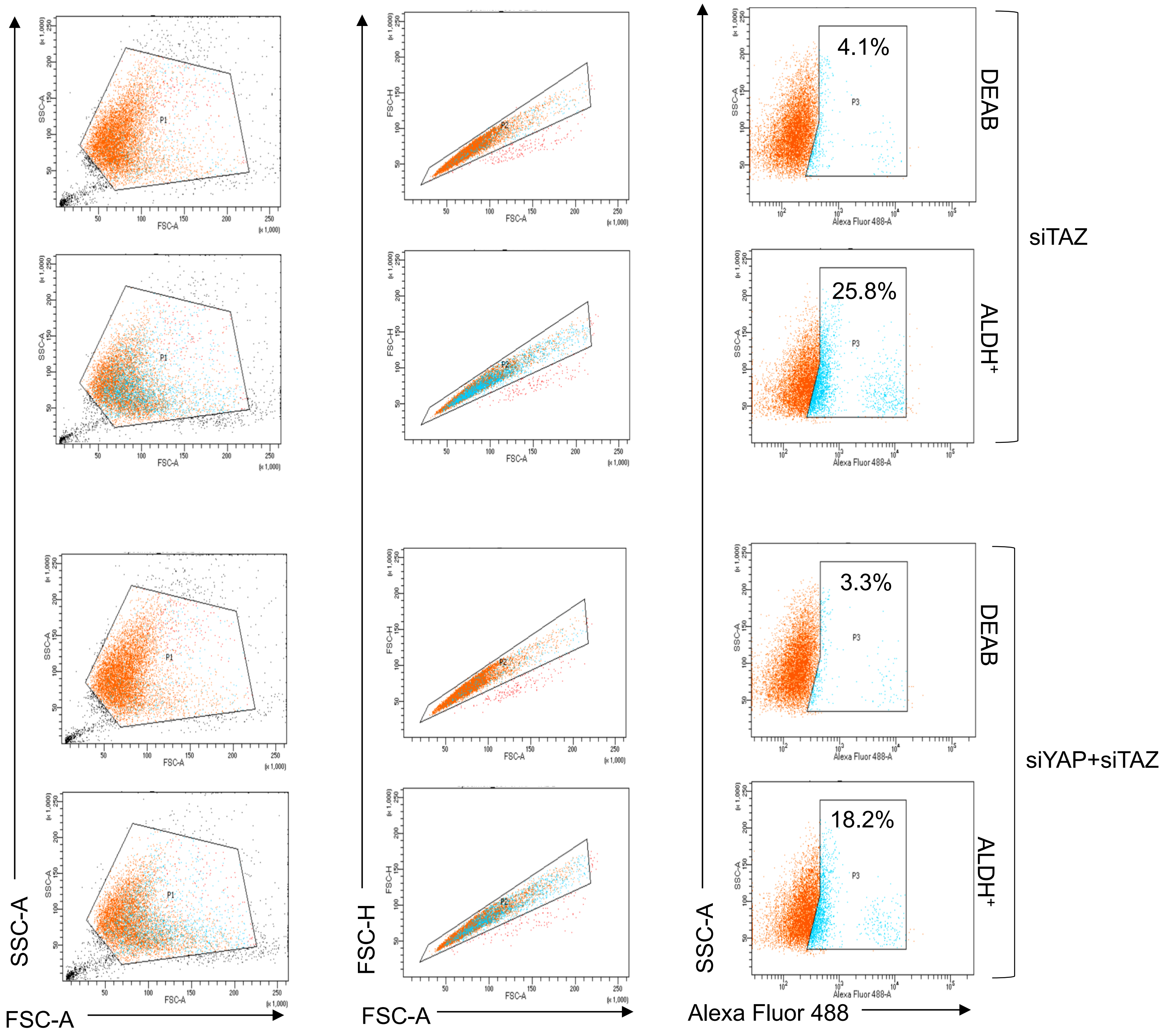

Supplementary Figure S12h.

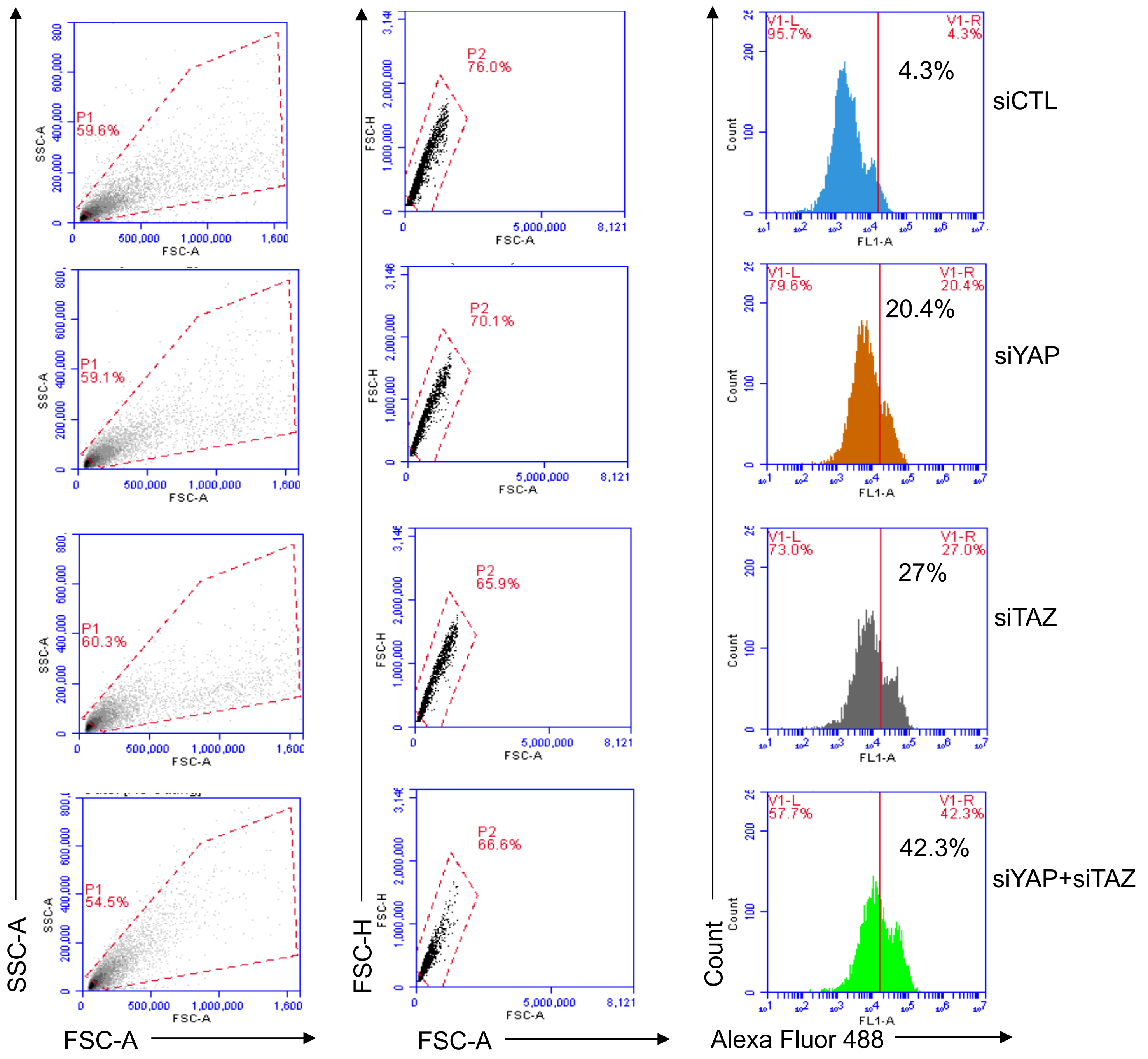

Supplementary Figure S24d.

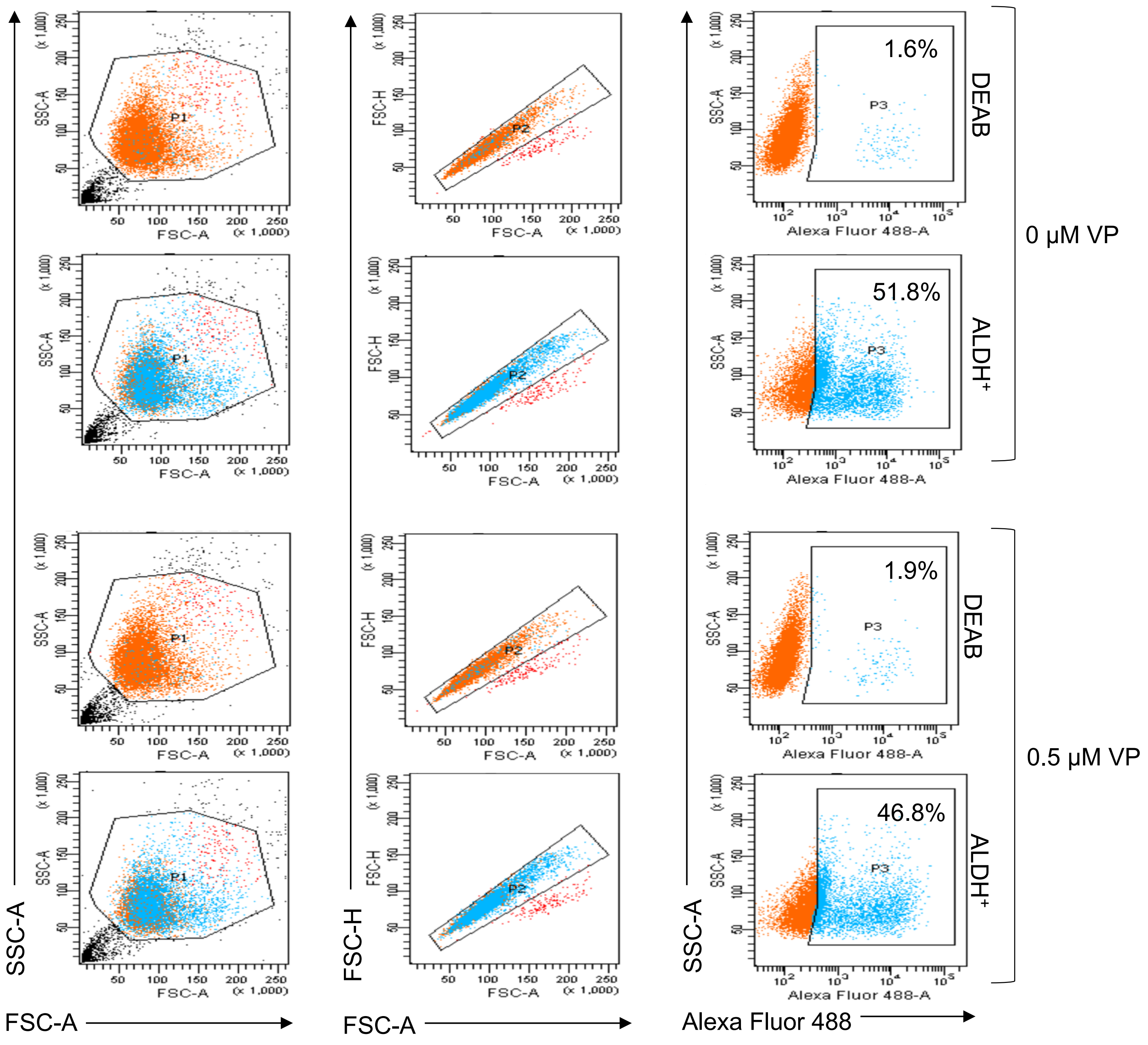

Supplementary Figure S24d.

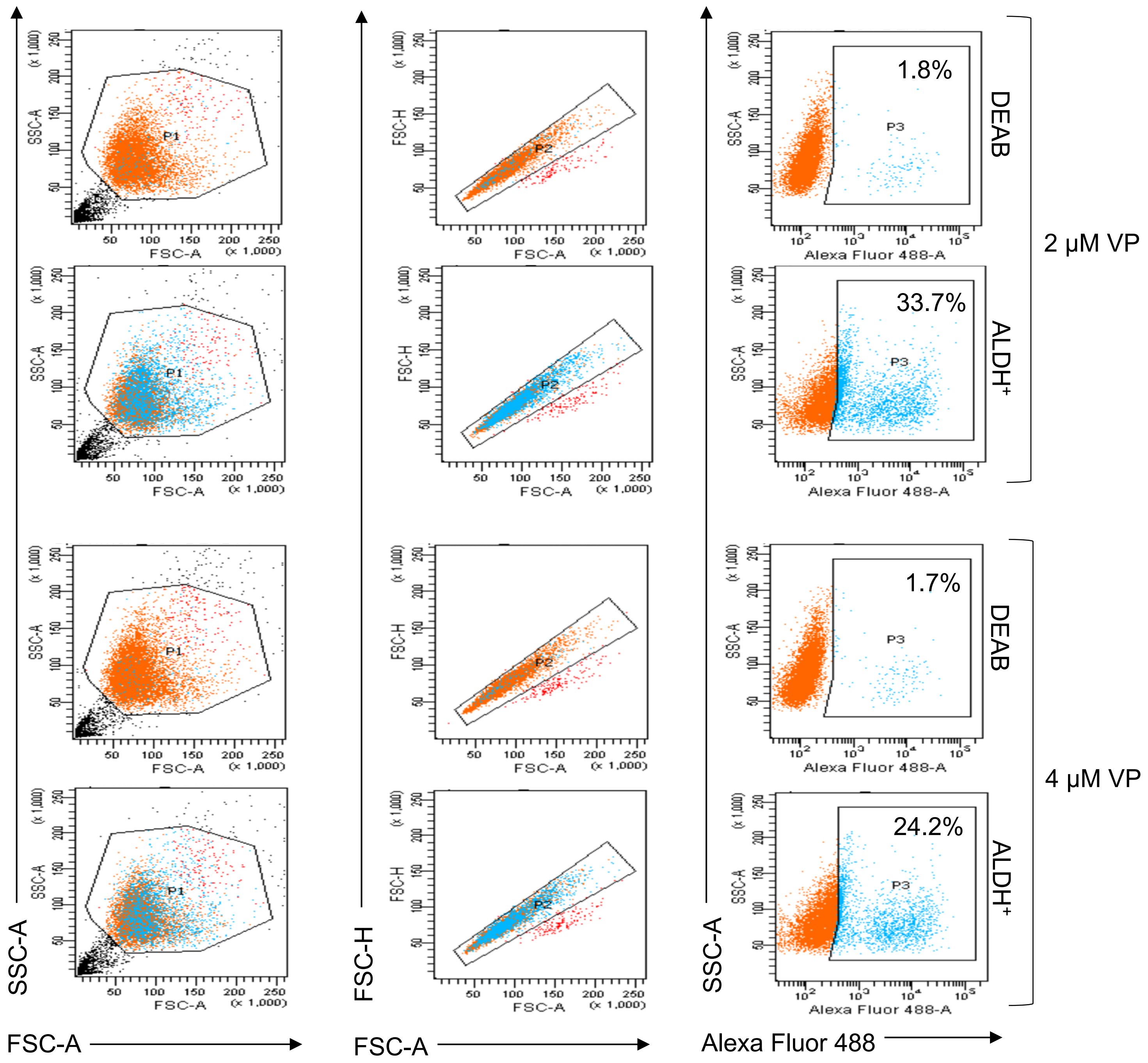

Supplementary Figure S25d.

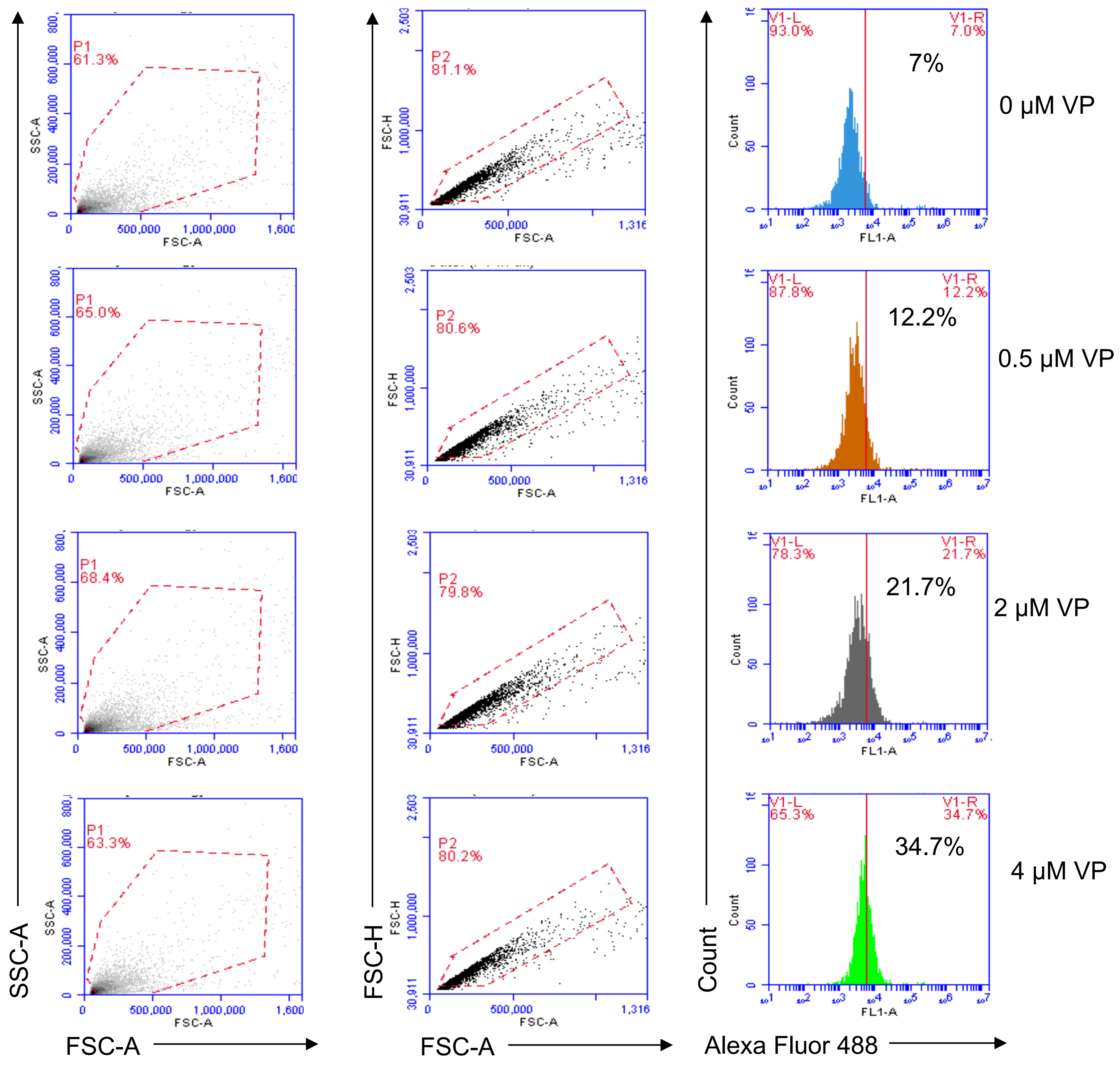

Supplement: Supplementary file 2 — Supplementary File [file 41392_2025_2133_MOESM2_ESM.pdf]
