# Supplementary material for: Precise targeting of transcriptional co-activators YAP/TAZ annihilates chemoresistant brCSCs by alteration of their mitochondrial homeostasis
Source: Signal Transduct Target Ther. 2025 Feb 21;10:61. doi: 10.1038/s41392-025-02133-x (PMC11842803; doi:10.1038/s41392-025-02133-x)

Gating strategy for CD44/CD24 immunostaining using flow cytometry

Fig 2f

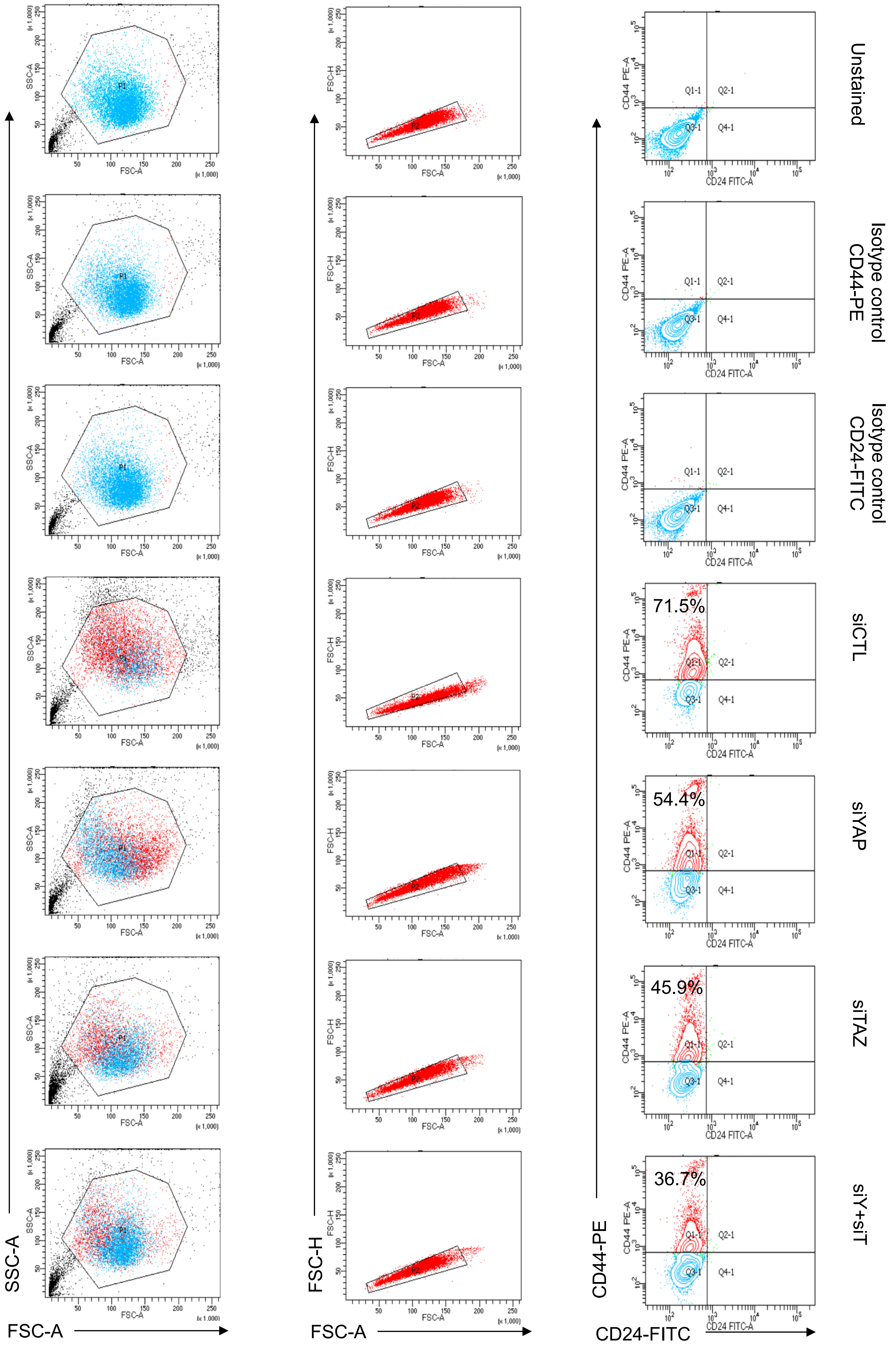

Unstained
Isotype control CD44-PE
Isotype control CD24-FITC
siCTL
siYAP
siTAZ
siY+siT
71.5%
54.4%
45.9%
36.7%
SSC-A
FSC-A
FSC-H
FSC-A
CD44-PE
CD24-FITC
P1
P2
Q1-1
Q2-1
Q3-1
Q4-1

Fig 5e

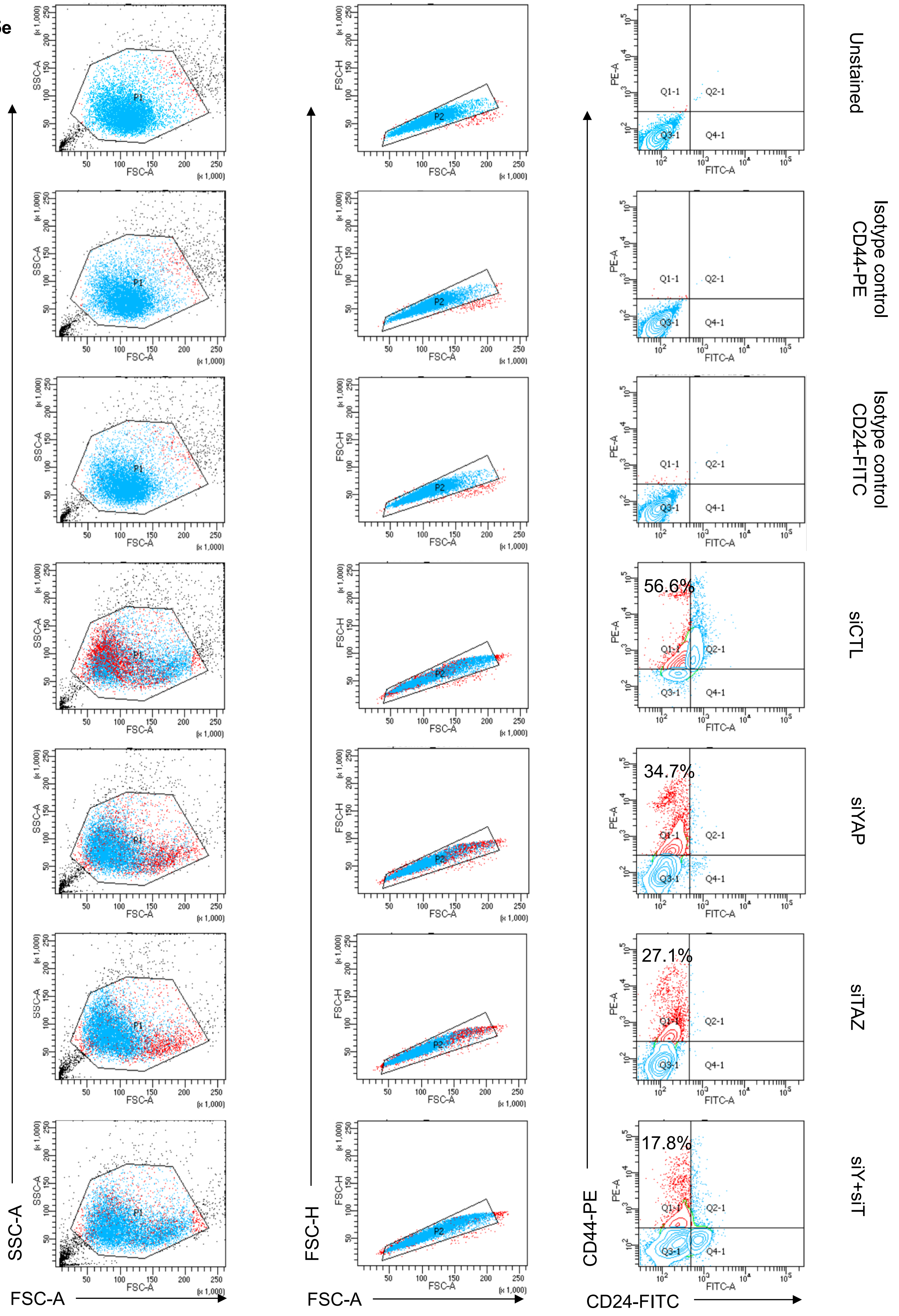

Unstained
Isotype control CD44-PE
Isotype control CD24-FITC
siCTL
siYAP
siTAZ
siY+siT
56.6%
34.7%
27.1%
17.8%
SSC-A
FSC-A
FSC-H
FSC-A
CD44-PE
CD24-FITC
P1
P2
Q1-1
Q2-1
Q3-1
Q4-1
PE-A
FITC-A
(x 1,000)

Fig 6g

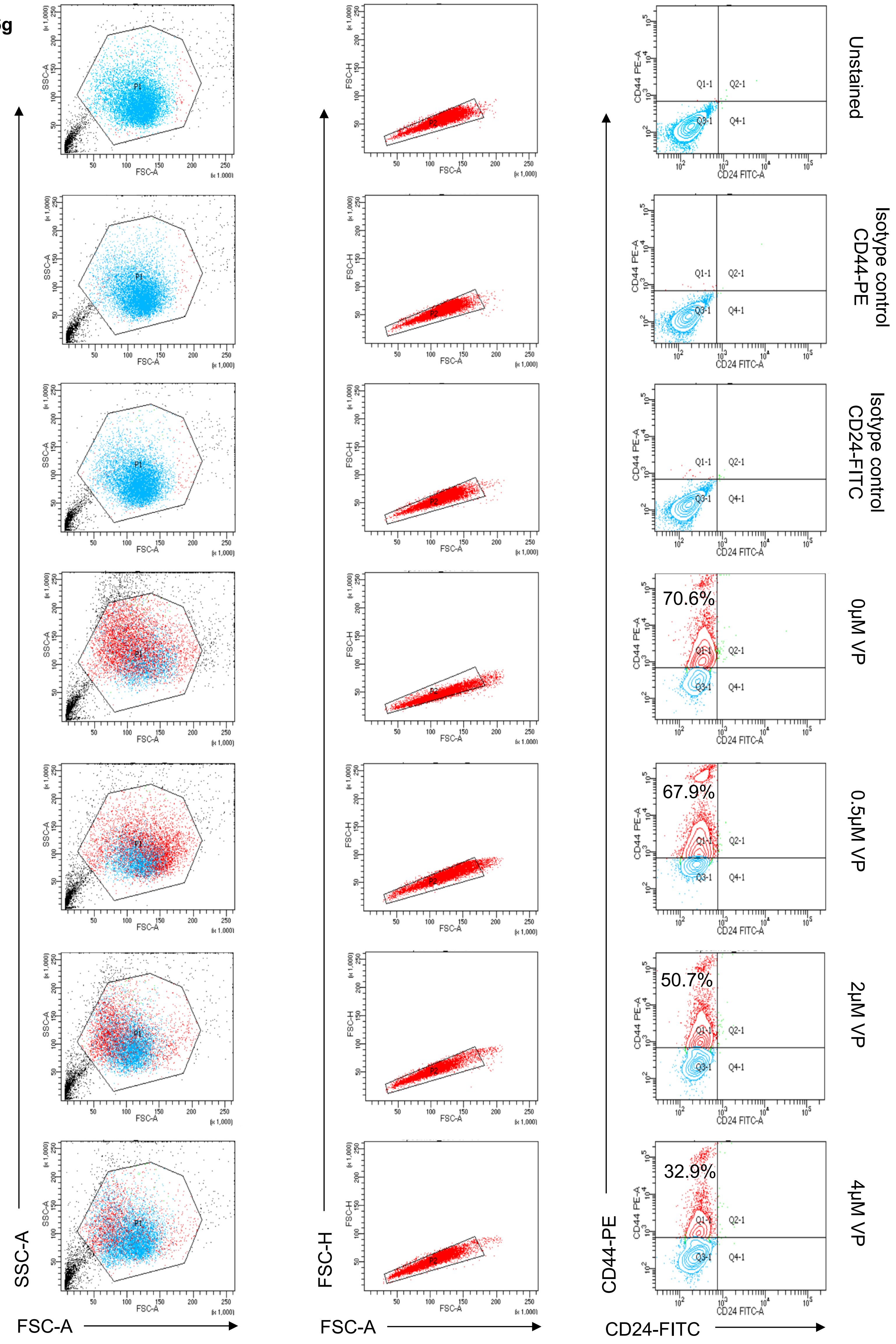
Unstained
Isotype control CD44-PE
Isotype control CD24-FITC
0μM VP
0.5μM VP
2μM VP
4μM VP
70.6%
67.9%
50.7%
32.9%
SSC-A
FSC-A
FSC-H
FSC-A
CD44-PE
CD24-FITC

Fig 6i

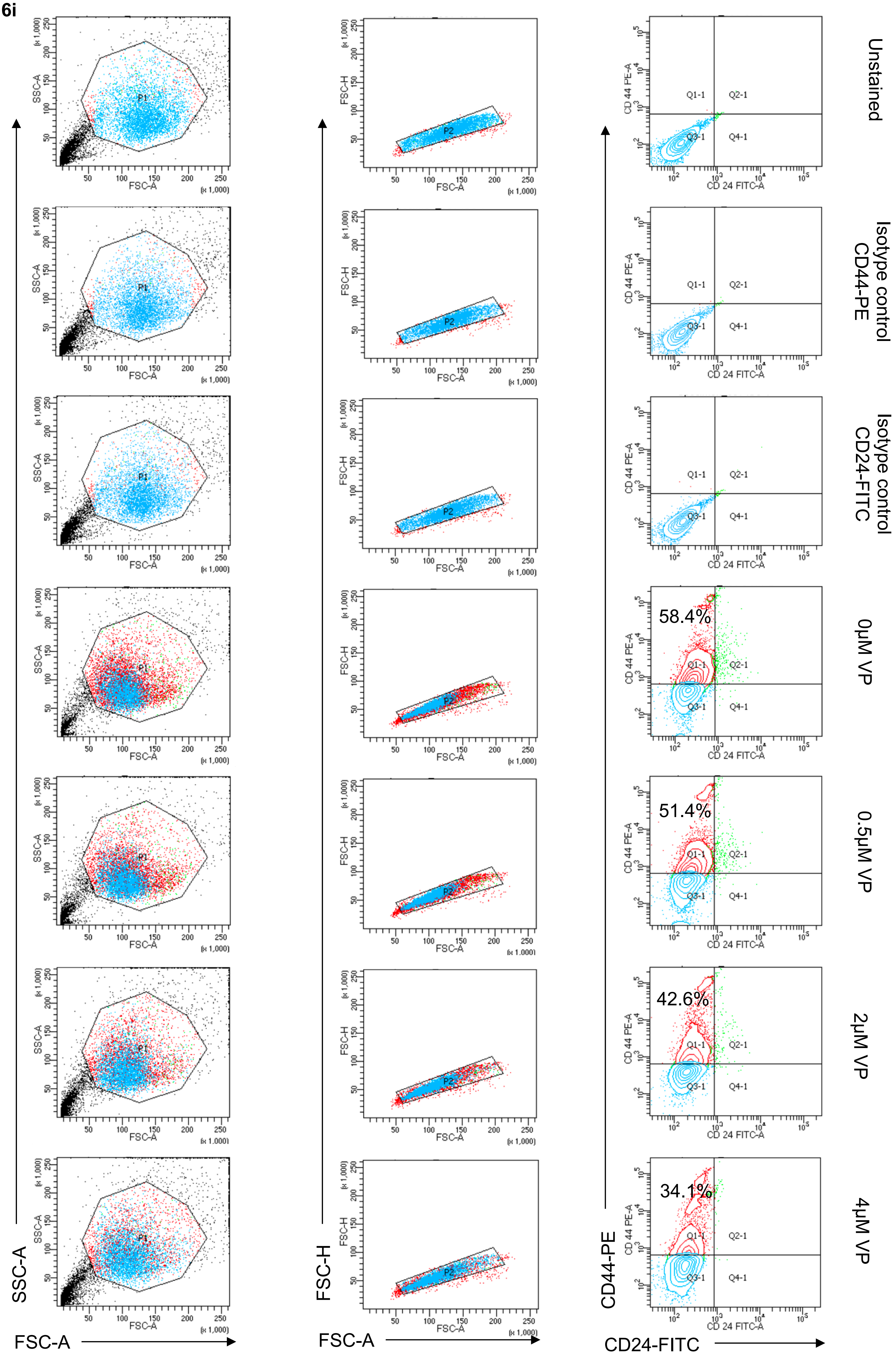

Unstained
Isotype control CD44-PE
Isotype control CD24-FITC
0µM VP
0.5µM VP
2µM VP
4µM VP
58.4%
51.4%
42.6%
34.1%
Q1-1
Q2-1
Q3-1
Q4-1
P1
P2
SSC-A
FSC-A
FSC-H
CD 44 PE-A
CD 24 FITC-A
CD44-PE
CD24-FITC

Fig 8f

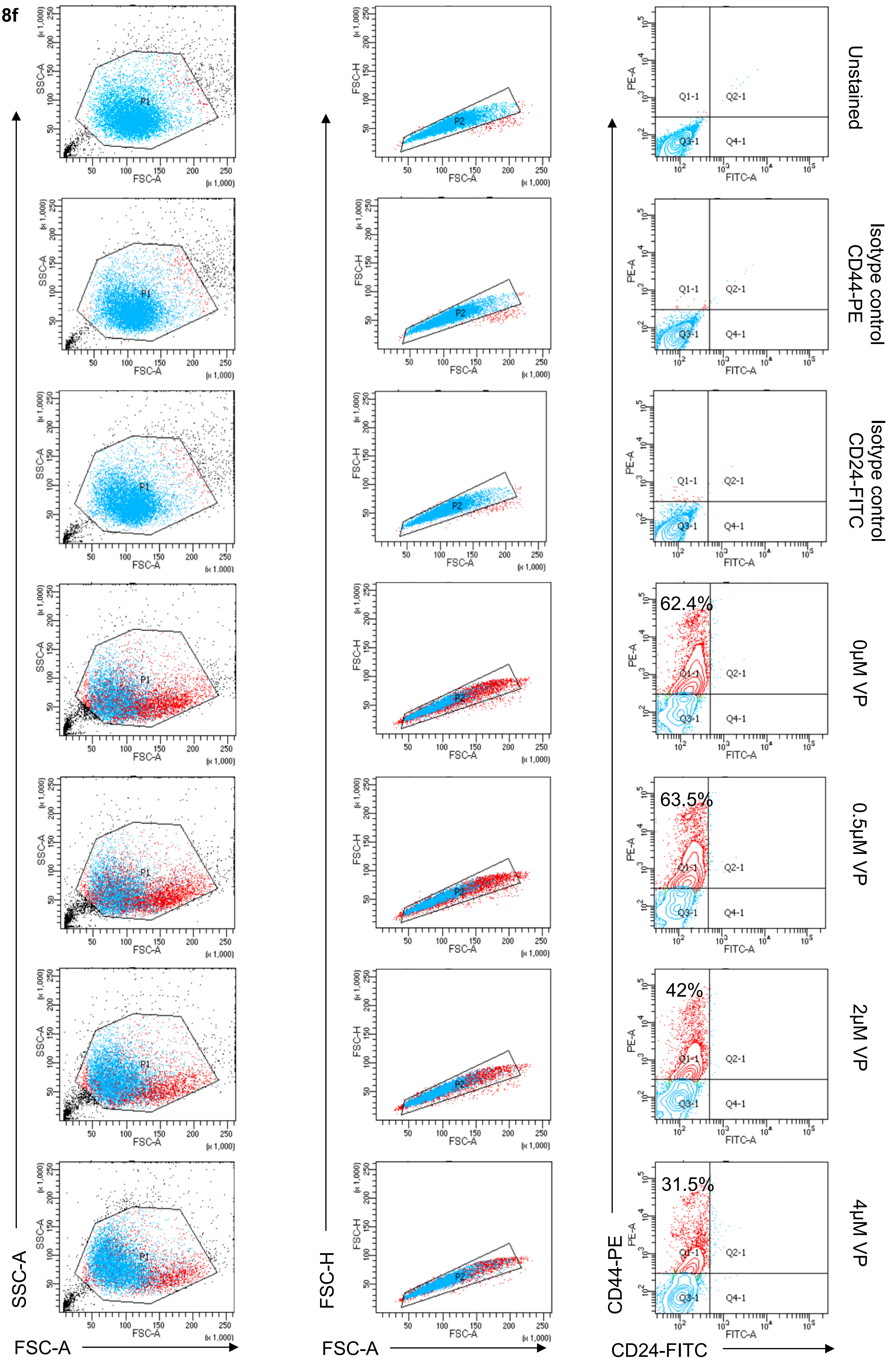
SSC-A
FSC-A
FSC-H
FSC-A
CD44-PE
CD24-FITC
P1
P2
Q1-1
Q2-1
Q3-1
Q4-1
PE-A
FITC-A
Unstained
Isotype control CD44-PE
Isotype control CD24-FITC
0µM VP
62.4%
0.5µM VP
63.5%
2µM VP
42%
4µM VP
31.5%

Supplementary Fig S3a

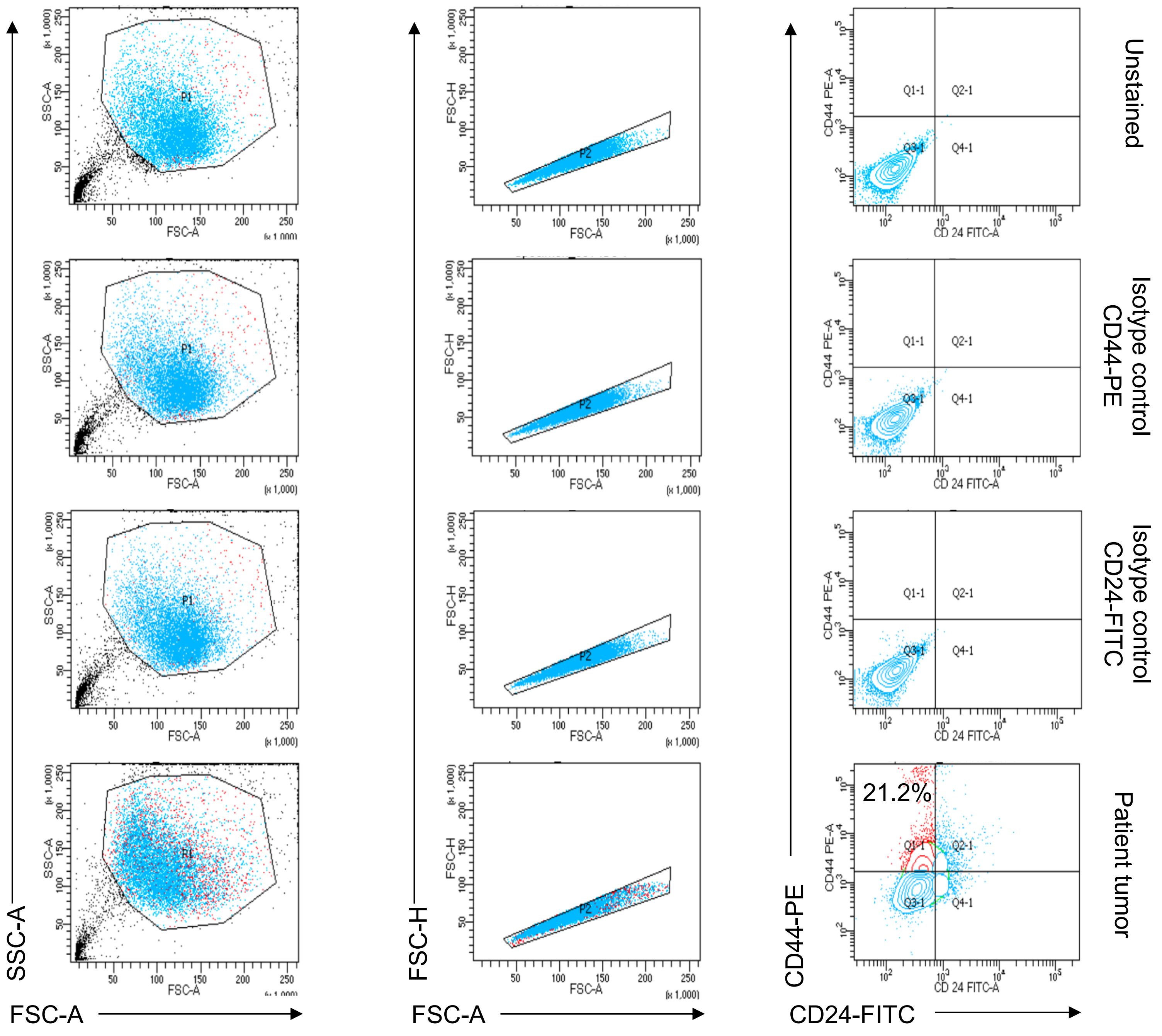

Supplementary Fig S4c

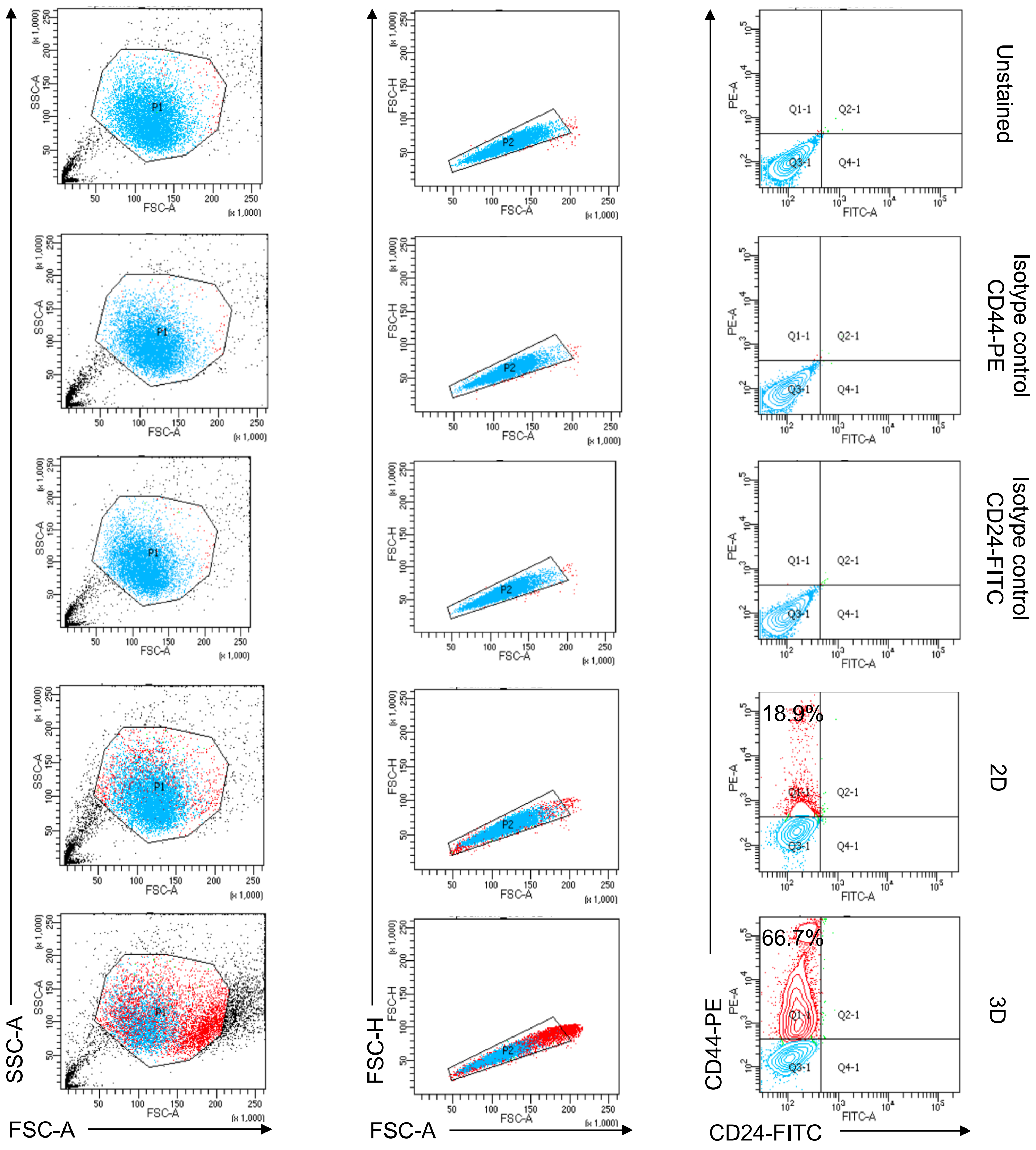

Supplement: Supplementary file 3 — Supplementary files [file 41392_2025_2133_MOESM3_ESM.pdf]
