# Supplementary material for: Precise targeting of transcriptional co-activators YAP/TAZ annihilates chemoresistant brCSCs by alteration of their mitochondrial homeostasis
Source: Signal Transduct Target Ther. 2025 Feb 21;10:61. doi: 10.1038/s41392-025-02133-x (PMC11842803; doi:10.1038/s41392-025-02133-x)
Supplement: Supplementary file 4 — Data Set 1 [file 41392_2025_2133_MOESM4_ESM.pdf]

Western Blots

Figures 1 to 8

Fig. 1a

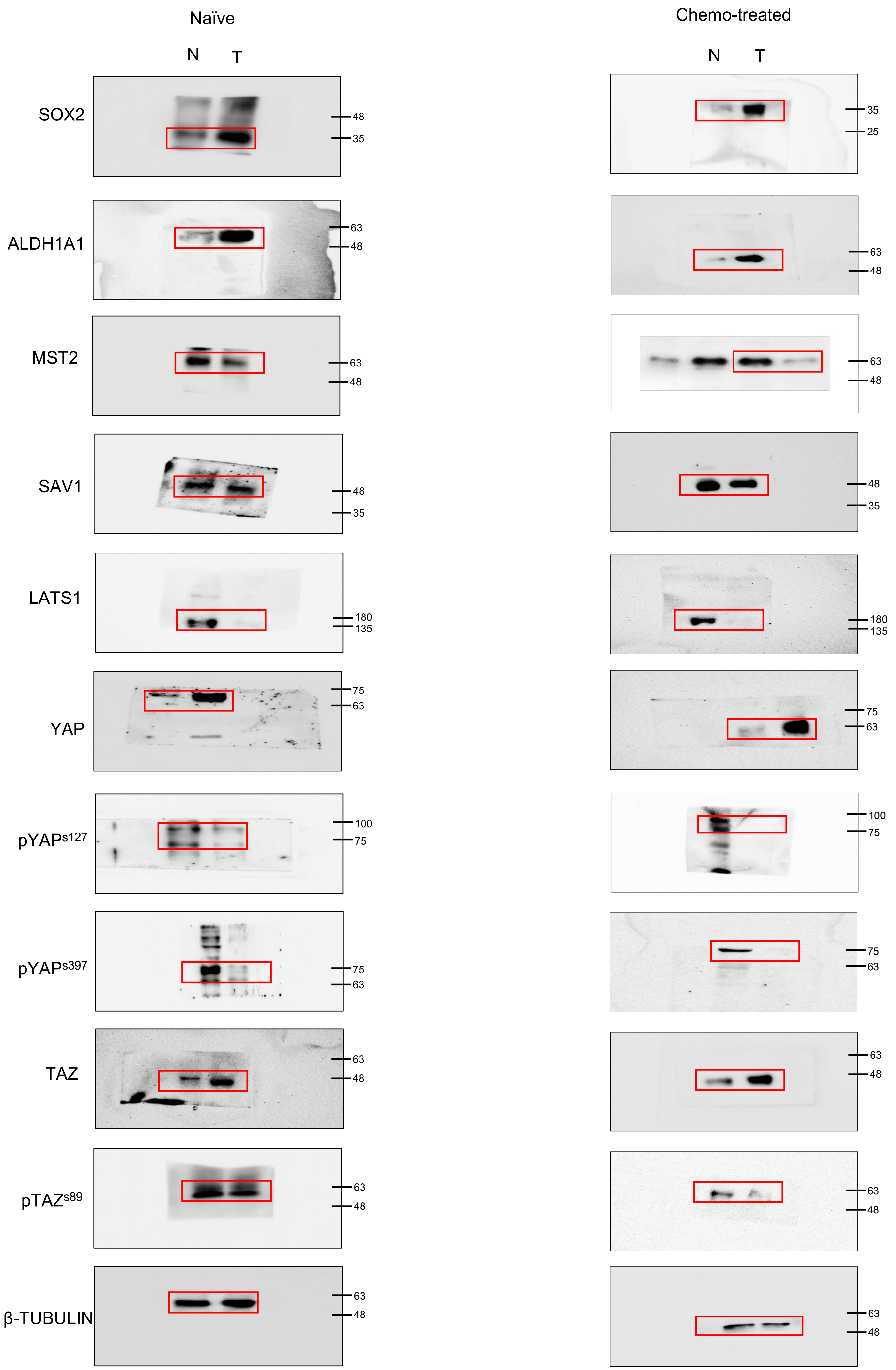

#The results are derived from multiple blots, with constitutive proteins consistently analyzed

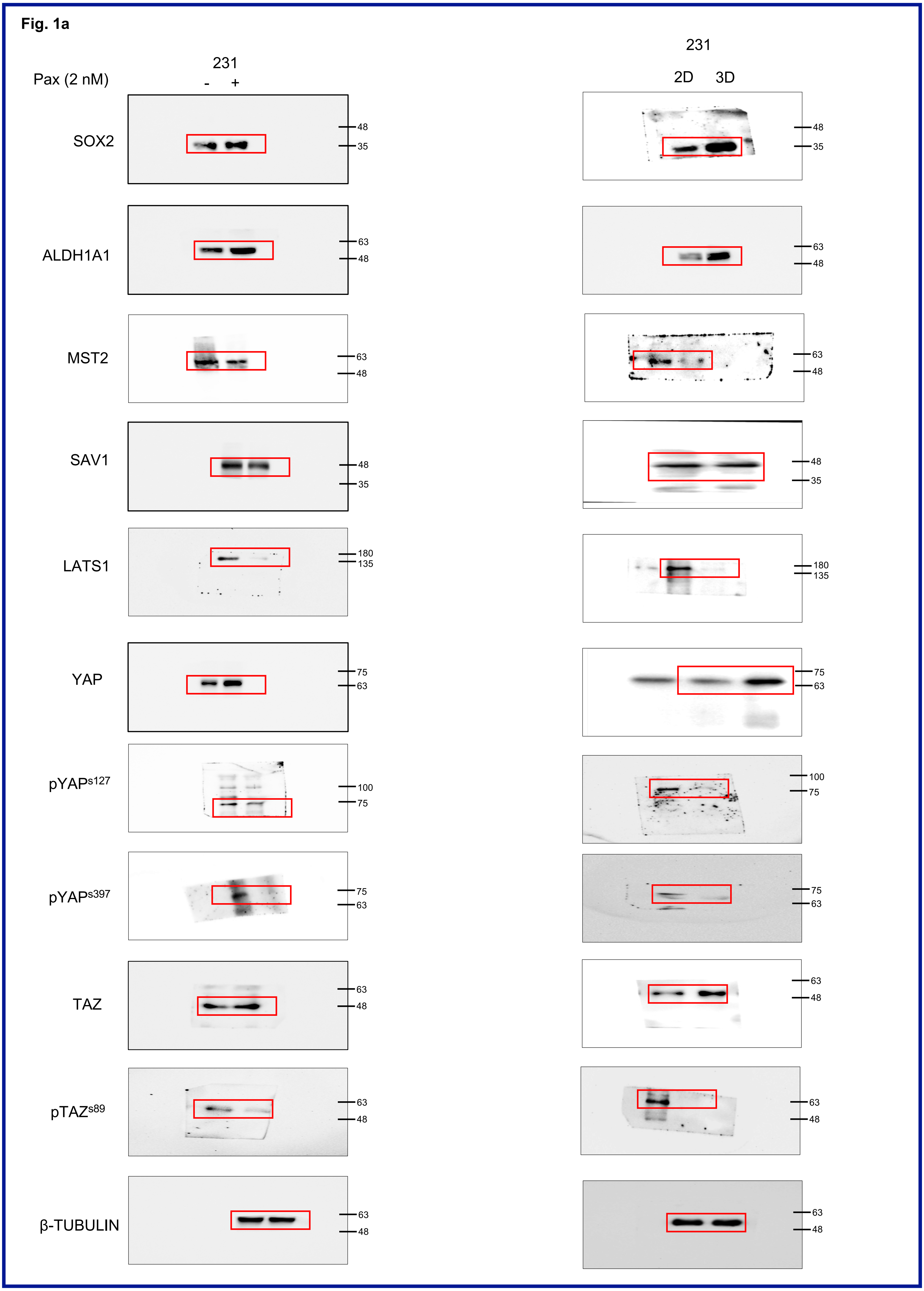

#The results are derived from multiple blots, with constitutive proteins consistently analyzed

Fig. 1c

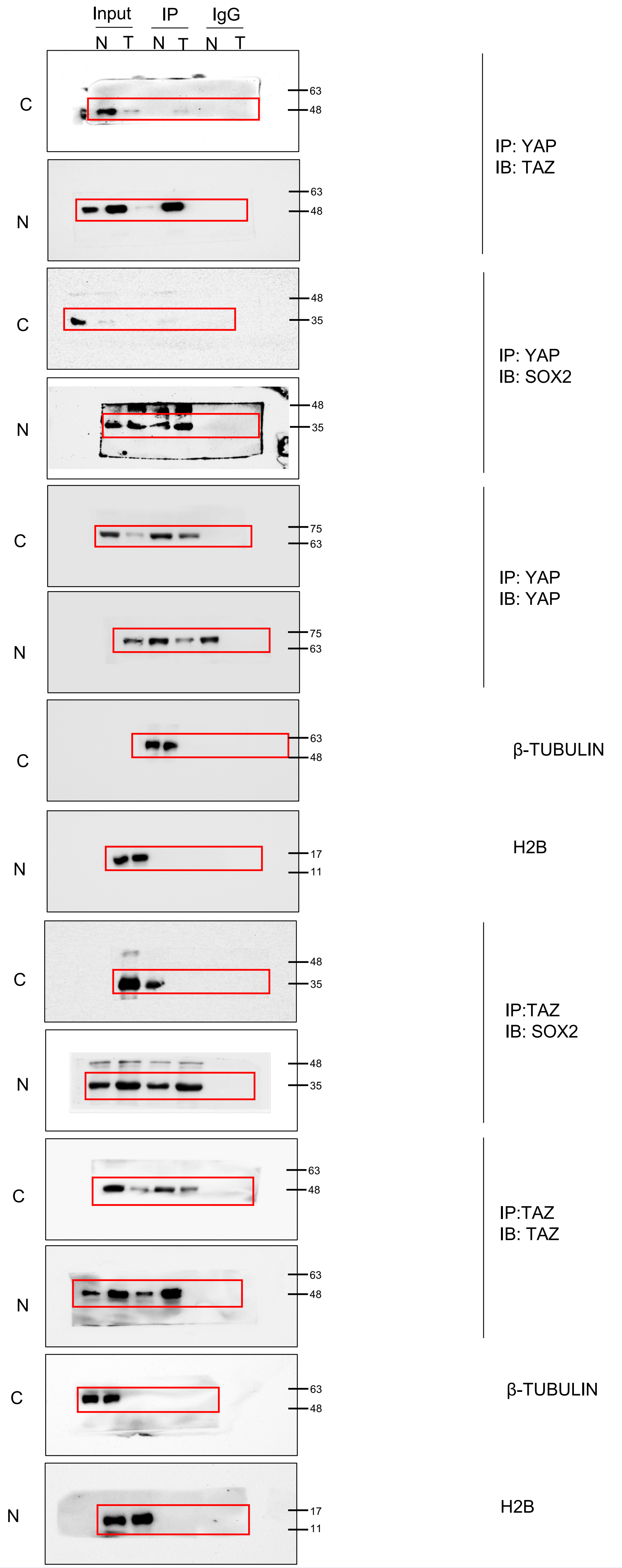

#The results are derived from multiple blots, with constitutive proteins consistently analyzed

Fig. 1d

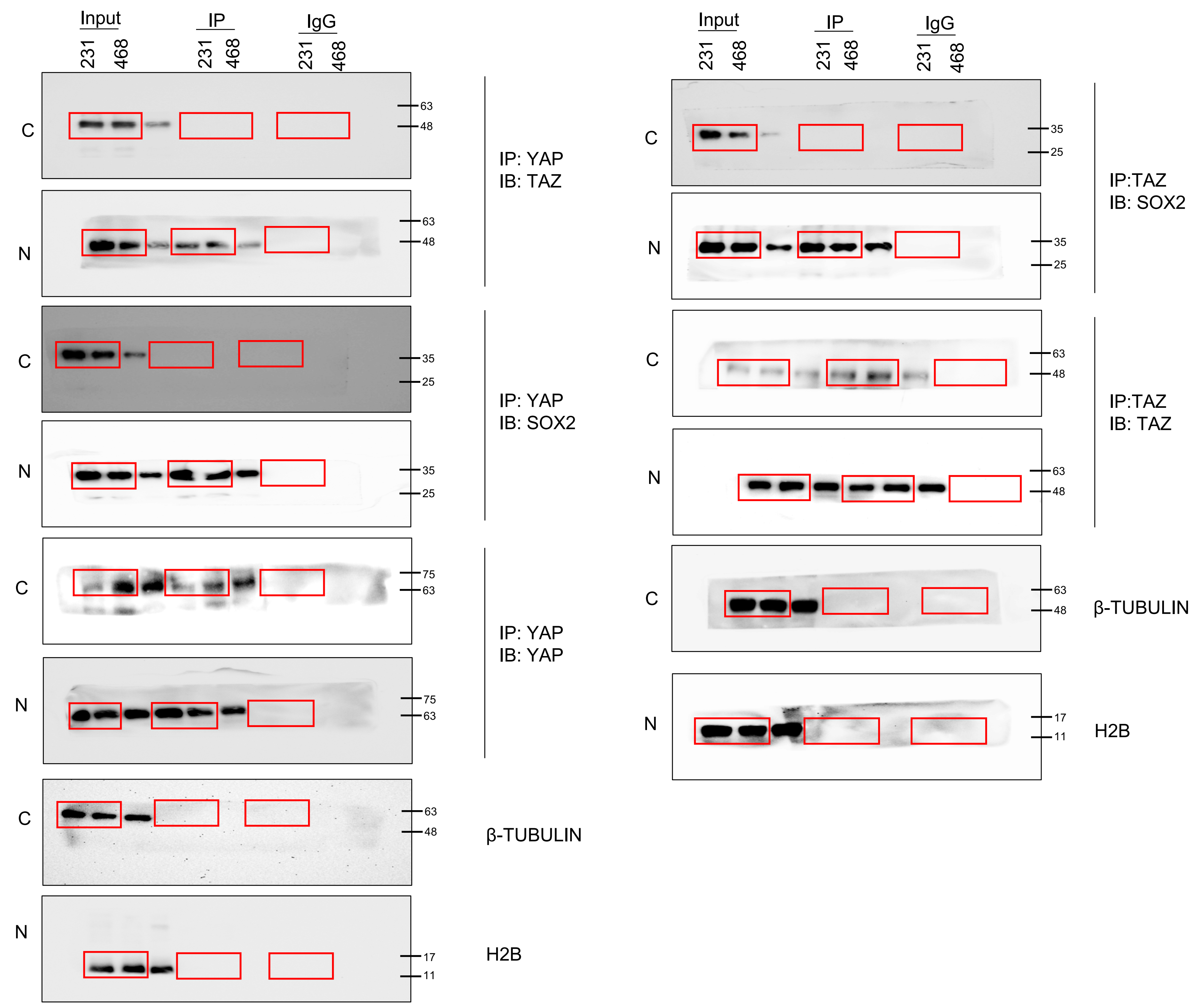

#The results are derived from multiple blots, with constitutive proteins consistently analyzed

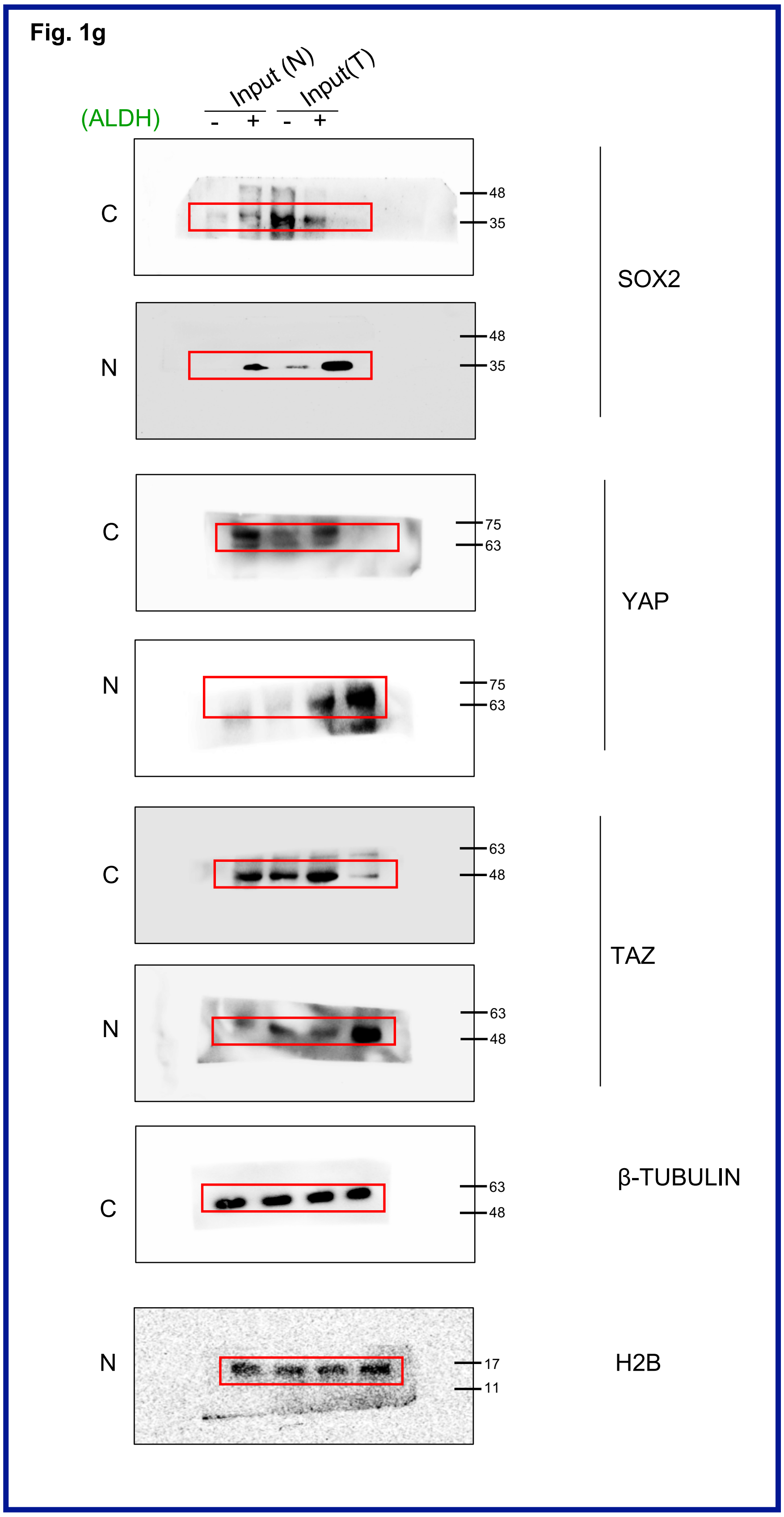

#The results are derived from multiple blots, with constitutive proteins consistently analyzed

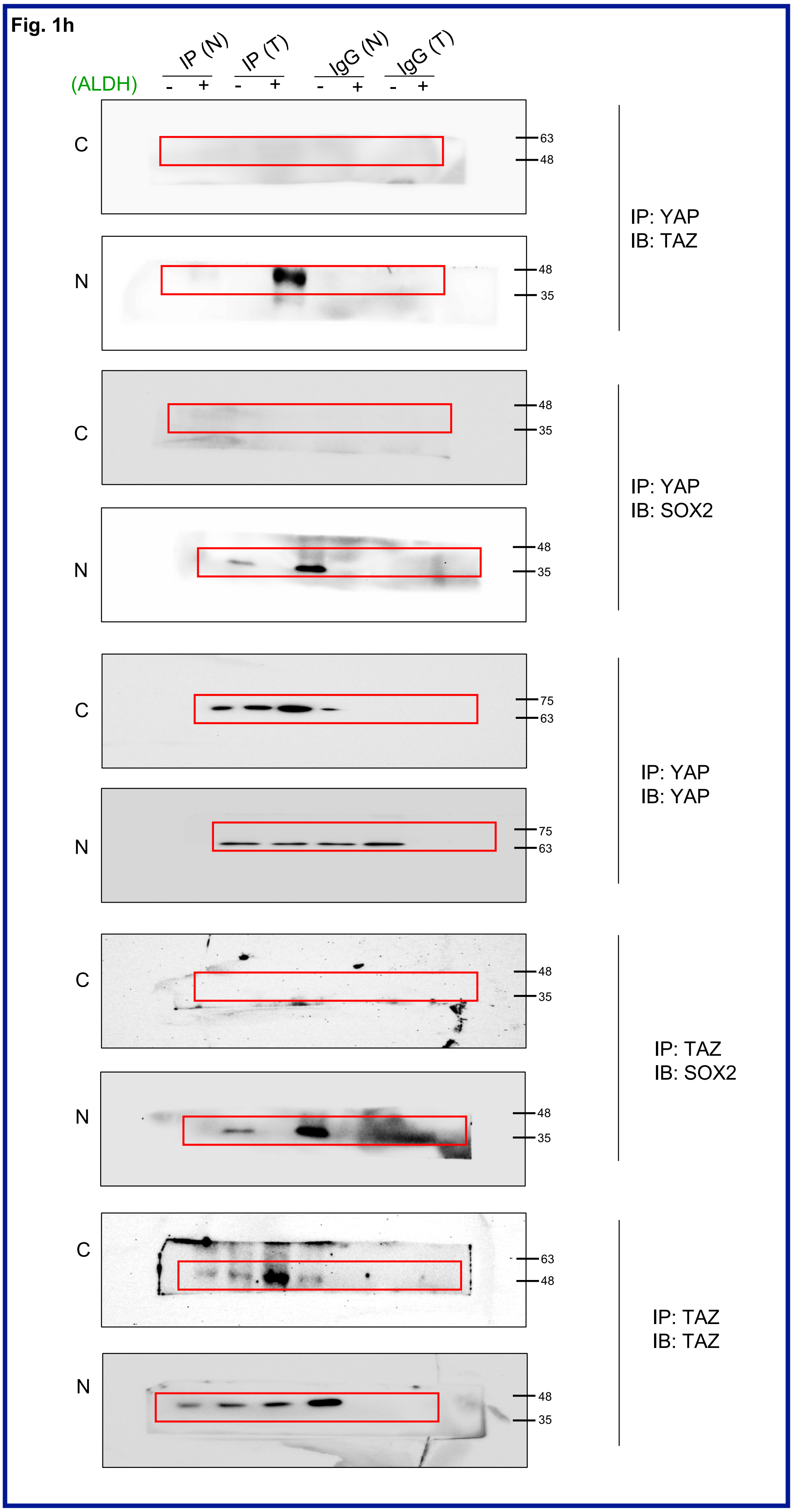

#The results are derived from multiple blots, with constitutive proteins consistently analyzed

Fig. 1m

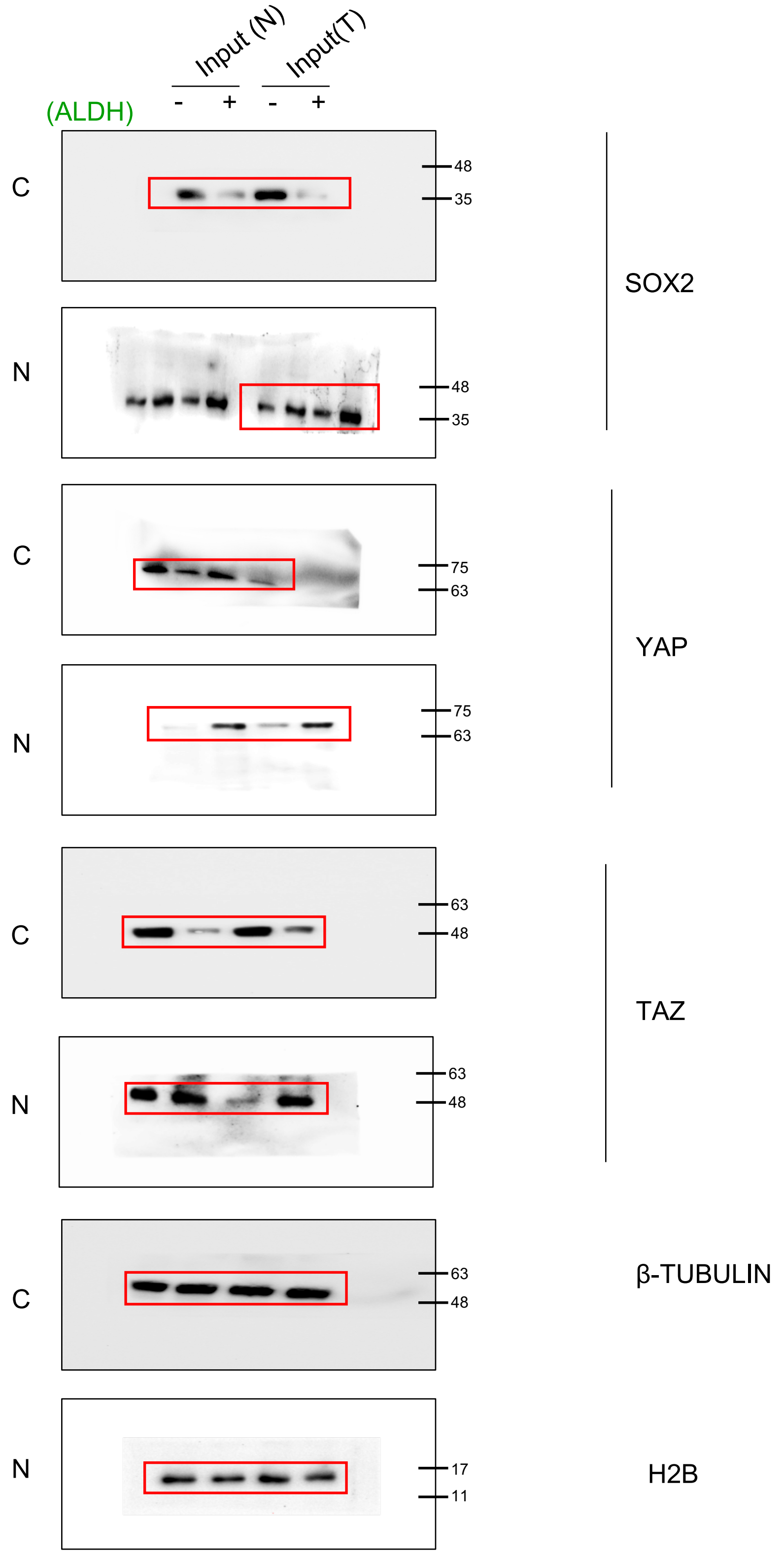

#The results are derived from multiple blots, with constitutive proteins consistently analyzed

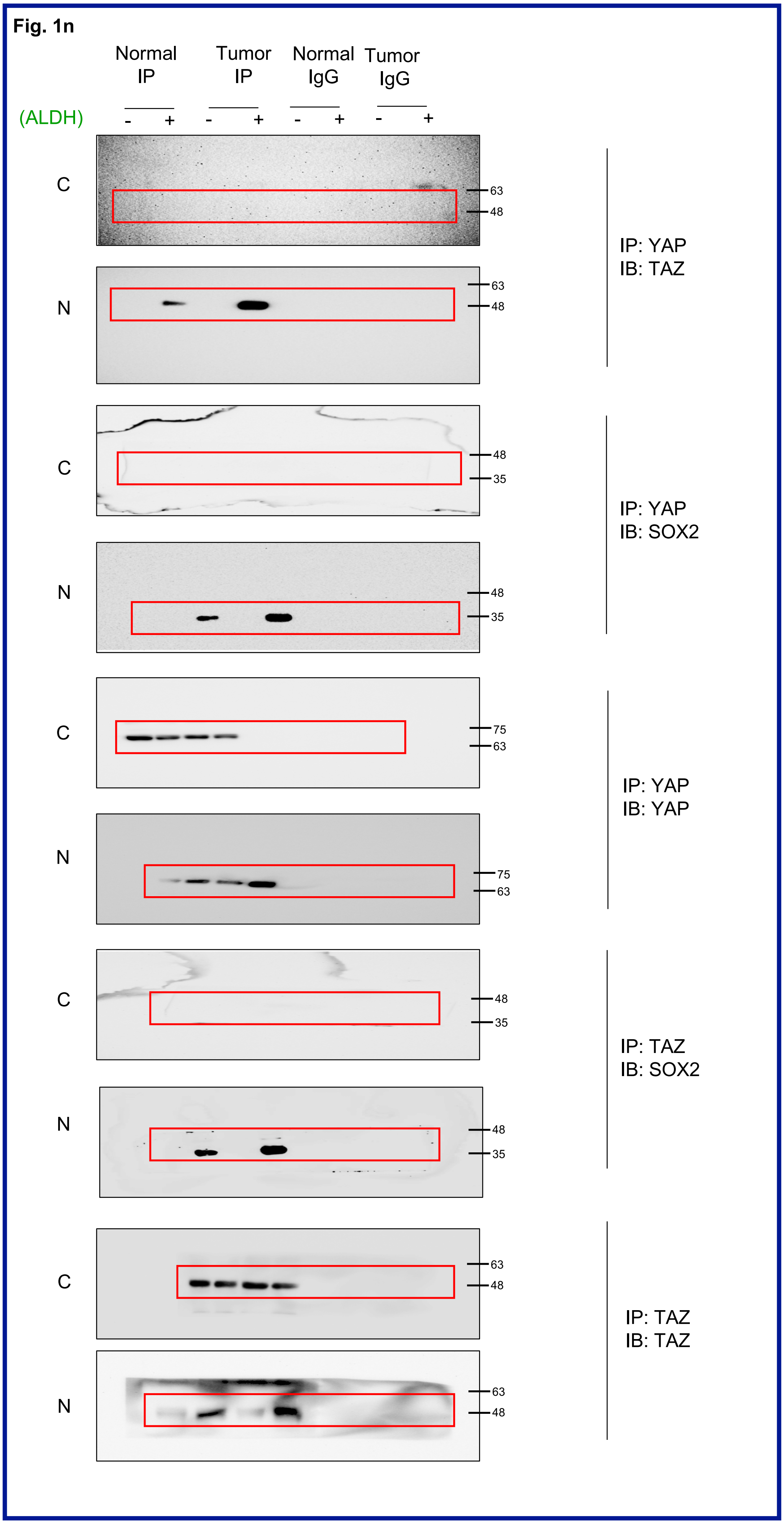

#The results are derived from multiple blots, with constitutive proteins consistently analyzed

Fig. 1o

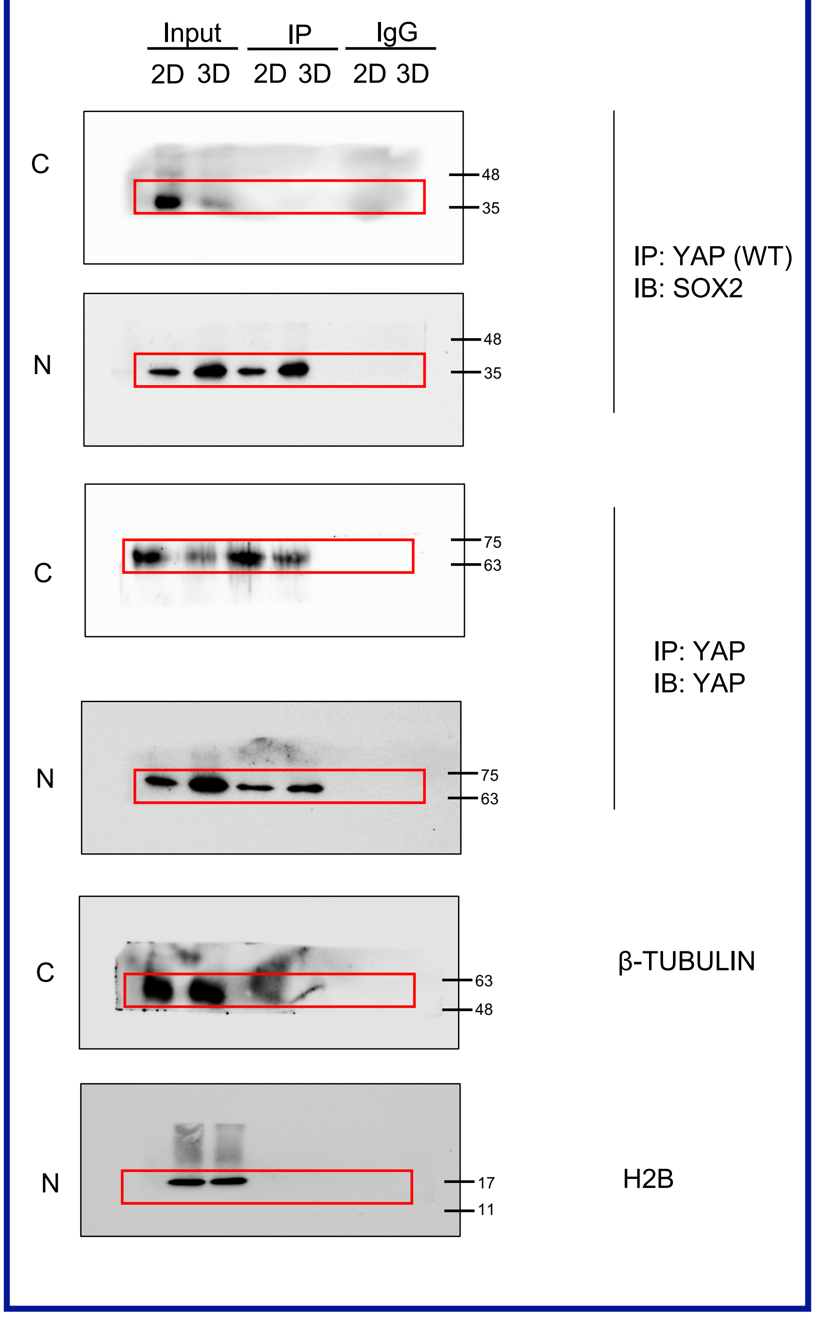

Fig. 1p

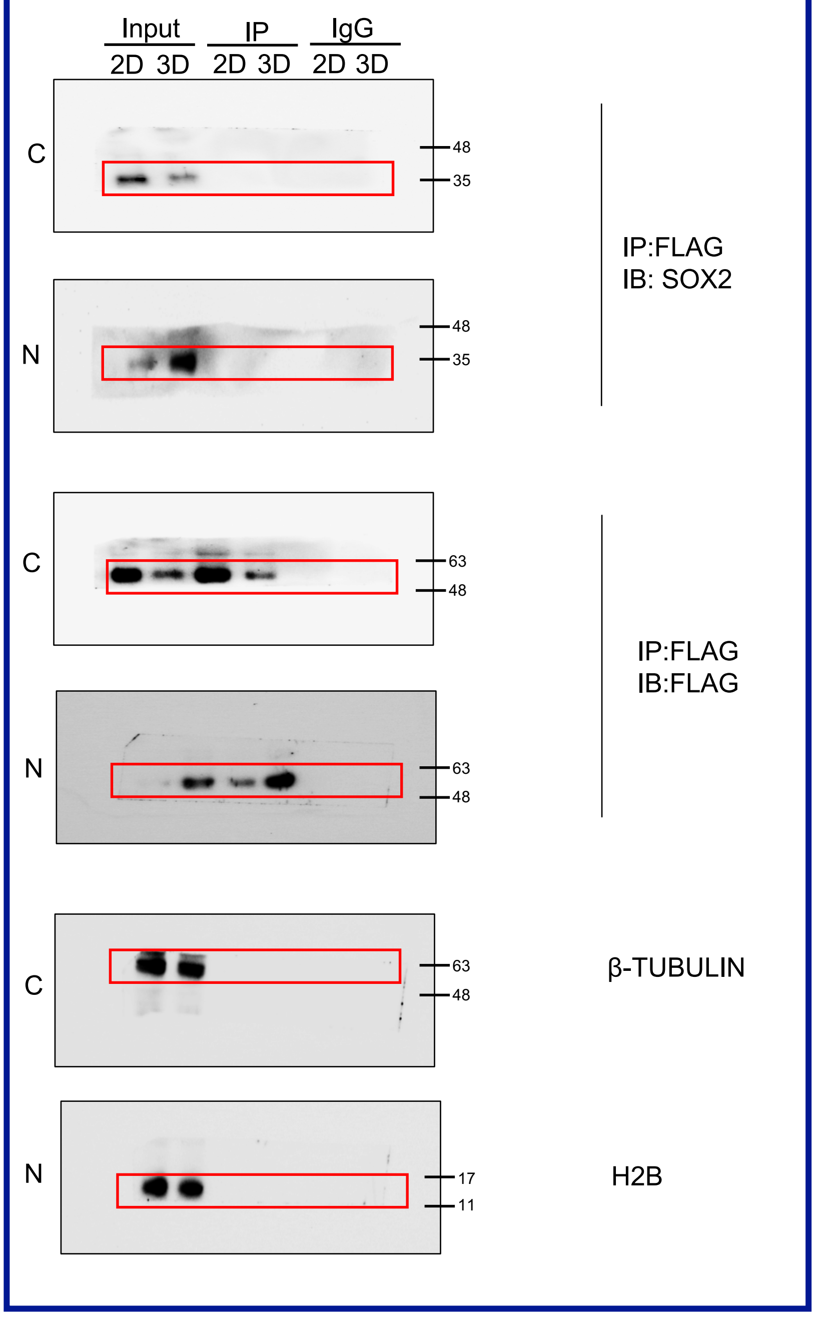

#The results are derived from multiple blots, with constitutive proteins consistently analyzed

Fig. 2c

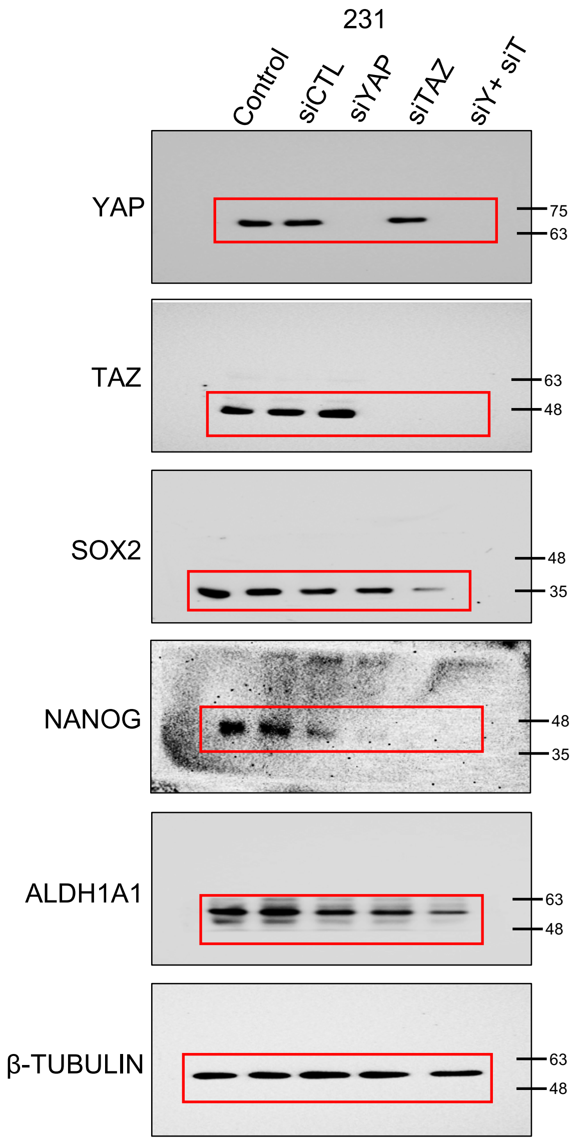

Fig. 2j

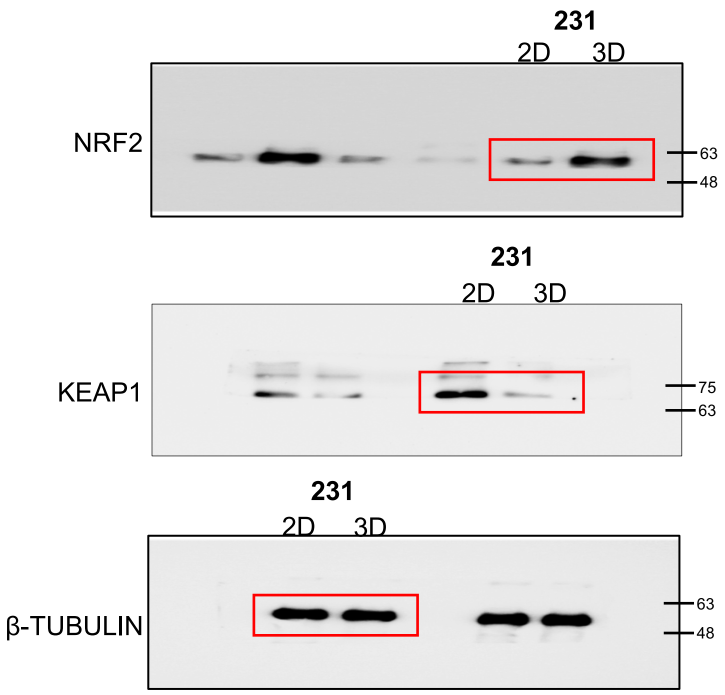

Fig. 2k

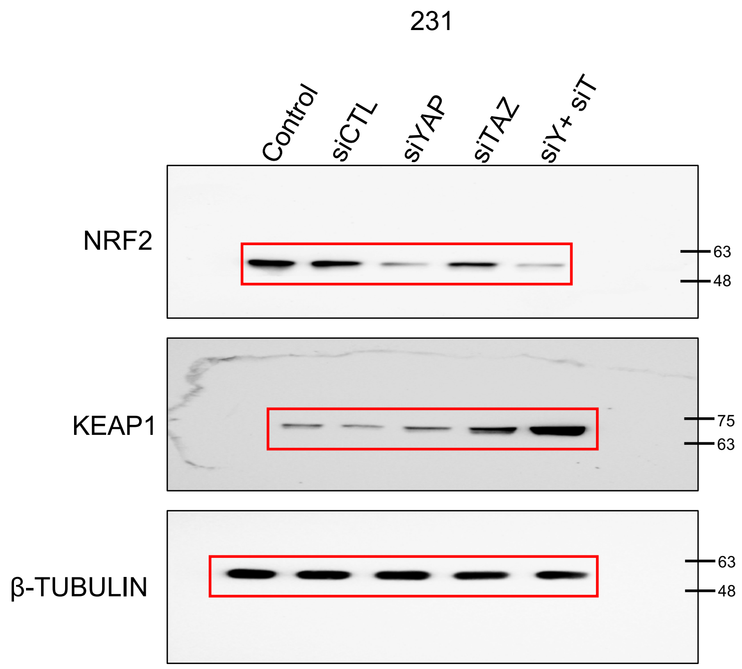

#The results are derived from multiple blots, with constitutive proteins consistently analyzed

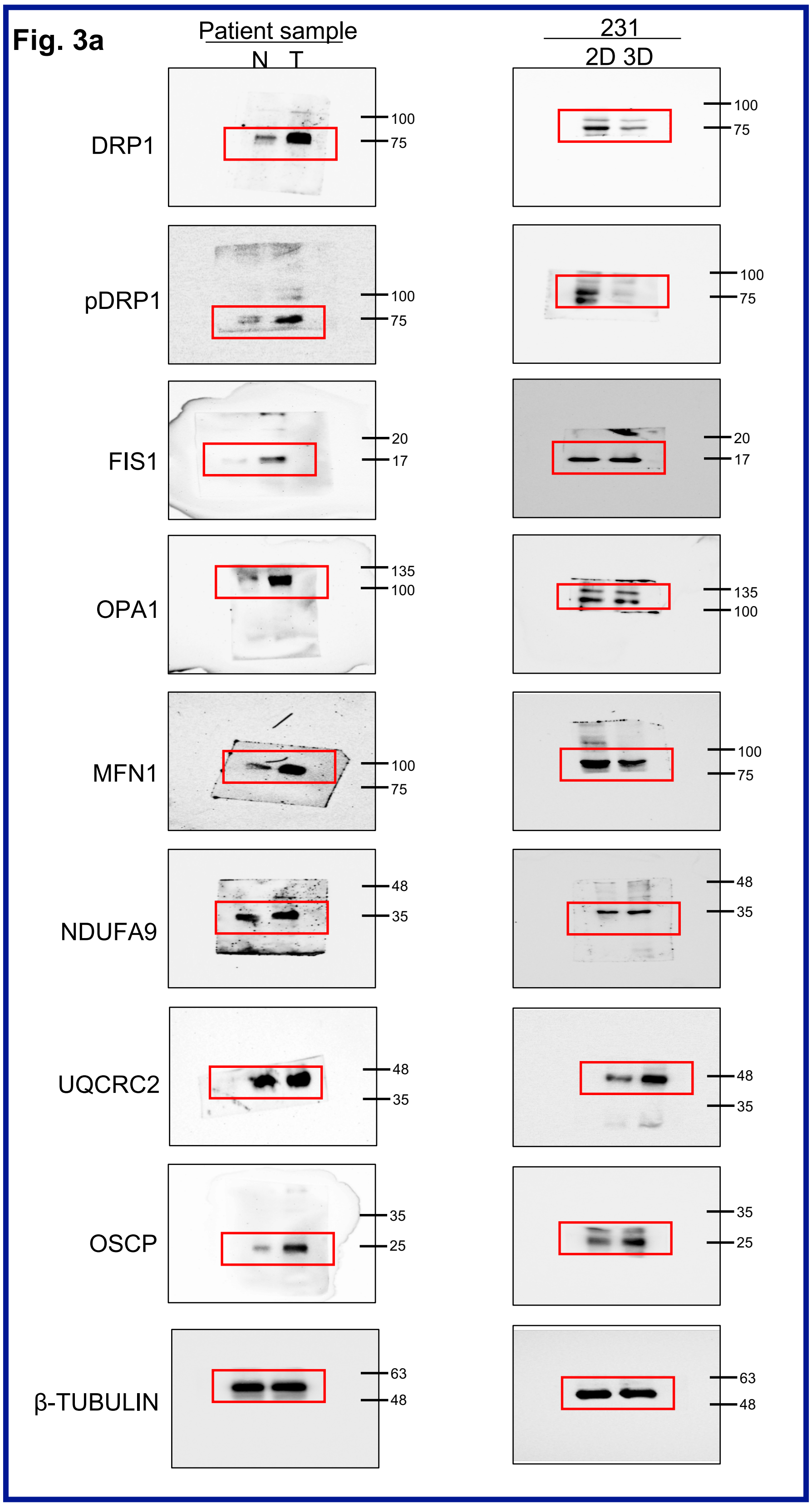

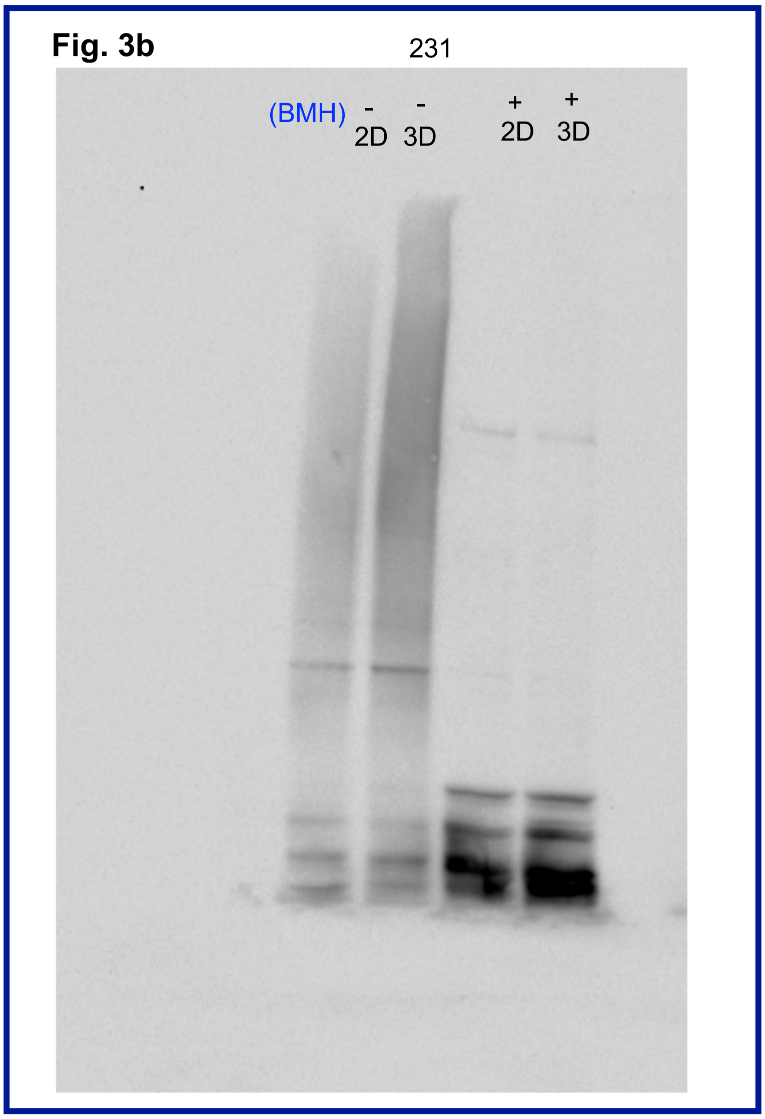

#The results are derived from multiple blots, with constitutive proteins consistently analyzed

Fig. 4a

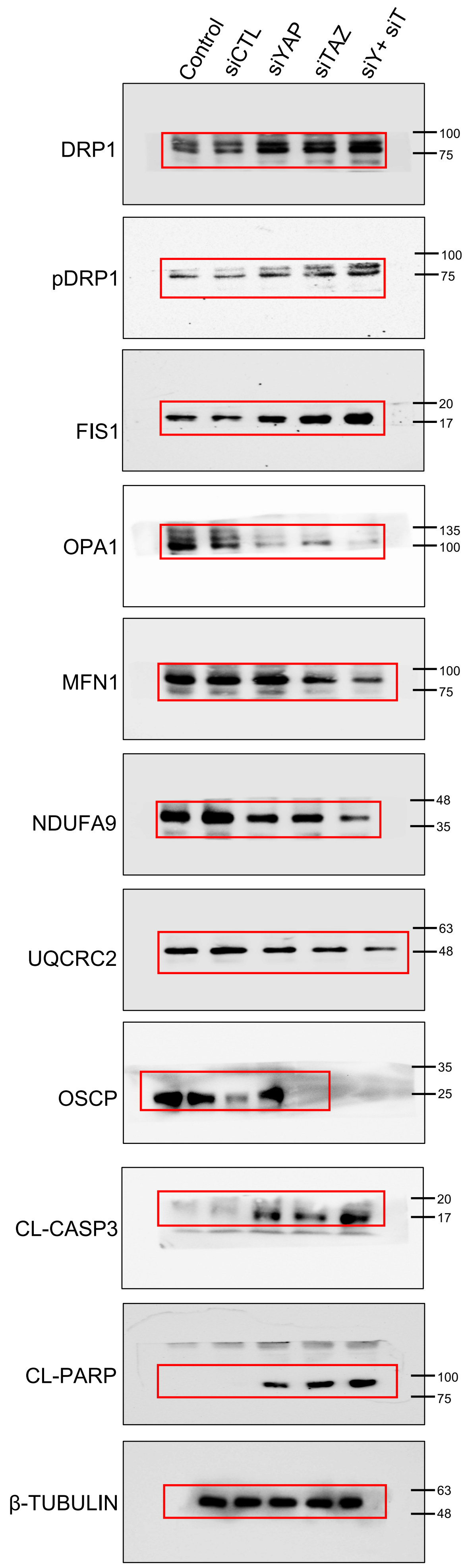

Fig. 4b

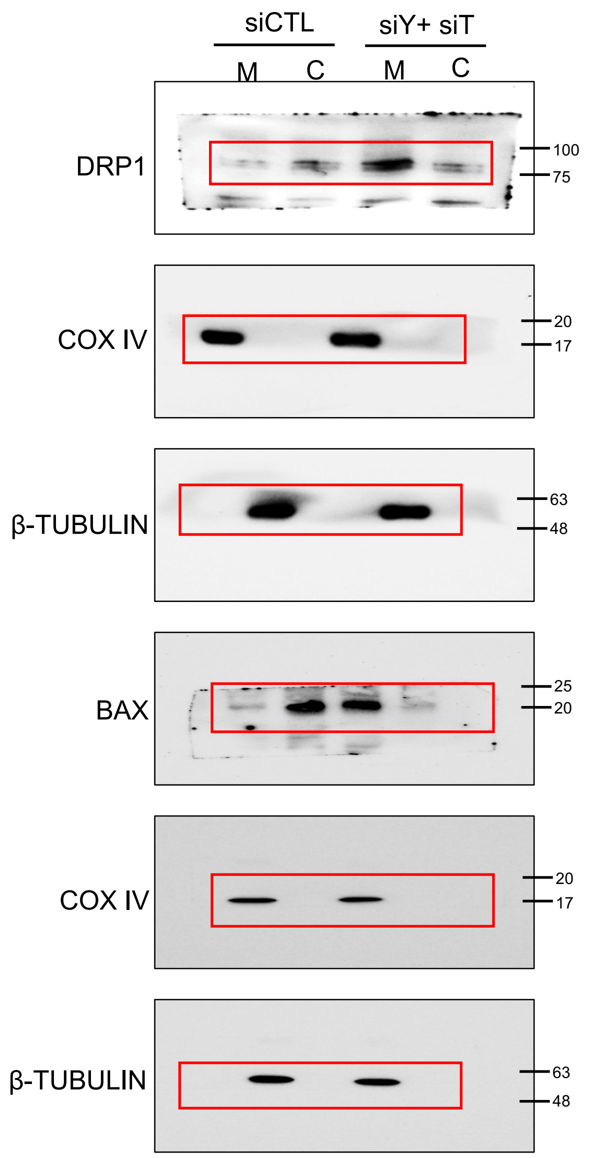

#The results are derived from multiple blots, with constitutive proteins consistently analyzed

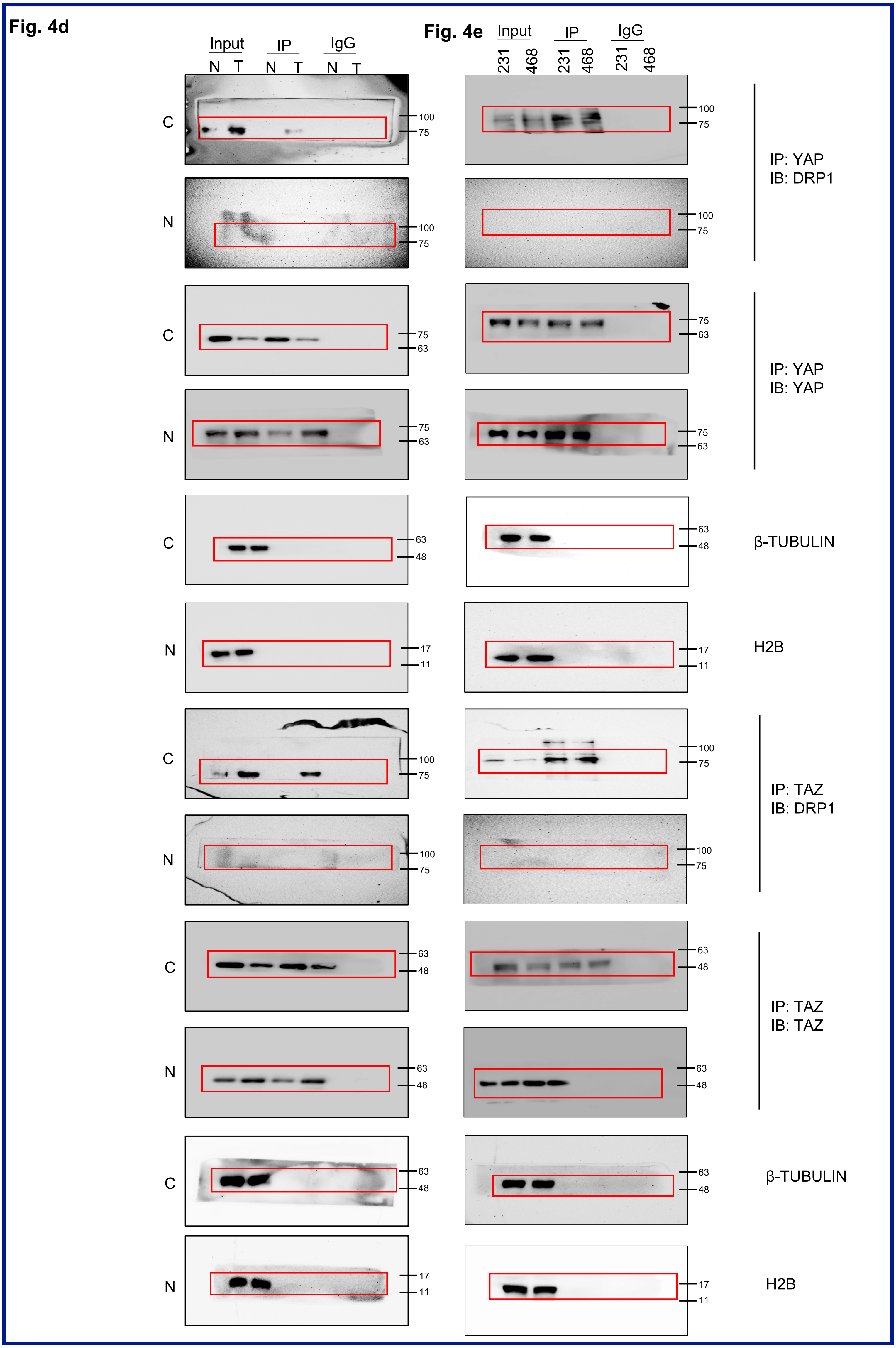

#The results are derived from multiple blots, with constitutive proteins consistently analyzed

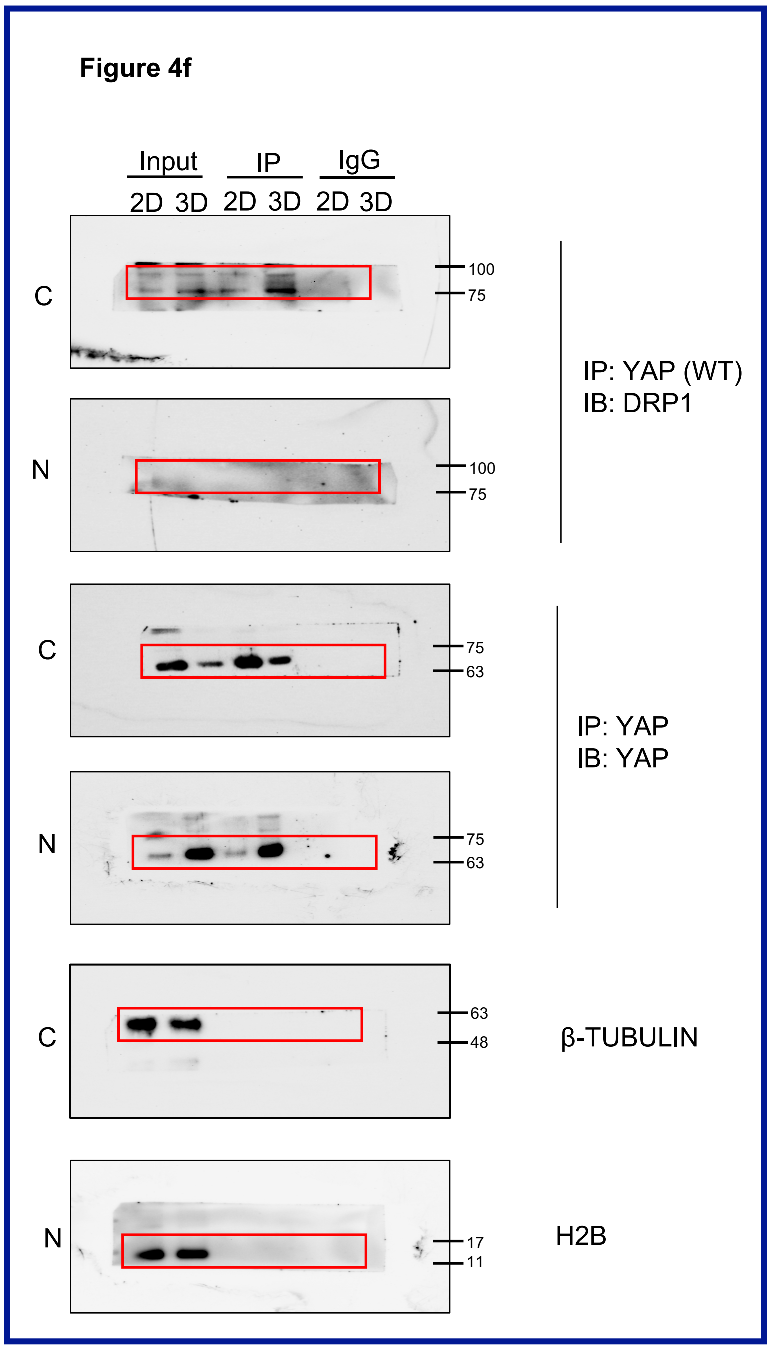

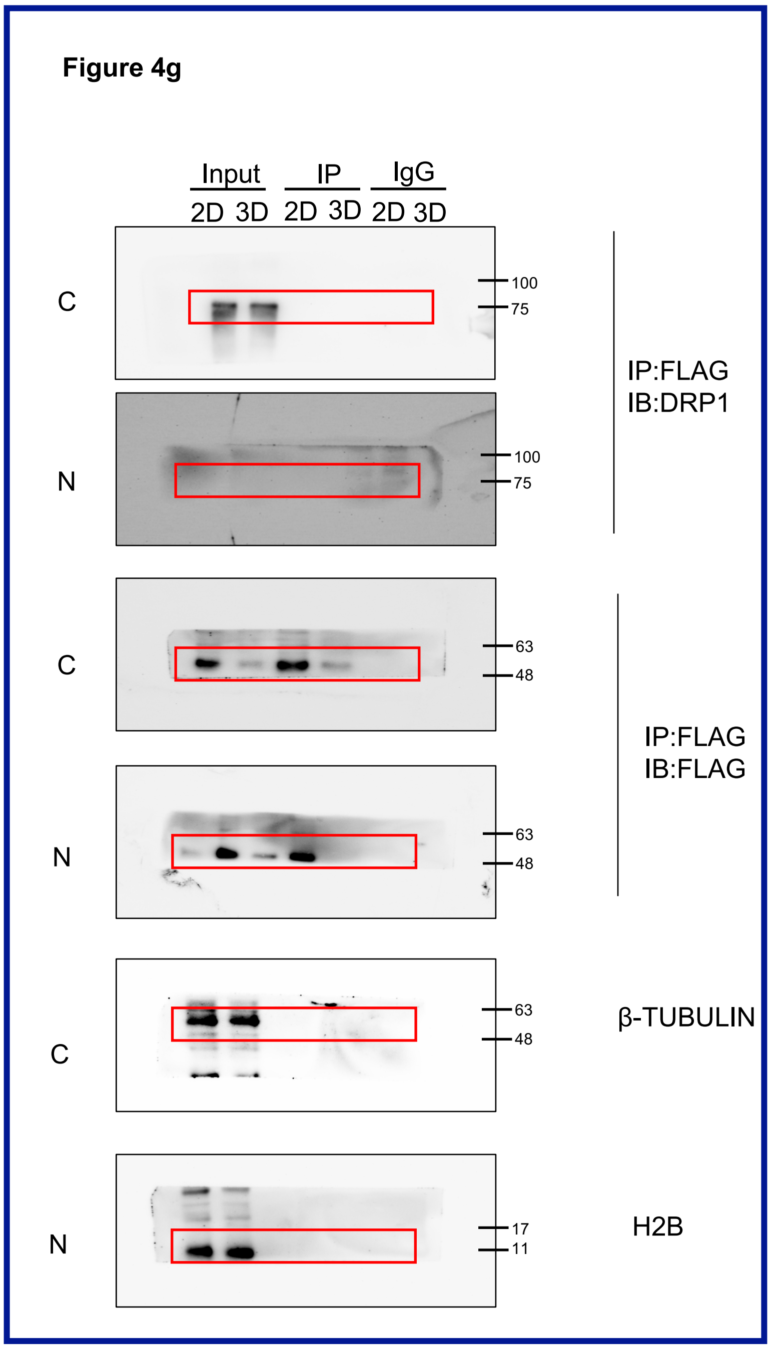

#The results are derived from multiple blots, with constitutive proteins consistently analyzed

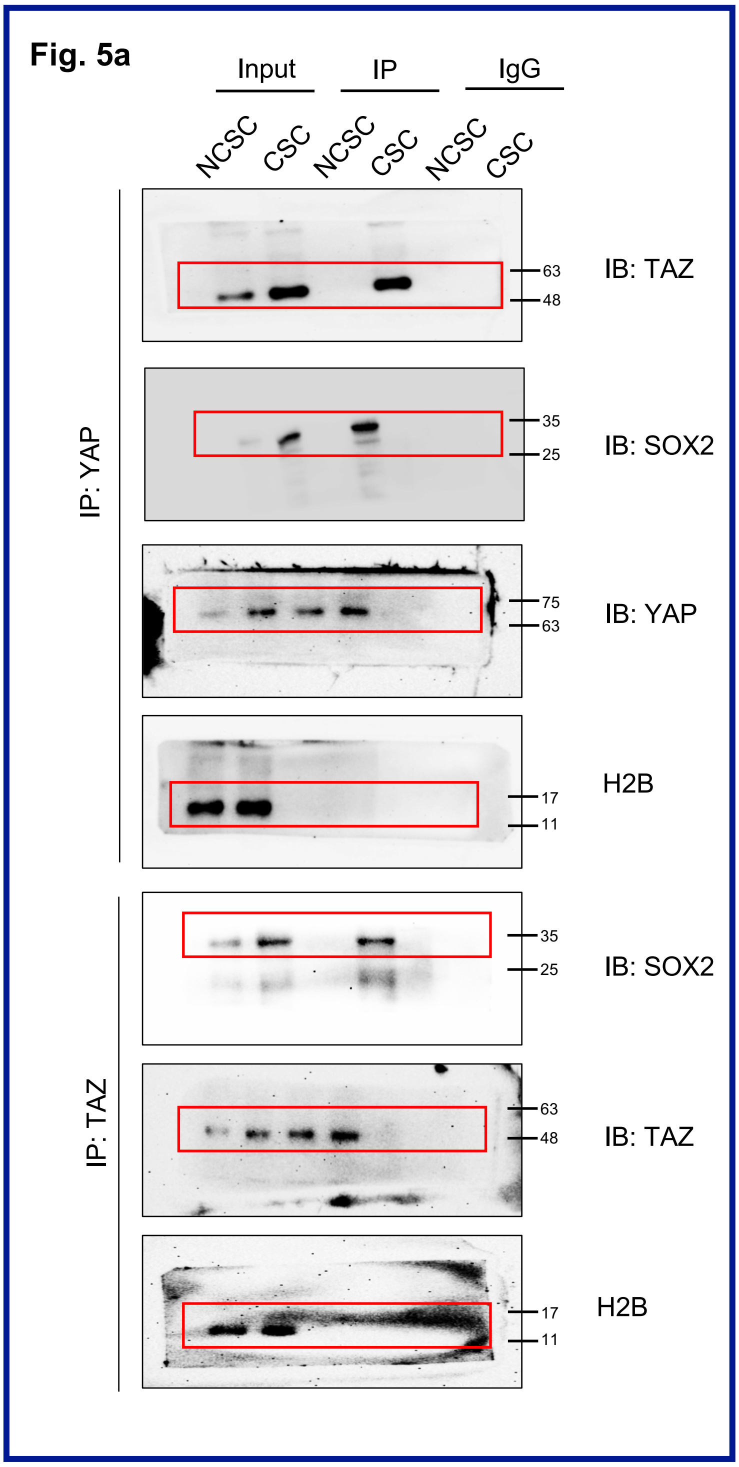

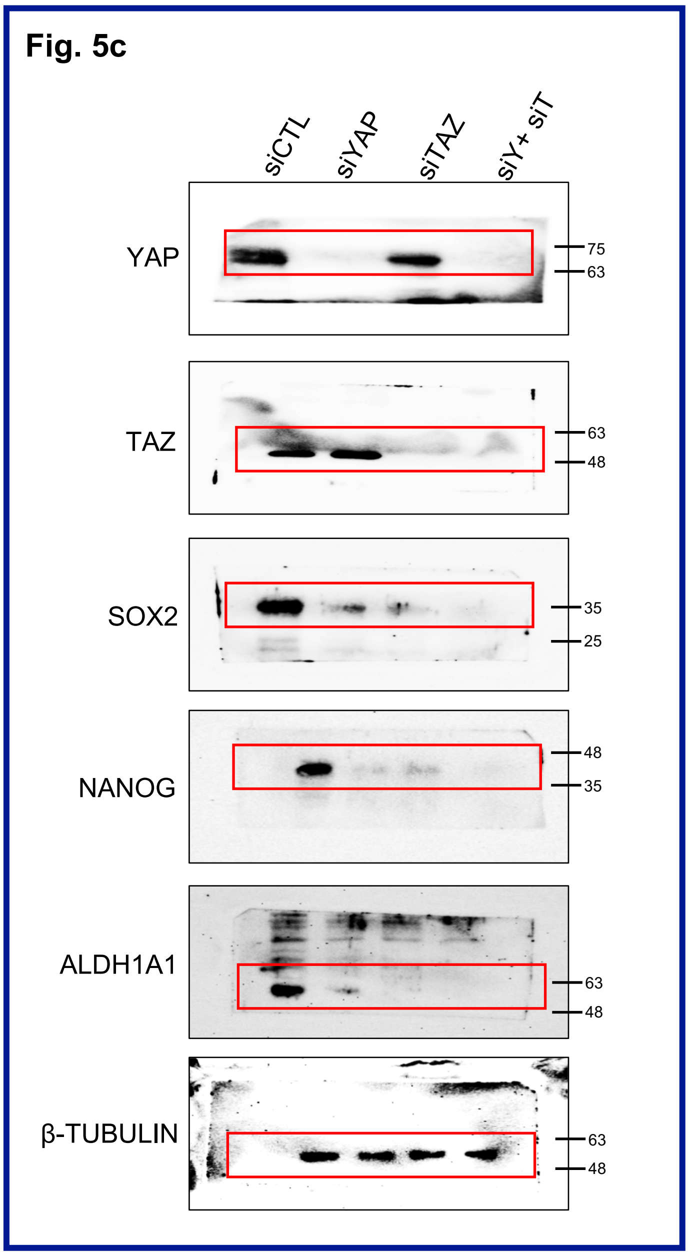

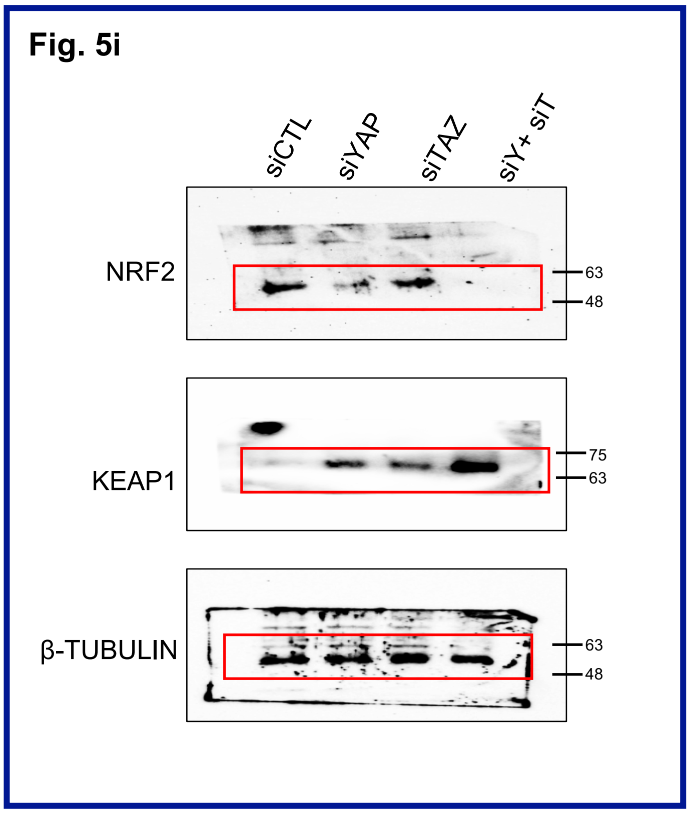

#The results are derived from multiple blots, with constitutive proteins consistently analyzed

Fig. 5l

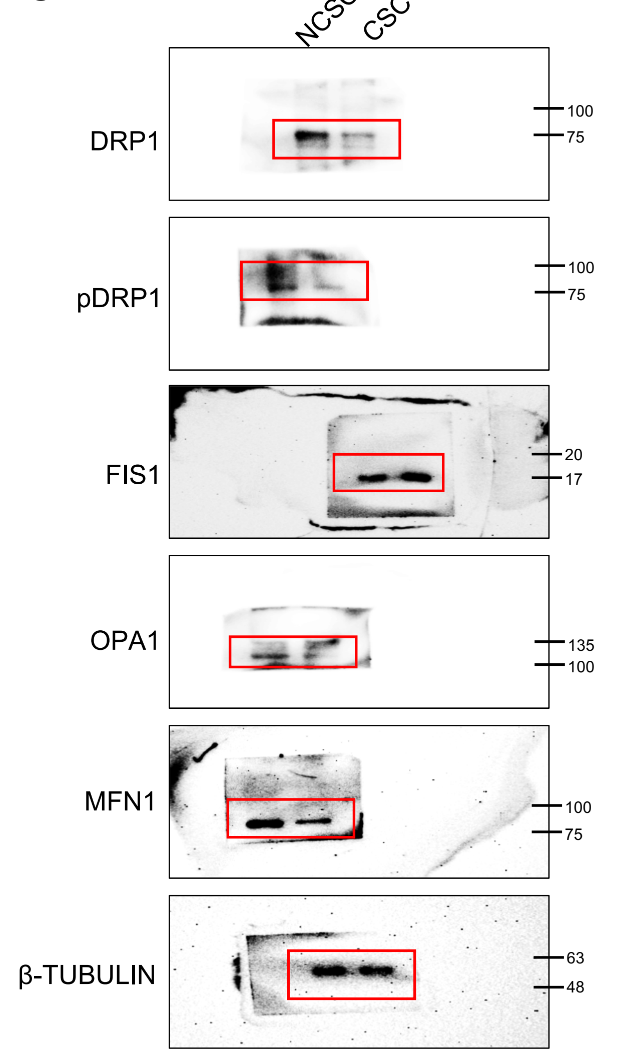

Fig. 5n

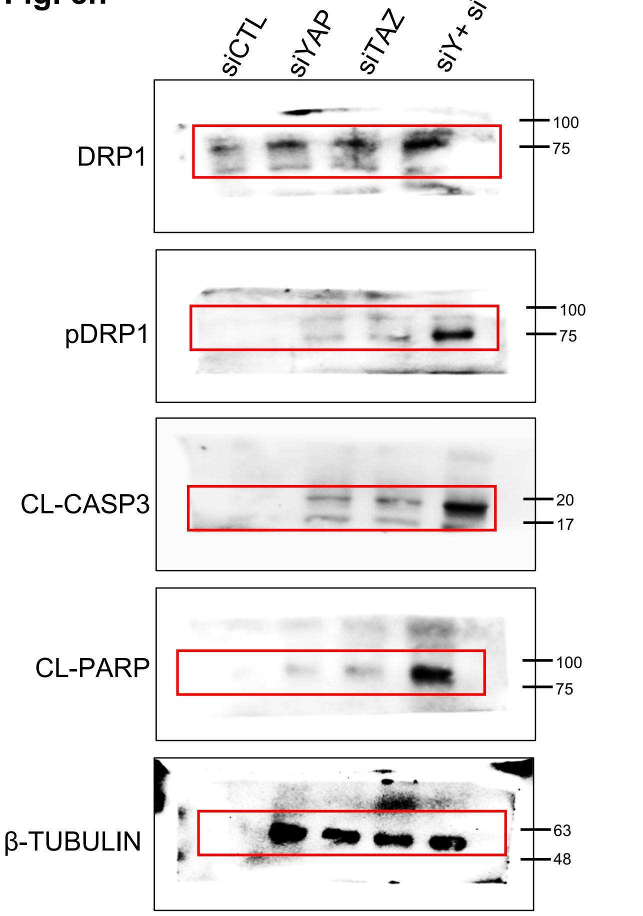

Fig. 5o

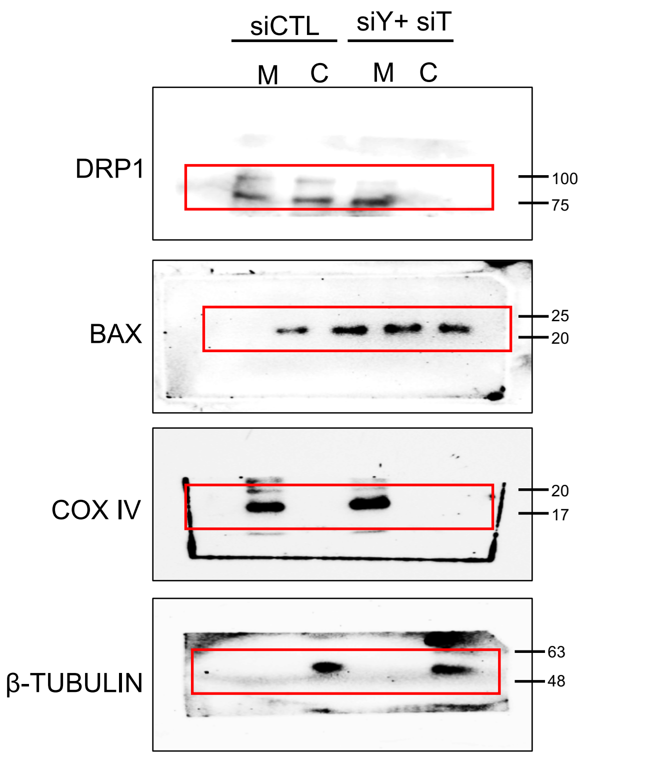

Fig. 5p

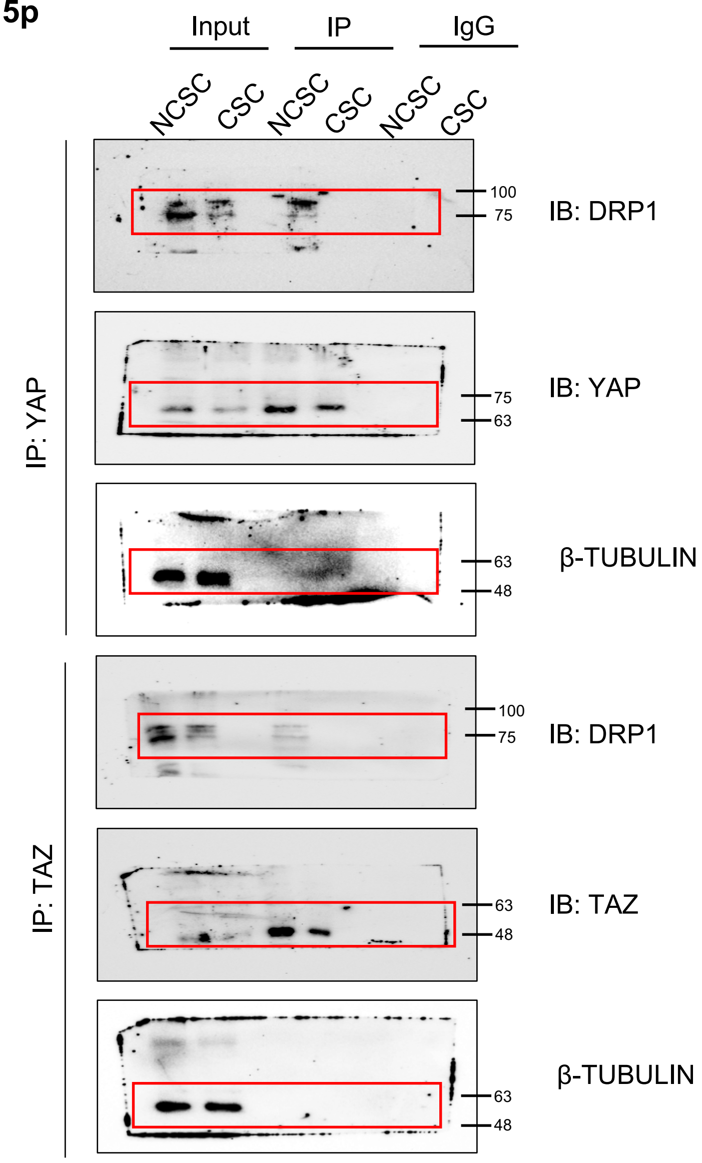

#The results are derived from multiple blots, with constitutive proteins consistently analyzed

Fig. 6b

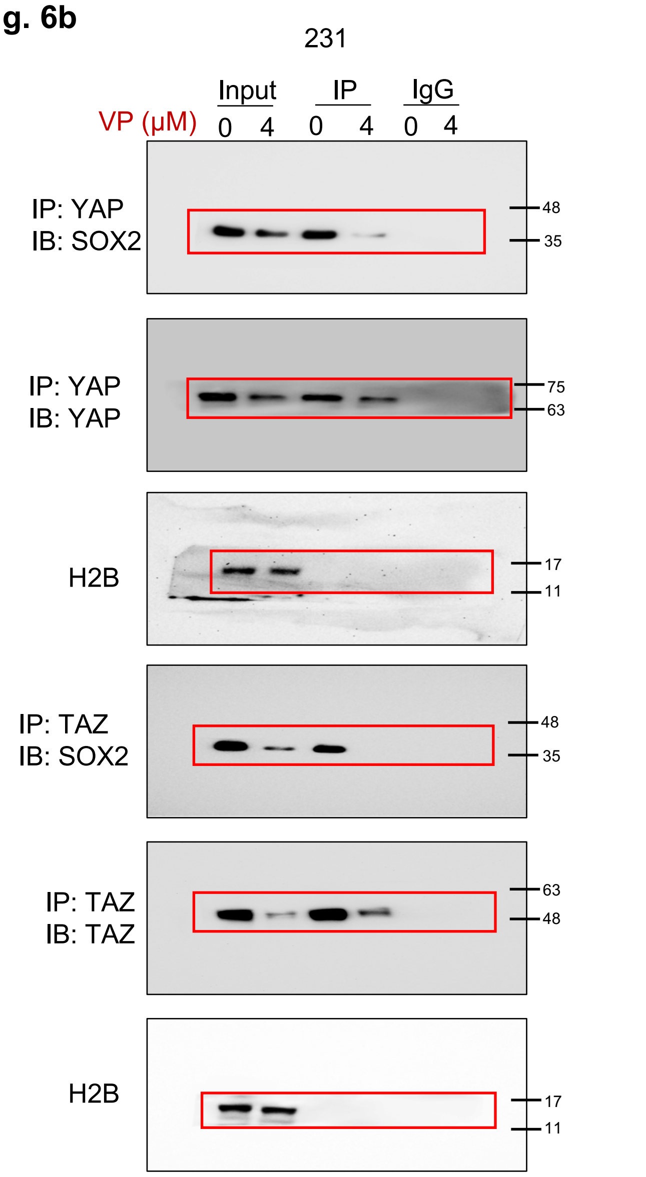

Fig. 7a

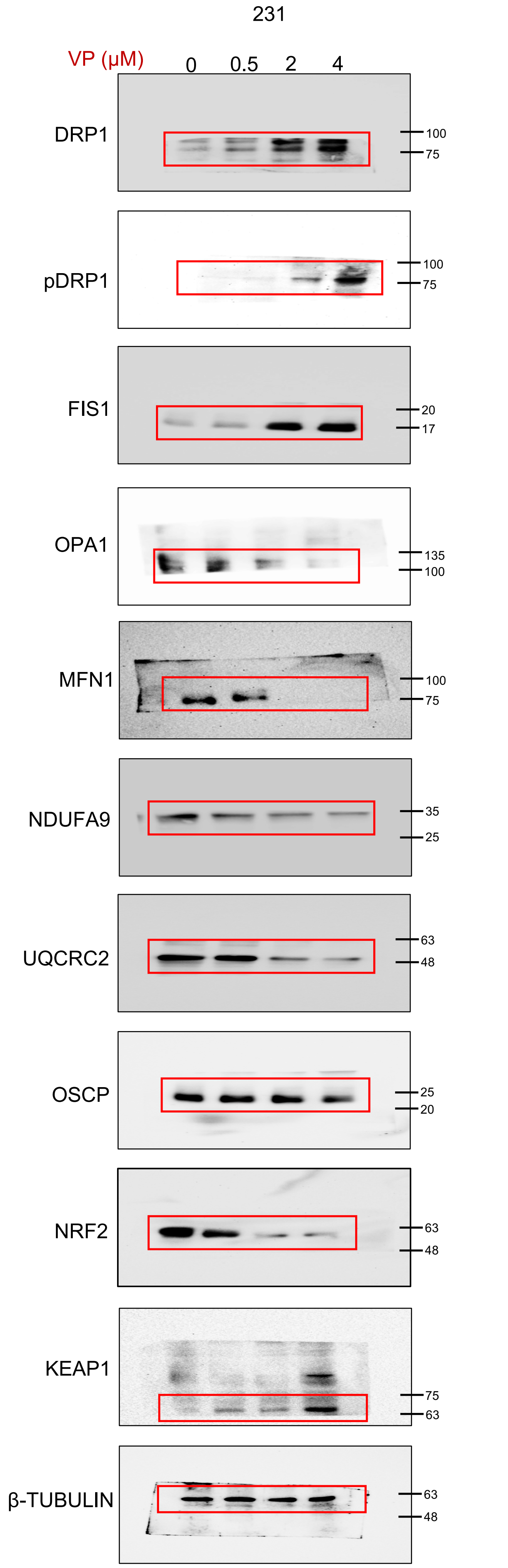

#The results are derived from multiple blots, with constitutive proteins consistently analyzed

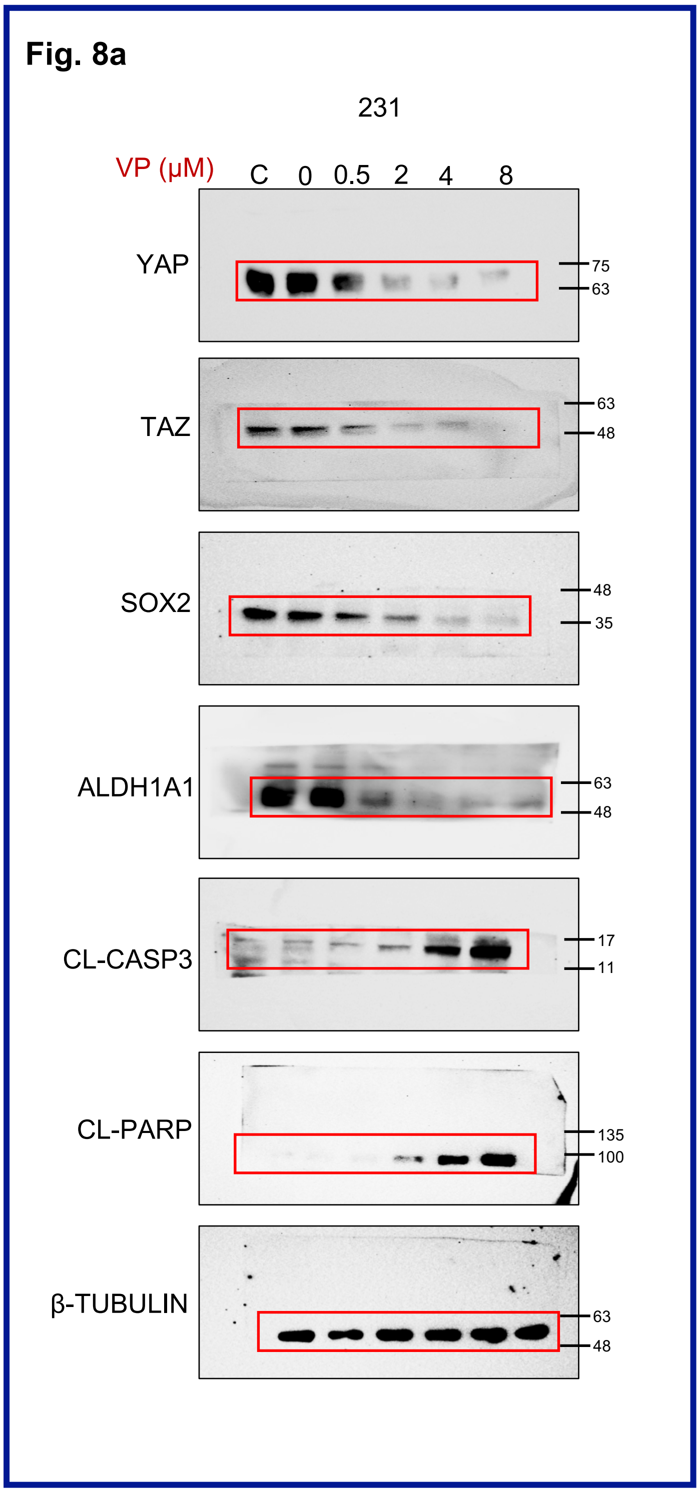

#The results are derived from multiple blots, with constitutive proteins consistently analyzed

Western Blots

Supplementary Figures S1 to S26

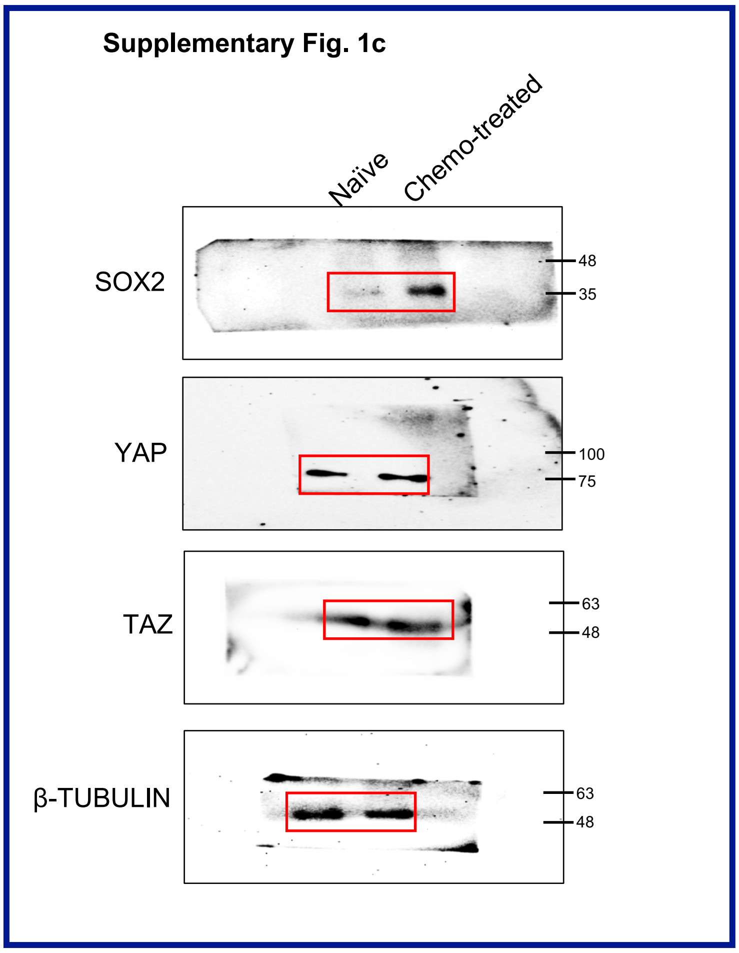

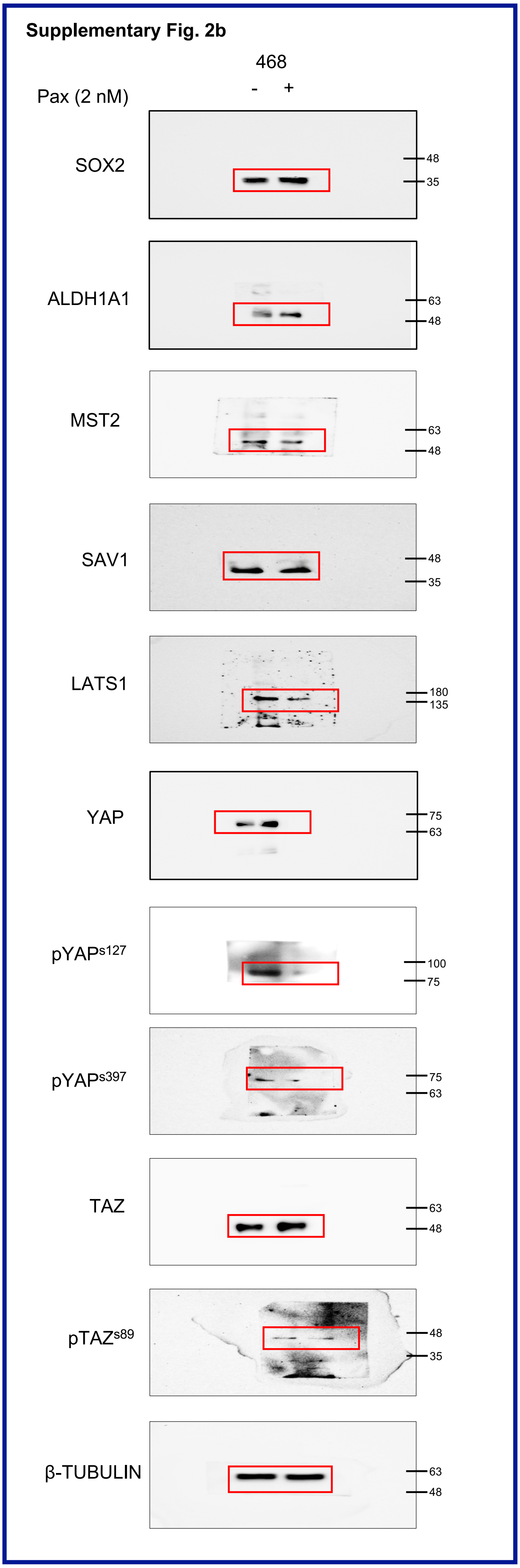

#The results are derived from multiple blots, with constitutive proteins consistently analyzed

Supplementary Figure S2e.

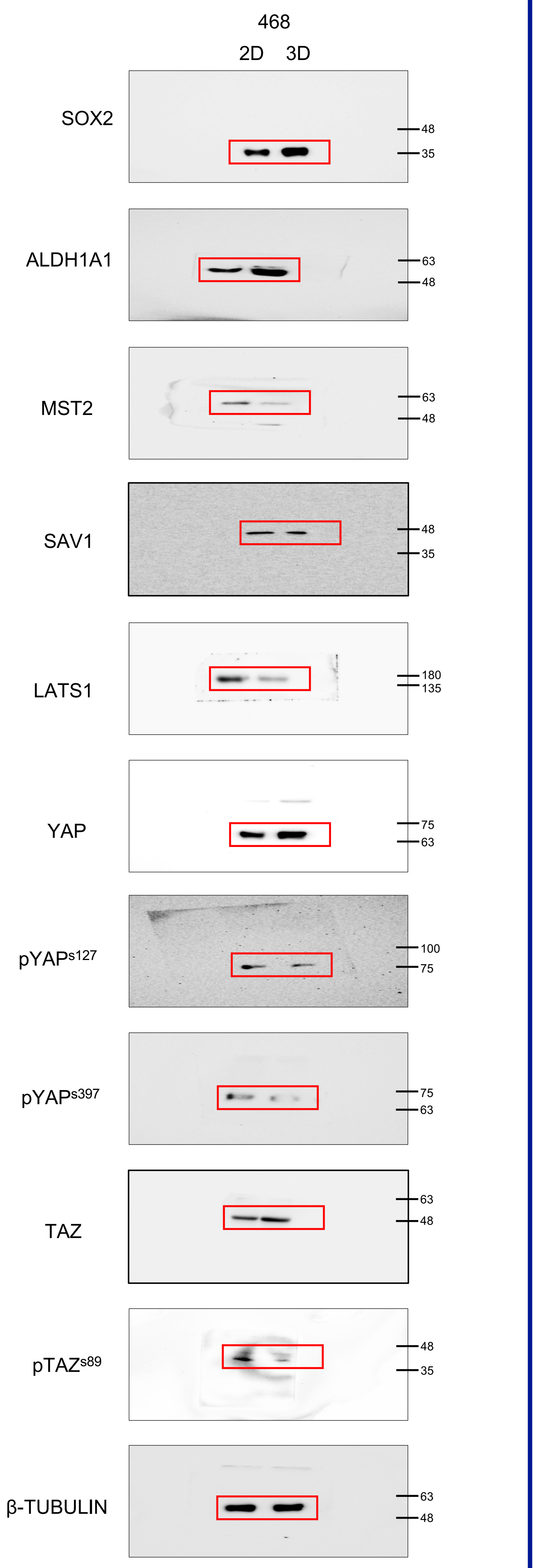

Supplementary Figure S3b.

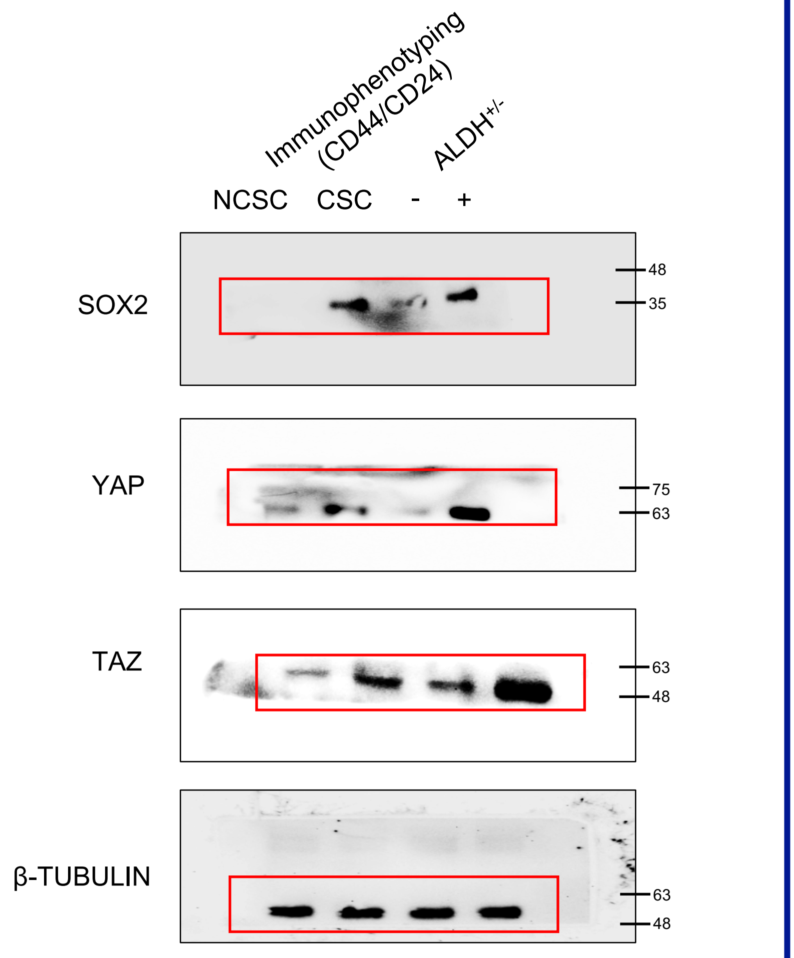

#The results are derived from multiple blots, with constitutive proteins consistently analyzed

Supplementary Figure S4e.

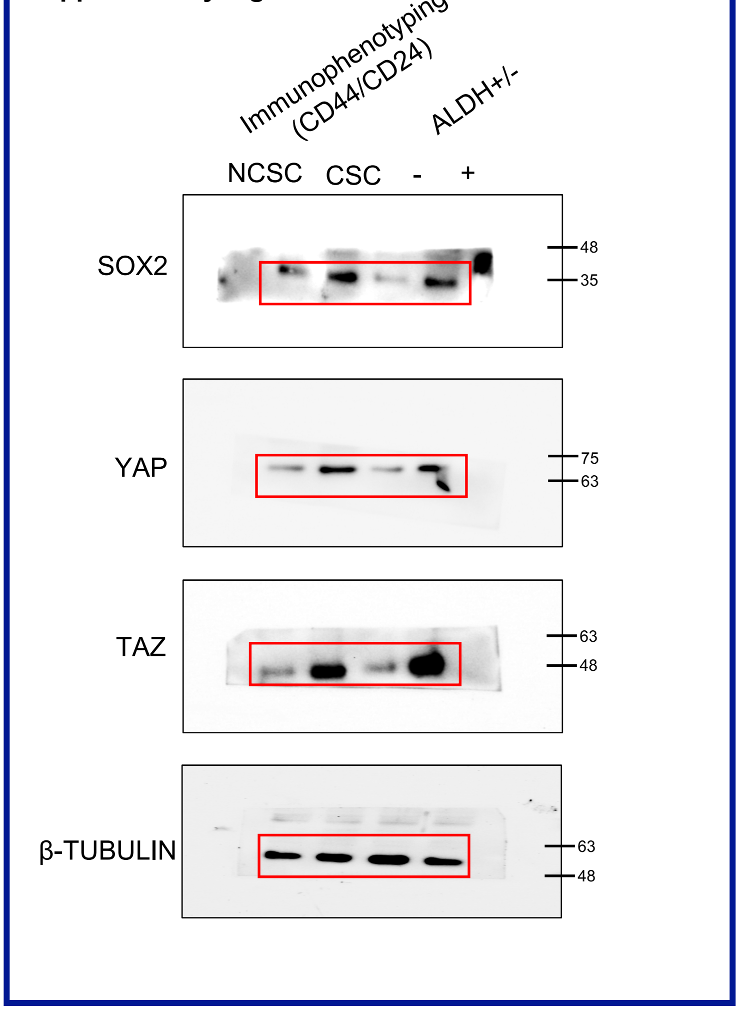

Supplementary Figure S4g.

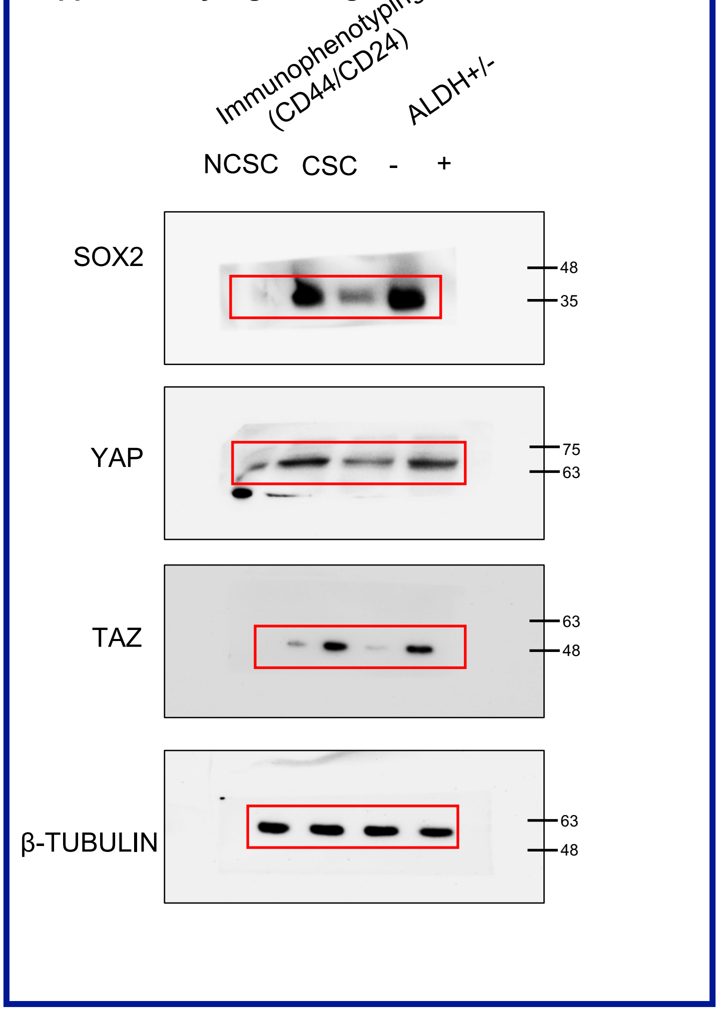

Supplementary Figure S4i.

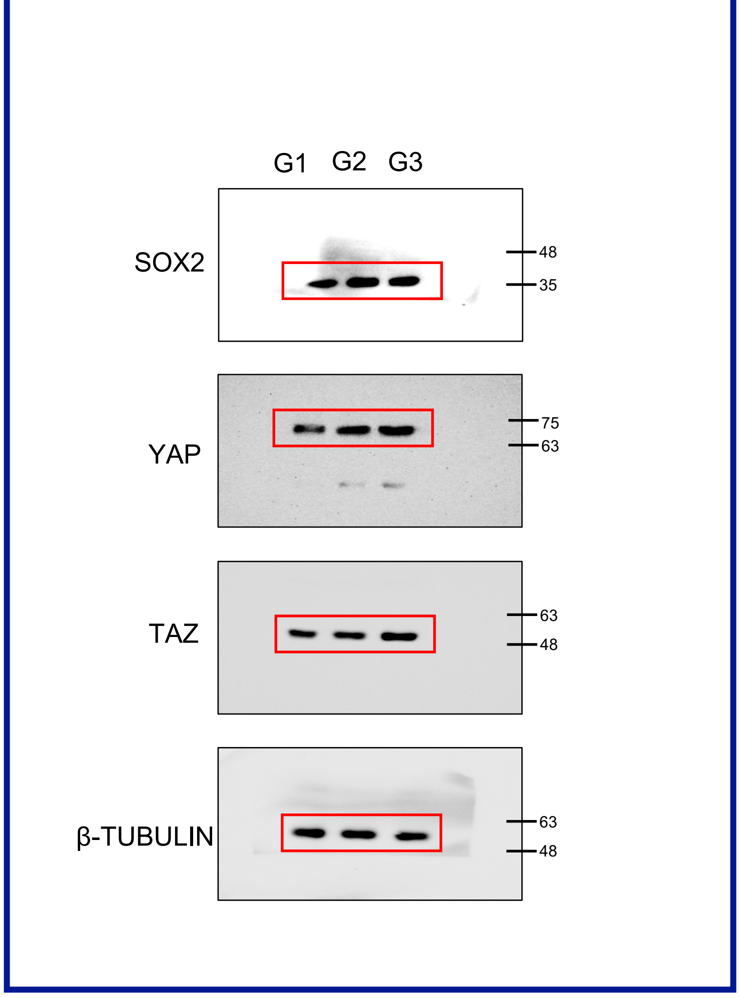

#The results are derived from multiple blots, with constitutive proteins consistently analyzed

Supplementary Figure S11f.

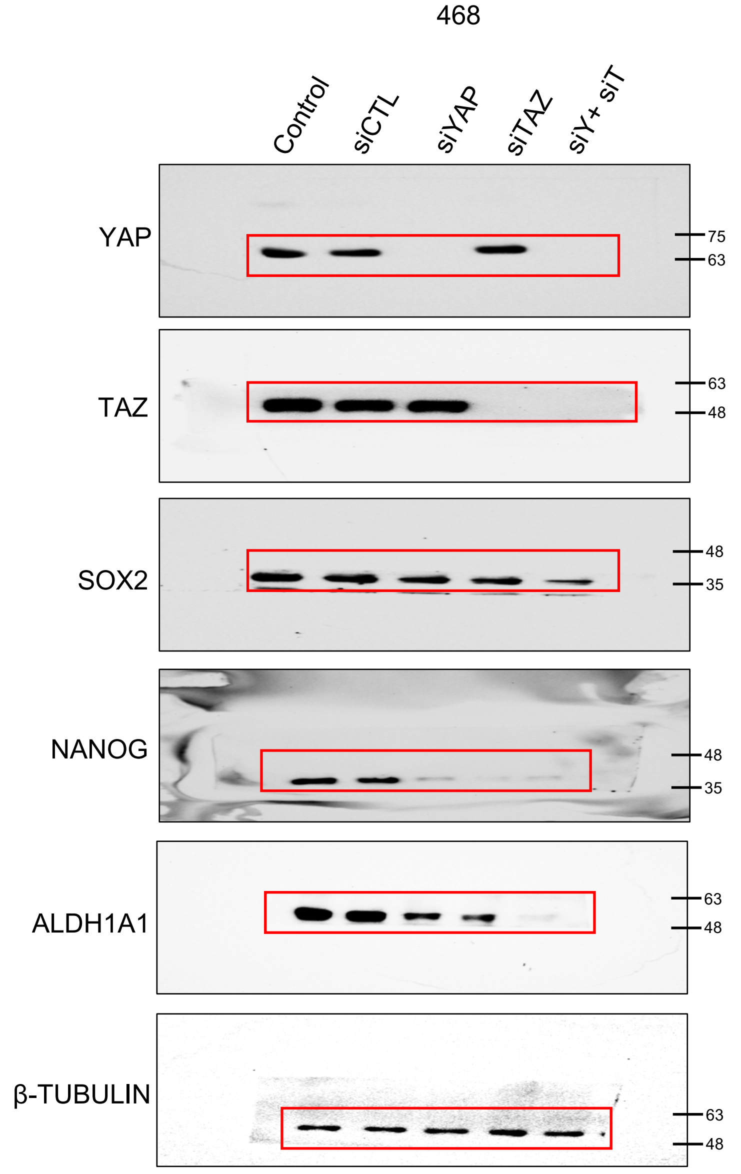

Supplementary Figure S12c.

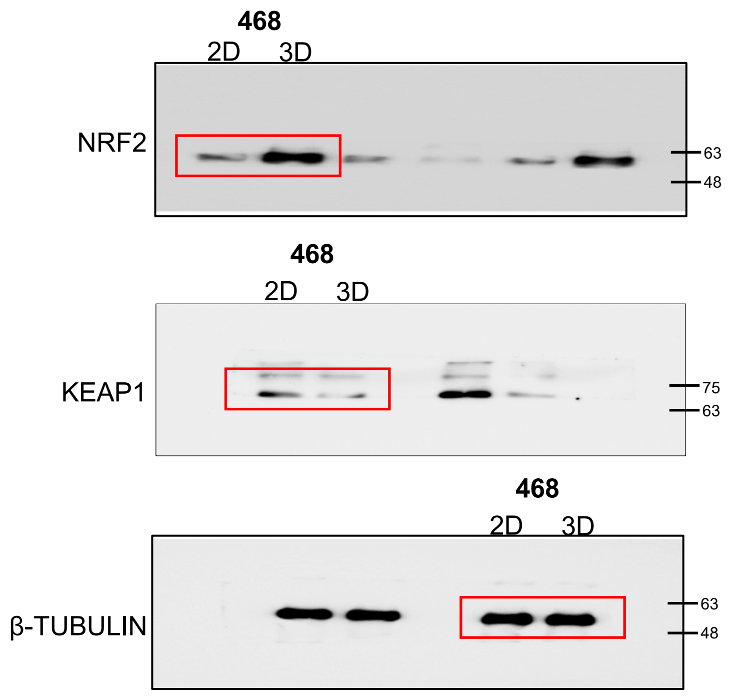

#The results are derived from multiple blots, with constitutive proteins consistently analyzed

Supplementary Figure S12f.

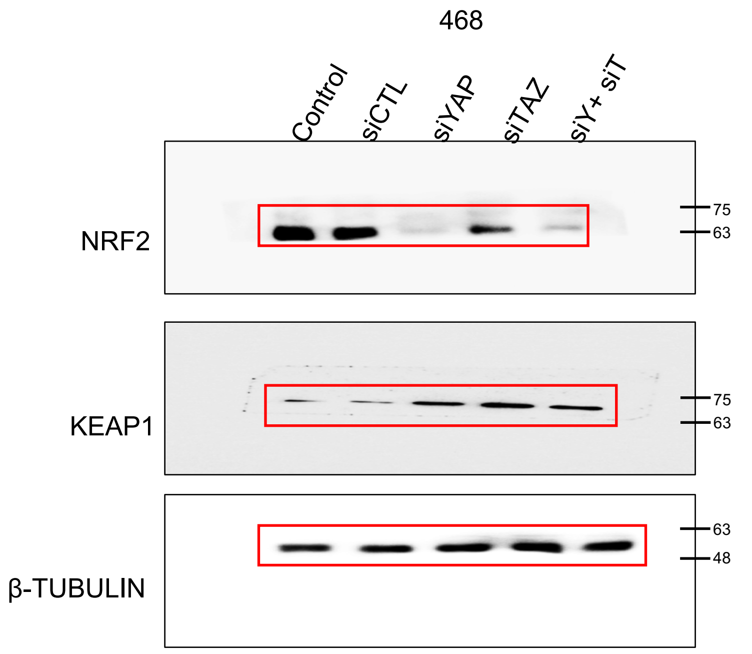

Supplementary Figure S13c.

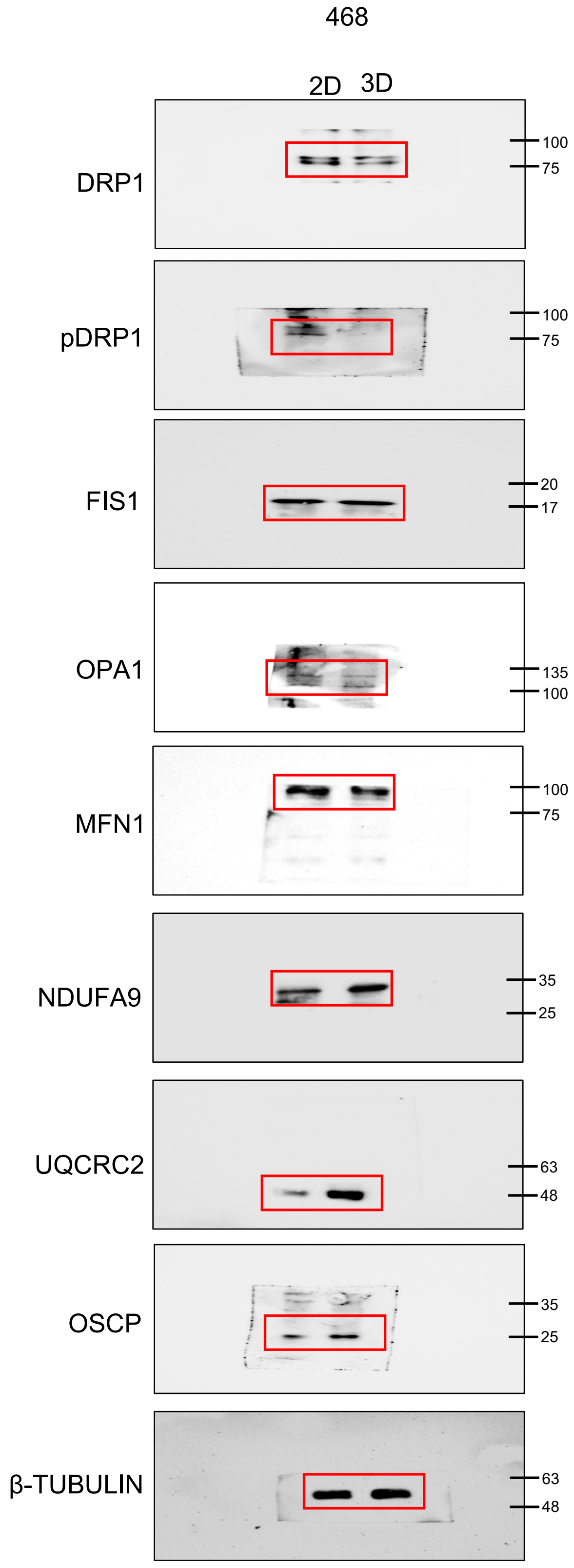

#The results are derived from multiple blots, with constitutive proteins consistently analyzed

Supplementary Figure S14b.

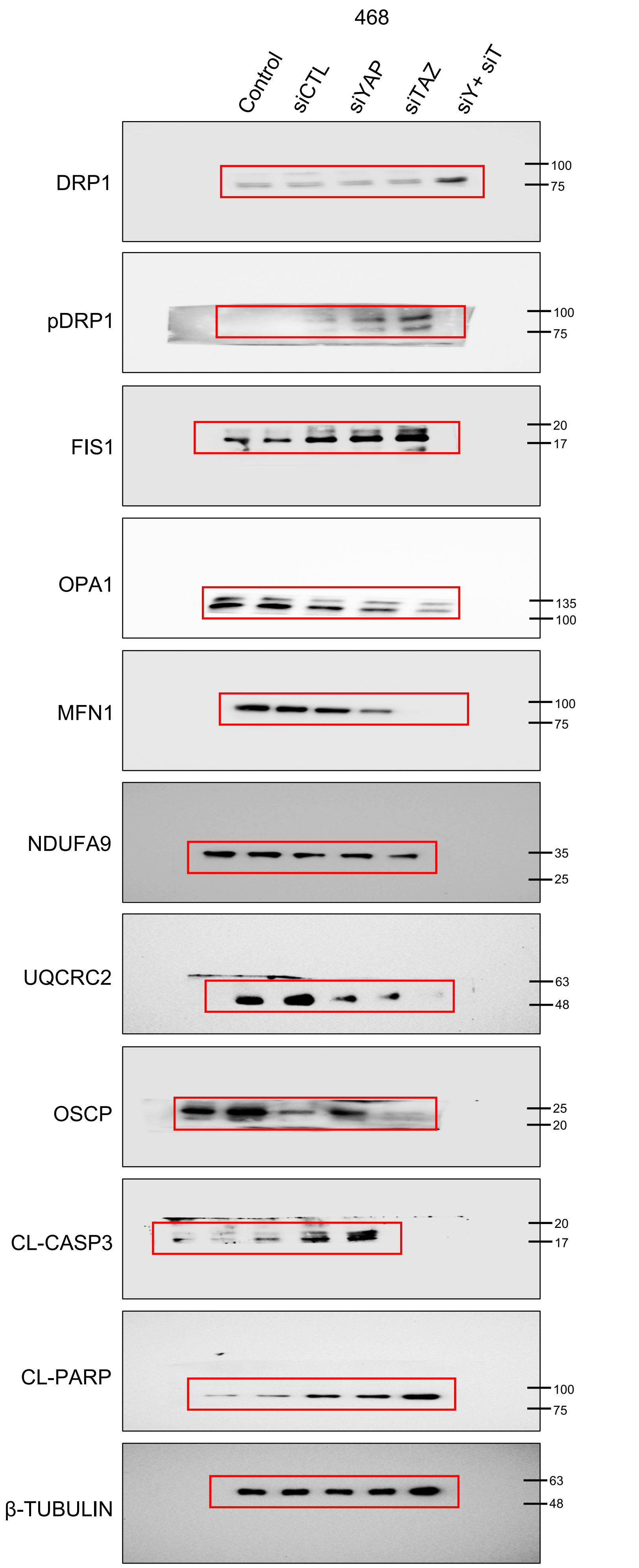

Supplementary Figure S14e.

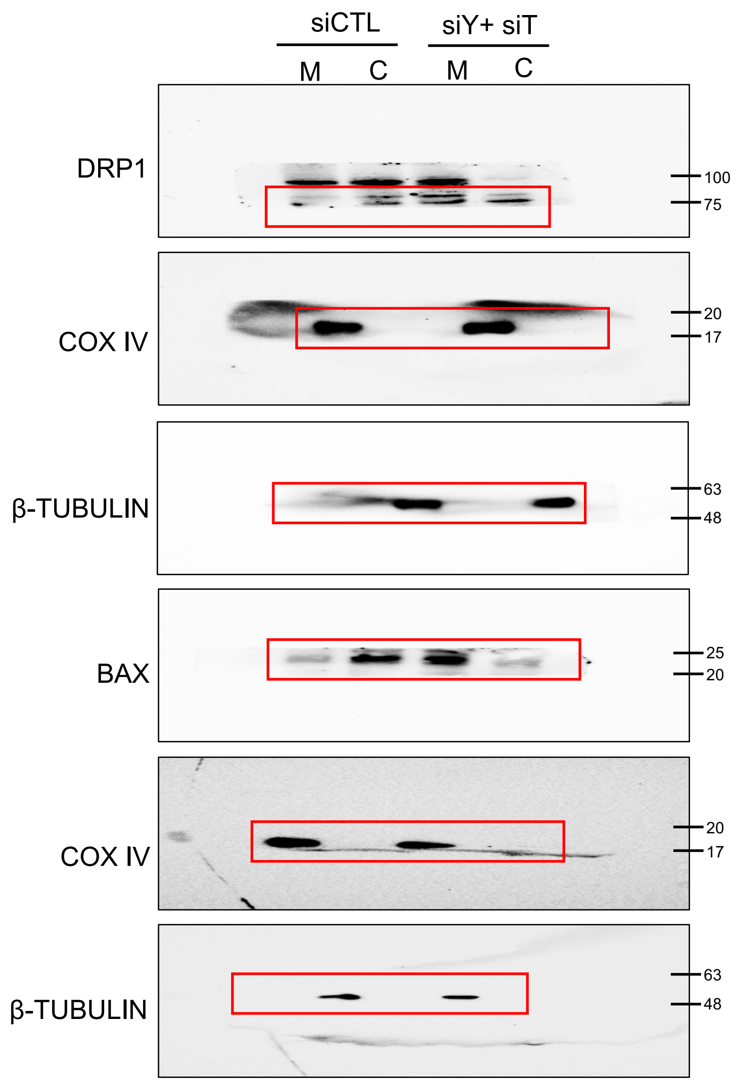

#The results are derived from multiple blots, with constitutive proteins consistently analyzed

Supplementary Figure S25b.

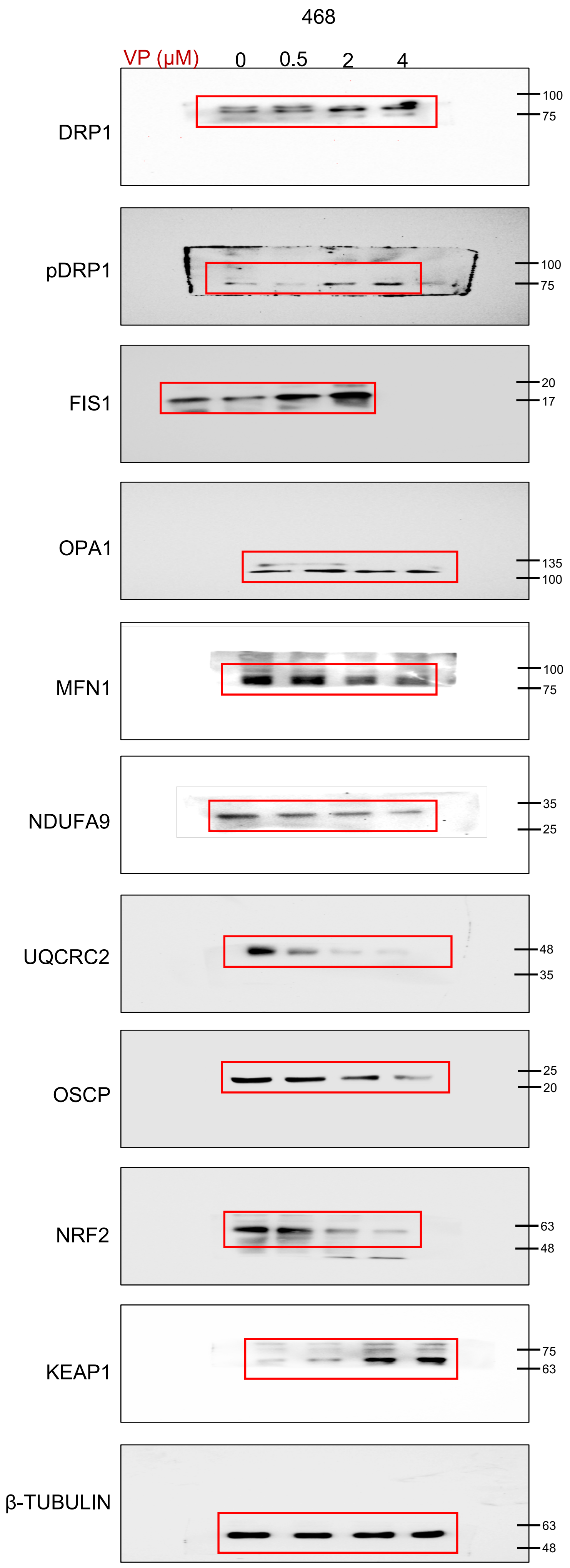

Supplementary Figure S26b.

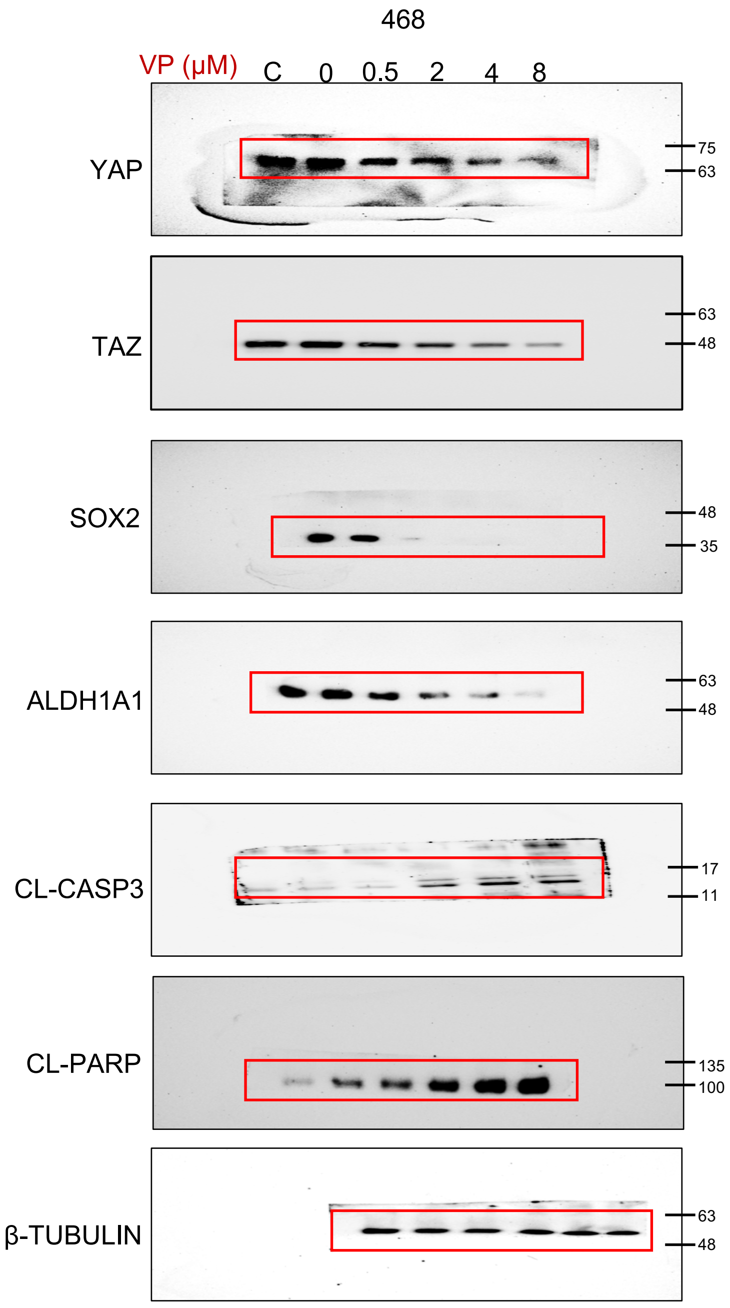

#The results are derived from multiple blots, with constitutive proteins consistently analyzed
